# Late Quaternary dynamics of Arctic biota from ancient environmental genomics

**DOI:** 10.1038/s41586-021-04016-x

**Published:** 2021-10-20

**Authors:** Yucheng Wang, Mikkel Winther Pedersen, Inger Greve Alsos, Bianca De Sanctis, Fernando Racimo, Ana Prohaska, Eric Coissac, Hannah Lois Owens, Marie Kristine Føreid Merkel, Antonio Fernandez-Guerra, Alexandra Rouillard, Youri Lammers, Adriana Alberti, France Denoeud, Daniel Money, Anthony H. Ruter, Hugh McColl, Nicolaj Krog Larsen, Anna A. Cherezova, Mary E. Edwards, Grigory B. Fedorov, James Haile, Ludovic Orlando, Lasse Vinner, Thorfinn Sand Korneliussen, David W. Beilman, Anders A. Bjørk, Jialu Cao, Christoph Dockter, Julie Esdale, Galina Gusarova, Kristian K. Kjeldsen, Jan Mangerud, Jeffrey T. Rasic, Birgitte Skadhauge, John Inge Svendsen, Alexei Tikhonov, Patrick Wincker, Yingchun Xing, Yubin Zhang, Duane G. Froese, Carsten Rahbek, David Nogues Bravo, Philip B. Holden, Neil R. Edwards, Richard Durbin, David J. Meltzer, Kurt H. Kjær, Per Möller, Eske Willerslev

**Affiliations:** 1 Department of Zoology, University of Cambridge, Cambridge, UK; 2 Lundbeck Foundation GeoGenetics Centre, GLOBE Institute, University of Copenhagen, Copenhagen, Denmark; 3 The Arctic University Museum of Norway, UiT— The Arctic University of Norway, Tromsø, Norway; 4 Department of Genetics, University of Cambridge, Cambridge, UK; 5 Université Grenoble Alpes, Université Savoie Mont Blanc, CNRS, LECA, Grenoble, France; 6 Center for Macroecology, Evolution and Climate, GLOBE Institute, University of Copenhagen, Copenhagen, Denmark; 7 Department of Geosciences, UiT—The Arctic University of Norway, Tromsø, Norway; 8 Université Paris-Saclay, CEA, CNRS, Institute for Integrative Biology of the Cell (I2BC), Gif-sur-Yvette, France; 9 Génomique Métabolique, Genoscope, Institut François Jacob, CEA, CNRS, Université Evry, Université Paris-Saclay, Evry, France; 10 Institute of Earth Sciences, St Petersburg State University, St Petersburg, Russia; 11 Arctic and Antarctic Research Institute, St Petersburg, Russia; 12 School of Geography and Environmental Science, University of Southampton, Southampton, UK; 13 Alaska Quaternary Center, University of Alaska Fairbanks, Fairbanks, AK USA; 14 Centre d’Anthropobiologie et de Génomique de Toulouse, Université Paul Sabatier, Faculté de Médecine Purpan, Toulouse, France; 15 National Research University, Higher School of Economics, Moscow, Russia; 16 Department of Geography and Environment, University of Hawaii, Honolulu, HI USA; 17 Department of Geosciences and Natural Resource Management, University of Copenhagen, Copenhagen, Denmark; 18 Carlsberg Research Laboratory, Copenhagen, Denmark; 19 Center for Environmental Management of Military Lands, Colorado State University, Fort Collins, CO USA; 20 Faculty of Biology, St Petersburg State University, St Petersburg, Russia; 21 Department of Glaciology and Climate, Geological Survey of Denmark and Greenland, Copenhagen, Denmark; 22 Department of Earth Science, University of Bergen, Bergen, Norway; 23 Bjerknes Centre for Climate Research, Bergen, Norway; 24 US National Park Service, Gates of the Arctic National Park and Preserve, Fairbanks, AK USA; 25 Zoological Institute, , Russian Academy of Sciences, St Petersburg, Russia; 26 Resource and Environmental Research Center, Chinese Academy of Fishery Sciences, Beijing, China; 27 College of Plant Science, Jilin University, Changchun, China; 28 Department of Earth and Atmospheric Sciences, University of Alberta, Edmonton, Alberta Canada; 29 Center for Global Mountain Biodiversity, GLOBE Institute, University of Copenhagen, Copenhagen, Denmark; 30 School of Environment, Earth and Ecosystem Sciences, The Open University, Milton Keynes, UK; 31 Department of Anthropology, Southern Methodist University, Dallas, TX USA; 32 Department of Geology, Quaternary Sciences, Lund University, Lund, Sweden; 33 Wellcome Trust Sanger Institute, Wellcome Genome Campus, Cambridge, UK; 34 MARUM, University of Bremen, Bremen, Germany

**Keywords:** Ecological networks, Palaeoecology, Metagenomics, Next-generation sequencing, Climate-change ecology

## Abstract

During the last glacial–interglacial cycle, Arctic biotas experienced substantial climatic changes, yet the nature, extent and rate of their responses are not fully understood^1–8^. Here we report a large-scale environmental DNA metagenomic study of ancient plant and mammal communities, analysing 535 permafrost and lake sediment samples from across the Arctic spanning the past 50,000 years. Furthermore, we present 1,541 contemporary plant genome assemblies that were generated as reference sequences. Our study provides several insights into the long-term dynamics of the Arctic biota at the circumpolar and regional scales. Our key findings include: (1) a relatively homogeneous steppe–tundra flora dominated the Arctic during the Last Glacial Maximum, followed by regional divergence of vegetation during the Holocene epoch; (2) certain grazing animals consistently co-occurred in space and time; (3) humans appear to have been a minor factor in driving animal distributions; (4) higher effective precipitation, as well as an increase in the proportion of wetland plants, show negative effects on animal diversity; (5) the persistence of the steppe–tundra vegetation in northern Siberia enabled the late survival of several now-extinct megafauna species, including the woolly mammoth until 3.9 ± 0.2 thousand years ago (ka) and the woolly rhinoceros until 9.8 ± 0.2 ka; and (6) phylogenetic analysis of mammoth environmental DNA reveals a previously unsampled mitochondrial lineage. Our findings highlight the power of ancient environmental metagenomics analyses to advance understanding of population histories and long-term ecological dynamics.

## Abstract (web summary)

A large-scale metagenomic analysis of plant and mammal environmental DNA reveals complex ecological changes across the circumpolar region over the past 50,000 years, as biota responded to changing climates, culminating in the postglacial extinction of large mammals and emergence of modern ecosystems.

## Main

Climate changes are amplified at high latitudes and have pronounced effects on Arctic ecosystems^1^. Their effects on Arctic plant and animal communities, as well as the human populations who are dependent on them, would have been especially pronounced during the extremely cold and arid Last Glacial Maximum (LGM) (26.5–19 ka)^2^ and later during the rapid warming that preceded the Holocene. However, precisely what those effects were, and how they played out across the Arctic, are not fully understood. These dynamics were further complicated by differences in the timing and extent of glaciation in different regions across this vast and topographically complex landscape. Previous studies based on pollen and plant macrofossils have documented substantial spatiotemporal variations in Arctic vegetation over the past 50,000 years (50 kyr)^1,3^, yet it continues to be debated how climatic changes during this period affected plant communities in different regions of the Arctic, and how changes in climate and vegetation may have affected large mammals (that is, megafauna)^4–6^. Skeletal remains show that several megafaunal species, including woolly mammoth (*Mammuthus primigenius*), woolly rhinoceros (*Coelodonta antiquitatis*), steppe bison (*Bison priscus*) and horse (*Equus* spp.), were abundant in the Arctic during the Pleistocene epoch, but are thought to have become regionally or globally extinct by the onset of the Holocene^4,5^. However, the precise timing of megafaunal extinctions, and whether and to what extent some of these taxa survived into the Holocene, is uncertain. Similarly, the contribution of various abiotic and biotic drivers to the extinction process of different taxa remains an open question^7,8^.

To address these knowledge gaps, we performed a metagenomics analysis of ancient environmental DNA (eDNA) of plants and animals recovered from sediments from sites distributed across much of the Arctic covering the past 50 kyr. Relative to other palaeoecological proxies (such as pollen and macrofossils), ancient eDNA offers distinct advantages—including greater taxonomic resolution across the full tree of life^9^ and higher spatial and temporal precision than pollen—as eDNA mainly derives from the local community^10^. We used metagenomic analysis rather than the widely used metabarcoding approach because it enables the sequencing of DNA fragments from entire genomes without taxon-specific amplifications, therefore improving the specificity and sensitivity of taxonomic identification, as well as facilitating the authentication of endogenous ancient DNA from modern contaminants^9^. However, metagenomic analysis requires genome-scale reference data, which are limited for most regions of the world, including the Arctic. Thus, a key component of our study is the generation of a substantial corpus of plant reference sequences.

## Metagenomic dataset and database

**Fig. 1:**
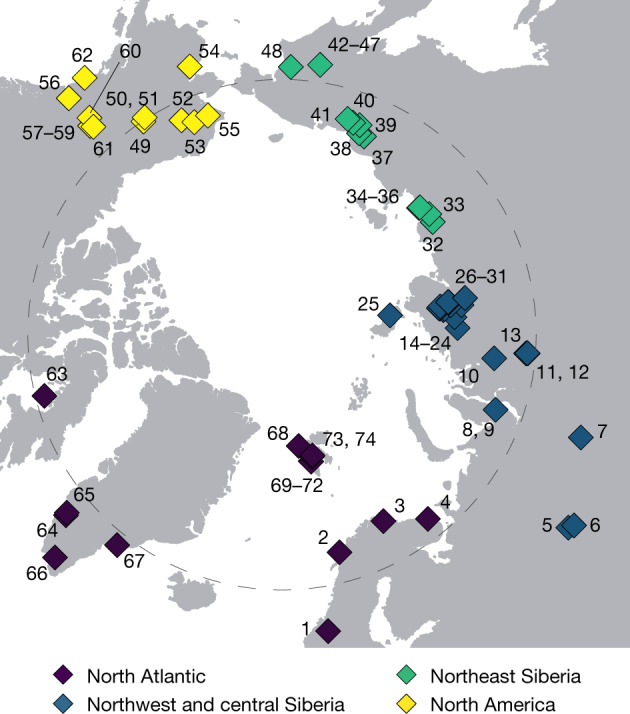
Site distribution (North Pole-centred view). Samples (*n* = 535) from a total of 74 circumpolar sites were grouped into four geographical regions (Supplementary Information 2). The grey dashed circle indicates the Arctic Circle (66.5° N). Site IDs are labelled on the map. The corresponding information is provided in Supplementary Data 1.

We generated the eDNA metagenomic dataset from 535 sediment samples obtained at 74 circumpolar sites (Fig. 1). Samples come from lake sediments and stratigraphic exposures (unconsolidated permafrost). For the purpose of understanding regional variability, we grouped sites into four regions: North Atlantic; northwest and central Siberia; northeast Siberia; and North America (Fig. 1). Sample ages span the past 50 kyr, albeit in varying numbers, from all regions with the notable exception of the North Atlantic, which was largely covered by ice sheets that often erased pre-LGM deposits^2,11^.

From the 535 samples, we generated 10.2 billion sequencing reads that passed the filtering criteria and were used for analysis (Methods). We created a comprehensive reference database for taxonomic identification by merging the NCBI-nt and NCBI-RefSeq databases, and supplemented the limited genomic-scale public reference data for Arctic species with 12 Arctic animals and an extensive sequencing effort of 1,541 modern Holarctic plant genome skims (PhyloNorway; Methods). These new sequences comprise 311.3 million whole-genome contigs and provide a broader and more reliable plant reference database than previously available. The merged reference database contains a total of 380.4 million entries and covers about 1.47 million organisms. We developed a *k*-mer-based method to evaluate the availability and coverage of our combined reference database for different taxa (Methods) and found that it covers a wide range of both Arctic and non-Arctic species (Supplementary Information 9.2.3). Accordingly, the addition of our new reference genomes did not cause bias towards Arctic taxa, providing confidence in our identifications. We used robust approaches to identify taxa from individual reads and collated the resulting taxonomic composition at the generic or familial level (Methods). We applied several methods to authenticate the plant and animal taxonomic profiles; the identifications were reliably classified despite the short DNA sequences that were preserved in these samples (Methods).

Moreover, 131 samples in this dataset were processed for metabarcoding, targeting the short DNA barcodes of plants^12^, enabling a comparison between the two approaches (Methods). The results showed that the metagenomic analysis captured greater floristic and faunal diversity and achieved better taxonomic resolution (Supplementary Information 11.2). We also found that only about 1.26% of the plant DNA reads are of ribosomal and chloroplast origin (Supplementary Information 9.2.5), suggesting that the metabarcoding approach—which relies on organelle DNA—makes use of only a small fraction of preserved DNA. However, we acknowledge that these comparisons are sample- and method-specific; more studies are needed before broader conclusions about the relative merits of the two approaches can be reached.

## Circum-Arctic vegetation dynamics

**Fig. 2:**
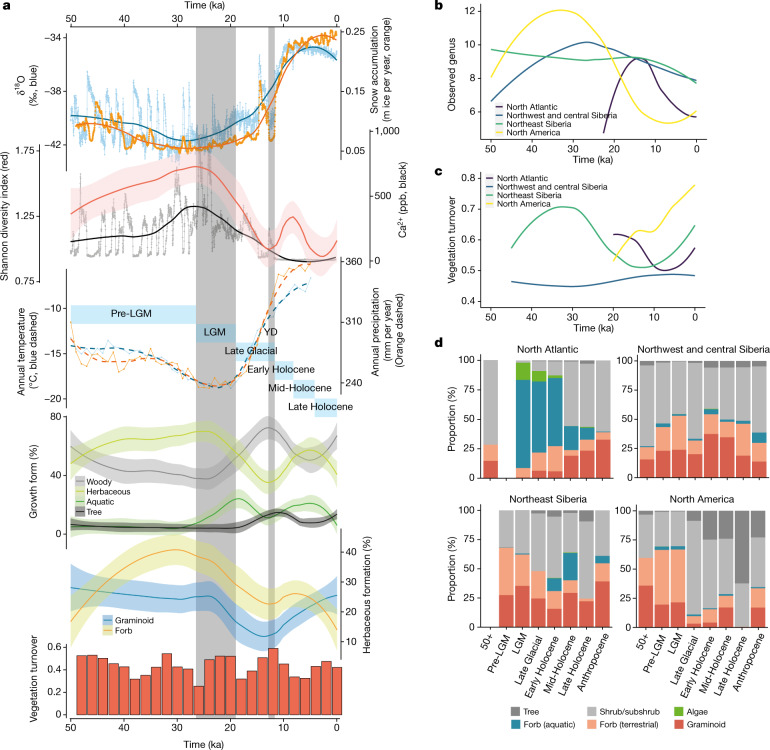
Climate and vegetation changes over the past 50 kyr. **a**, Pan-Arctic climate changes and vegetation variations. LGM (26.5−19 ka) and Younger Dryas (YD) (12.9−11.7 ka) are indicated by grey bars. The six time intervals are indicated by light blue bars (Supplementary Information 2). The error bands denote s.e. From top to bottom (see Methods for detailed calculations): the Greenlandic ice-core δ^18^O ratio and snow accumulation rate; the plant Shannon diversity and the Greenlandic ice-core calcium concentration; the average modelled annual temperature and precipitation for all eDNA sampling sites; the proportion of plant growth forms; the proportion of the herbaceous plant growth forms; and the vegetation turnover rates. **b**, The number of observed genera in different regions. **c**, Regional vegetation turnovers. **d**, Regional vegetation morphological compositions. The sample sizes for each region and time interval are provided in Supplementary Information 2. Calculations are supplied in the Methods.

**Extended Data Fig. 1:**
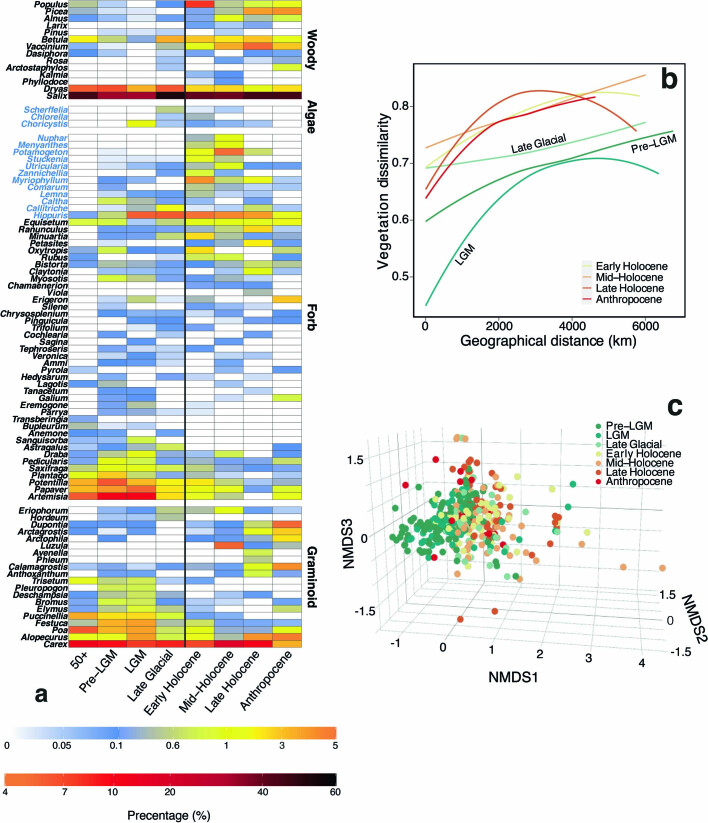
Circum-Arctic plant abundance variations and vegetation similarity clustering. **a**, Pan-Arctic plant abundance heatmap. **b**,  Spatial vegetation dissimilarities. Pairwise spatial beta-diversities (dissimilarities between every two plant communities) against the geographical distances between the two communities. **c**, Non-metric Multidimensional Scaling (NMDS, k=3) on vegetation communities.

We combined plant assemblages that were reconstructed from all of the samples to describe the temporal changes in floristic composition, diversity and community structure across the Arctic (Fig. 2a and Extended Data Fig. 1). Our results show substantial and repeated responses of Arctic vegetation to changing climates over the past 50 kyr.

The overall floristic diversity increased steadily from 50 ka and reached its highest levels at the onset of the LGM (about 26.5 ka), when the climate reached its coldest and driest point at many locations^2,11^ (Fig. 2a). Vegetation turnover was high before about 38 ka, and the identified shrubs, forbs and grasses suggest a shifting mosaic of steppe–tundra vegetation. Herbaceous plants were the dominant plant group until about 19 ka, with forbs more abundant than graminoids (Fig. 2a), but not as dominant as suggested by a previous metabarcoding study^12^. Trees and aquatic plants were limited in distribution to lower-latitude sites—consistent with overall dry and cold climate conditions during this period. The scarcity of cold-tolerant trees such as *Pinus* and *Picea*, and absence of *Larix*, reflect low precipitation and strong winds (Fig. 2a and Extended Data Fig. 1a).

The transition into the LGM featured declining temperature and precipitation (Fig. 2a). Across the Arctic, trees remained absent, and there was a sharp decrease in floristic diversity, mainly caused by the decline in herbaceous taxa. Overall, vegetation turnover was consistently high during this decline in diversity, suggesting that cold and dry extremes caused the loss of taxa from all plant communities, although the taxa that were dominant in the pre-LGM period remained (Extended Data Fig. 1a). LGM vegetation dissimilarity was the lowest of all time periods (Extended Data Fig. 1b, c), indicating considerable homogeneity across much of the unglaciated Arctic.

After the LGM, warming towards the Bølling–Allerød interstadial (approximately 14.6–12.9 ka)^13^ led to vegetation divergence among sites (Extended Data Fig. 1b, c). There was a substantial increase in the abundance of woody plants (such as *Salix* and *Betula*), whereas the herbaceous diversity continued to decline, causing the overall diversity to reach its lowest point at the beginning of the cold Younger Dryas stadial (approximately 12.9–11.7 ka)^14^ (Fig. 2a). The abundance of woody taxa and vegetation turnover rate reached the highest point during the Younger Dryas; the latter is consistent with the intensive climate changes that mark the transition from the Pleistocene to the Holocene.

Shortly after the Younger Dryas, summer insolation peaked and atmospheric CO_2_ reached Holocene levels^15^. Previously abundant plant taxa such as *Artemisia* and *Poa* rapidly declined or vanished locally. Other plant taxa, particularly boreal trees and prostrate shrubs (such as *Vaccinium*), appeared and later became abundant (Extended Data Fig. 1a), suggesting that there was a shift from open, cold-adapted tundra–steppe to a mosaic of herbaceous and woody plant communities. The floristic diversity of this more mesophilic vegetation increased during the Early Holocene as climate continued to warm and effective precipitation increased, but then declined during the middle Holocene (Fig. 2a).

Owing to dating uncertainties and limits on the temporal resolution of palaeoclimatic simulations, our results captured only broader changes in vegetation dynamics under climate change. During much of the past 50 kyr, overall plant diversity decreased when the proportion of trees and shrubs increased, as they outcompete herbaceous taxa through shading^16^. By contrast, when climate became more suitable for herbaceous taxa, diverse taxa expanded to share the landscape, and the overall diversity therefore increased.

## Regional vegetation dynamics

**Extended Data Fig. 2:**
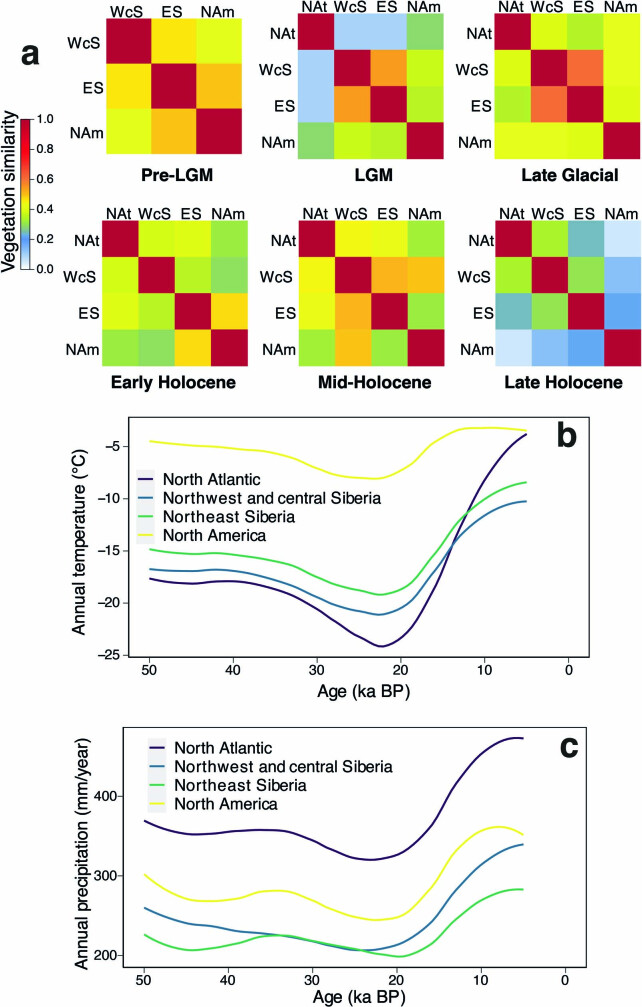
Regional vegetation differences and climate changes. **a**, Vegetation similarities between each two regions. All identified plant genera across sites in a region during a time interval were merged as a plant assemblage. Spatial beta-diversity between every two assemblages were calculated and illustrated. NAt, North Atlantic; WcS, Northwest and central Siberia; ES, Northeast Siberia; Nam, North America. **b** and **c**, Modelled annual temperature and precipitation in different regions. Means of the modelled annual temperature and precipitation values (Methods) at all eDNA sampling sites within a region at each 1,000-year time step were calculated. The changing trends are illustrated.

**Extended Data Fig. 3:**
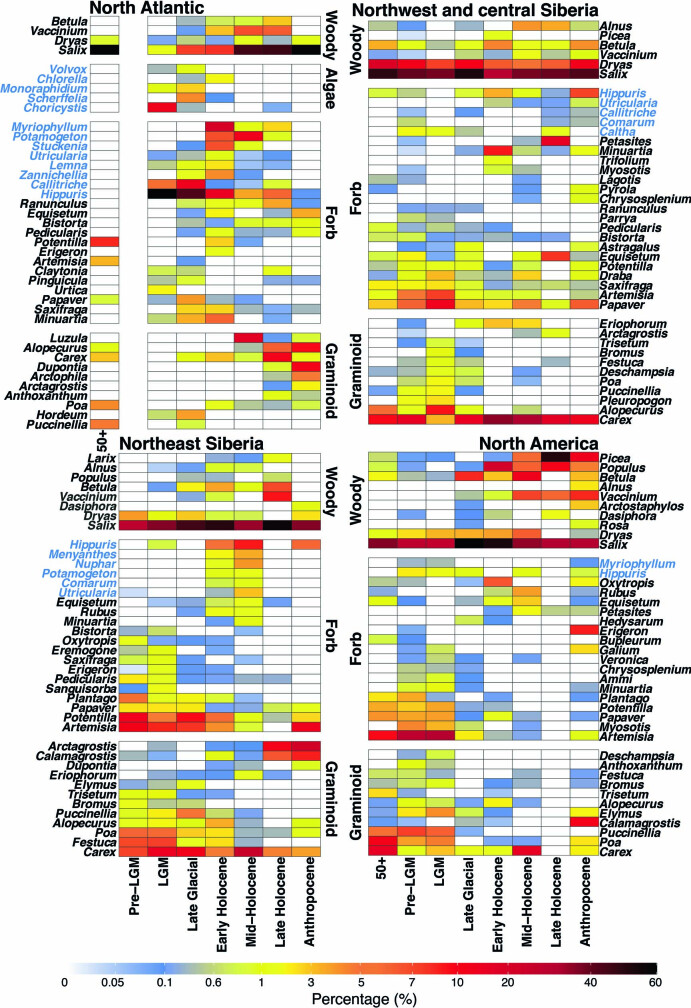
**Regional plant abundance heatmaps**. Heatmaps show the relative abundances of the 40 abundant plant genera in each region.

Underlying the generalized pattern of Holarctic vegetation changes are significant geographical differences. Early in postglacial times, the North Atlantic experienced the sharpest rises in taxonomic richness (Fig. 2b), along with the steepest temperature increase (Extended Data Fig. 2b). The increase in postglacial richness was probably driven by species dispersals coupled with habitat diversification^17^, that is, gynomorphically dynamic substrates that were exposed by glacial retreat and shaped by meltwater. The resultant vegetation initially had low diversity but was rich in aquatic taxa (Fig. 2b, d). The abundance of aquatic taxa relates in part to the prevalence of samples from lakes in the North Atlantic (Supplementary Information 10), but nonetheless highlights the ability of aquatic plants to disperse rapidly into newly deglaciated terrain containing abundant streams and lake basins^18^. As the postglacial climate continued to warm, the overall proportion of aquatic taxa declined as trees and shrubs (for example, *Betula*, *Salix* and *Vaccinium*) became abundant in this region (Fig. 2d and Extended Data Fig. 3).

Northeast Siberia and North America experienced less radical postglacial changes in vegetation type (Fig. 2c, d). During the Late Glacial, trees and shrubs became more widely distributed, and floristic diversity started to decline—a trend that was especially pronounced in North America (Fig. 2b, d). By about 12 ka, rising sea levels had flooded the Bering Strait, and the vegetation on each side started to diverge (Extended Data Fig. 2a). In northeast Siberia, greater effective moisture within the Holocene led to the expansion of aquatic plants (such as *Hippuris* and *Menyanthes*). The previously dominant steppe taxa (for example, *Poa* and *Artemisia*) declined, although sedges, of which many species are hygrophilous, continued to be abundant (Extended Data Fig. 3). The vegetation of this region became a mosaic of steppe and tundra elements. In North America, trees such as *Populus* and *Picea* became more widespread during the Early Holocene and previously widespread steppe species declined (Fig. 2d and Extended Data Fig. 3). A broad, southern swath of eastern Beringia became boreal forest.

In contrast to the changes observed in these regions, vegetation in northwest and central Siberia remained relatively unchanged through the Pleistocene–Holocene transition (Fig. 2c, d). However, some cold- and/or dry-adapted taxa (such as *Artemisia* and *Poa*) were replaced by forbs that were better adapted to warmer climates, and *Salix* was partially replaced by *Betula* and *Alnus* (Extended Data Fig. 3). The vegetation in this region persisted as a steppe–tundra mosaic through much of the Holocene, probably due to central Siberia’s extreme climatic continentality caused by the Siberian anticyclone^19^, which created largely ice-free conditions during the LGM and fostered dry hydrogeological conditions in postglacial times that mitigated the effects of rising global temperatures on vegetation^11^.

Overall, these results show that postglacial plant communities regionally diverged in response to warming temperatures, increasing moisture, retreating ice sheets and marine transgressions. Although regions that were once overridden by continental ice sheets experienced extreme vegetation changes, the vegetation in unglaciated interior regions remained rather stable. This maritime–continental contrast highlights the importance of moisture in driving ecosystem changes in the Arctic^7,20^. We next incorporate these insights into vegetation dynamics, together with other potential drivers, into a model to identify the factors influencing animal distributions.

## Animal distribution drivers

We developed a model using reconstructed animal distributions and floristic compositions, modelled palaeoclimate variables and inferred human occurrences (Methods) to examine the relative effects of abiotic and biotic factors on Arctic mammal distributions over the past 50 kyr.

**Fig. 3:**
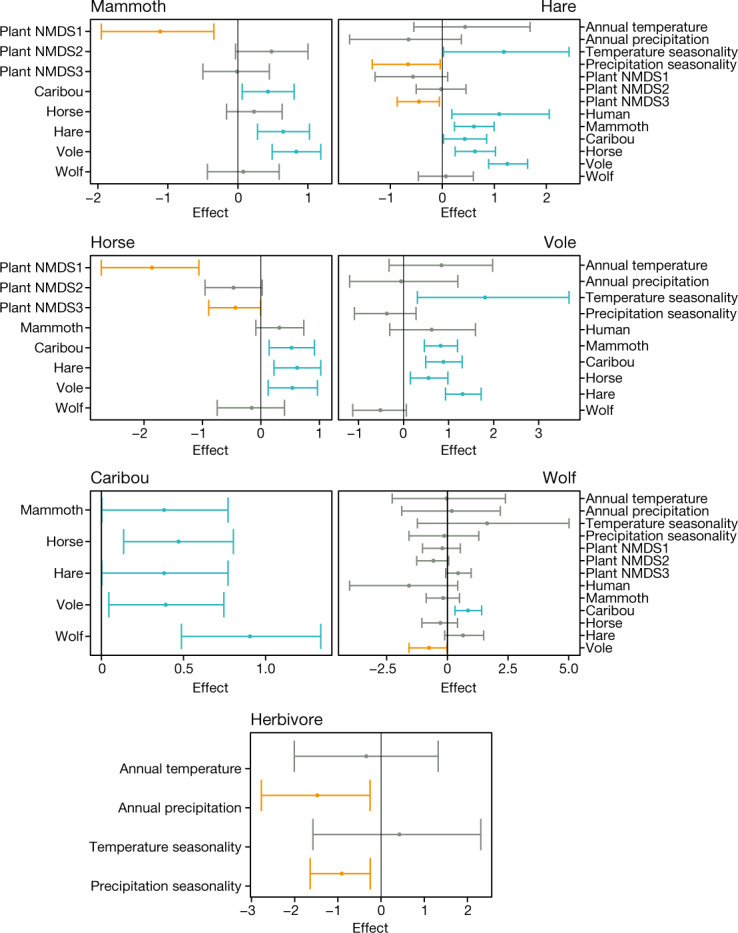
Spatiotemporal models to retrodict the explanatory factors for animal distribution. The values indicate posterior parameter estimates of covariate effects for the models explaining the presence–absence of each animal’s eDNA. Only covariates included in the model with lowest Watanabe–Aikaike information criterion are shown (Methods). The dots represent the posterior means, and the whiskers represent the posterior 2.5% and 97.5% quantiles. Covariate effects of which the 2.5% and 97.5% quantiles are both negative (red), and effects of which the 2.5% and 97.5% quantiles are both positive (blue) are indicated.

We found that certain herbivores tend to co-occur in time and space. For example, the eDNA presences of caribou, hare and vole are statistically strong co-indicators for the presence of horse and mammoth eDNA (Fig. 3). This suggests that co-existence was more common among Arctic herbivores than interspecies exclusion^21^. By contrast, the distribution of humans over time was almost entirely unrelated to the presence of most herbivores (apart from hares) (Fig. 3). Given that the model purposefully overestimated the presence of humans (Methods), their largely independent distributions from megafauna, their sparseness in the high Arctic before 4 ka (Supplementary Data 7) and the scarcity of kill sites in archaeological records, the notion of human overkill as the cause of Arctic megafaunal extinction is highly improbable^6,8^. Interestingly, the only predator–prey relationship of note in the model is the significant positive effect of caribou on the distribution of wolves (Fig. 3), probably reflecting that the wolf is well-adapted to hunt caribou.

**Extended Data Fig. 4:**
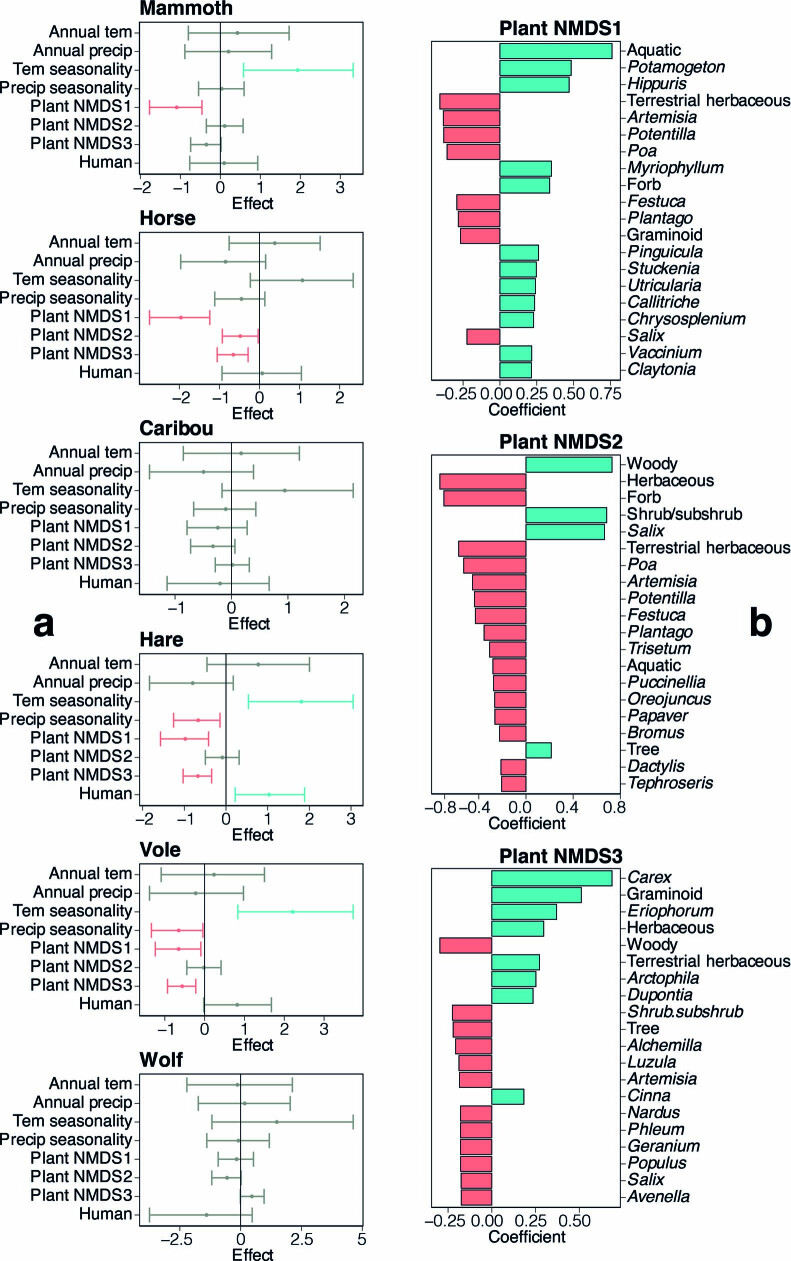
Environmental explanatory factors for animal distribution, and plant NMDS components. **a**, Posterior parameter estimates of covariate effects for the models explaining the presence/absence of each animal’s eDNA using climate, human presence and plant NMDS as explanatory variables. The dots represent the posterior means, and the whiskers represent the posterior 2.5% and 97.5% quantiles. The colour red denotes covariate effects whose 2.5% and 97.5% quantiles are both negative, while the colour blue denotes covariate effects 2.5% and 97.5% quantiles are both positive. **b**, The plant genera and morphological forms correlated to the 3 components of plant NMDS. Plant genera (morphological forms) are ranked by the p-value of t-test, and only the top 20 Pearson correlations are shown. The colour red denotes negative correlations while the colour blue denotes positive correlations.

To better gauge the explanatory power of environmental variables, we removed the effects of the presence of the eDNA of other animals (Extended Data Fig. 4a and Methods). The most consistent and widely prevalent patterns are the generally negative effects of plant NMDS1 and NMDS3—the first and third components of the non-metric multidimensional scaling (NMDS) of the vegetation compositions (Methods)—on the presence of animal eDNA. Plant NMDS1 reflects an aquatic-to-terrestrial plant gradient, and plant NMDS3 reflects a graminoids-to-woody plant gradient, particularly sedges within the graminoids, which include species that are prominent in present-day wetland communities (Extended Data Fig. 4b). These two negative covariates apply to the distribution of both small (vole and hare) and large (horse and mammoth) mammals, indicating that a wetter environment with a high proportion of hygrophilous plants (that is, moisture-loving plants) was a key factor restricting animal distributions. The distribution of mammoths tends to be positively affected by plant NMDS2, which mainly reflects the proportion of woody plants (particularly shrubs and subshrubs) as opposed to herbaceous plants, whereas the reverse is true for horses (Fig. 3). We also found that horses are more sensitive to vegetation composition compared with other herbivores (Supplementary Information 13.3). These findings support the hypothesis that horses were more restricted to a grassland environment and may also indicate a greater dietary flexibility in mammoths.

When each herbivore species is considered individually, the only climate variable that is consistently and positively associated with the presence of their eDNA is temperature seasonality (Fig. 3 and Extended Data Fig. 4a), consistent with expectations based on the continental climate associated with the Mammoth Steppe, a biome that is associated with extremely cold and dry conditions that supported abundant large mammal grazers^19^. The importance of climatic variables becomes more evident when herbivores are considered as a group. Precipitation—in greater amounts and seasonality—is a principal negative factor in the distribution of Arctic herbivores (Fig. 3), presumably because increased snow cover during winter limited the food access of grazers, and a wetter substrate is more difficult for them to exploit, in contrast to the firm and dry ground of the steppe–tundra^7,19^.

## Late-surviving megafauna

The timing of Arctic megafaunal extinction is a matter of debate, not least because last appearance dates (LADs) are repeatedly revised as younger fossils are reported^5,6^, and also because discovering the remains of the last surviving individuals of a species is extremely unlikely^22^. As a result, LADs systematically underestimate when a species disappeared, raising the possibility that populations persisted longer than is now evident^4,23^. The extinction timing can be better gauged with eDNA; an animal leaves behind only a single skeleton, which is much less likely to be preserved, recovered and dated, when compared with the amount of DNA it continuously spread into the environment while it was alive.

**Fig. 4:**
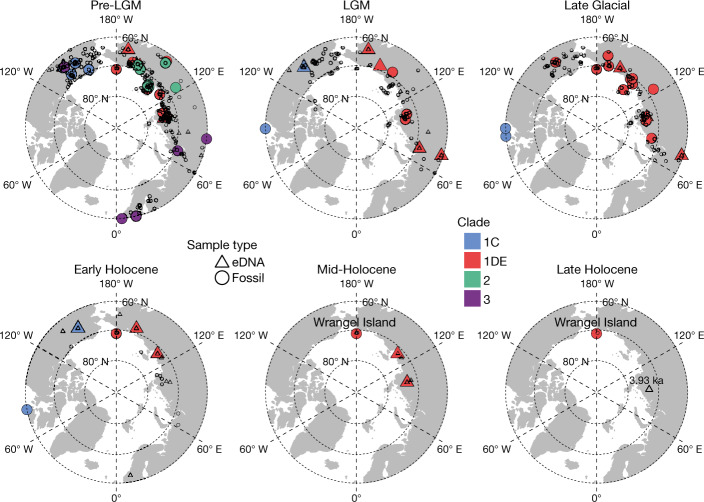
Mammoth distribution and mitochondrial haplotypes. A total of 78 mammoth mitochondrial genomes and 159 eDNA-identified mammoths (79 among them were assigned to mitochondrial haplotypes) are shown. Records of dated mammoth fossils^62^ are also plotted. All samples older than 26.5 ka were combined into the pre-LGM interval.

**Extended Data Fig. 5:**
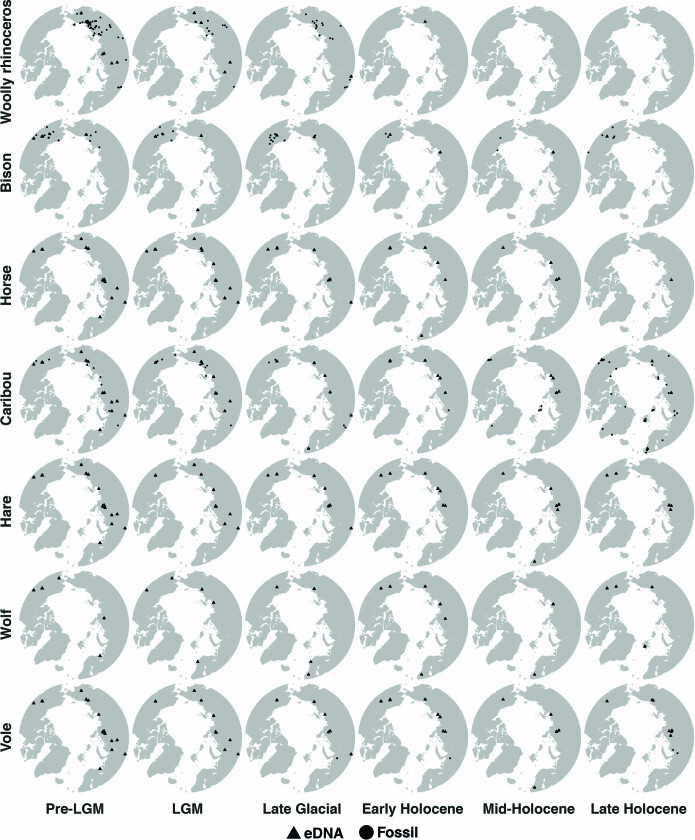
Distribution chronologies for woolly rhinoceros, bison, horse, caribou, hare, wolf, and vole. We combined our DNA results and the fossil records^62^ (available for woolly rhinoceros, bison, and caribou). Samples older than 26.5 ka were combined into Pre-LGM; samples younger than 4.2 ka were combined into the Late Holocene.

Our data indicate that mammoths survived into the Early Holocene in present-day continental northeast Siberia until 7.3 ± 0.2 ka (seven samples younger than 10 ka) and North America until 8.6 ± 0.3 ka. Notably, we recovered mammoth DNA from a series of samples from the Taimyr Peninsula that indicate the presence of mammoths in north central Siberia as late as 3.9 ± 0.2 ka (site LUR10) (Fig. 4 and Supplementary Information 3.3). The survival of mammoths into the Holocene in these regions is probably attributable to the persistence of the steppe–tundra vegetation of dry- and cold-adapted herbaceous plants that was present during the Pleistocene (Fig. 2d). This vegetation would have provided a suitable habitat for mammoths and possibly other dryland grazers such as horses (Extended Data Fig. 5), which are known to have survived in the region until at least 5 ka (ref. ^24^). Together, these eDNA results indicate that mammoths survived much longer than previously thought—which, on the basis of skeletal remains, was around 10.7 ka on continental Eurasia^25^ and around 13.8 ka in Alaska^8^. Given that humans occupied northern Eurasia sporadically from at least 40 ka and continuously after 16 ka (refs. ^26,27^), the late-surviving Taimyr mammoths potentially encountered and co-existed with humans over at least a 20-kyr interval, therefore giving no support to the human overkill (blitzkrieg) model that postulates the mammoth extinction occurred within centuries after the first human contact^6^.

We also detected woolly rhinoceros DNA as late as 9.8 ± 0.2 ka in northeast Kolyma, horse DNA in Alaska and the Yukon as late as 7.9 ± 0.2 ka, and bison as late as 6.4 ± 0.6 ka in high-latitude localities of northeast Siberia (Extended Data Fig. 5). All of these instances represent substantially later LADs than fossil-based dates (that is, for woolly rhinoceros in Eurasia, about 14 ka (ref. ^28^); and for horses and steppe bison in Alaska, 12.5 ka (refs. ^5,8^)). Collectively, these findings highlight the value of eDNA in improving megafauna extinction chronologies.

## Population diversity of megafauna

Megafaunal eDNA from across the Arctic also enables us to resolve population-level patterns, which is crucial for uncovering species-specific demographic and evolutionary responses to past climatic and environmental changes. We applied a method for phylogenetically assigning the identified eDNA to mitochondrial haplogroups of mammoth and horse, the two most abundant species detected in our dataset (Methods).

**Extended Data Fig. 6:**
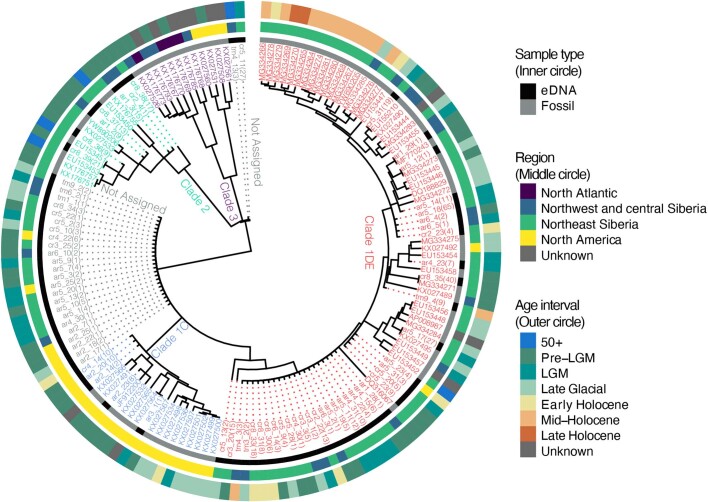
Mammoth mitochondrial phylogenetic tree. For placed eDNA samples the number of supporting single-nucleotide polymorphisms is given in braces (Methods). IDs for the Wrangel Island population are underlined.

A mammoth phylogeny composed of four previously described major mitochondrial clades (clade 1, including 1C and 1DE, and clades 2 and 3)^29^ was reconstructed from 78 mammoth mitochondrial genomes. The recovered mammoth eDNA was then assigned to a best-fit node on the tree based on single-nucleotide polymorphism (SNP) support/conflict, enabling clade assignment for 79 eDNA samples (Extended Data Fig. 6).

The mammoth haplogroups that we identified are consistent with those that were previously identified from fossil remains and have comparable biogeographical and biostratigraphic distributions (Fig. 4). Overall, clade 3 was present mainly in Europe and northwest Siberia, whereas clade 2 occurred mostly in central and northeast Siberia. Clade 1 was widely scattered across North America and the Asian Arctic, with 1DE occurring throughout Siberia and 1C in North America. Temporally, clades 2 and 3 were the older lineages, and disappeared between 40 ka and 30 ka. Only clade 1 survived past the LGM, with the last 1C individual dating to 10.35 ka. Like the late-surviving mammoths on Wrangel Island^30^, the late-surviving mammoths on mainland Siberia were also members of 1DE, the only clade detected to date that postdates the Early Holocene (that is, after 8.2 ka). However, despite belonging to the same clade, none of the mainland late-surviving populations is placed in the Wrangel Island haplogroup (Extended Data Fig. 6). Furthermore, we note that two mammoth eDNA samples (cr5_11 and tm4_13) attach to the existing tree at the shared root of clades 2 and 3 (Extended Data Fig. 6), with cr5_11 containing many sequence variants not found in previously sequenced samples (Supplementary Information 14.1.2), suggesting that they represent a separate and previously unrecorded mitochondrial lineage. The distinctive mitochondrial genome haplogroups, together with the shrinking and increasingly isolated occurrences of mammoths (Fig. 4), hint that Siberian mainland mammoths experienced a similar fate to those on Wrangel and St Paul Islands. However, whether the precise causes of their disappearance were the same^4,30^, and whether the mainland mammoth also accumulated detrimental mutations consistent with genetic decline^31^, will require further data to resolve.

The reliability of our method was further corroborated on the horse phylogeny (Supplementary Information 14.2). Successful assignment of ancient eDNA data to mitochondrial haplogroups, even when the DNA is highly degraded, highlights the potential for applying eDNA analysis to uncover population histories in regions in which fossils are rare or absent.

## Concluding remarks

Controversy has persisted for decades over the nature of the Mammoth Steppe, a distinctive, now-vanished biome dominated by large mammal grazers^1,19,32^. Some studies, emphasizing the abundance of grazers (and the absence of large browsers), suggest that broad swaths of the unglaciated Late Pleistocene Arctic were covered by an extensive steppe dominated by low-sward herbaceous plants that were well-suited for megafaunal grazers^19,32^. Others, on the basis of pollen and plant macrofossil records, suggest that Arctic vegetation during this period was regionally diverse and included both tundra and steppe taxa^3,33^. Our results suggest the nature of the Mammoth Steppe lies in between these two seemingly conflicting interpretations. Consistent with the view of the Mammoth Steppe as a biome of intercontinental extent, our data show that various regions of the Arctic supported a more homogenous vegetation cover before and during the LGM (Extended Data Fig. 1b, c). We also found evidence of an elevated and episodic turnover of plant taxa during the Late Pleistocene compared with during the Holocene (Fig. 2a), consistent with inferences about changeable vegetation types during the glacial age based on the network of palaeobotanical (and fossil insect) sites presently available^3,12^. Jointly, our results suggest that the Mammoth Steppe was a regionally complex cryo-arid steppe, composed of forbs, graminoids and willow shrubs.

Our findings relating to the late survival of megafauna have important implications for the debate over the causes of Late Quaternary extinctions. Megafaunal survival into the Holocene indicates that, at least in certain parts of the Arctic and Subarctic, humans coexisted with these species for tens of thousands of years, which implies that human hunting was not an important factor in their extinction^6,25^. Instead, our results suggest that their extinction came when the last pockets of the steppe–tundra vegetation finally disappeared, when the Arctic-wide paludification was brought on by warmer and wetter climates^7,20^.

What we have mined from this substantial dataset does not exploit its full potential. For example, we detected DNA of Camelidae (most probably the Arctic camel^34^) and *Panthera* (possibly the steppe lion). However, due to a lack of reference genomes for these species, we could not confirm these identifications. This constraint also applies to other species because our reference database—large as it is—is far from complete, despite our extensive sequencing efforts. With more species sequenced and new bioinformatics methods developed, this dataset can be reanalysed to explore more questions of Arctic biotic history.

Our study demonstrates how metagenomic analysis of eDNA extracted from ancient sediments can provide diverse insights, from detailed records of past flora and fauna to reconstructions of population histories and biotic interactions, to a greatly expanded spatiotemporal network of palaeoecological records. These advances are important in the context of continuous efforts to elucidate the past 50 kyr of Arctic biotic dynamics, especially given that the coevolution of plant and animal species, and their responses to the past climatic changes across this vast region, have previously been challenging to address at this resolution and at this scale using classical palaeobotanical and palaeontological data.

## Methods

### Sampling, chronology and eDNA taphonomy

Sampling and subsampling methods are described in Supplementary Information 1. Sample ages were determined through conventional or accelerator mass spectrometer radiocarbon (^14^C) as well as optically stimulated luminescence. In total, 631 radiocarbon ages and 81 optically stimulated luminescence dates were used. For sedimentary sections with multiple contiguous dates without stratigraphic inversions, age–depth models were built to calculate sedimentation rates and estimate the ages of undated samples within these sections. All radiocarbon ages are in calibrated years before present, calibrated using IntCal20 (ref. ^35^). Chronological information is provided in Supplementary Information 2 and Supplementary Data 1 and 2.

To determine whether DNA was in situ, control samples were obtained from modern surfaces, from water in adjacent rivers and lakes, and from stratigraphic layers bracketing the samples. Consistent with previous eDNA studies in the Arctic^12,23,36^, we found no evidence of DNA leaching or redeposition in either terrestrial or lake sediment samples (Supplementary Information 5).

### DNA extraction and sequencing

We tested the performance of different operations included in the widely used ancient eDNA extraction protocols^36–38^ and a variety of purification methods on different sediment sample types. On the basis of these tests, we developed two new eDNA-extraction protocols that were optimized for isolating and purifying eDNA from our sediment samples (Supplementary Information 6.1 and 6.2). The InhibitEx-based protocol was then applied for extracting DNA from all samples. DNA extracts were thereafter converted into sequencing libraries according to the standard protocol^39^, and sequenced using Illumina platforms after quality controls (Supplementary Information 6.3). All DNA extractions and pre-index analyses were performed in the dedicated ancient DNA laboratories at the Centre for GeoGenetics, University of Copenhagen, according to established ancient DNA protocols^40^.

### PhyloNorway plant genome database construction

The PhyloNorway plant genome database was constructed by sequencing 1,541 Arctic and boreal plant specimens collected from herbaria. DNA was extracted from the selected specimens using a modified Macherey–Nagel Nucleospin 96 Plant II protocol. Two different library preparation protocols were applied depending on DNA yields. All of the libraries were then sequenced. Nuclear ribosomal DNA and chloroplast genome from each plant were assembled to evaluate the data quality. Whole-genome contigs for each plant were assembled and annotated as the final reference database. A list of plant species, herbarium information, DNA extraction, sequencing and database statistics are supplied in Supplementary Data 3. Data for three standard barcodes skimmed from this database were also used in ref. ^41^. Details are provided in Supplementary Information 7.

### Taxonomic identification, authentication and quantification

We performed taxonomic classification by mapping reads against a comprehensive genomic database that was annotated with taxonomic information according to the principle of the Holi pipeline^36^. Details of the composition of the reference database are provided in Supplementary Information 9.2.1.

All reads were first quality-controlled, and each read was then offered an equal chance to be aligned against all entries in the database after duplicate removal (Supplementary Information 9.1 and 9.2). No limitation to specific taxonomic group, geography or environment was applied for the alignment. The lowest common ancestor of all of the hits with 100% similarity was assigned to each read that had been aligned to multiple taxa. The taxonomic coverage of different database compositions and their effects on taxa identification were evaluated using a *k*-mer-based method (Supplementary Information 9.2.2). We found that using a proper reference database is important for eDNA metagenomics-based taxa identification, particularly for ancient datasets in which the DNA is highly fragmented. Even reference genome availability across taxa can improve the sensitivity and specificity of the identification by increasing the identified reads and correcting the misidentifications (Supplementary Information 9.2.5). Taxa that were detected in the laboratory controls were combined into a list, and all of the listed taxa were subtracted from samples (Supplementary Information 9.3). The resulting plant and animal taxonomic profiles were thereafter parsed for additional authentication using a series of conservative thresholds (Supplementary Information 9.4 and 9.6), on the basis of an Arctic flora and faunal checklist (Supplementary Information 8). Plant taxa that passed these filters all have Arctic or boreal distributions (Supplementary Information 9.4). All eDNA reads aligned to an animal were further confirmed as exclusive alignments, by requiring perfect alignment to that animal, and no alignment to any other organisms when allowing for 1 or 2 mismatches (Supplementary Information 9.6.3). The two extinct animals— mammoth and woolly rhinoceros— were also confirmed by the DNA-damage patterns (Supplementary Information 9.6.2).

Relative abundances for plants were estimated on the basis of the number of the assigned reads, by excluding the effects of DNA degradation in different samples, and eliminating the effects of the sequencing depth among different samples and the efficiency of the taxa-identification pipeline among different taxa (Supplementary Information 9.5).

### Vegetation diversity and dissimilarity

The Shannon diversity index was calculated according to the method in ref. ^42^. Plant morphological forms were assigned at the genus level on the basis of the plant trait database of eFloras (http://www.efloras.org). Beta-diversity (dissimilarity) between every two plant assemblages was calculated according to the method in ref. ^43^. For the pan-Arctic vegetation turnover (Fig. 2a), plant genera identified in all samples in each 2,000-year interval were combined as an assemblage; beta-diversity between each two consecutive intervals was calculated. Regional vegetation turnover (Fig. 2c) was calculated at 5,000-year intervals. NMDS (*k* = 3; Extended Data Fig. 1c) was performed using the R package vegan^44^, allowing 100,000 iterations of random starting to find the best convergent solution. Correlations between the abundance of each plant genus (or proportion of each morphological form) and the values of each of the three NMDS components (Extended Data Fig. 4b) were assessed using the Pearson product–moment correlation and *t*-test (*P* < 0.05).

### Comparison of eDNA shotgun metagenomics and metabarcoding

We applied two modules for comparing the metabarcoding and shotgun metagenomics in taxa identifications. (1) We conducted the two sequencing techniques in parallel on 14 DNA extracts to directly compare the retrieved taxonomic profiles. (2) We compared the floristic profiles reconstructed by this study and a previous metabarcoding study^12^ on 131 overlapping samples of the two datasets. The results show that metagenomics performed better on our samples in both captured floristic and faunal diversity. Details are provided in Supplementary Information 11.

### Palaeoclimate panels and human distribution niche modelling

For the ice-core data from Greenland (Fig. 2a), we rescaled the available δ^18^O ratios (20-year slices) retrieved from NGRIP1 (ref. ^45^), NGRIP2 (ref. ^46^) and GISP2 (ref. ^47^) to the range of the corresponding ratio of GRIP^48^, for which there are valid values for all age slices, using the rescale function in the R package scales. The mean of the available ratios for each time slice from the four datasets was calculated and used. Calcium concentrations were calculated from refs. ^49,50^ using the same method as for δ^18^O. Snow-accumulation rates were based on GISP2 (ref. ^51^).

We also modelled monthly palaeoclimate anomalies at 1,000-year time steps using an emulator^52^ and downscaled them onto a modern baseline climatology (CHELSA)^53^ at a spatial resolution of 1°. From these data, we calculated four environmental variables—annual mean temperature, temperature seasonality, annual precipitation and precipitation seasonality—that were used to represent the climate for each of our eDNA sites. Details are provided in Supplementary Information 12.1.

We developed distribution models to map environmentally suitable conditions for Palaeolithic human occurrence in steps of 1,000 years from 5 ka to 31 ka and steps of 2,000 years from 32 ka to 47 ka. First, geo-references for human remains in the Arctic were collected and dates from ^14^C calibrations inferred from two databases CARD2.0 (ref. ^54^) and the Palaeolithic of Europe^55^. These data were filtered for quality, resulting in a final set of 6,497 occurrences. From 32 ka to 47 ka, we calculated 2,000-year averages of the four environmental variables. We then generated five-algorithm ensemble models at each time step to characterize the climatic niche of Palaeolithic humans. We validated all of the models by assessing the area under the receiver operating characteristic curve (AUC) and true skill statistic; we also used model AUCs to generate weighted ensemble models at each time step. Finally, we projected the ensemble models into geographic space to map climatic suitability for humans, expressed as the potential presence or absence at each time step at each of the eDNA sites. Details are provided in Supplementary Information 12.2.

### Spatiotemporal models for animal eDNA

We combined our animal eDNA data with the modelled climate variables, projected human occurrence and the NMDS ordinations of vegetation to examine the relative impacts of climate, human activity and vegetation on the geographical distributions of a selected group of Arctic mammals. We developed a method to spatiotemporally model animal eDNA presence, using these three sets of variables, while accounting for auto-correlation in time and space. The method uses a hierarchical Bayesian model that includes a spatiotemporal Gaussian random field, and was implemented in R-INLA^56,57^. We used the Watanabe–Akaike information criterion to assess the model fit using different sets of covariates. Detailed methods are provided in Supplementary Information 13.

### Mammoth and horse mitochondrial haplotyping

We placed eDNA mitochondrial reads for mammoth and horse into their respective mitochondrial reference phylogenies using recently developed software^58^. We used existing variation to assign informative markers onto branches of a mitochondrial phylogeny, then determined the number of supporting and conflicting single-nucleotide polymorphisms for each eDNA sample on each branch of the tree to place the sample onto the most likely branch. Detailed methods are provided in Supplementary Information 14.

### Statistics and data visualization

Changing trends are illustrated against time (Fig. 2a–c and Extended Data Fig. 2b, c) or distance (Extended Data Fig. 1b) via the Loess Smooth (span = 4) function in the R package ggplot2 (ref. ^59^), with original data points or confidence intervals (s.e.) shown when other curves are not obstructed. The heat maps showing the mean of a genus’ proportions across all samples within an age interval were generated using the R package ComplexHeatmap^60^. The mammoth phylogenetic tree was illustrated using ggtree, which is included in the R package ggplot2. The base map source for Fig. 1 and Extended Data Fig. 5 was Arctic SDI and, for Fig. 4, was the R package maptools.

### Reporting summary

Further information on research design is available in the Nature Research Reporting Summary linked to this paper.

## Online content

Any methods, additional references, Nature Research reporting summaries, source data, extended data, supplementary information, acknowledgements, peer review information; details of author contributions and competing interests; and statements of data and code availability are available at 10.1038/s41586-021-04016-x.

## Supplementary information

Supplementary Information See SI guide for full description of contents.

Reporting Summary

Supplementary Data 1 Site description An Excel table covering the site metadata for all 74 eDNA sampling sites.

Supplementary Data 2 Sample metadata & age-dpeth models.xlsx An Excel table covering the sample metadata for all 535 eDNA samples, and the age–depth models.

Supplementary Data 3 PhyloNorway metadata An Excel table covering the metadata for all 1,541 PhyloNorway herbarium specimens.

Supplementary Data 4 Arctic flora and fauna checklist An Excel table covering the assembled Arctic plant and animal checklist.

Supplementary Data 5 Plant abundance An Excel table covering the reconstructed plant genera abundance.

Supplementary Data 6 Animal distribution & DNA damage An Excel table covering the animal existence/absence matrix and the animal ancient DNA damage rate.

Supplementary Data 7 Human Presence/Absence A PDF file showing the modelled environmentally suitable conditions for Palaeolithic human occurrence.

Supplementary Data 8 Human Presence/Absence for eDNA sites An Excel table covering the modelled human existence/absence matrix for eDNA sampling sites.

Supplementary Data 9 Horse mitochondrial genomes An Excel table covering the metadata for the horse mitochondrial genomes used for constructing the horse phylogeny.

## Data Availability

Adapter-removed plant or animal eDNA data were deposited at EMBL-ENA under project accession ERP127790. The raw data of PhyloNorway plant genome database are available at EMBL-ENA under project accession PRJEB43865. Assembled plant genome contigs of the PhyloNorway database are available at DataverseNO^61^. NCBI databases are available at the NCBI ftp server (https://ftp.ncbi.nlm.nih.gov). The Canadian Archaeological Radiocarbon Database (CARD2.0) is available online (https://www.canadianarchaeology.ca). The Radiocarbon Palaeolithic Europe Database is available online (https://ees.kuleuven.be/geography/projects/14c-palaeolithic). All other data are provided in the Supplementary Information and Supplementary Data 1–9.

## Code availability

Scripts are archived at GitHub (https://github.com/wyc661217/Arctic_eDNA_2021).
