# Author Correction: Late Quaternary dynamics of Arctic biota from ancient environmental genomics

**DOI:** 10.1038/s41586-022-05359-9

**Published:** 2022-09-26

**Authors:** Yucheng Wang, Mikkel Winther Pedersen, Inger Greve Alsos, Bianca De Sanctis, Fernando Racimo, Ana Prohaska, Eric Coissac, Hannah Lois Owens, Marie Kristine Føreid Merkel, Antonio Fernandez-Guerra, Alexandra Rouillard, Youri Lammers, Adriana Alberti, France Denoeud, Daniel Money, Anthony H. Ruter, Hugh McColl, Nicolaj Krog Larsen, Anna A. Cherezova, Mary E. Edwards, Grigory B. Fedorov, James Haile, Ludovic Orlando, Lasse Vinner, Thorfinn Sand Korneliussen, David W. Beilman, Anders A. Bjørk, Jialu Cao, Christoph Dockter, Julie Esdale, Galina Gusarova, Kristian K. Kjeldsen, Jan Mangerud, Jeffrey T. Rasic, Birgitte Skadhauge, John Inge Svendsen, Alexei Tikhonov, Patrick Wincker, Yingchun Xing, Yubin Zhang, Duane G. Froese, Carsten Rahbek, David Nogues Bravo, Philip B. Holden, Neil R. Edwards, Richard Durbin, David J. Meltzer, Kurt H. Kjær, Per Möller, Eske Willerslev

**Affiliations:** 1 Department of Zoology, University of Cambridge, Cambridge, UK; 2 Lundbeck Foundation GeoGenetics Centre, GLOBE Institute, University of Copenhagen, Copenhagen, Denmark; 3 The Arctic University Museum of Norway, UiT— The Arctic University of Norway, Tromsø, Norway; 4 Department of Genetics, University of Cambridge, Cambridge, UK; 5 Université Grenoble Alpes, Université Savoie Mont Blanc, CNRS, LECA, Grenoble, France; 6 Center for Macroecology, Evolution and Climate, GLOBE Institute, University of Copenhagen, Copenhagen, Denmark; 7 Department of Geosciences, UiT—The Arctic University of Norway, Tromsø, Norway; 8 Université Paris-Saclay, CEA, CNRS, Institute for Integrative Biology of the Cell (I2BC), Gif-sur-Yvette, France; 9 Génomique Métabolique, Genoscope, Institut François Jacob, CEA, CNRS, Université Evry, Université Paris-Saclay, Evry, France; 10 Institute of Earth Sciences, St Petersburg State University, St Petersburg, Russia; 11 Arctic and Antarctic Research Institute, St Petersburg, Russia; 12 School of Geography and Environmental Science, University of Southampton, Southampton, UK; 13 Alaska Quaternary Center, University of Alaska Fairbanks, Fairbanks, AK USA; 14 Centre d’Anthropobiologie et de Génomique de Toulouse, Université Paul Sabatier, Faculté de Médecine Purpan, Toulouse, France; 15 National Research University, Higher School of Economics, Moscow, Russia; 16 Department of Geography and Environment, University of Hawaii, Honolulu, HI USA; 17 Department of Geosciences and Natural Resource Management, University of Copenhagen, Copenhagen, Denmark; 18 Carlsberg Research Laboratory, Copenhagen, Denmark; 19 Center for Environmental Management of Military Lands, Colorado State University, Fort Collins, CO USA; 20 Faculty of Biology, St Petersburg State University, St Petersburg, Russia; 21 Department of Glaciology and Climate, Geological Survey of Denmark and Greenland, Copenhagen, Denmark; 22 Department of Earth Science, University of Bergen, Bergen, Norway; 23 Bjerknes Centre for Climate Research, Bergen, Norway; 24 US National Park Service, Gates of the Arctic National Park and Preserve, Fairbanks, AK USA; 25 Zoological Institute, , Russian Academy of Sciences, St Petersburg, Russia; 26 Resource and Environmental Research Center, Chinese Academy of Fishery Sciences, Beijing, China; 27 College of Plant Science, Jilin University, Changchun, China; 28 Department of Earth and Atmospheric Sciences, University of Alberta, Edmonton, Alberta Canada; 29 Center for Global Mountain Biodiversity, GLOBE Institute, University of Copenhagen, Copenhagen, Denmark; 30 School of Environment, Earth and Ecosystem Sciences, The Open University, Milton Keynes, UK; 31 Department of Anthropology, Southern Methodist University, Dallas, TX USA; 32 Department of Geology, Quaternary Sciences, Lund University, Lund, Sweden; 33 Wellcome Trust Sanger Institute, Wellcome Genome Campus, Cambridge, UK; 34 MARUM, University of Bremen, Bremen, Germany

**Keywords:** Ecological networks, Palaeoecology, Metagenomics, Next-generation sequencing, Climate-change ecology

Correction to: *Nature* 10.1038/s41586-021-04016-x Published online 20 October 2021

In the version of this article initially published, there were mismatches between sample ID and the data in the second tag “damage” for Supplementary Data 6: Animal distribution & DNA damage. This occurred through reformatting in manuscript preparation and does not affect the rest of the paper. The corrected table has been uploaded to the HTML version of the article.

