# Supplementary material for: Late Quaternary dynamics of Arctic biota from ancient environmental genomics
Source: Nature. 2021 Oct 20;600(7887):86–92. doi: 10.1038/s41586-021-04016-x (PMC8636272; doi:10.1038/s41586-021-04016-x)
Supplement: Supplementary file 1 — See SI guide for full description of contents. [file 41586_2021_4016_MOESM1_ESM.pdf]

nature portfolio

https://doi.org/10.1038/s41586-021-04016-x

Supplementary information

Late Quaternary dynamics of Arctic biota from ancient environmental genomics

In the format provided by the authors and unedited

Late Quaternary Dynamics of Arctic Biota from Ancient Environmental Genomics

Yucheng Wang[1,2,35], Mikkel Winther Pedersen[2,35], Inger Greve Alsos[3,35], Bianca De Sanctis[1,4], Fernando Racimo[2], Ana Prohaska[1], Eric Coissac[3,5], Hannah Lois Owens[6], Marie Kristine Føreid Merkel[3], Antonio Fernandez-Guerra[2], Alexandra Rouillard[2,7], Youri Lammers[3], Adriana Alberti[8,9], France Denoeud[9], Daniel Money[1], Anthony H. Ruter[2], Hugh McColl[2], Nicolaj Krog Larsen[2], Anna A. Cherezova[10,11], Mary E. Edwards[12,13], Grigory B. Fedorov[10,11], James Haile[2], Ludovic Orlando[14], Lasse Vinner[2], Thorfinn Sand Korneliussen[2,15], David W. Beilman[16], Anders A. Bjørk[17], Jialu Cao[2], Christoph Dockter[18], Julie Esdale[19], Galina Gusarova[3,20], Kristian K. Kjeldsen[21], Jan Mangerud[22,23], Jeffrey T. Rasic[24], Birgitte Skadhauge[18], John Inge Svendsen[22,23], Alexei Tikhonov[25], Patrick Wincker[9], Yingchun Xing[26], Yubin Zhang[27], Duane G. Froese[28], Carsten Rahbek[6,29], David Bravo Nogues[6], Philip B. Holden[30], Neil R. Edwards[30], Richard Durbin[4], David J. Meltzer[2,31], Kurt H. Kjær[2], Per Möller[32], Eske Willerslev[1,2,33,34]*

[1]Department of Zoology, University of Cambridge, Downing St., Cambridge, CB2 3EJ, UK. [2]Lundbeck Foundation GeoGenetics Centre, GLOBE Institute, University of Copenhagen, Øster Voldgade 5-7, 1350 Copenhagen, Denmark. [3]The Arctic University Museum of Norway, UiT - The Arctic University of Norway, NO-9037 Tromsø, Norway. [4]Department of Genetics, University of Cambridge, Downing St., Cambridge, CB2 3EJ, UK. [5]Univ. Grenoble-Alpes, Univ. Savoie Mont Blanc, CNRS, LECA, 38000, Grenoble, France. [6]Center for Macroecology, Evolution and Climate, GLOBE Institute, University of Copenhagen, Universitetsparken 15, Bld. 3, 2100 Copenhagen Ø, Denmark. [7]Department of Geosciences, UiT - The Arctic University of Norway, NO-9037 Tromsø, Norway. [8]Université Paris-Saclay, CEA, CNRS, Institute for Integrative Biology of the Cell (I2BC), 91198, Gif-sur-Yvette, France. [9]Génomique Métabolique, Genoscope, Institut François Jacob, CEA, CNRS, Univ Evry, Université Paris-Saclay, 91057 Evry, France. [10]Institute of Earth Sciences, St. Petersburg State University, 7-9 Universitetskaya Emb., St. Petersburg 199034, Russia. [11]Arctic and Antarctic Research Institute, 38 Beringa St., St. Petersburg 199397, Russia. [12]School of Geography and Environmental Science, University of Southampton, University Road, Southampton SO17 1BJ, UK. [13]Alaska Quaternary Center, University of Alaska Fairbanks, Fairbanks, AK 99775, USA. [14]Centre d'Anthropobiologie et de Génomique de Toulouse, Université Paul Sabatier, Faculté de Médecine Purpan, 37 allée Jules Guesde, Bâtiment A, 31000 Toulouse, France. [15]National Research University, Higher School of Economics, 20 Myasnitskaya Ulitsa, 101000, Moscow, Russia. [16]Department of Geography and Environment, University of Hawaii, Honolulu, HI 96822, USA. [17]Department of Geosciences and Natural Resource Management, University of Copenhagen, Øster Voldgade 10, 1350 Copenhagen, Denmark. [18]Carlsberg Research Laboratory, J.C. Jacobsens Gade 4, DK-1799 Copenhagen V, Denmark. [19]Center for Environmental Management of Military Lands, Colorado State University, Fort Collins, Colorado 80523,

USA. [20]Faculty of Biology, St Petersburg State University, 7-9 Universitetskaya Emb., St. Petersburg 199034, Russia. [21]Department of Glaciology and Climate, Geological Survey of Denmark and Greenland, Øster Voldgade 10, 1350 Copenhagen K, Denmark. [22]Department of Earth Science, University of Bergen, Norway. [23]Bjerknes Centre for Climate Research, Bergen, Norway. [24]U.S. National Park Service, Gates of the Arctic National Park and Preserve, Fairbanks, Alaska 99709, U.S.A. [25]Zoological Institute, Russian academy of sciences, St. Petersburg 199034, Russia. [26]Resource and Environmental Research Center, Chinese Academy of Fishery Sciences, Beijing 100141, China. [27]College of Plant Science, Jilin University, Changchun, Jilin 130062, China. [28]Department of Earth and Atmospheric Sciences, University of Alberta, Edmonton, Canada. [29]Center for Global Mountain Biodiversity, GLOBE Institute, University of Copenhagen, Universitetsparken 15, 2100 Copenhagen, Denmark. [30]School of Environment, Earth and Ecosystem Sciences, The Open University, Milton Keynes, UK. [31]Department of Anthropology, Southern Methodist University, Dallas, Texas, 75275, U.S.A. [32]Department of Geology, Quaternary Sciences, Lund University, Sölvegatan 12, SE-22362, Lund, Sweden. [33]Wellcome Trust Sanger Institute, Wellcome Genome Campus, Cambridge CB10 1SA, UK. [34]MARUM, University of Bremen, Bremen, Germany.

[35]These authors contributed equally: Yucheng Wang, Mikkel Winther Pedersen, Inger Greve Alsos

*To whom correspondence should be addressed: E-mail: ew482@cam.ac.uk.

1. Site and sample 1
1.1 Site selection 1
1.2 Sampling and subsampling 1
2. Geographical regions and time period definitions 2
3. Chronology 3
3.1 Dating method 4
3.2 Age-depth modelling 4
3.3 Chronology for the site with late surviving mammoth 23
4. Contamination control 27
5. Sedimentary DNA taphonomy 27
6. DNA laboratory methodology 29
6.1 Extraction test 29
6.1.1 Test content 30
6.1.2 Test procedures 31
6.2 Extraction protocols 36
6.2.1 InhibitEx based protocol 36
6.2.2 Zymo-Spin based protocol 36
6.3 Library preparation and sequencing 37
7. PhyloNorway plant genome database 38
7.1 Sampling and DNA extraction 39
7.2 Library preparation and sequencing 40
7.3 nrDNA and cpGenome assembly and annotation 41
7.4 Quality control 45
7.5 Reference database construction 46
7.5.1 Reads filtering 46
7.5.2 Contigs assembling 46
7.5.3 Database formation and annotation 46
8. Arctic flora and fauna checklist 47
9. Bioinformatics for taxa identification 48
9.1 Reads quality controls 48
9.2 Reference database creation and taxonomic classification 49
9.2.1 Database composition 49
9.2.2 Database coverage for arctic taxa 51
9.2.3 Database coverage for the Asian steppe taxa 55
9.2.4 Sequencing reads alignment 58
9.2.5 Specificity and sensitivity for taxonomic classifications 59
9.3 Laboratory controls 65
9.4 Plant identification authentication 67
9.5 Plant abundance estimation 69
9.5.1 Effect of DNA damage 69
9.5.2 Elimination of the sequencing depth effect 71
9.5.3 Effect of taxa identification efficiency 73
9.6 Megafauna authentication 81
9.6.1 Mammalian taxa grouping 81
9.6.2 DNA damage 82
9.6.3 Thresholds for megafauna authentication 86
10. Effect of sample type on identified plant 88

10.1 Sample type distribution and effect on vegetation diversity 88
10.2 Effect on algae and aquatic proportion 89
11. Metabarcoding *versus* shotgun metagenomics 91
11.1 Direct comparison 92
11.1.1 Data generation 92
11.1.2 Data processing 92
11.1.3 Comparison 93
11.2 Comparison with the 2014 metabarcoding dataset 95
12. Climate and human distribution modelling 97
12.1 Modelled paleoclimate 97
12.2 Human occurrence modeling 99
13. Spatiotemporal modelling of animal eDNA data 101
13.1 The model 101
13.2 Predicting animal eDNA sampling probabilities 103
13.3 Modeling covariates 105
13.4 Partitions in time and space 109
14. Mammoth and horse mitochondrial haplotype 123
14.1 Mammoth 123
14.1.1 Reference tree 125
14.1.2 Phylogenetic assignment 128
14.1.3 Result 132
14.2 Horse 134
14.2.1 Reference tree and eDNA sample assignment 134
14.2.2 Results 135
15. Code and script archive 138
References 138

1. Site and sample

1.1 Site selection

The past two decades have seen several independent expeditions sampling sedimentary deposits across a wide variety of locations in the circumpolar, undertaken by members of the Centre for GeoGenetics and associated collaborators. The resulting circumpolar sediment sample repository contains more than 5,000 samples, from which we assembled samples for this study following 5 criteria. (i) The site (or section) exhibits no sign of reworking or leaching, preferable to be lacustrine or permafrost sedimentary profile (see Section 5 and the corresponding references). (ii) The sampling method should comply with ancient DNA standards (see Section 1.2). (iii) Samples should come from well-characterized sedimentary profiles with explicit documentation on the stratigraphy. (iv) The age of the samples can be precisely determined (see Section 3). (v) The age frame of the sedimentary section is largely younger than 50 kilo annum Before Present (ka BP).

Filtering under these criteria resulted in a total of 535 samples from 74 sites across the Arctic. Complete sample metadata including site names, locations, coordinates, chronology, sedimentological context, and for previously published samples the corresponding references[1-30] is supplied in Supplementary Data 1 and Supplementary Data 2.

1.2 Sampling and subsampling

Owing to site-specific sedimentological differences and different sampling strategies from various research groups involved, samples were collected using different methods, but all with deliberate precautions to avoid DNA-sensitive contamination. In general, sampling was either performed by directly withdrawing samples from the profile *in situ*, or by taking out larger bulk samples that later were subsampled under clean-controlled conditions in the dedicated laboratory at the Centre for GeoGenetics, University of Copenhagen. More detailed description of the two methods follows below.

In the first method, the profile was first smeared with highly-concentrated solution of a synthetic recognizable DNA tracer as described in ref. 31, for tracking potential contaminations on the profile surface that were introduced during excavation. Wearing

facemask, sleeves, gloves and hairnet, the tracer-contaminated surface was then removed at each sampling point using disposable sterile scalpels, thereafter a sterile centrifuge tube was pushed into the profile and then withdrawn, with the sediment sample contained within. The tube was then sealed and packed in a separate zip-lock bag. All samples were kept frozen until subsequent analysis, first in iceboxes during fieldwork, and then in -20 °C freezers once available.

The second method of obtaining bulk samples was undertaken by drilling or cutting out a big sediment chunk directly from the sedimentary profile. Bulk samples were thereafter kept frozen until further subsampling. Prior to the subsampling of bulk samples, the surface was smeared with the DNA tracer same as the first method, and thereafter subsampled by first removing the outer layer and then drilling the inner intact sediment inside a walk-in freezer, as described in ref. 32, with wearing full-body suit, gloves, mask, hairnet and sleeves.

A few permafrost sites did not have exposed profiles, and were therefor drilled from the surface down, resulting in vertical cores as described in ref. 14. Subsampling for permafrost cores followed the method for bulk sample subsampling described above.

Lake cores were obtained using percussion corers deployed from the top of frozen lakes or from the side of a floating raft. Subsampling was performed as described in ref. 27 and 33.

2. Geographical regions and time interval definitions

The selected 535 samples were grouped into 4 geographical regions and 8 age intervals (Supplementary Table 2 and Supplementary Data 2) to better illustrate the variations corresponding to the main climatic periods in different regions.

Importantly, a few geographical outliers were merged with a neighboring region. Sites from Arctic Scandinavia, Svalbard, Greenland, and Baffin Island are combined as North Atlantic. Sites from Yamal Peninsula, Gydan Peninsula and Taymyr Peninsula, together with the Zaozer'e site and Garchi Site in Komi and the Yuribei River site in Yugra are merged as the Northwest and central Siberia (Northwest and central Siberia). Although the 2 Komi sites are located west of the Ural Mountains and therefore technically belongs to Europe, they are both climatically and chronologically more similar to the sites in

Northwest and central Siberia. Sites distributed along the Siberian coastline east of Taymyr Peninsula, as well as one site from the Kolyma lowlands are designated to the geographical region Northeast (NE) Siberia. Sites from Alaska and Yukon were combined into the geographical region North America.

For the age grouping, samples older than 50 kyr BP were grouped as “50+”, while modern surface samples or samples with radiocarbon resulting very young ages (>1950 AD) are assigned to the Anthropocene[34]. Based on climatic changes since 50 ka BP, the later part of the Late Pleistocene is split in three intervals sliced by the Last Glacial maximum (LGM)[35], as Pre-LGM, LGM, and Late Glacial. Holocene is split into three intervals according to the GTS2020[36], as Early Holocene (the Greenlandian Stage, also known as Lower Holocene), Mid-Holocene (Middle Holocene, the Northgrippian Stage), and Late Holocene (the Meghalayan Stage, also known as Upper Holocene). Details for the range of age intervals, and the number of samples falling within each geographical region and age interval are summarized in the Supplementary Table 2. Additional information for each sample is supplied in Supplementary Data 2.

Supplementary Table 2 | Temporal and spatial distribution of eDNA samples

Interval	Age frame	North Atlantic	Northwest and central Siberia	Northeast Siberia	North America	Sum
50+	older than 50 ka BP	3	7	1	15	26
Pre-LGM	50-26.5 ka BP	0	51	55	26	132
LGM	26.5-19 ka BP	5	20	28	10	63
Late Glacial	19-11.7 ka BP	8	27	9	20	64
Early Holocene	11.7-8.2 ka BP	16	9	18	28	71
Mid-Holocene	8.2-4.2 ka BP	26	22	23	9	80
Late Holocene	4.2-0 ka BP	44	7	6	4	61
Anthropocene	younger than 1950 AD	13	13	2	10	38
Sum	NA	115	156	142	122	535

3. Chronology

Ages were mainly determined by two dating methods: conventional or Accelerator Mass Spectrometer (AMS) radiocarbon dating (^{14}C, on terrestrial organic material), and Optically Stimulated Luminescence (OSL, on silty-sandy sediment), and occasionally

supported by some other measures (see Supplementary Data 1 for details). When consecutively dated samples in a profile and the sample depths or heights are available, age-depth model for the stratigraphic section was built using an algorithm applying an autoregressive gamma process[37]. The established age-depth model was then used to provide ages for samples within the section that had not been directly dated. A full list of the chronological information can be found in Supplementary Data 1 and Supplementary Data 2.

3.1 Dating method

We combined both previously published and new dates, resulting in a total of 631 ^{14}C ages for the chronology determinations. The ^{14}C dating were performed at a number of laboratories, including: the AMS Radiocarbon Dating Laboratory, Lund University, Sweden (LuS); Poznan Dating Laboratory, Poland (Poz); Oxford Radiocarbon Accelerator Unit, England (OxA); Queen's University Belfast, Northern Ireland (UBA); University of Cologne AMS, Germany (COL); University of California, Irvine, USA (UCIAMS); NSF AMS Lab at the University of Arizona, USA (AA); Geological Survey, Canada (GSC); Beta Analytic, USA (Beta); Trondheim AMS, Norway (TRa); Trondheim AMS, Norway (TUa). All finite ages are given as radiocarbon years (^{14}C BP) with 1σ age deviation, as well as calibrated radiocarbon years calibrated using the R package Bchron[38] based on the IntCal20[39].

A total of 81 OSL dates were used for chronology determination. OSL dating was preformed following the method in ref. 10. Details for each sample are supplied in Supplementary Data 2.

3.2 Age-depth modelling

For sites with only sampling height available, the heights were first converted into relative depths. We used R package rbacon[40] for age-depth modeling. Function “Bacon” was applied for building the model, with accepting the suggested accumulation rate (suggest = T). All Bayesian age-depth models are shown in Supplementary Figure 3.2.1 - 3.2.19, and data for all models is supplied in Supplementary Data 2.

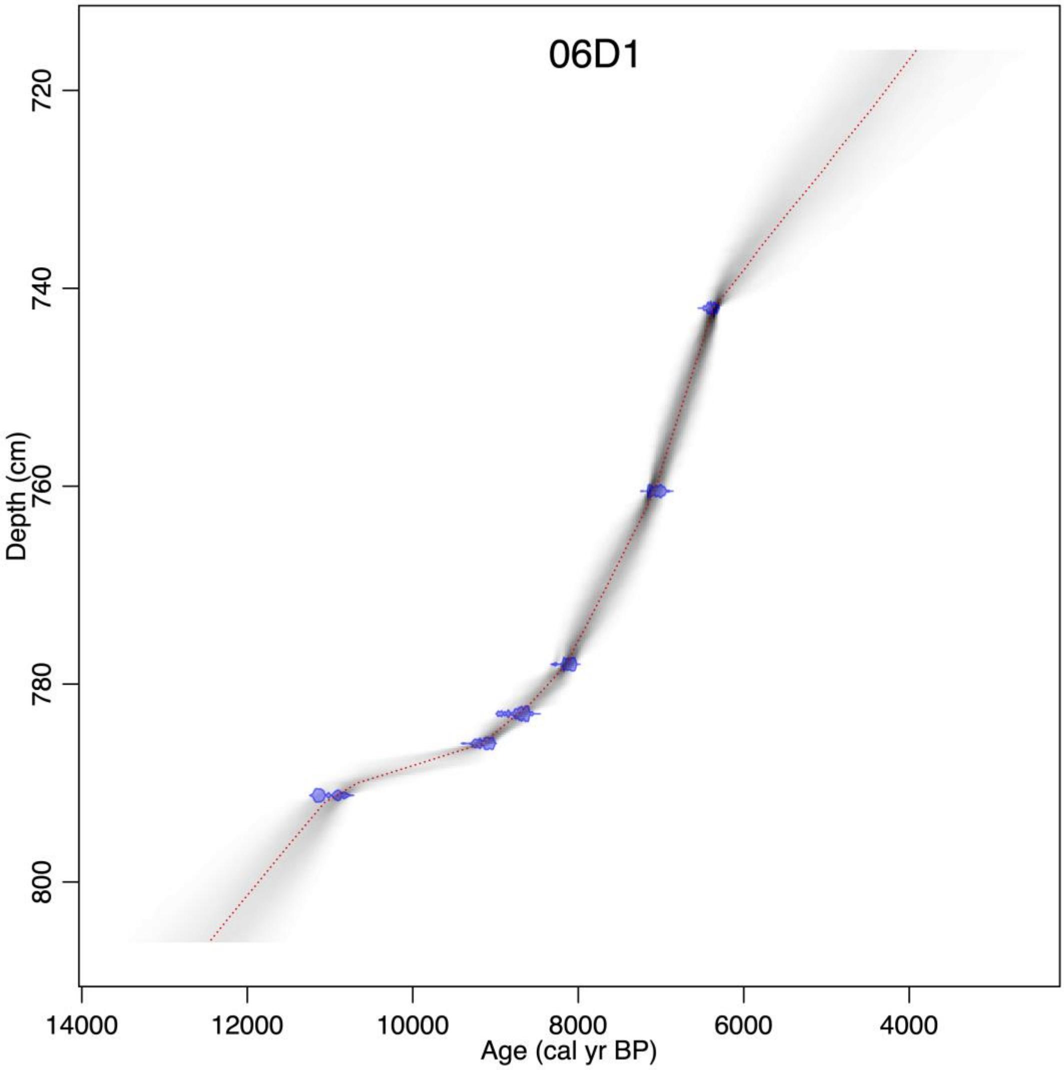

Supplementary Figure 3.2.1 | Age–depth model for site 06D1 (Site ID 1, Dovre 2006). Blue bars denote calibrated radiocarbon ages included for the model; red bars denote calibrated radiocarbon ages excluded; red line denotes the best-fitted model; grey shadow denotes the 95% confidence interval.

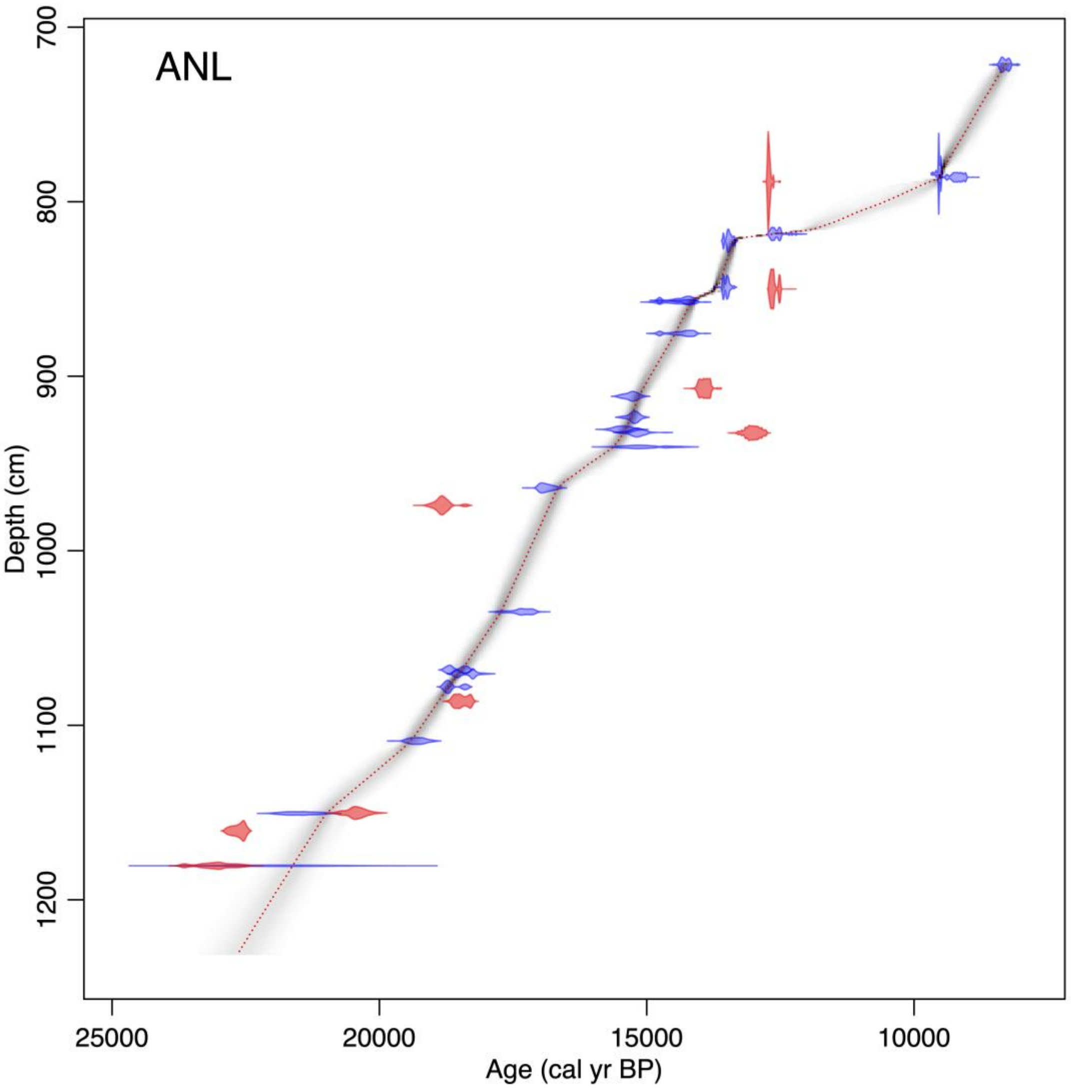

Supplementary Figure 3.2.2 | Age–depth model for site ANL (Site ID 2, Endlettvatnet, Andøya). Blue bars denote calibrated radiocarbon ages included for the model; red bars denote calibrated radiocarbon ages excluded; red line denotes the best-fitted model; grey shadow denotes the 95% confidence interval.

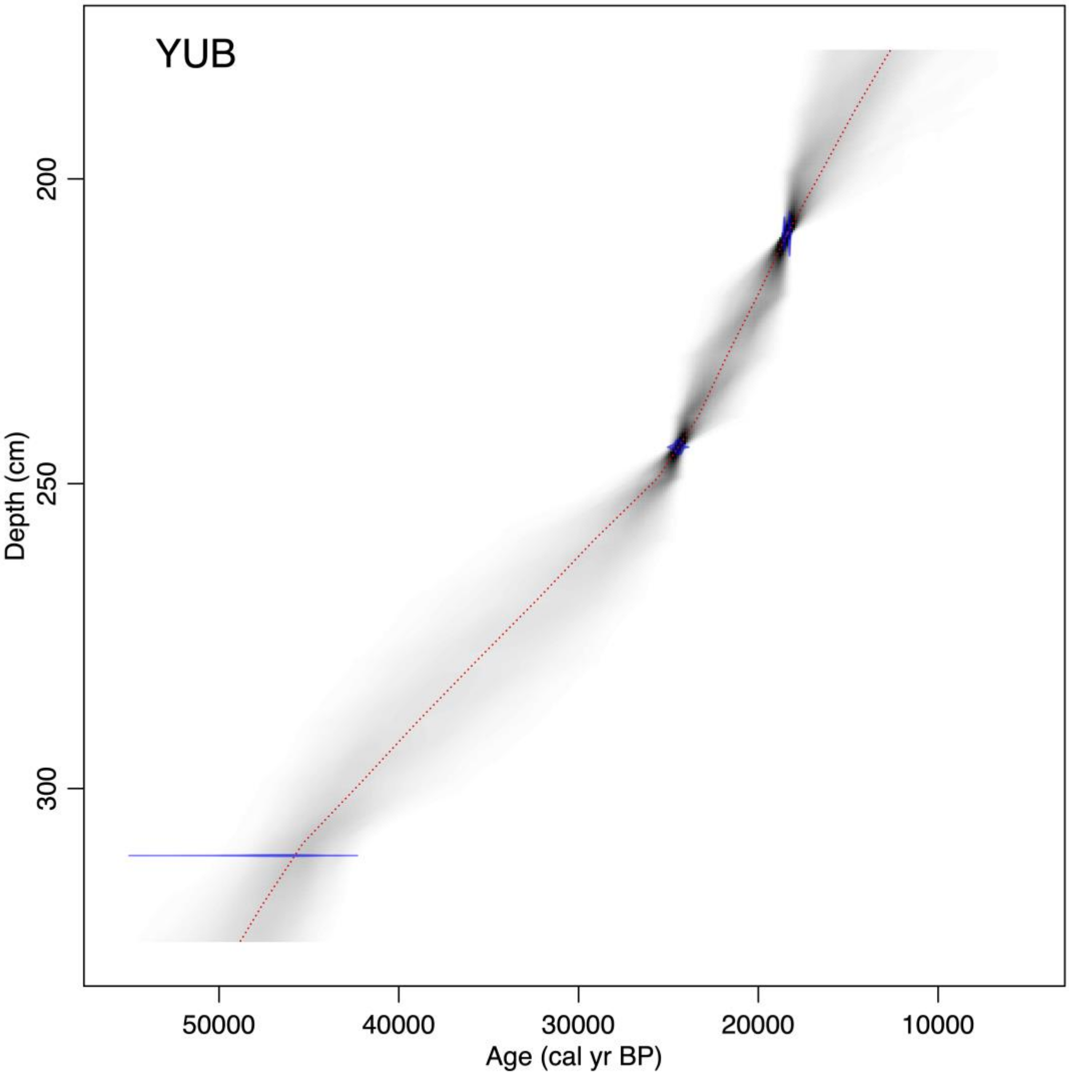

Supplementary Figure 3.2.3 | Age–depth model for site YUB (Site ID 7, Yuribei River). Blue bars denote calibrated radiocarbon ages included for the model; red bars denote calibrated radiocarbon ages excluded; red line denotes the best-fitted model; grey shadow denotes the 95% confidence interval.

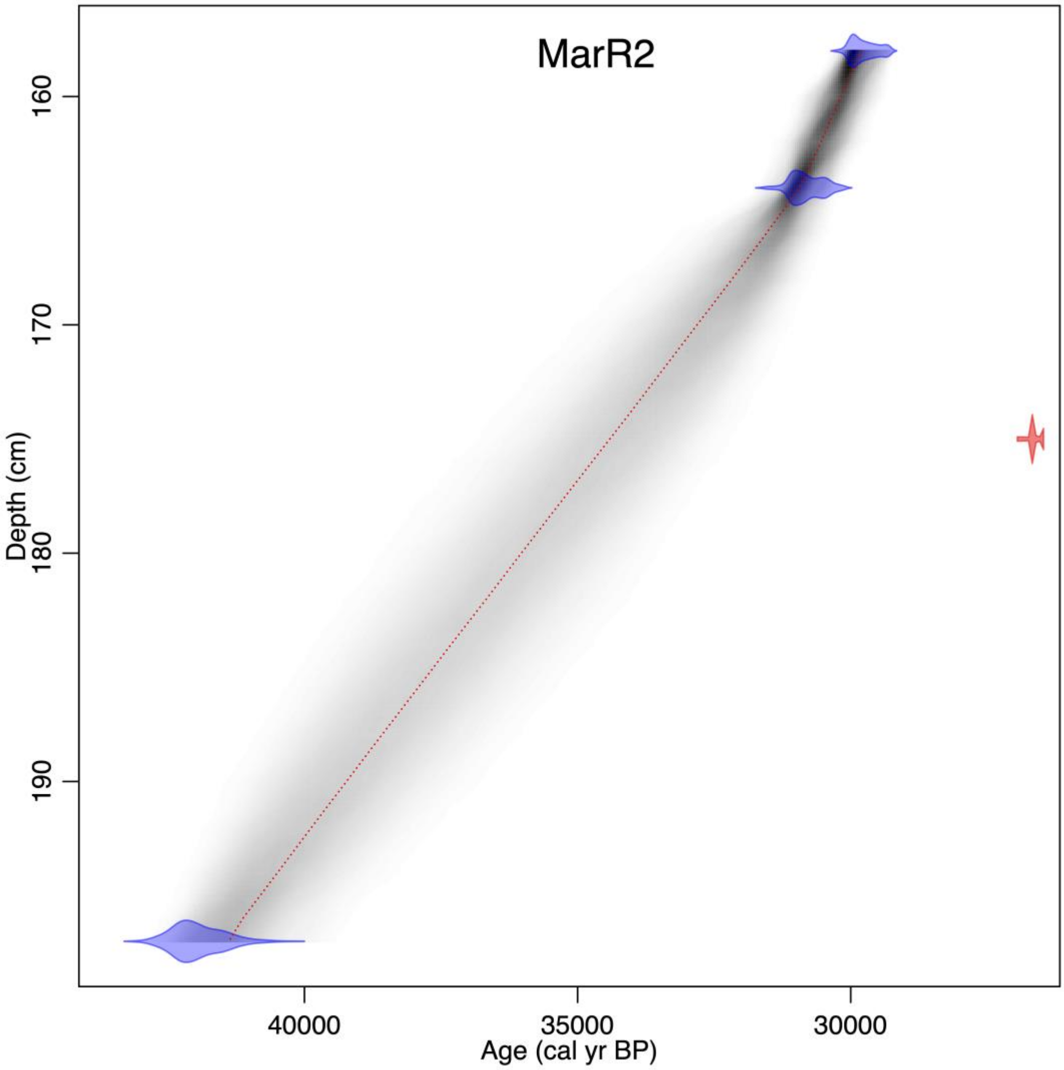

Supplementary Figure 3.2.4 | Age–depth model for site MarR2 (Site ID 9, Marita River 2). Blue bars denote calibrated radiocarbon ages included for the model; red bars denote calibrated radiocarbon ages excluded; red line denotes the best-fitted model; grey shadow denotes the 95% confidence interval.

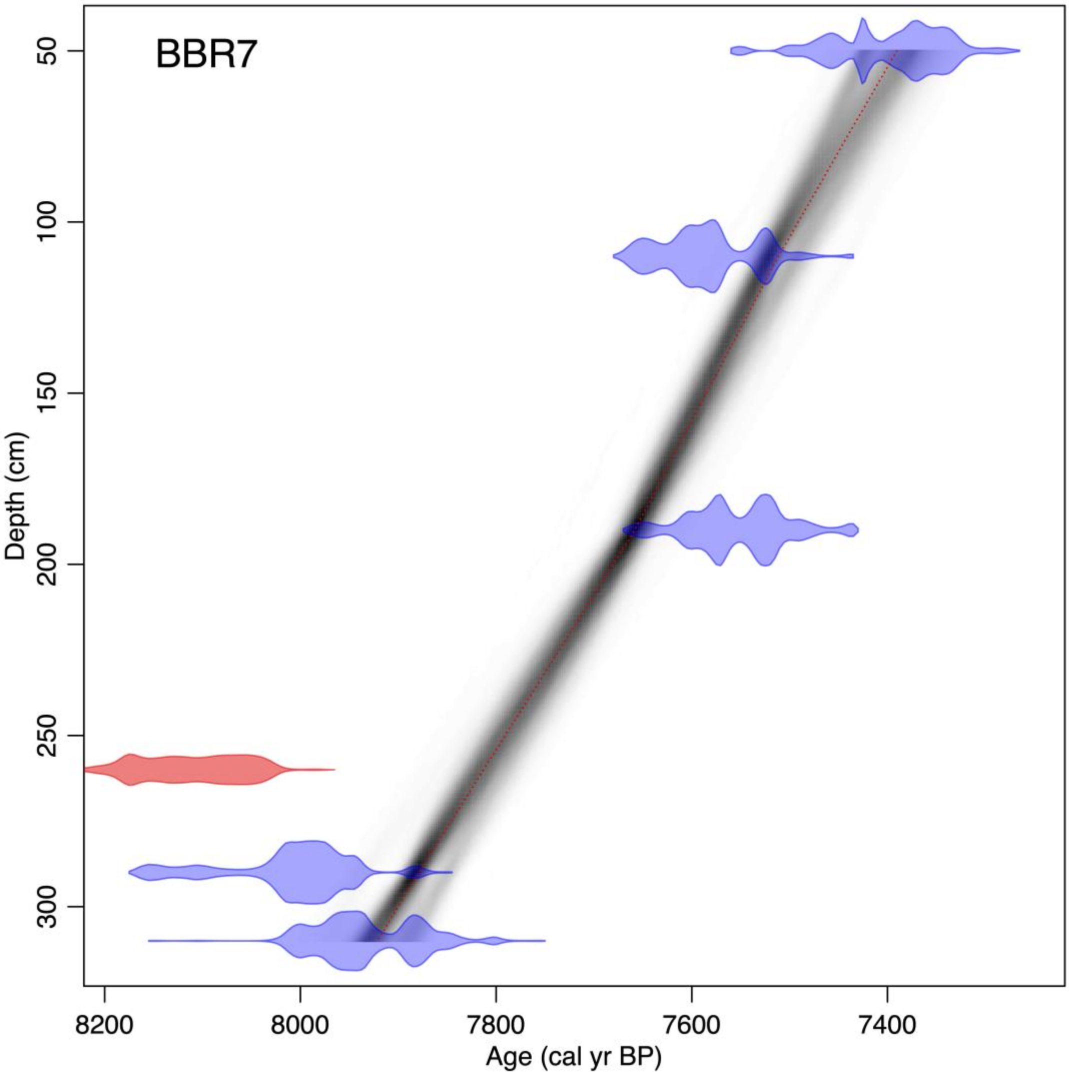

Supplementary Figure 3.2.5 | Age‒depth model for site BBR7 (Site ID 23, Bol'shaya Balaknya River, site 7). Blue bars denote calibrated radiocarbon ages included for the model; red bars denote calibrated radiocarbon ages excluded; red line denotes the best-fitted model; grey shadow denotes the 95% confidence interval.

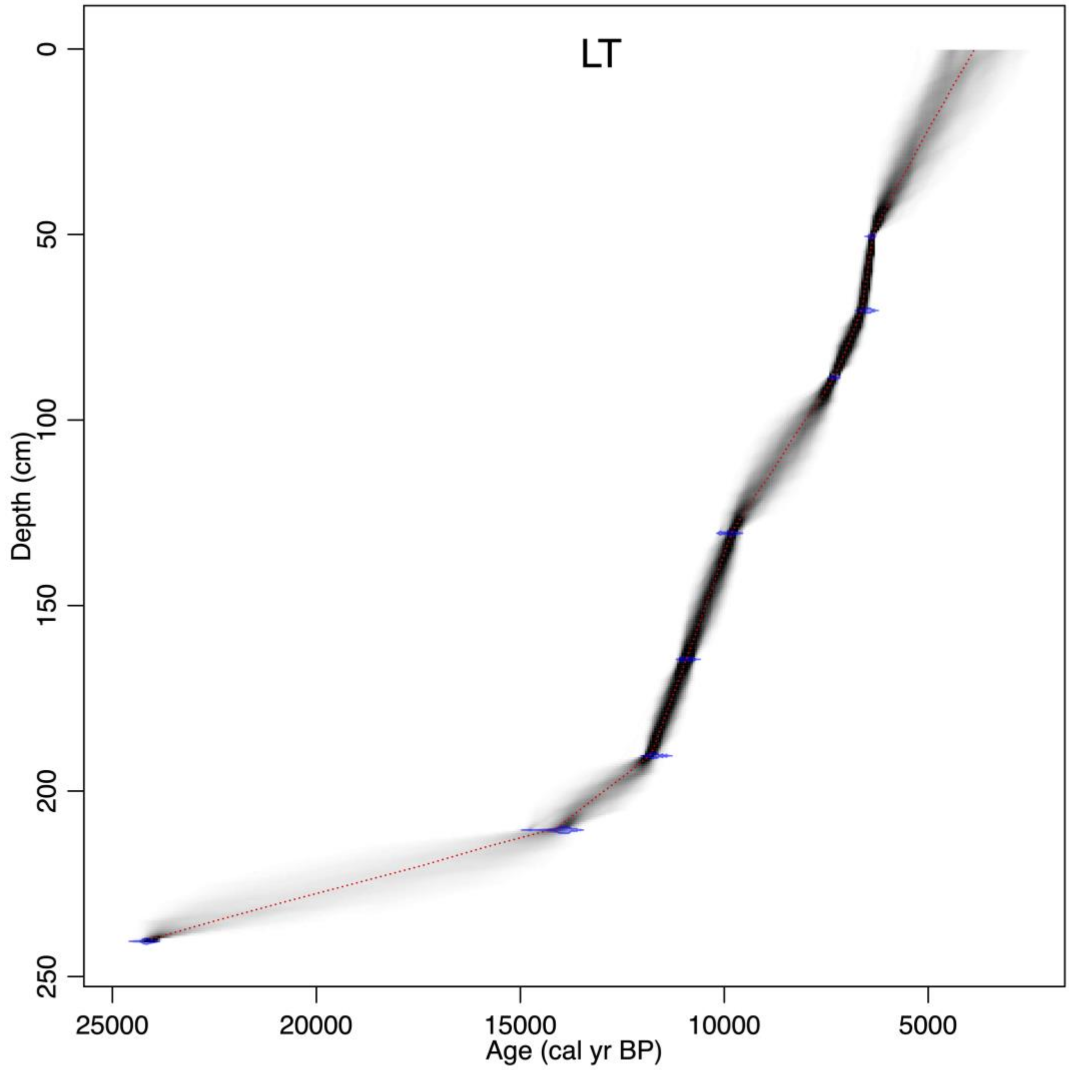

Supplementary Figure 3.2.6 | Age–depth model for site LT (Site ID 25, Lake Tvyordoe). Blue bars denote calibrated radiocarbon ages included for the model; red bars denote calibrated radiocarbon ages excluded; red line denotes the best-fitted model; grey shadow denotes the 95% confidence interval.

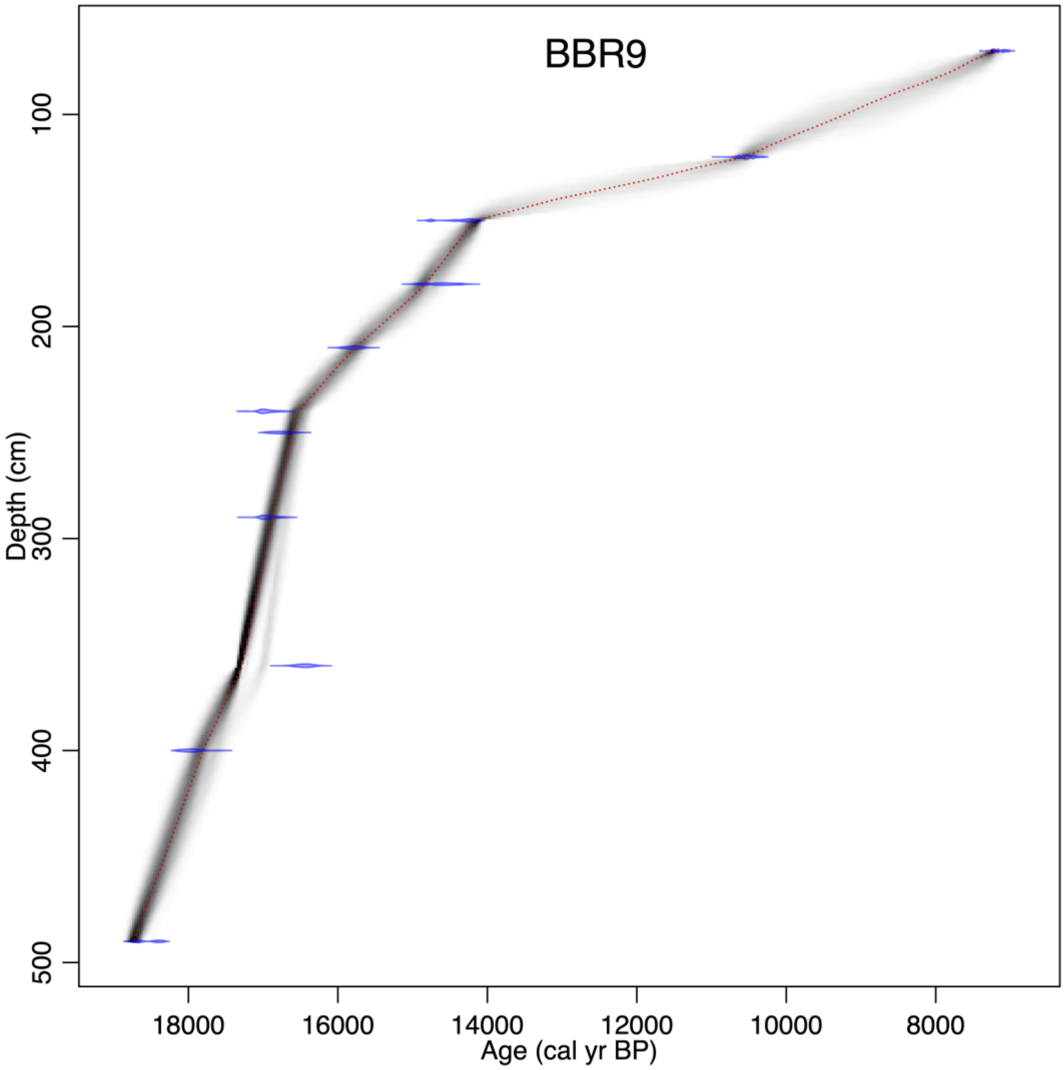

Supplementary Figure 3.2.7 | Age–depth model for site BBR9 (Site ID 26, Bol'shaya Balaknya River, site 9). Blue bars denote calibrated radiocarbon ages included for the model; red bars denote calibrated radiocarbon ages excluded; red line denotes the best-fitted model; grey shadow denotes the 95% confidence interval.

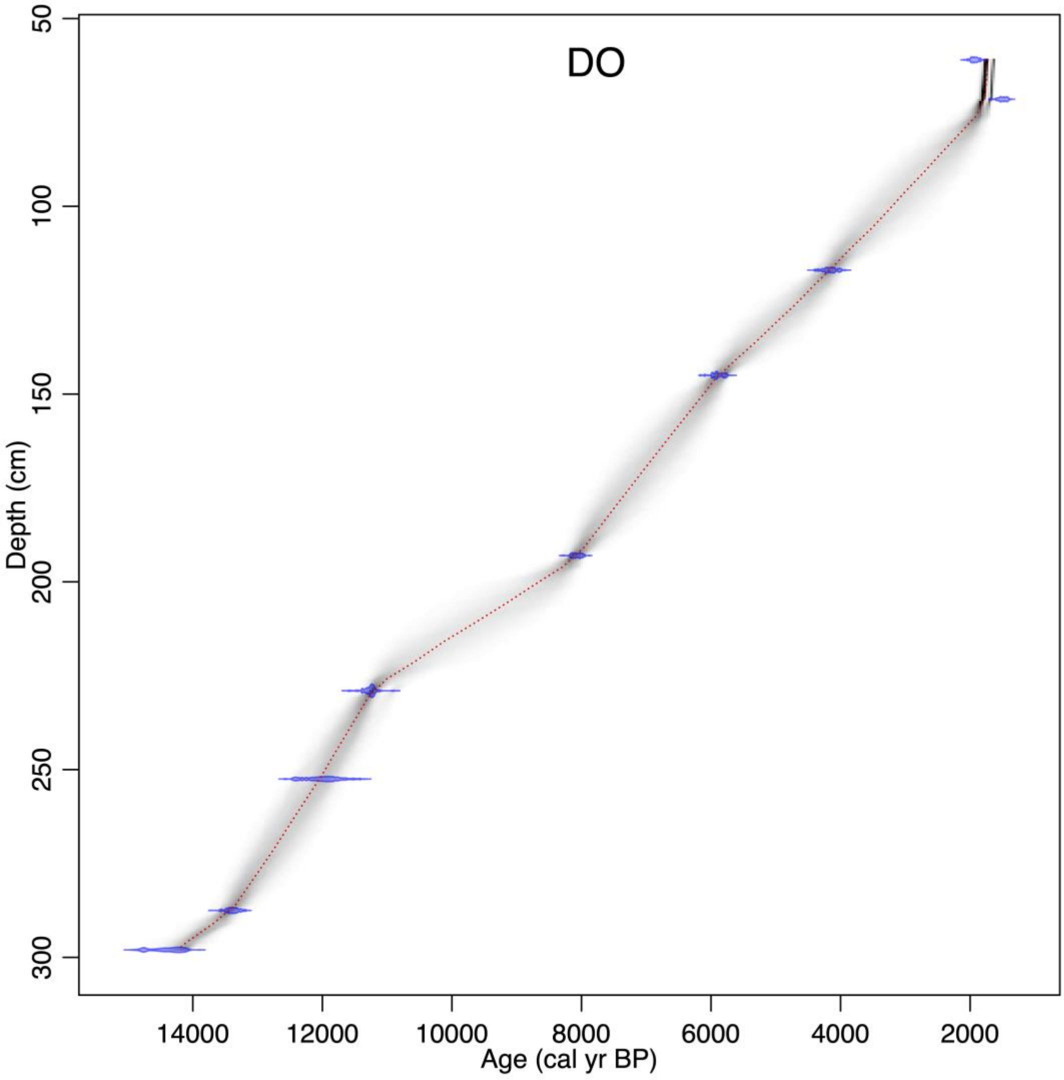

Supplementary Figure 3.2.8 | Age–depth model for site DO (Site ID 32, Dolgoe Ozero). Blue bars denote calibrated radiocarbon ages included for the model; red bars denote calibrated radiocarbon ages excluded; red line denotes the best-fitted model; grey shadow denotes the 95% confidence interval.

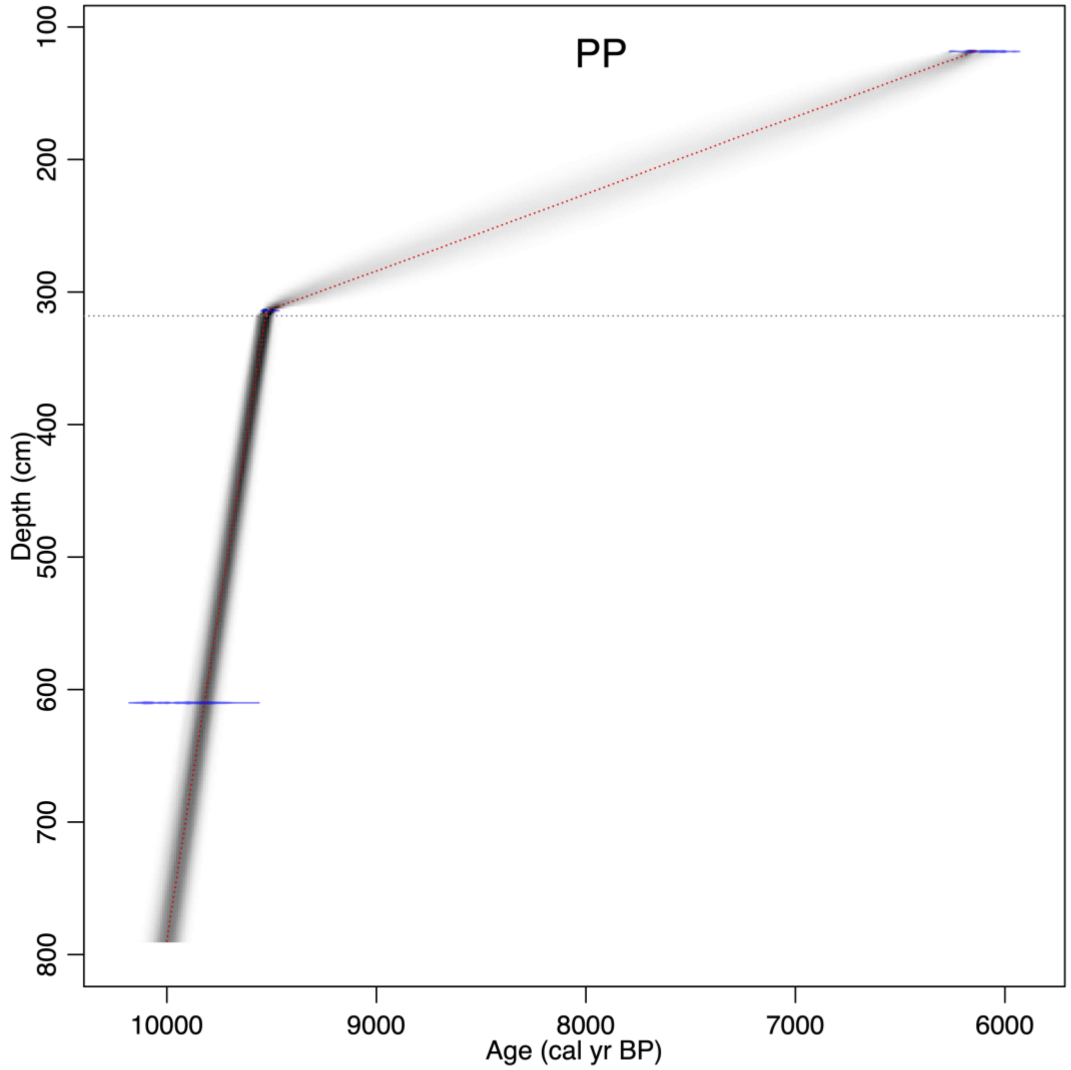

Supplementary Figure 3.2.9 | Age–depth model for the upper section of site PP (Site ID 41, Pleistocene Park). A hiatus was introduced at 318 cm. Blue bars denote calibrated radiocarbon ages included for the model; red bars denote calibrated radiocarbon ages excluded; red line denotes the best-fitted model; grey shadow denotes the 95% confidence interval.

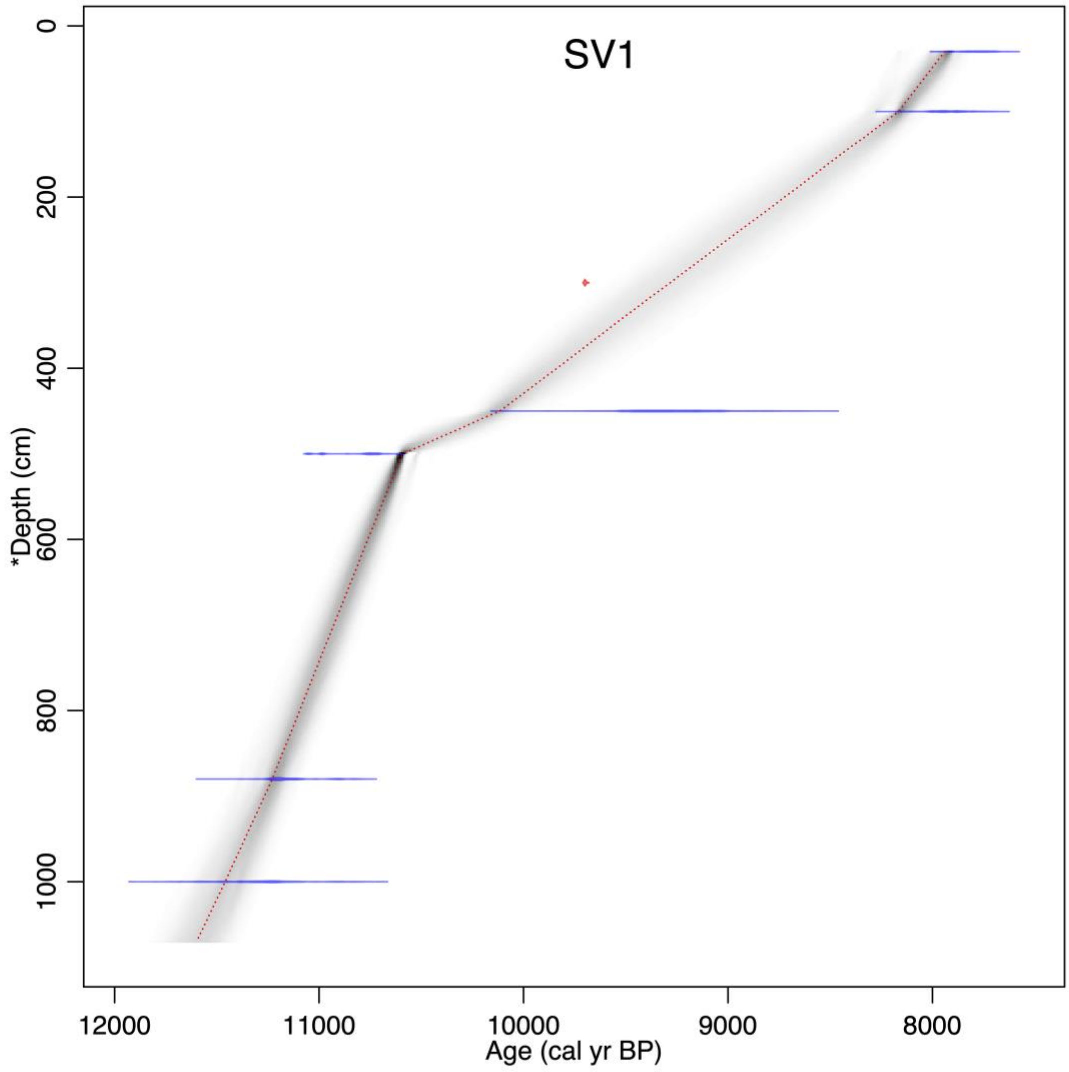

Supplementary Figure 3.2.10 | Age–depth model for site SV1 (Site ID 50, Stevens Village 1). Blue bars denote calibrated radiocarbon ages included for the model; red bars denote calibrated radiocarbon ages excluded; red line denotes the best-fitted model; grey shadow denotes the 95% confidence interval.

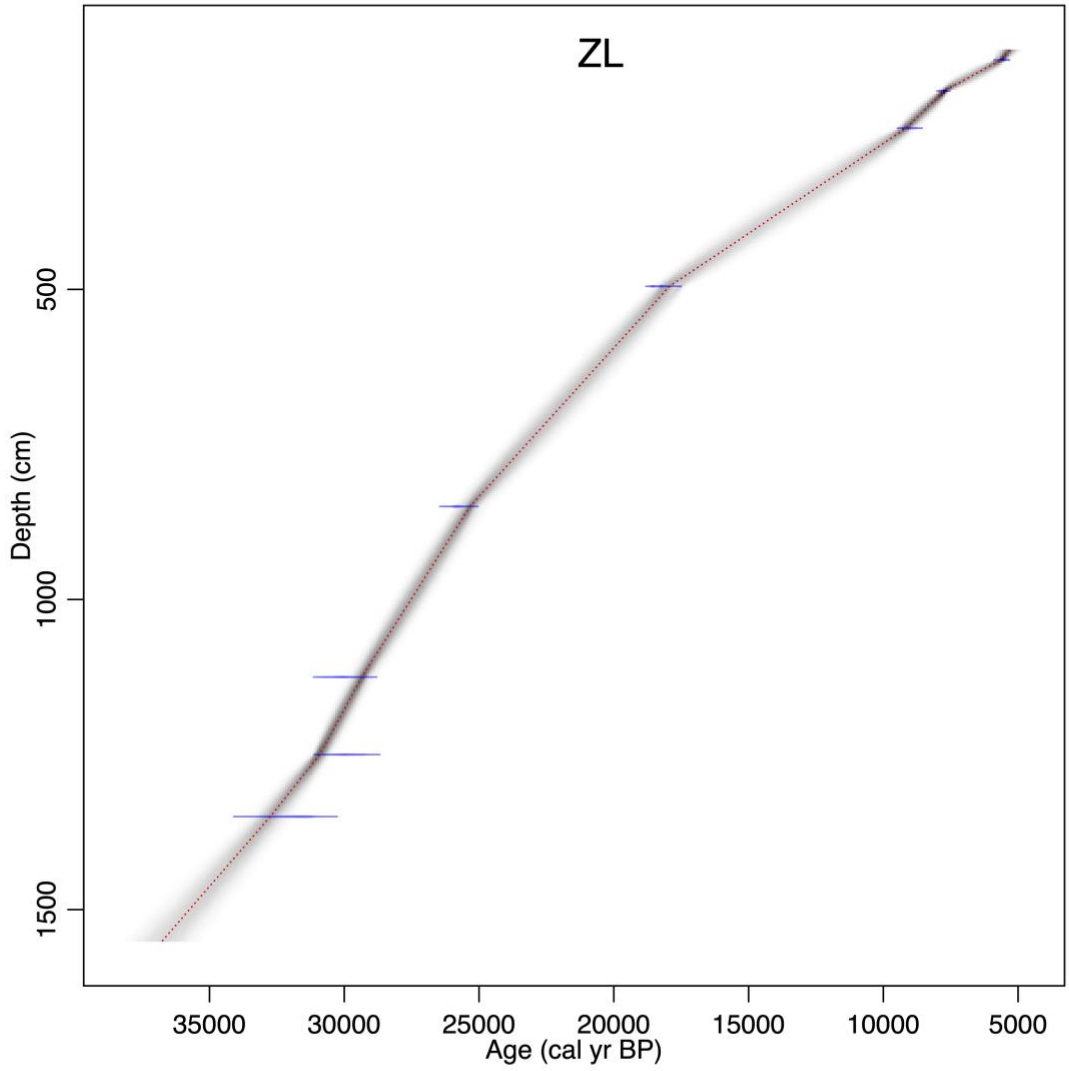

Supplementary Figure 3.2.11 | Age–depth model for site ZL (Site ID 54, Zagoskin Lake). Blue bars denote calibrated radiocarbon ages included for the model; red bars denote calibrated radiocarbon ages excluded; red line denotes the best-fitted model; grey shadow denotes the 95% confidence interval.

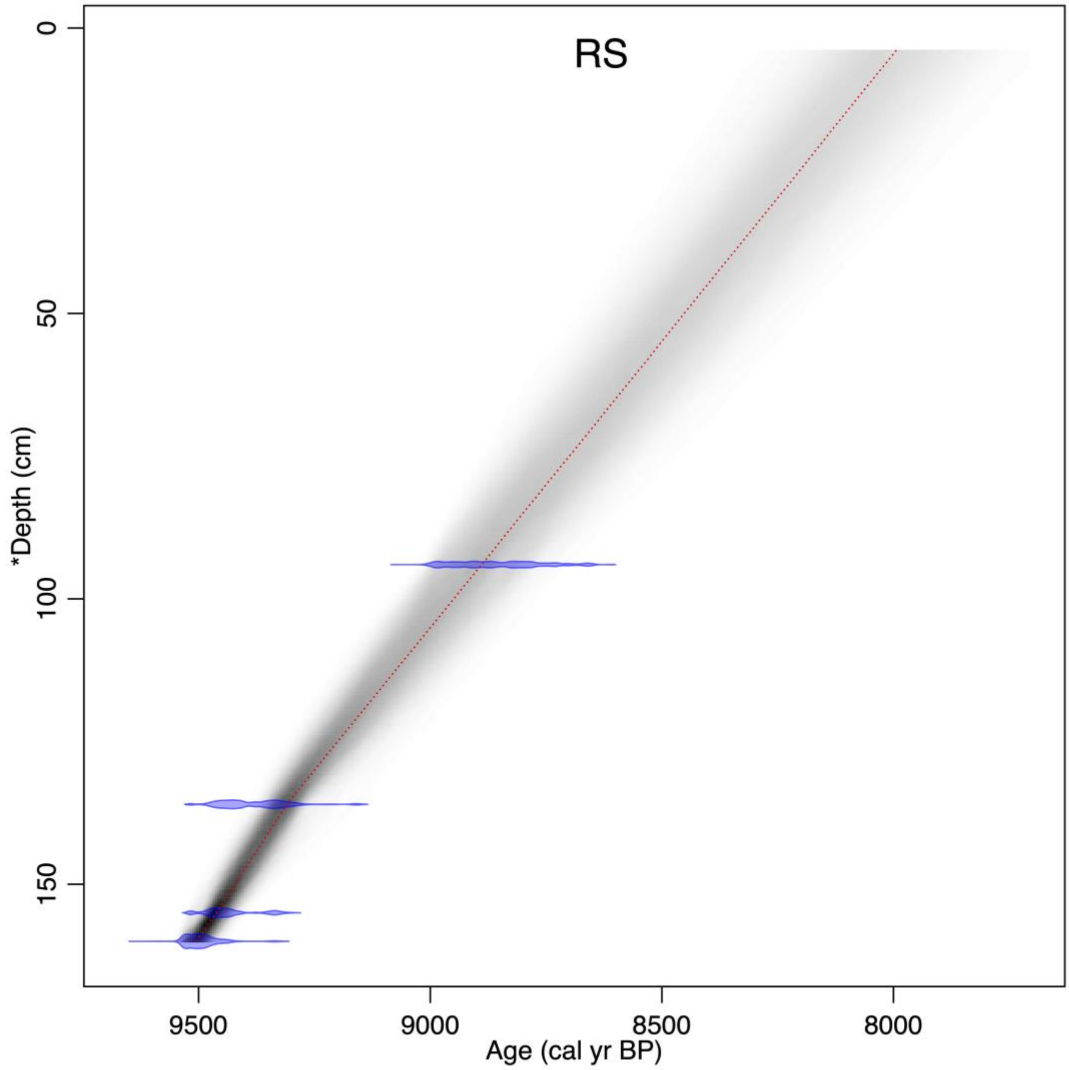

Supplementary Figure 3.2.12 | Age–depth model for the Unit 3 (U3) of site RS (Site ID 57, Ross Mine). Blue bars denote calibrated radiocarbon ages included for the model; red bars denote calibrated radiocarbon ages excluded; red line denotes the best-fitted model; grey shadow denotes the 95% confidence interval.

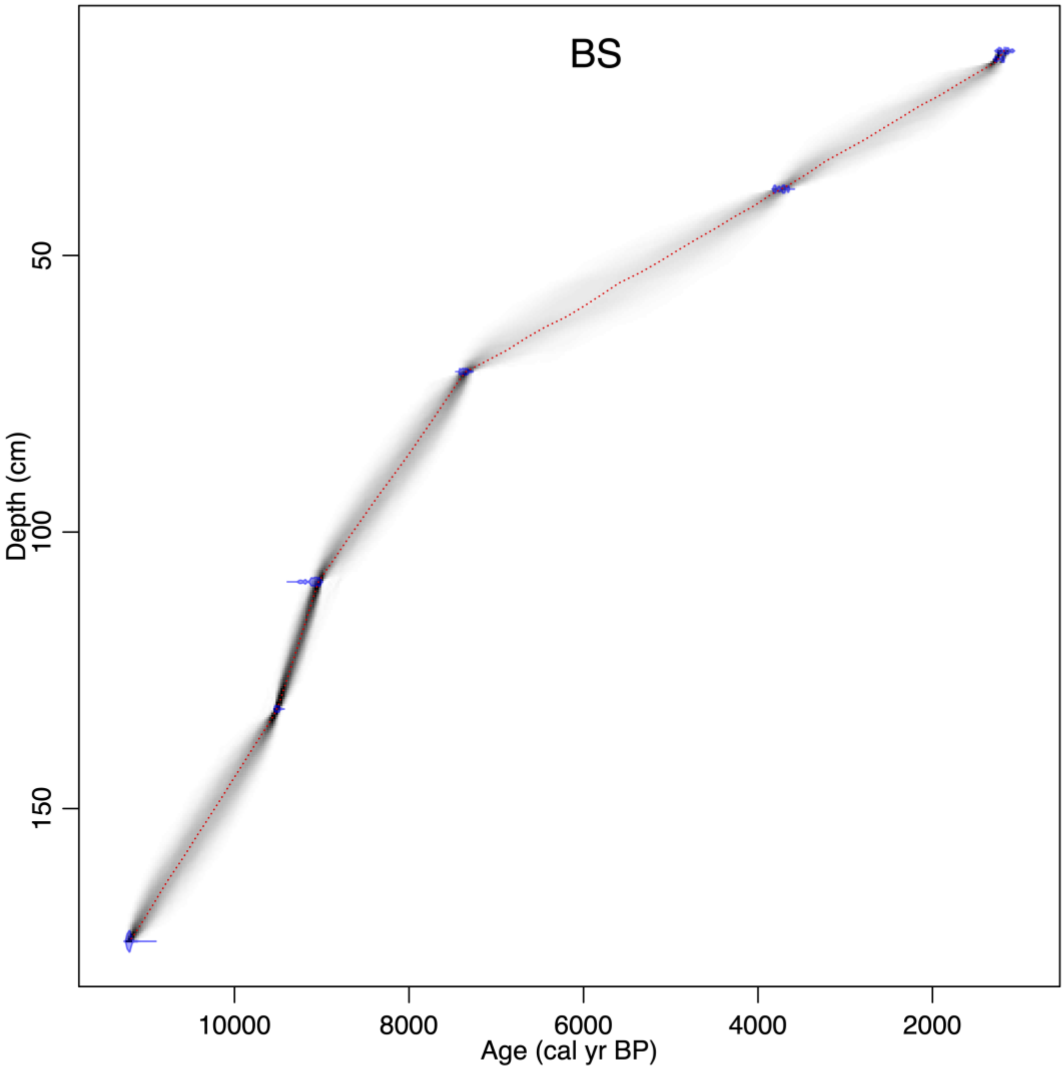

Supplementary Figure 3.2.13 | Age–depth model for site BS (Site ID 63, Baffin Island). Blue bars denote calibrated radiocarbon ages included for the model; red bars denote calibrated radiocarbon ages excluded; red line denotes the best-fitted model; grey shadow denotes the 95% confidence interval.

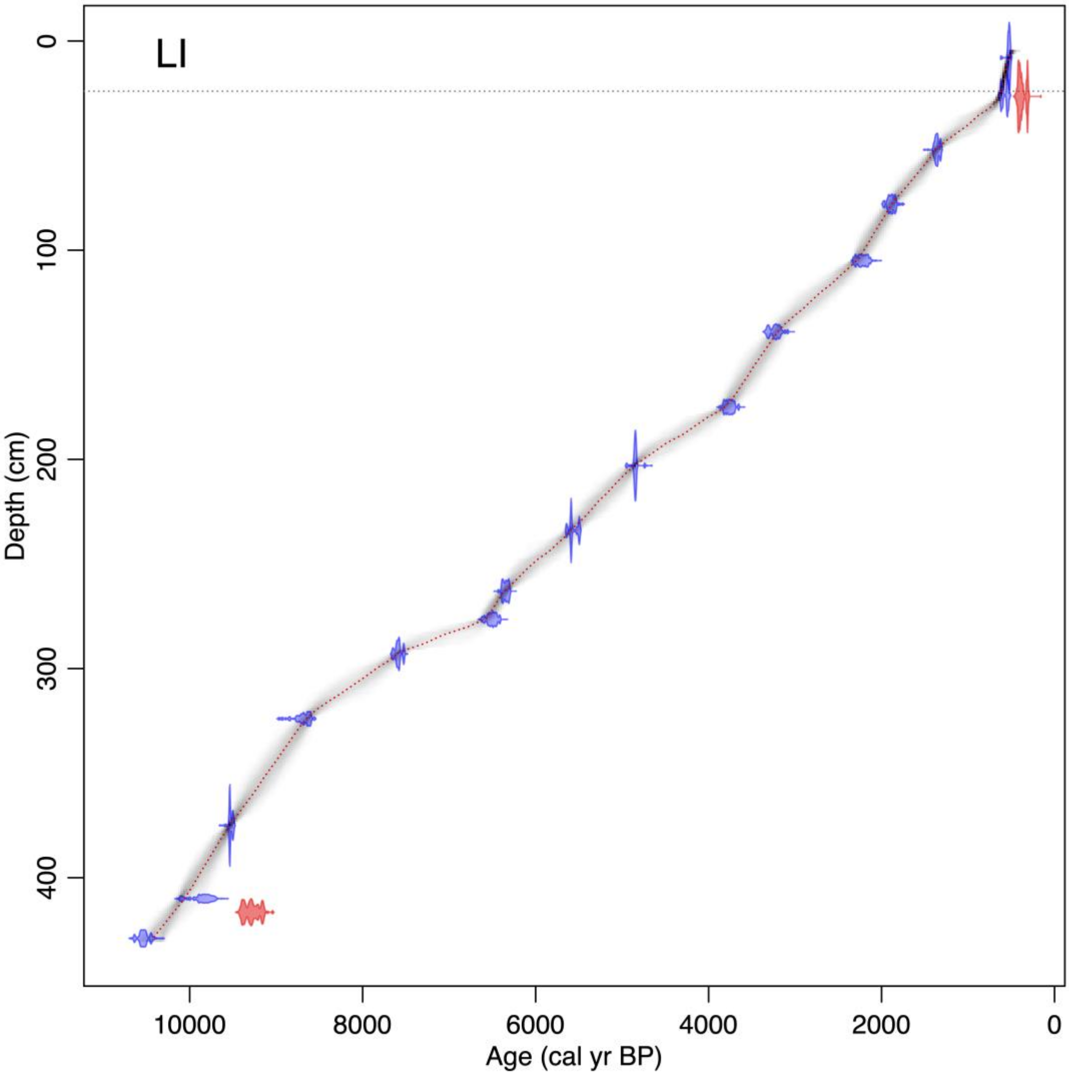

Supplementary Figure 3.2.14 | Age–depth model for site LI (Site ID 64, Lake Iversen). A hiatus was introduced at 24 cm. Blue bars denote calibrated radiocarbon ages included for the model; red bars denote calibrated radiocarbon ages excluded; red line denotes the best-fitted model; grey shadow denotes the 95% confidence interval.

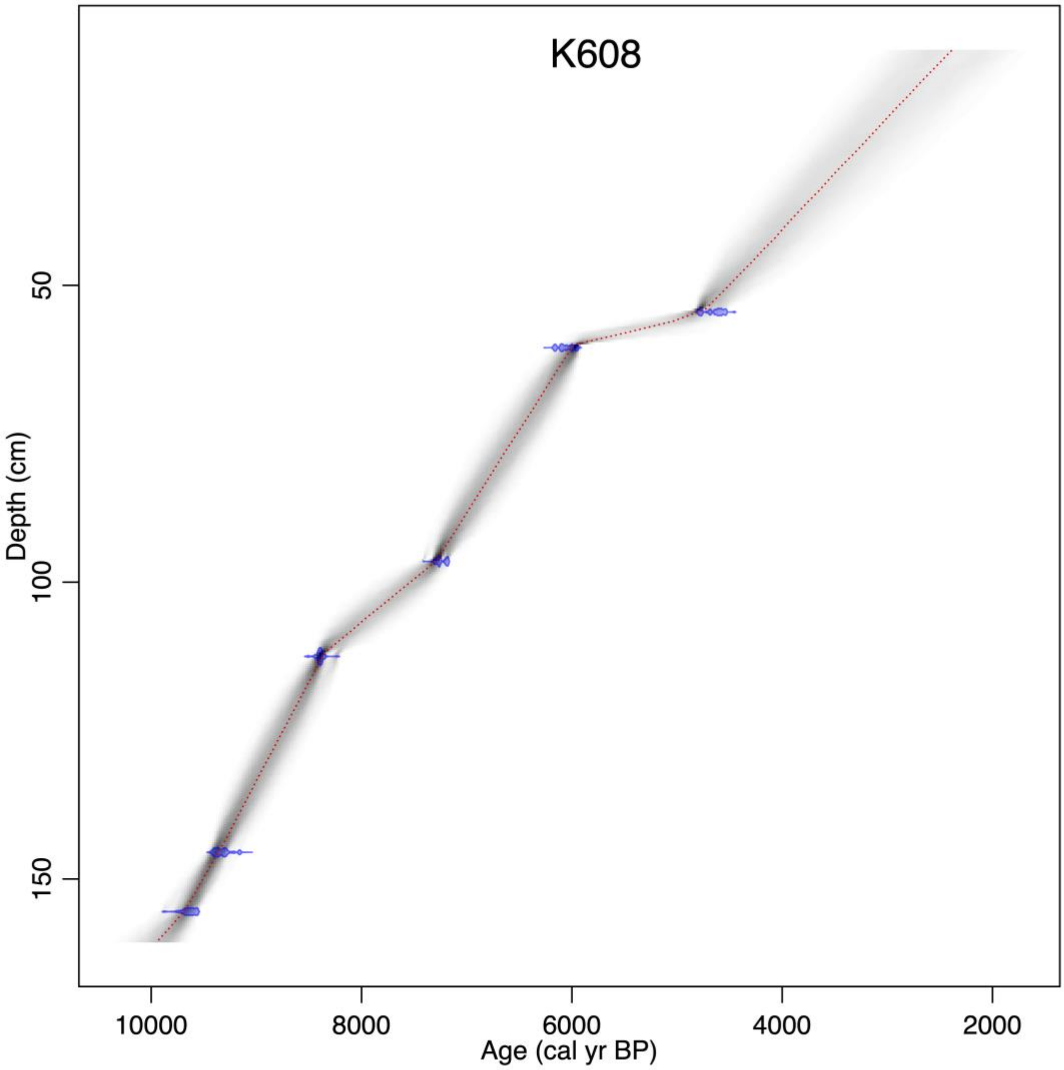

Supplementary Figure 3.2.15 | Age–depth model for site K608 (Site ID 65, Kan608m). Blue bars denote calibrated radiocarbon ages included for the model; red bars denote calibrated radiocarbon ages excluded; red line denotes the best-fitted model; grey shadow denotes the 95% confidence interval.

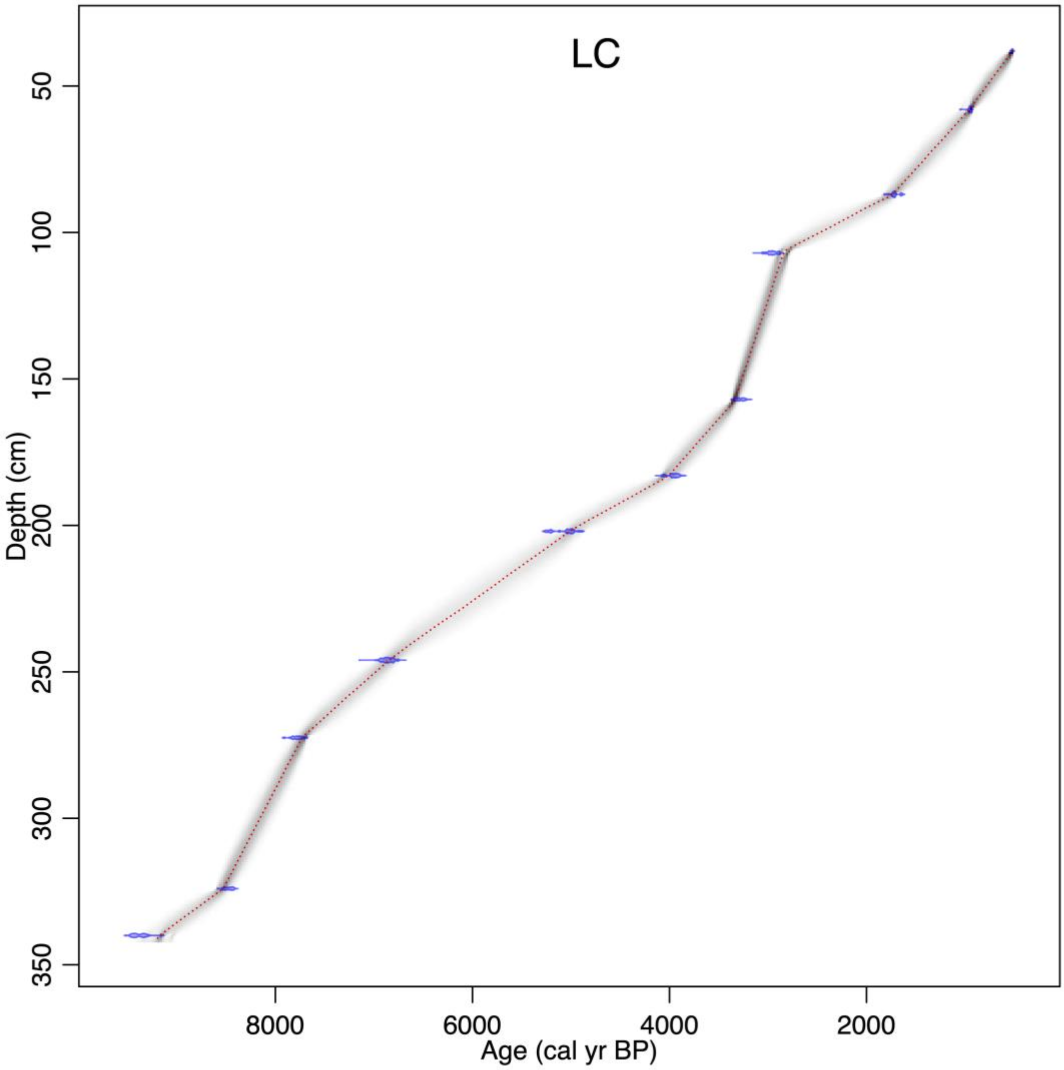

Supplementary Figure 3.2.16 | Age–depth model for site LC (Site ID 66, Lake Comarum). Blue bars denote calibrated radiocarbon ages included for the model; red bars denote calibrated radiocarbon ages excluded; red line denotes the best-fitted model; grey shadow denotes the 95% confidence interval.

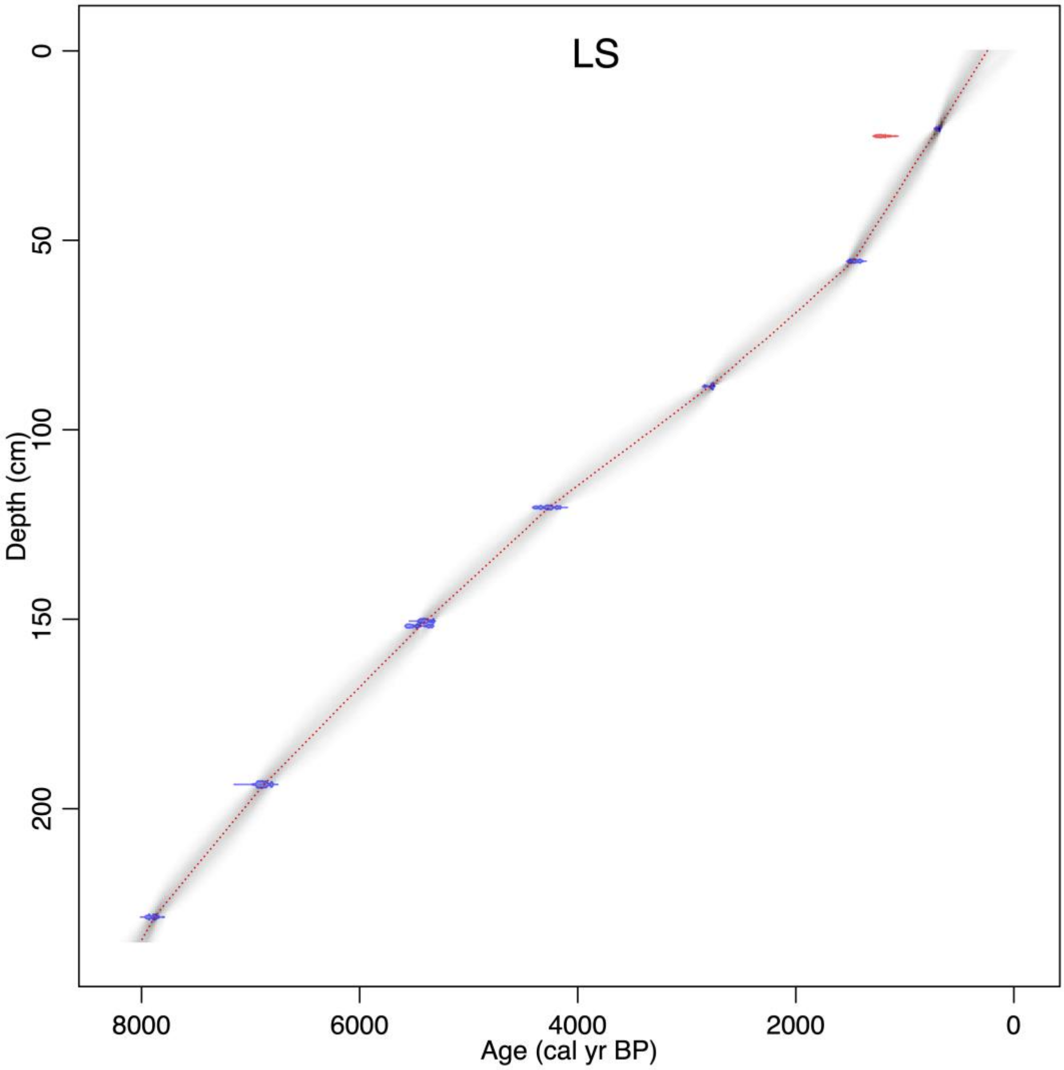

Supplementary Figure 3.2.17 | Age–depth model for site LS (Site ID 67, Lake Smaragd). Blue bars denote calibrated radiocarbon ages included for the model; red bars denote calibrated radiocarbon ages excluded; red line denotes the best-fitted model; grey shadow denotes the 95% confidence interval.

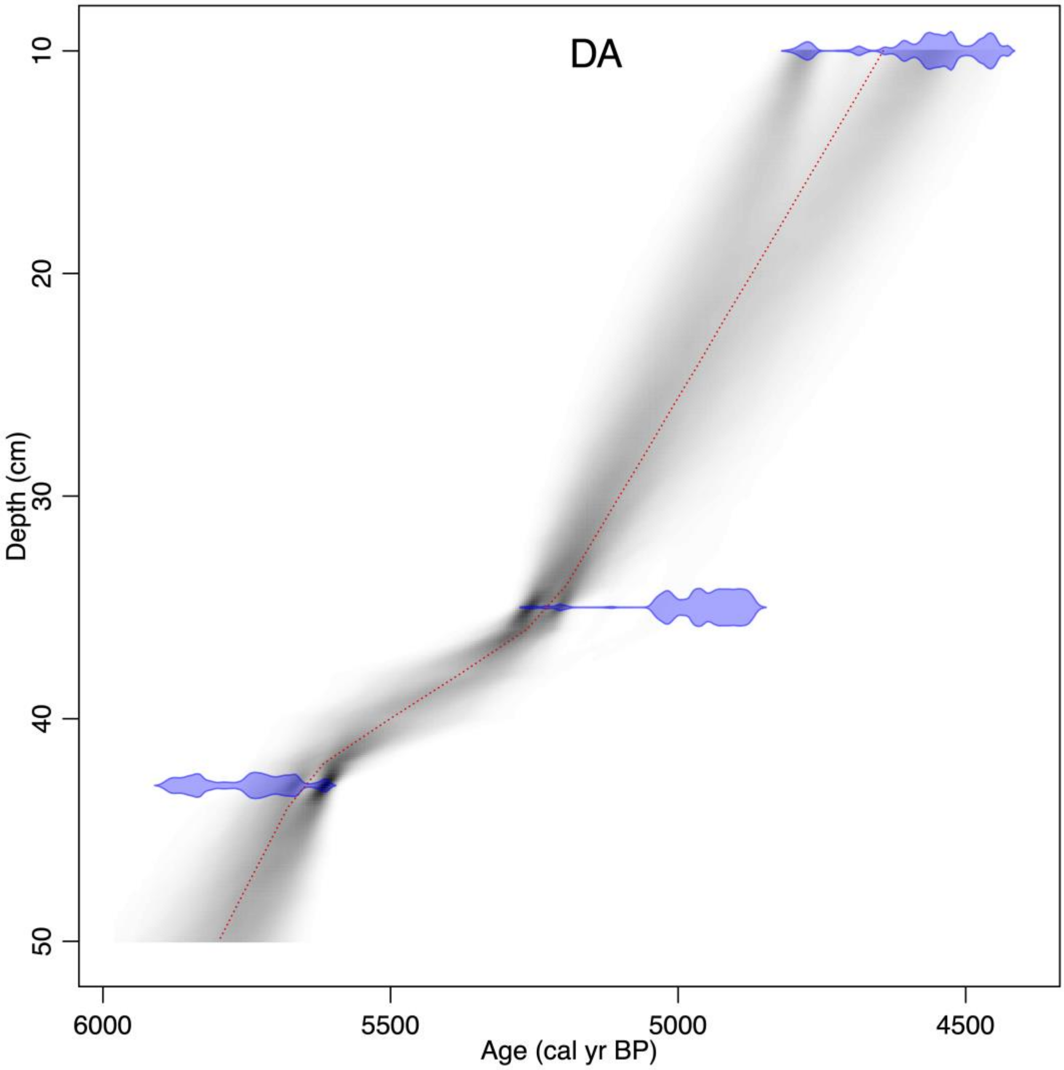

Supplementary Figure 3.2.18 | Age–depth model for site DA (Site ID 68, Danskøya). Blue bars denote calibrated radiocarbon ages included for the model; red bars denote calibrated radiocarbon ages excluded; red line denotes the best-fitted model; grey shadow denotes the 95% confidence interval.

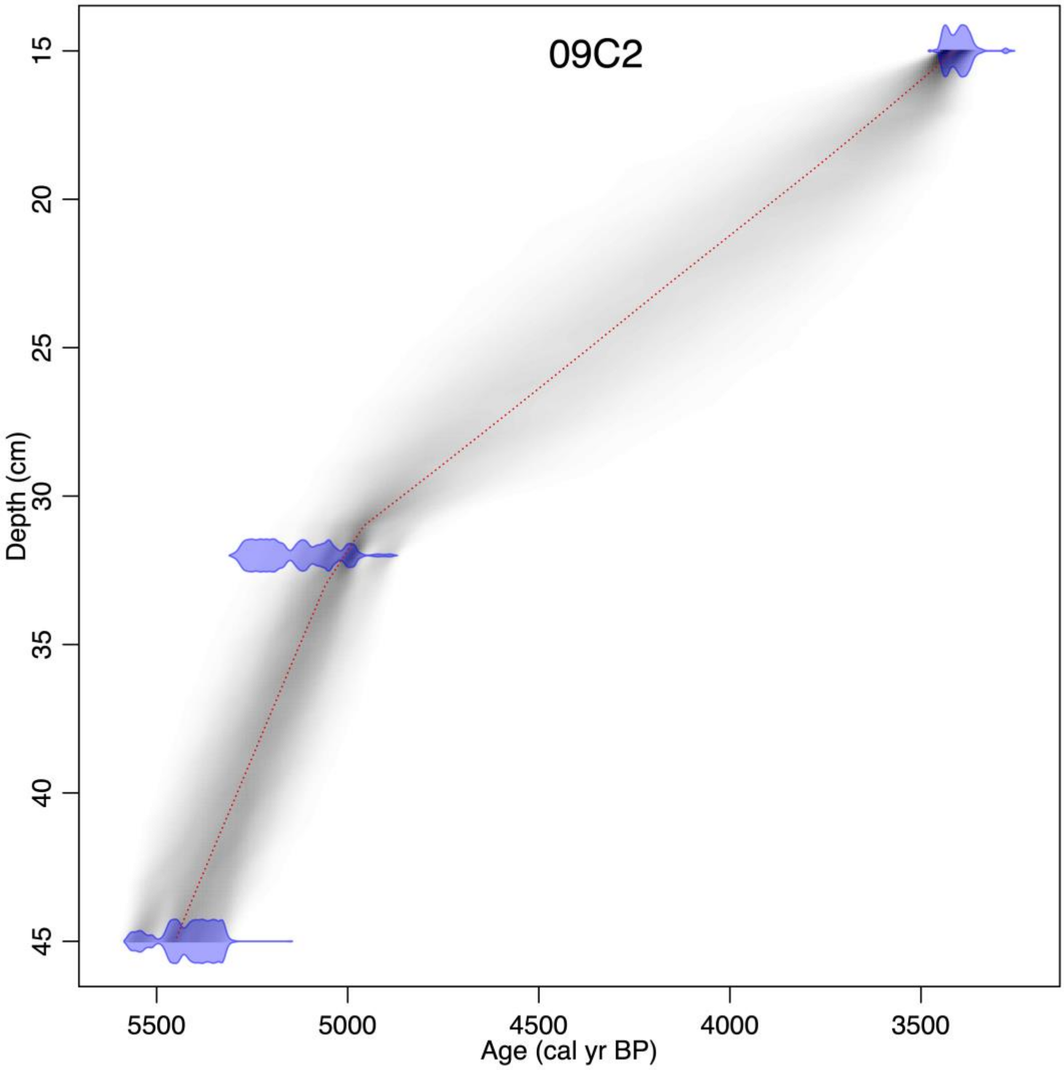

Supplementary Figure 3.2.19 | Age–depth model for site 09C2 (Site ID 71, Colesdalen, 2009 site2). Blue bars denote calibrated radiocarbon ages included for the model; red bars denote calibrated radiocarbon ages excluded; red line denotes the best-fitted model; grey shadow denotes the 95% confidence interval.

3.3 Chronology for the site with late surviving mammoth

There are a total of 15 samples younger than 10 ka that had mammoth DNA detected, sampled from 11 different sites from all the four regions across the Arctic (Supplementary Table 3.3.1). The chronology information for these sites can be found in the Supplementary Data 1 and 2, as well as the corresponded references. Here we further detail the chronological and sedimentological context for site LUR10 (Luktakh River, site 10, Site ID 14), from where the latest mammoths (3.9±0.2 ka) in our dataset was identified.

LUR10 is a 14 m high river section, exposing two sedimentary units (Supplementary Figure 3.3.1). The lower unit (unit A; c. 10 m) is at the lowermost part composed of ripple and planar parallel-laminated sand sets with organic debris in ripple troughs, organic intraclasts (3-10 cm in diameter) and wood pieces. Upwards the sand forms shallow trough-shaped units, often with organic coarse detritus (2-3 cm) beds at trough bottoms, interbedded with planar parallel-laminated sand. Unit A is interpreted as fluvial point-bar deposits. The upper part of the section (Unit B; c. 4 m) is, above an erosional unconformity, composed of very well-sorted planar parallel-laminated medium sand, rich distributed organic debris, debris mats and has indications of palaeo-soil development. Unit B is interpreted as an aeolian deposit. There is a conspicuous occurrence of upright standing Larix tree trunks at c. 8-9 m above river level, i.e., in unit A sediments. This represents a land surface invaded by larch forest, later killed off due to resumed sediment deposition.

Unit A is chronologically well constrained with two OSL dates and one radiocarbon date, all in an age succession within 5.2±0.3, 4.7±0.2 and 3.9±0.2 ka. The unit B aeolian deposits are recently deposited; OSL and radiocarbon dating in the upper part of the sediment give ages within the last centuries. DNA samples were collected from the unit A (n=3) in the same position as for the OSL and radiocarbon samples, as well as the top surface as control (Supplementary Figure 3.3.1). Mammoth DNA was only detected in one sample tm4_8 and shows DNA damage patterns (Supplementary Data 6).

Supplementary Table 3.3.1 | Samples younger than 10 ka with mammoth DNA detected.

Sample ID	Age (ka BP)	Site ID	Site abbreviation	Age interval	Region
tm4_8	3.9	14	LUR10	Late Holocene	Northwest and central Siberia
tm6_8	6.6	30	KS1	Mid-Holocene	Northwest and central Siberia
cr2_26	7.3	36	BK3	Mid-Holocene	Northeast Siberia
tm4_4	7.8	23	BBR7	Mid-Holocene	Northwest and central Siberia
cr9_2	7.8	32	DO	Mid-Holocene	Northeast Siberia
tm4_3	7.8	23	BBR7	Mid-Holocene	Northwest and central Siberia
tm9_4	8.1	35	BK2	Mid-Holocene	Northeast Siberia
ar5_23	8.6	50	SV1	Early Holocene	North America
cr8_11	8.9	1	06D1	Early Holocene	North Atlantic
ar3_30	9.4	57	RS	Early Holocene	North America
cr8_30	9.6	41	PP	Early Holocene	Northeast Siberia
ar1_5	9.6	18	BBR1	Early Holocene	Northwest and central Siberia
cr8_31	9.7	41	PP	Early Holocene	Northeast Siberia
cr8_33	9.8	41	PP	Early Holocene	Northeast Siberia
cr8_34	9.9	41	PP	Early Holocene	Northeast Siberia

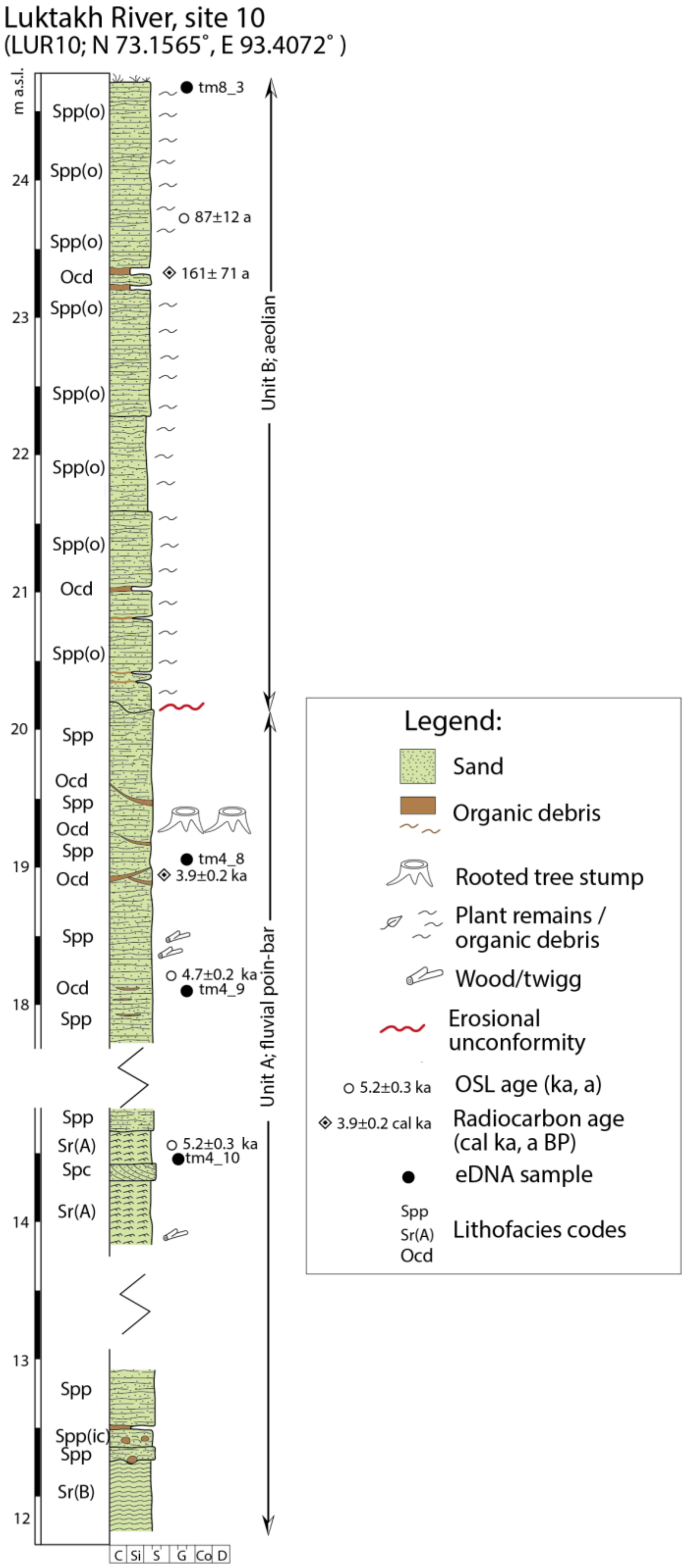

Supplementary Figure 3.3.1 | Sedimentary log for site LUR10 (Luktakh River, site 10, Site ID 14)

4. Contamination control

We used a concentrated synthetic short DNA fragment tracer for monitoring potential contamination introduced during sampling and subsampling (laboratory operations see Section 1.2). If the vector sequence appeared in the sequencing data of a sample, the sample was marked as contaminated and removed from the subsequent analysis (method see Section 9.1).

To avoid potential contaminations introduced during DNA laboratory operations, all DNA extractions and pre-indexed operations were performed in the dedicated ancient DNA laboratories at the Centre for GeoGenetics, University of Copenhagen, following established ancient DNA protocols[41-43].

Additionally, a total of 86 extraction and sequencing library building negative controls were introduced for 554 DNA extracts (some samples were extracted multiple times, see Supplementary Table 6.3.1). There was at least one control for every 7 extracts in each laboratory process. In order to trace potential contaminants from the reagents and DNA laboratory operations, all controls were processed together with samples under identical conditions throughout the complete process from subsampling to sequencing. Methods for dealing with the detected contaminants in controls are described in Section 9.3.

5. Sedimentary DNA taphonomy

While the actual state of ancient environmental DNA in soil and sediment samples remain unclear[44,45], it likely derives from two fractions. The first fraction is assumed to come from a complex mixture of preserved intracellular DNA deriving from above and below ground plant biomass, animal dung, urine, epidermal cells, hair, and bone fragments but not from pollen and macrofossils[11,42,46,47]. The second fraction consists of extracellular DNA that, upon cell decay, is released into the environment, fragmented, and thereafter bound to organic and inorganic particles that protect it against further degradations[48-50].

Organic material and DNA from low temperature regions and frozen arctic conditions have been found to be well preserved for hundreds of thousands of years[32,44,51]. These conditions, combined with the aeolian transport of sediment particles, facilitate the *in-situ*

accumulation of mineral components embedded with biological material and extracellular DNA, thus creating stratigraphic layers that correspond to different time periods[4].

Leaching and reworking of DNA across stratigraphic boundaries can hamper the reliability of ancient eDNA studies performed in temperate regions[52]. However, this has been shown to be inconsequential within permafrost[32], recently frozen/thawed sediment[47], or lake sediment[51,53]. As an additional precaution, we tested whether re-deposition was a factor by extracting and sequencing DNA from modern surface samples collected from different sites, *e.g.* ground surface of the permafrost sedimentary profiles, to test whether, *e.g.*, aeolian or snow-melt stream transport can introduce DNA from eroded older deposits. In addition, we tested water samples collected in rivers adjacent to sampled sediment outcrops to test whether flowing water can act as a similar vector. We found that the flora and fauna in these modern samples solely represents extant species. This provides additional support that either aeolian or stream re-deposition, nor rivers, pose a risk for false positives.

We then used the stratigraphic nature of the deposits to test whether vertical leaching within the profiles has occurred. Firstly, we extracted the identified megafauna list from layers immediately below and/or above where the megafauna DNA was found posterior than previous extinction dates. We found that in most cases megafauna DNA is not detected in these adjacent layers, suggesting that vertical leaching had not occurred, consistent with previous studies[20,46,54]. In addition, for sites where the floristic composition had been rebuilt based on pollen analysis, we compared the results to our DNA reconstructions and found closely corresponding results. Lastly, we observed that the species compositions were distinct between samples from the same profile but of different ages – further confirming that leaching or re-deposition has not occurred in these settings.

In one case, however, we detected mammoth DNA in surface samples of the river beach bellow the sampled river section of site BBR1 (Site ID 18, Bol'shaya Balaknya River, Site ID 1). However, the river beach is littered with bones of mammoth, bison and muskox (see Supplementary Data 1), indicating that this surface layer had been eroded and mixed with old sediment outcrops. We therefore marked all surface samples from the site as "disturbed" and discarded them from downstream analysis.

6. DNA laboratory methodology

We sought to find an optimal method for extracting and purifying DNA from all our samples, which are highly heterogeneous as they originated from a broad range of localities with different geologic contexts. To achieve this, we tested three different lysis buffers incorporated in the widely-applied ancient eDNA extraction protocols, and five purification methods on four selected samples with different sediment-organic compositions. Two extraction protocols performed equally well. For consistency, all samples were extracted using the same protocol, and thereafter built into sequencing libraries and sequenced on Illumina platforms. Tests and protocols are described in detail below.

6.1 Extraction test

Ancient DNA extraction from sediments can generally be split into two steps: (i) releasing extracellular DNA from sediment particles (organic and inorganic) and ancient intracellular materials into lysis buffer and (ii) purifying the DNA by chemical or physical removal and/or precipitation of non-DNA components. While these are common for all nucleic acid isolations, what complicates our analysis are that ancient DNA is often very low in concentration and typically highly degraded (< 150 base pairs)[45,55].

Most commercial extraction protocols are designed for retrieval of high concentrated, long DNA molecules from fresh materials. These protocols therefore include 'aggressive' treatments that enhance DNA release and purity by using high incubation temperatures, silica binding filters and strong detergents, therefore unsuitable for ancient DNA, as preserving the short, fragmented DNA molecules is crucial, and introducing further damage on the DNA should be avoided.

Sediment samples are conglomerates of complex components. A variety of inhibiting substances coexist with DNA in the samples, such as humic acid, cell debris and proteins. These substances can reduce the DNA purity and obstruct or even completely block the subsequent enzymatic reactions. Isolation of DNA from these conglomerates is complicated as some inhibiting components have a similar molecular weight and polarity as DNA molecules[56], resulting in the co-extraction of inhibitors together with DNA.

Purification of ancient eDNA is therefore a fine balance between removing inhibitors while still retaining sufficient short ancient DNA molecules for analysis.

6.1.1 Test content

In total, we identified five variables that potentially influence the DNA extraction yield: (i) lysis buffer, (ii) incubation time, (iii) DNA up-concentration, (iv) purification methods and (v) sediment composition. In order to optimize DNA yield and purity from our samples, we therefore tested (ii) three lysis buffers, (ii) two incubation durations, (iii) the effects of applying DNA up-concentration, and (iv) five purification methods on (v) four different sediment types. A list of tests is shown in Supplementary Table 6.1.1 and details are described in below.

The three tested buffers are: (i) the EDTA/Tris-HCl buffer [50mM EDTA (pH 8.0), 50mM Tris-HCl (pH 8.0), 150mM NaCl, 2.5% N-Lauroylsarcosine and 500mM mercaptoethanol] that was originally designed for extracting DNA from modern sediment, but had been shown also effective for releasing DNA from ancient sediment[33,57], (ii) the Sodium-Phosphate buffer (0.5M Sodium-Phosphate at pH 7.0) that has been reported successful in isolating DNA from lake sediment and archaeological cave sediment[58,59], and (iii) the EDTA buffer (0.5M EDTA at pH 8.0) that is commonly used for recovering DNA from skeletal specimens, but is also effective for cave sediments[59,60].

Longer incubation time has been suggested for enhancing DNA release and thus increasing the yield[33]. However the incubation time in published protocols varies from 30 minutes[59] to a few hours[41], and up to overnight[33]. To confirm the benefits of longer incubation, we tested the performance of 30 minutes and overnight incubation.

Amicon Ultra Centrifugal Filters are applied broadly in molecular biology for up-concentrating DNA by reducing the solution volume from milliliters to microliters[61-63], and has also been reported effective for increasing ancient eDNA extraction yield[33]. We therefore evaluated the effects of up-concentration on the DNA yield by using the Amicon Ultra Centrifugal Filters (30 kDa).

Five purification modules were combined into five different purifying methods (see Supplementary Table 6.1.1). The five modules include: (i) MinElute (Qiagen MinElute PCR Purification Kit) which is a commonly used DNA purification kit in molecular

biology, (ii) Phenol-Chloroform [UltraPure Phenol:Chloroform:Isoamyl Alcohol (25:24:1, v/v)], a widely used reagent for separating nucleic acids from proteins and lipids, (iii) PowerMax C2 and C3, two inhibitor-removal reagents that are incorporated in the DNeasy PowerMax Soil DNA Isolation Kit (MO BIO) which had been reported effective for removing impurities from DNA extracts of sub-Arctic lake sediment[33] and frozen midden deposits[57], (iv) InhibitEx tablet (Qiagen), which is designed for precipitating non-DNA components from DNA extracts, and (v) the OneStep PCR Inhibitor Removal Kit (Zymo Research), of which the Zymo-Spin III-HRC Column is designed to retain non-DNA inhibitors.

Supplementary Table 6.1.1 | Sample types, lysis buffers and purification methods tested for optimization of the ancient eDNA extraction protocol.

Sample types	Lysis buffers	Purification methods
Organic-rich clay	EDTA/Tris-HCl	MinElute
Clay	Phosphate	Phenol-Chloroform + MinElute
Peat	EDTA	Phenol-Chloroform + Powermax C2 and C3
Sandy peat		Phenol-Chloroform + inhibitex tablet+MinElute
		Phenol-Chloroform + Zymo-Spin + MinElute

6.1.2 Test procedures

Given that the MinElute and Phenol-Chloroform purification normally do not appreciably reduce DNA concentration, we applied the first two purification methods for evaluating other variables first, in the following four steps.

(1) Precisely weigh 1 g (± 0.01) of sediment into a sterile 15 mL spin tube for each of the 4 sample types. Prepare and heat the 3 buffers to 37 °C. Add 6 mL of each buffer together with 80 µL proteinase K (100µg/µL) to each sample. Homogenize sample and buffer using a FastPrep-24 for 2 rounds at 4.5 m/s speed for 20 s. Rotate the reactions at 37 °C for 30 min. Centrifuge at 4000 x g for 10 min and subsample 2 mL of the supernatant from each reaction into a 15 mL spin tube (set 1).

(2) Rotate the remaining samples in lysis buffer overnight at 37 °C. Centrifuge all samples at 4000 x g for 10 min and subsample 2 mL supernatant of each sample into two new 15 mL spin tubes, respectively as set 2 and 3. Add 5 mL Phenol-Chloroform to each of the set

3 samples, and rotate gently for 10 minutes at room temperature. Centrifuge at 4000 x g for 5 minutes and transfer supernatant to clean 15 mL spin tubes.

(3) Subsample 100 µL from all samples into each of the 3 sets and purify using a Qiagen MinElute PCR purification kit following the manufacturer's instructions but with two modifications: (1) modify 500 mL Qiagen PB buffer by adding 20mL 3M NaoAC pH 5 (v/v 1:25) and 0.52 mL phenol red (v/v 1:1000) and (2) elute the DNA in 15 µL Qiagen EB buffer.

(4) Transfer the remaining solution from all 3 sets to 30 kDa Amicon Ultra-4 Centrifugal Filters, and centrifuge at 4000 x g until ~100 µL of retentate left on the filter. Exchange buffer by washing the filter 2 times with 750 µL Qiagen EB buffer and centrifuge to 100 µL left on the filter for each run. Purify the retentate using the Qiagen MinElute protocol as in step 3.

These 4 steps generated 6 experimental treatments (Supplementary Table 6.1.2), on 3 buffers, 4 sample types and 1 negative control, totally 90 measurements. The performances were evaluated by (i) DNA concentration measured by Qubit 3.0, (ii) qPCR amplification of Illumina library (if possible) and (iii) molarity of target size DNA molecules (100-1000 base pairs) measured by Agilent Bioanalyzer 2100 High-sensitive chip.

Supplementary Table 6.1.2 | DNA extraction testing treatment.

Treatment	Set	Amicon up-concentration
1	Set 1 (30 minutes incubation)	No
2	Set 1 (30 minutes incubation)	Yes
3	Set 2 (overnight incubation)	No
4	Set 2 (overnight incubation)	Yes
5	Set 3(as set 2 + Phenol-Chloroform purified)	No
6	Set 3(as set 2 + Phenol-Chloroform purified)	Yes

We found, on average, that samples incubated overnight recovered ~3.8 fold more DNA than the samples incubated for 30 minutes. Additionally, DNA concentrations increased by 9.63, 2.80 and 2.19 folds after Amicon millipore filter up-concentration, for set 1, 2 and 3 respectively. For samples purified by Phenol-Chloroform (set 3), we found an additional 1.94 fold yield increase, which may result from the cleaner extracts giving in better Qubit measurements. See Supplementary Figure 6.1.1 for details. An overnight incubation,

together with Amicon filter up-concentration and Phenol-Chloroform purification therefore are recognized as optimal.

For the buffer efficiency, on average EDTA/Tris-HCl buffer treated samples yielded 2.47 ng/µL DNA, which is 1.45 and 1.46 folds higher than Sodium-phosphate and EDTA buffer, respectively. In addition, the EDTA/Tris-HCl buffer performed better for the clay sediments, releasing 1.63 and 5.24 folds more DNA than the other two buffers, but showed no superiority for the peat sediment. However, with the overnight incubation combined with Amicon filter up-concentration and Phenol-Chloroform purification, EDTA/Tris-HCl buffer treated peat samples yielded an average of 1.62 ng/µL DNA, which is sufficient for downstream analysis. The EDTA/Tris-HCl buffer was therefore selected as the lysis buffer.

Despite Phenol-Chloroform and MinElute purifications, all extracts were still dark colored, indicating the extracts still containing excess amounts of non-DNA components[64-66]. And as these colored extracts also failed to be measured on BioAnalyzer or converted into Illumina libraries, we decided that further purifications were required.

An additional test was therefore set up to compare to the performance of the other 3 purification methods, via the following 4 steps:

(1) Precisely weigh 1 g (± 0.01) sediment from each of the 4 sediment types. Prepare and heat the EDTA/Tris-HCl buffer to 37 °C. Add 6 mL lysis buffer and 80 µL proteinase K (100µg/µL) to each sample. Homogenize the sample and buffer using MP Bio FastPrep-24 for 2 rounds at 4.5 m/s speed for 20 s. Incubate these reactions by rotating at 37 °C overnight. Each sample is then centrifuged at 4000 x g for 10 min and 2ml is subsampled into 3 clean tubes. Each subsample is then subjected to one of the three purification methods following 2a, 2b and 2c.

(2a) Add 1.5 mL C2 buffer to the 2 mL extract and mix by vortexing. Incubate the reaction at 4 °C for 10 minutes and centrifuge at 4000 x g for 3 minutes. Transfer the supernatant to a new tube and add 1.25 mL buffer C3. Repeat the incubation and centrifuge at 4000 x g for 3 minutes. Purify the reaction using 5 mL Phenol-Chloroform and up-concentrate the reaction with an Amicon filter as described above. Wash the filter twice with Qiagen EB buffer and finally centrifuge until 50 µL is retained on filter.

(2b) Purify the 2 mL extracts with Phenol-Chloroform as described above and transfer the supernatant into 2.5 mL Qiagen buffer ASL. Add half a Qiagen InhibitEx tablet into each reaction and vortex immediately for 5 minutes. Incubate the reaction for 2 minutes at room temperature and then centrifuge at 5000 x g for 5 minutes. Transfer the supernatant to a clean tube and centrifuge again at 5000 x g for 5 minutes. Concentrate the supernatant through an Amicon filter until 100 µL is retained. Purify the retentate left on filter using MinElute and elute by 35 µL Qiagen EB buffer.

(2c) Purify the 2 mL extracts with Phenol-Chloroform and up-concentrate the supernatant using an Amicon filter until less than 100 µL remains on the filter. Purify the retentate on filter using a Zymo-Spin III-HRC column following the manufacturer's instructions. Purify the flow-through using a MinElute and elute in 35 µL Qiagen EB buffer.

We found that after any of the three additional purifications, all extracts became transparent and could be successfully converted into sequencing libraries and measured by bioanalyzer. The performance of the purifications was therefore evaluated based on the DNA concentration and molarity of target size DNA molecules (100 - 1000 base pairs). We found that InhibitEx tablets (2b) and Zymo-Spin columns (2c) purifications performed better than PowerMax C2 and C3 (2a), yielding higher DNA concentrations and more target size DNA molecules in the final extracts (Supplementary Figure 6.1.1 e and f). InhibitEx tablets purification performed better for the organic-rich clay sample, but overall both the 2a and 2b purification methods yielded sufficient DNA for subsequent analysis.

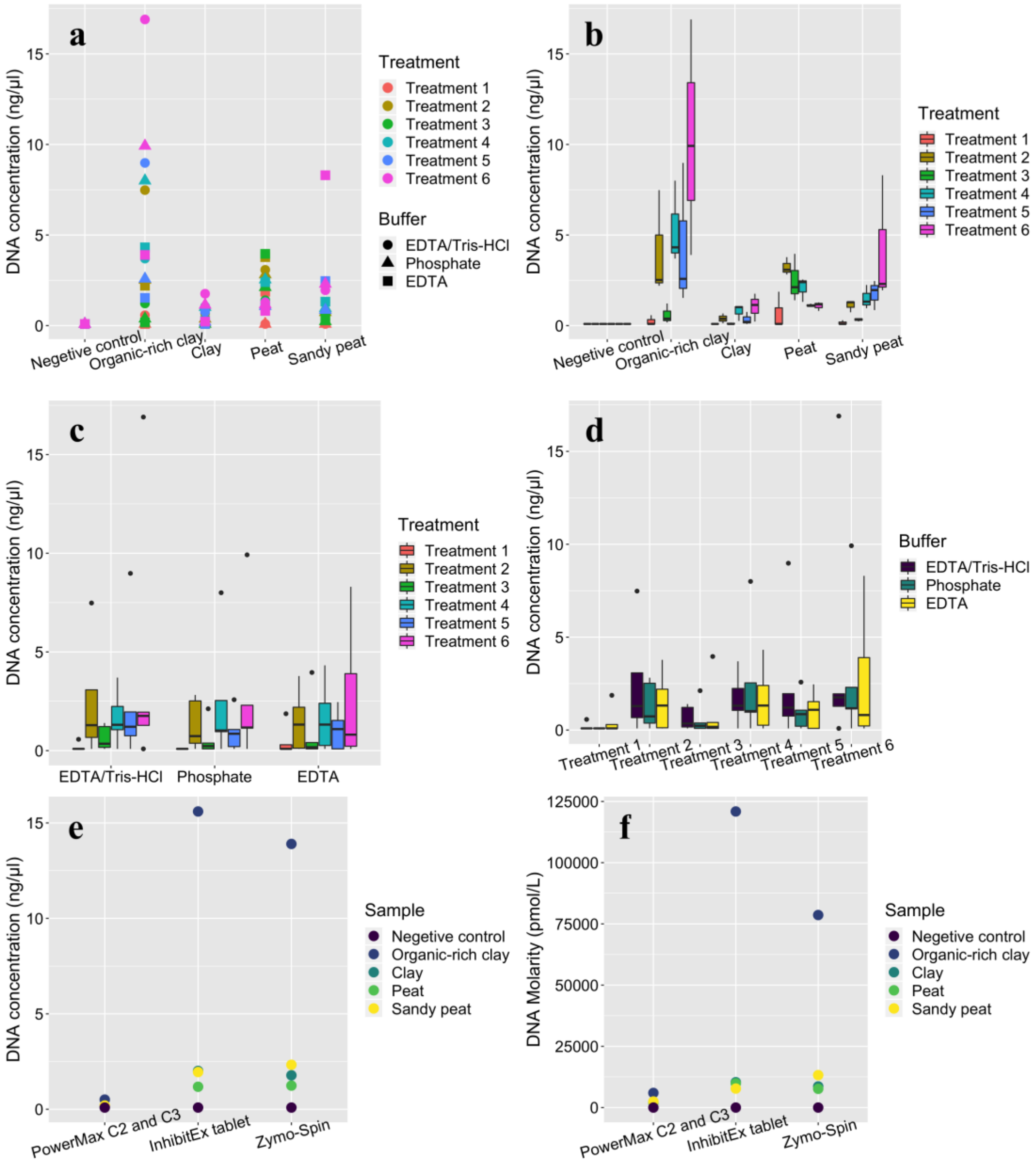

Supplementary Figure 6.1.1 | DNA concentration and molarity (100-1000 bp) in different extraction tests. For boxplot, the bar denotes the median; the lower and upper hinges denote the 25th and 75th percentiles, respectively; the upper and lower whisker extended from the hinge denote the largest value no further than 1.5 * IQR (inter-quartile range) and the smallest value at most 1.5 * IQR of the hinge, respectively; data beyond the end of the whiskers are shown as outliers. **a,** DNA yields by different extraction buffers and treatments on the 4 sediment types. **b,** DNA yields by different treatments on the 4 sample types (n=3 extraction buffers). **c,** DNA yields by different treatments on the 3 extraction buffers (n=4 sample types). **d,** DNA yields by different extraction buffers on the

6 treatments (n=4 sample types). **e and f**, DNA yields from the 3 additional purification methods.

6.2 Extraction protocols

Based on the above tests, two optimized ancient eDNA extraction protocols were developed as described below. The InhibitEx-based protocol was applied for extracting DNA for all samples in this study.

6.2.1 InhibitEx based protocol

(1) Prepare the lysis buffer stock by mixing 50 mM EDTA (pH 8.0), 50 mM Tris-HCl (pH 8.0), 150 mM NaCl, 2.5% N-Lauroylsarcosine. Add 500 mM mercaptoethanol, 500 mM DTT and 0.5 mg/mL Proteinase K to the stock immediately before use.

(2) Add 3 mL lysis buffer to 0.5-5 gram wet sediment sample. Vortex for 2 x 20 seconds at 4.5 m/s on a FastPrep (15 x12 module).

(3) Rotate the reactions at 37 °C overnight and centrifuge at 4000 x g for 10 min. Transfer the supernatant to 5 mL UltraPure Phenol:Chloroform:Isoamyl Alcohol (25:24:1, v/v) and rotate for 20 minutes at room temperature.

(4) Centrifuge the reaction at 5000 x g for 5 minutes and transfer the supernatant to 2.5 mL Qiagen buffer ASL. Add one InhibitEx tablet and vortex immediately for 10 minutes. Incubate the reaction for 2 minutes at room temperature and then centrifuge at 5000 x g for 5 minutes. Transfer the supernatant to a clean tube and centrifuge again at 5000 x g for 5 minutes.

(5) Transfer the supernatant to a 30 kDa Amicon Ultra-4 Centrifugal Filter, and centrifuge at 5000 x g until less than 150 µL remains on the filter.

(6) Transfer the retentate on filter to 600 µL buffer PB (modified by adding v/v 1:25 3M NaoAC pH 5 and v/v 1:1000 phenol red), and purify using a Qiagen MinElute column, washing twice with 700 µL buffer PE and eluting in 35 µL buffer EB.

6.2.2 Zymo-Spin based protocol

(1) Prepare the lysis buffer stock by mixing 50 mM EDTA (pH 8.0), 50 mM Tris-HCl (pH 8.0), 150 mM NaCl, 2.5% N-Lauroylsarcosine. Add 500 mM mercaptoethanol, 500 mM DTT and 0.5 mg/mL Proteinase K to the stock immediately before use.

(2) Add 3 mL lysis buffer to 0.5-5 gram wet sediment samples. Vortex for 2x20 seconds at 4.5 m/s on a FastPrep (15 x12 module).

(3) Rotate the reactions at 37 °C overnight and centrifuge at 4000 x g for 10 min. Transfer the supernatant to 5 mL UltraPure Phenol:Chloroform:Isoamyl Alcohol (25:24:1, v/v) and rotate for 20 minutes at room temperature.

(4) Centrifuge the reaction at 5000 x g for 5 minutes and transfer the supernatant onto a 30 kDa Amicon Ultra-4 Centrifugal Filter. Centrifuge at 5000 x g until less than 150 µL remains on the filter.

(5) Add 600 µL Prep-solution onto a Zymo-Spin III-HRC column, centrifuge for 3 minutes at 8000 x g. Transfer the retentate left on the Amicon filter into the Zymo-Spin column, and centrifuge for 3 minutes at 13,300 x g.

(6) Transfer the flow-through to 600 µL buffer PB (modified by adding v/v 1:25 3M NaoAC pH 5 and v/v 1:1000 phenol red), and purify by Qiagen MinElute column with 700 µL buffer PE washing 2 times and eluting by 35 µL buffer EB.

(7) Repeat step 5 and 6 if extract is still colored.

6.3 Library preparation and sequencing

All 554 DNA extracts (including 17 laboratory duplicates and 1 triplicate; see Table 6.3.1) and 86 negative controls were converted into Illumina sequencing libraries following the library building protocol described in ref. 67. We removed adapter-dimers and indexing primer dimers using 1:1.4 volume (library:beads solution) Agencourt AMPure beads (Beckman Coulter, CA, USA) following the manufacturer's instruction. Forty-six of these libraries (sample batches tm1, tm2, tm3, tm4 and tm5 including corresponding negative controls) were sequenced as single-indexed libraries. The remaining 596 libraries were all sequenced as dual-indexed libraries. Sequencing for 34 libraries (tm3, tm4, tm5 and tm6 samples including the corresponding controls) were performed at BGI-Shenzhen on Illumina Hiseq2500 platform (150 bp PE, single-indexed). All other sequencing was performed on Illumina HiSeq2500 (81 bp SR, single-indexed) or HiSeq4000 instruments (81 bp SR, dual-indexed) at The Danish National High-Throughput DNA Sequencing Centre in Copenhagen. All sequences were base-called using CASAVA 1.8.2 (Illumina) and demultiplexed using bcl2fastq.

Supplementary Table 6.3.1 | Extraction duplicates

Lab ID	Duplicate 1	Duplicate 2
ar1_1	tm6_12	
ar1_2	tm7_2	
ar1_3	tm7_3	
ar1_4	tm7_5	
ar1_10	tm6_5	
ar2_10	tm6_10	
ar2_12	tm7_7	tm9_10
ar2_13	tm9_11	
ar3_12	tm6_13	
ar3_28	tm6_19	
ar3_29	tm6_21	
ar3_31	tm6_22	
cr9_24	tm6_20	
tm2_4	tm3_6	
tm2_6	tm3_7	
tm4_12	tm7_1	
tm6_8	tm8_8	
tm9_2	tm6_4	

7. PhyloNorway plant genome database

The PhyloNorway plant genome database was constructed by collecting and sequencing 1,541 arctic and boreal plant specimens collected from herbaria. DNA was extracted from the selected specimens using a modified Macherey-Nagel Nucleospin 96 Plant II protocol. Two different library preparation protocols were applied depending on DNA yields. All libraries were then sequenced on an Illumina HiSeq2000 platform. Nuclear ribosomal DNA (nrDNA) and chloroplast genome (cpGenome) from each plant were assembled to evaluate the data quality and the genome coverage. Whole genome contigs for each plant were assembled and annotated as the reference database. See Supplementary Figure 7.1 for a schematic diagram illustrating the procedures. A list of plant species, herbarium information, DNA extraction, sequencing and database statistics are supplied in Supplementary Data 3. Data for three standard barcodes skimmed from this database has also been involved in ref. 68.

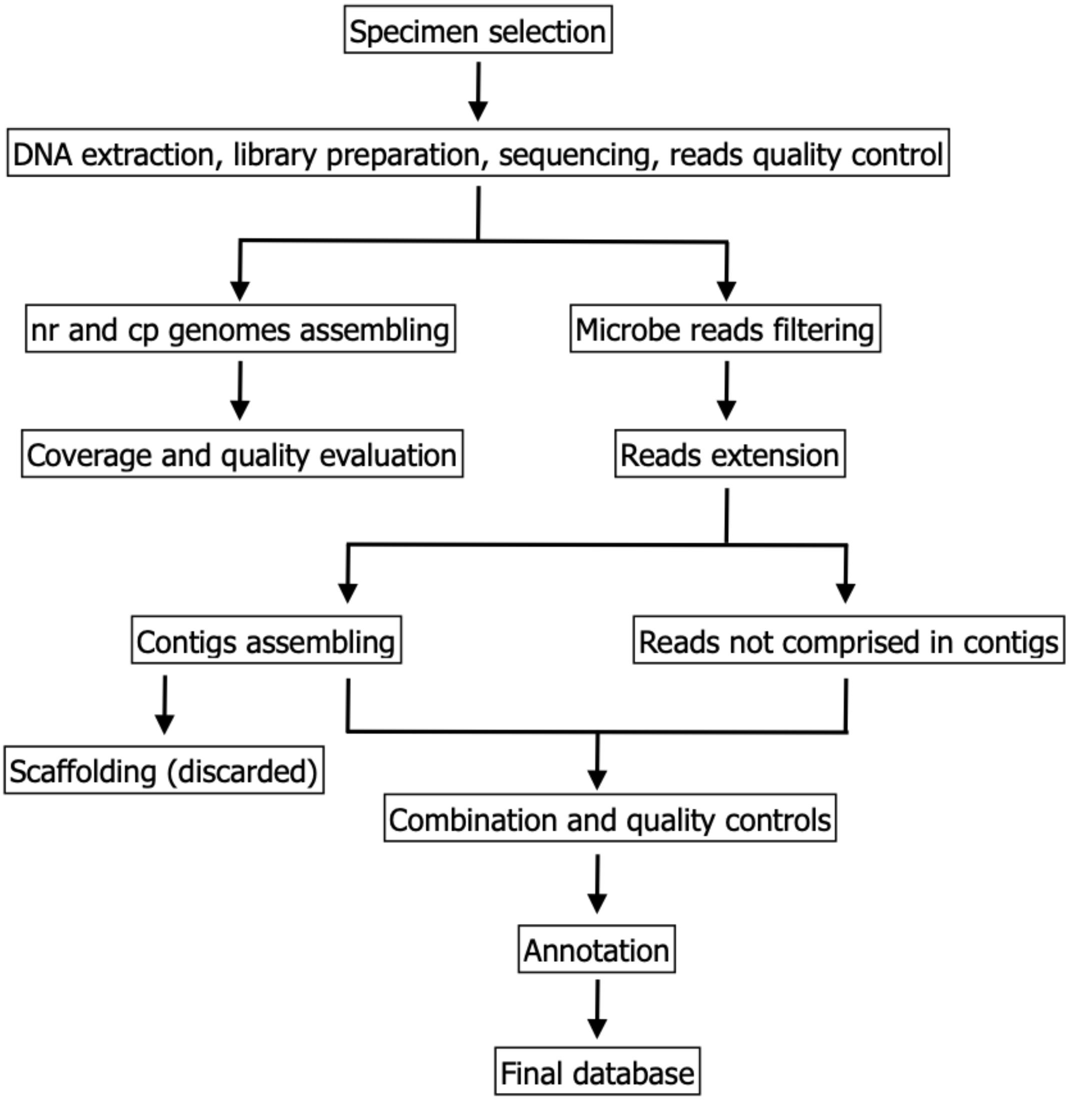

Supplementary Figure 7.1 | Schematic diagram for the procedures of the PhyloNorway database construction.

7.1 Sampling and DNA extraction

We sampled leaf material from herbarium specimens at Tromsø Museum (herbarium TROM). Specimens were selected by 4 criteria: (i) The species is native in arctic and/or boreal regions; (ii) The specimen appeared healthy, without any visible signs of fungal infection; (iii) Collection date for the specimen is as early in the growing season as possible; (iv) The sampling had proper documentation and reliable taxonomic identification. Common invasive plant species in the Arctic and sub-Arctic were also included.

DNA extractions were performed using the Macherey-Nagel Nucleospin 96 Plant II kit with the following specifications and modifications. A minimum of 20 mg dried sample was used if possible; for some specimens less material was available (see Supplementary Data 3). Two tungsten carbide beads (3 mm diameter) were added to each sample. The mix was then inserted into the TissueLyser and incubated for 4 x 1 minutes at 25 Hz. 50 mL Buffer PL1 and 1 mL RNase A were mixed as lysis buffer for 96 samples, and 500 µL lysis buffer was dispensed to each sample. The suggested brief spin before incubation was not performed. The reaction was then incubated at 65 °C overnight and centrifuged for 15 minutes at 13,200 rpm. A filtration step was performed after step 3 in the original protocol, and 400 µL cell lysate was loaded into a NucleoSpin Flash Filter Plate stacked on top of a square-well block, and then centrifuged for 2 minutes at 4600 rpm. Thereafter, 450 µL Binding Buffer PC was added to the square-well block. For step 6 in the original protocol, centrifugation was increased to 20 minutes at 6200 rpm. For the 3rd wash we made 2 modifications: (i) centrifuged for 2 minutes at 6200 rpm; (ii) the square-well block was emptied and re-centrifuged without seal for 5 minutes, and then dried in room temperature for 5 minutes. For step 7, we used 150 µL preheated Buffer PE and the flow-through was re-applied onto the filter to increase the DNA yield.

7.2 Library preparation and sequencing

The library preparation protocol applied was chosen based on the DNA extraction yields. When available, 250 ng of genomic DNA was sonicated using the E210 Covaris instrument (Covaris, Inc., USA). The NEBNext DNA Modules (New England Biolabs, MA, USA) were used for end-repair, 3`-adenylation and ligation of NextFlex DNA barcodes (Bio Scientific Corporation). After two consecutive 1x AMPure XP purifications, the ligated product was amplified with 12 PCR cycles using Kapa Hifi Hotstart NGS library Amplification kit (Kapa Biosystems, Wilmington, MA), followed by 0.6x AMPure XP purification. For extracts with a low DNA concentration, 10 - 50 ng of genomic DNA was sonicated. Fragments were end-repaired, 3'-adenylated and NEXTflex DNA barcoded adapters were added by using NEBNext Ultra II DNA Library prep kit for Illumina (New England Biolabs). After two consecutive 1x AMPure clean ups, the ligated products were PCR-amplified with the NEBNext Ultra II Q5 Master Mix included in the kit, followed by 0.8x AMPure XP purification.

All libraries were subjected to size profile analysis conducted by Agilent 2100 Bioanalyzer (Agilent Technologies, USA) and qPCR quantification (MxPro, Agilent Technologies, USA), and sequenced using 101 base-length read chemistry in a paired-end flow cell on the Illumina HiSeq2000 sequencer (Illumina, USA).

An Illumina filter was applied to remove the least reliable data from analysis. The raw data was filtered to remove any clusters with unclear base calls. Adapters, primers and nucleotides with a quality score <20 were trimmed from both ends. Sequences between the second unknown nucleotide (N) and the end of the read were also removed. After trimming, any reads shorter than 30 nucleotides were discarded. These trimming steps were achieved using the FastX package (http://hannonlab.cshl.edu/fastx_toolkit).

7.3 nrDNA and cpGenome assembly and annotation

For each sample, nrDNA and the chloroplast genome were assembled from the sequencing data using the ORG.Asm assembler (http://metabarcoding.org/asm), which applies a De Bruijn graph -based assembler specifically designed for the assembly of organelle and nrDNA from genome skimming datasets. These assemblies were carried out with default settings, unless otherwise specified.

The sequence data was indexed with the "oa index" using a variable length cut-off that retains 90% of the input sequences. The chloroplast protein coding genes and nrDNA from *Arabidopsis* were used to find the assembly seeds in the index sequence with the "oa seed" command. For both the chloroplast and nrDNA assemblies, the assembly graphs were constructed with "oa buildgraph" allowing up to 30 iterations for filling assembly gaps. The final assembled contigs were produced with "oa unfold" and "oa unfoldrdna" for the chloroplast and nrDNA assemblies, respectively. Generating a circular contig from the chloroplast assembly graph was attempted. Where a circular contig could not be obtained, the separate contigs were produced instead.

The assembled sequences were annotated with the ORG.Annot software package (http://metabarcoding.org/annot). This annotator is based on 200 chloroplast and ribosomal nuclear genomes representing arctic-alpine flora that were available at the time of software development, which resulted in annotated EMBL files. Overall assembling statistics are shown in Supplementary Table 7.3. The average contig number, assembly coverage and

length for abundant plant families are illustrated in Supplementary Figure 7.3.1, Supplementary Figure 7.3.2, and Supplementary Figure 7.3.3, respectively. A full list of summaries can be found in Supplementary Data 3.

Supplementary Table 7.3 | Summary for the nrDNA and cpGenome assembling of the PhyloNorway database.

	cpGenomes	nrDNA
Assembly	1272	1074
Complete genome	836	1063
Average coverage	171.6	519
Contigs	8868	1087
Average contigs number per assembly	6.97	1.01
Average contigs number for incomplete genomes	18.42	2.18

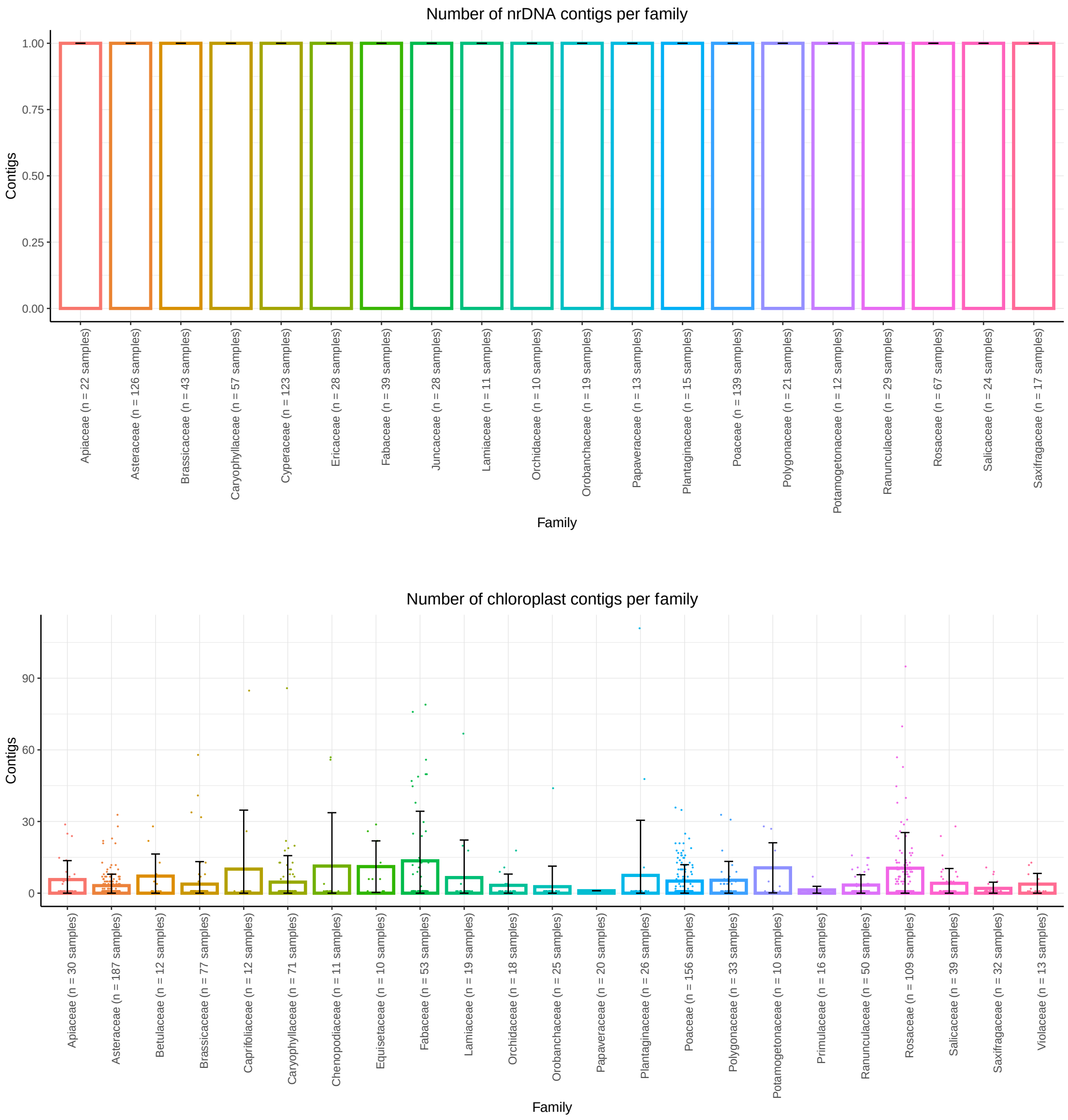

Supplementary Figure 7.3.1 | Assembly contig numbers for nrDNA and chloroplast genomes of the abundant plant families comprised in the PhyloNorway database. Numbers in the brackets after family name indicate the number of species included within that family; bars indicate the mean number of contigs per family; error bars denote the standard deviation based on the samples in each family and are cantered at the mean values. All nrDNA assemblies have only one contig, meaning that they were perfectly assembled without gaps.

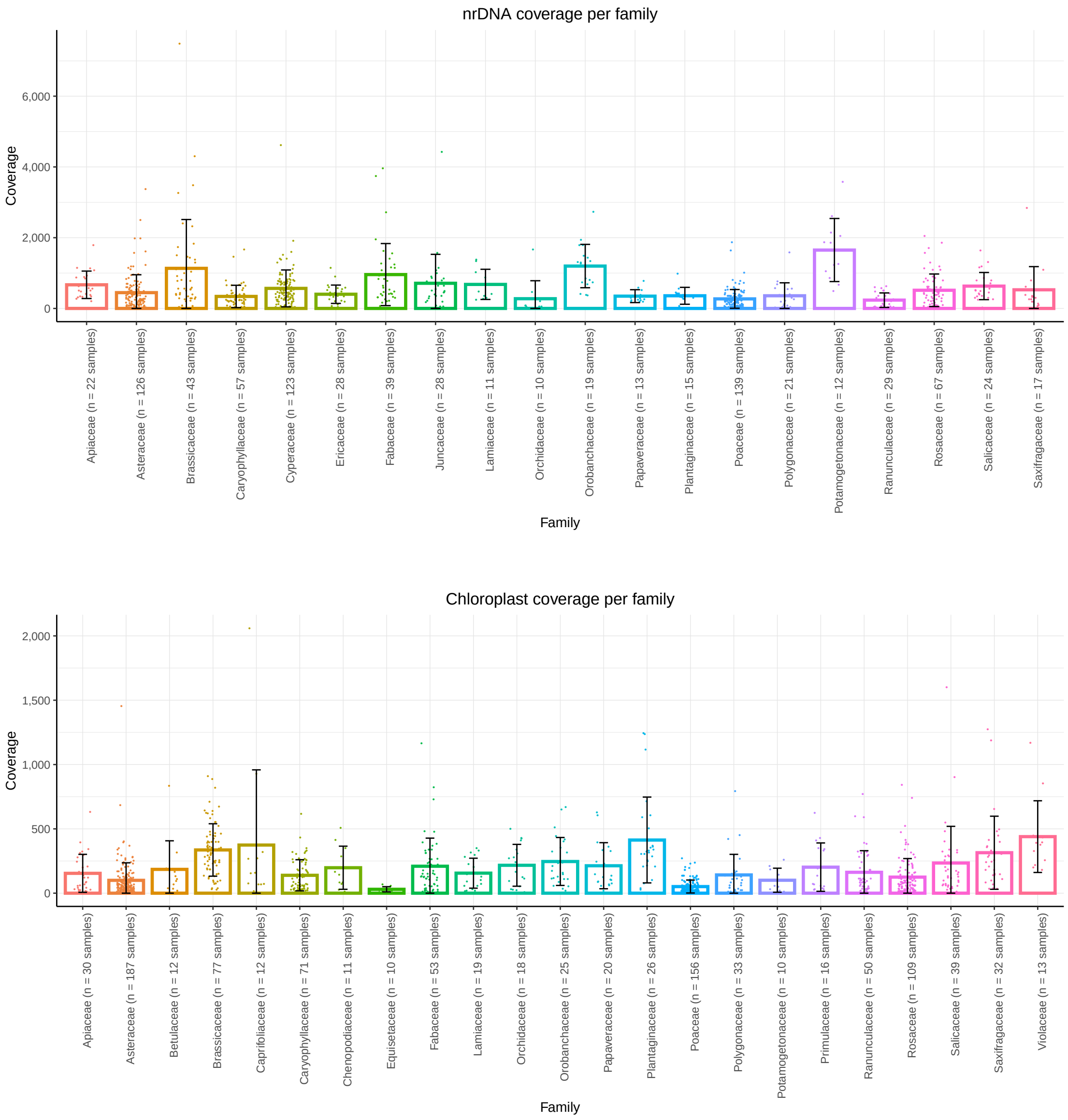

Supplementary Figure 7.3.2 | Coverage for nrDNA and chloroplast genomes of the abundant plant families comprised in the PhyloNorway database. Numbers in the brackets after family name indicate the number of species included within that family; bars indicate the mean coverage per family; error bars denote the standard deviation based on the samples in each family and are cantered at the mean values.

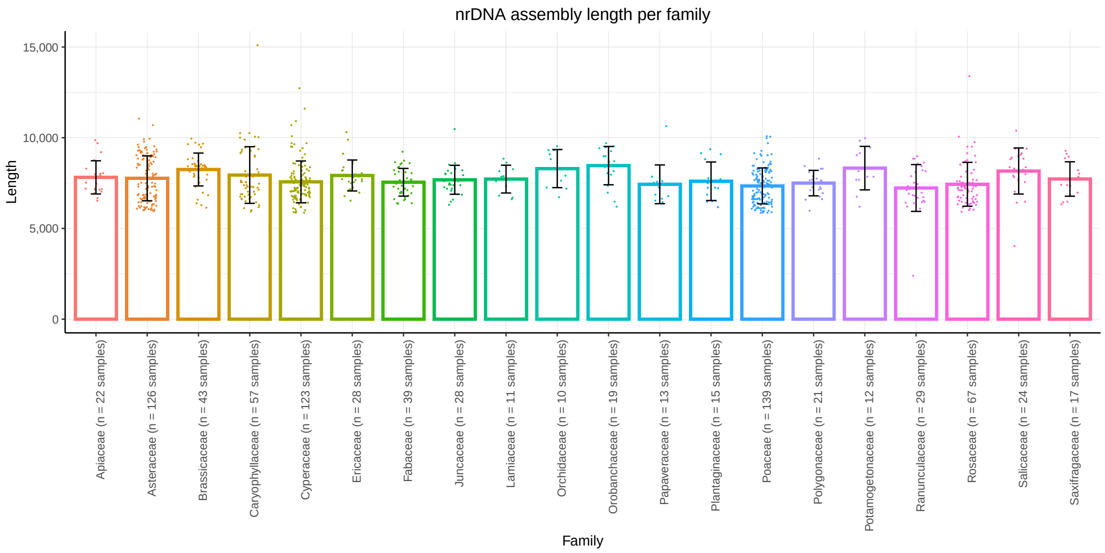

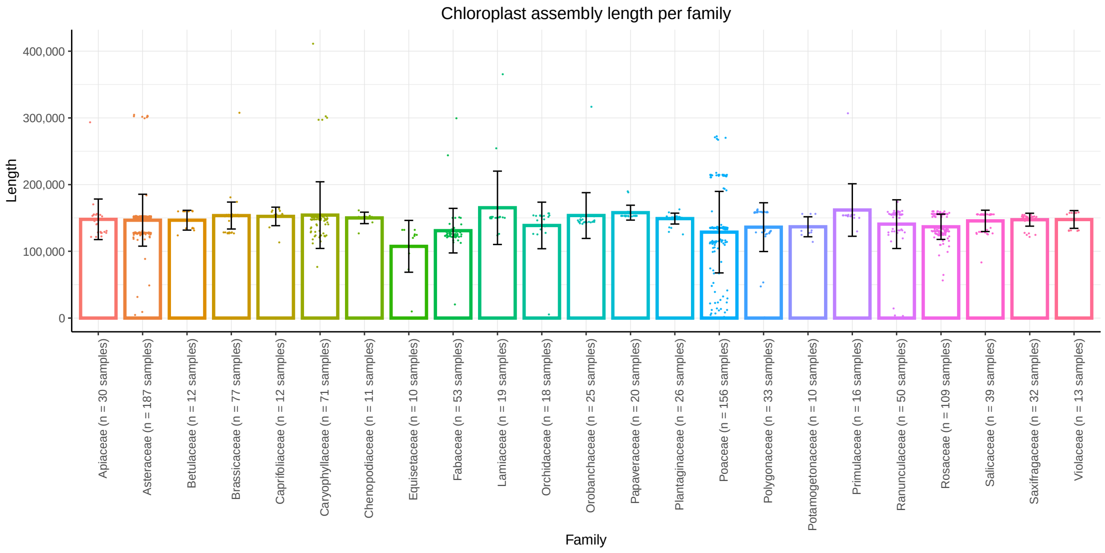

Supplementary Figure 7.3.3 | Assembly length for nrDNA and chloroplast genomes of the abundant plant families comprised in the PhyloNorway database. Numbers in the brackets after family name indicate the number of species included within that family; bars indicate the mean length per family; error bars denote the standard deviation based on the samples in each family and are cantered at the mean values.

7.4 Quality control

The chloroplast rbcL and nuclear ribomsal ITS2 barcode regions were extracted from the annotated nrDNA and cpGenomes database for quality controls. For each marker, the data was uploaded to BOLD systems (https://www.boldsystems.org) and analyzed via the Taxon ID Tree option (visualization via a simple NJ tree). Samples that were misplaced in

the tree were manually checked for misidentifications based on the uploaded herbarium material. Samples were removed if the final identification was unclear. A total of 12 plants were removed in this step. See Supplementary Data 3 for details.

7.5 Reference database construction

7.5.1 Reads filtering

We first screened the complete dataset for potential contamination of microbes. This was achieved by aligning all reads using bowtie2[69] (version 2.3.2, default settings) against a combined database including the fungi, viral, and archaea divisions of RefSeq (release 96). Non-aligning reads were directly written into new fastq files (--un, --un-conc) for subsequent analysis.

7.5.2 Contigs assembling

For each plant, all filtered reads were extended using the "tadpole" script in BBmap toolkit (https://github.com/BioInfoTools/BBMap/blob/master/sh/tadpole.sh), allowing a base extension up to 1000 nucleotides at both ends of each read (el=1000 er=1000). On average 58.58% of the reads were extended, and the mean read length was increased from 100.15 to 188.26.

Contigs were then assembled on the extended reads using megahit[70], by iterating k-mer size from 21 to 141 with a 10-mer interval (--k-min 21 --k-max 141 --k-step 10). An average of 1,423,881 contigs were generated for each plant, with mean length of 216 bases, and average maximum length of 55,988 bases. We further attempted to assemble contigs for each plant into genome scaffolds using SOAPdenovo[71], but the overall lengths were not significantly increased, and this step is therefore not included.

7.5.3 Database formation and annotation

We used bowtie2 to map all extended reads against the contigs for each plant, and extracted the reads not comprised in any contigs (--un, --un-conc) to form a subset. Reads pairs in this subset were then concatenated with AdapterRemoval (version 2.3.0)[72], requiring a minimum of 8 bases overlap (--collapse --minalignmentlength 8). For each plant, we then merged these concatenated reads and the above assembled contigs, and performed the following quality controls on the merged file. (i) Remove all records shorter than 300 bases,

via “seq” function in seqkit toolkit[73] (-m 300); (ii) Remove duplicates via “rmdup” function in seqkit toolkit by following the default settings. (iii) Remove low-complexity reads with dust values higher than 31 (cleaning up the homopolymer, dinucleotides, and trinucleotides repeats), through the “prinseq-lite” script (version 0.20.4, subrate=0.02, scanlimit=50) in PRINSEQ toolkit[74]. (iv) Remove records with similarity higher than 98%, via the “clumpify” script in BBMap (https://github.com/BioInfoTools/BBMap/blob/master/sh/clumpify.sh). This resulted in a final database containing an average of 203,047 records for each plant, with a mean length of 496 bases. See Supplementary Data 3 for contigs number and length for each plant.

To annotate the database, we replaced the ID of each record in the final database with a unique pseudo NCBI-style accession ID, and then matched the accession ID with the plant’s NCBI taxonomic ID (taxaID) by attaching extra lines to the “nucl_gb.accession2taxid.gz” file that was downloaded from NCBI Taxonomy on 10th November 2019.

8. Arctic flora and fauna checklist

To check the plant identifications, we merged the Panarctic Flora checklist[75] (PAF, http://panarcticflora.org/), an arctic bryophyte checklist (Belland R et al. unpublished), and a curated Svalbard flora list. We removed duplicates and synonyms and classified all taxa in the list to both genus and species ranks. BBTools Taxonomy server (https://taxonomy.jgi-psf.org) was then used to check if the taxon was covered by NCBI Taxonomy. We then used the function “tnrs” in R package taxize[76] to correct the ambiguous taxa names into the scientific names in NCBI Taxonomy, based on TNRS iPlant database. The corresponding NCBI taxaID was assigned to the taxa covered by NCBI Taxonomy. The final checklist contained 603 genus and 2431 species.

An arctic animal checklist was also assembled in the same format as for the plant, including the common arctic animal taxa which cover 18 genus and 47 species. The full checklist is supplied in Supplementary Data 4.

9. Bioinformatics for taxa identification

We performed the taxa identification by matching sequencing reads against a comprehensive genomic database that was annotated with taxonomic information (hereafter referred as the database in this section), following the principle of the "Holi" pipeline[33]. All reads were first quality controlled, and each read was then offered an equal chance to be aligned against all entries in the database. No limitation to specific taxonomic group, geography or environment was applied for the alignment. The lowest common ancestor (LCA) of all hits was assigned to each read that had been aligned to multiple taxa. The taxa coverage of different database compositions and their effects on taxa identification were tested. Taxa detected in laboratory controls were combined into a contamination list, and the listed taxa were subtracted from the complete dataset. The resulting plant and animal taxonomic profiles were thereafter parsed for additional authentication using a series of conservative thresholds and verifications. The relative abundances for plants were estimated based on the assigned reads number. Details are described as follows.

9.1 Reads quality controls

We first examined the presence of the DNA tracer applied during sampling and subsampling (Section 1.2 and Section 4) by screening for the forward and reverse complementary tracer sequence using zgrep on all fastq files. No samples contained the sequence of the tracer, indicating that the efforts to obtain uncontaminated samples were successful.

All raw reads were hereafter trimmed using AdapterRemoval2[72] (version 2.3.0) to remove Illumina adapters with allowing a maximum of 3 mismatches (--mm 3), and requiring a minimum base quality of 30 (--trimqualities --minquality 30) and read length ≥ 30 (--minlength 30). The fastp toolkit[77] (version 0.19.4) was then used for trimming poly G ends (-g --poly_g_min_len 5), poly X ends (-x --poly_x_min_len 5) and low-complexity reads (-y), and requiring read length ≥ 30 after trimming (-l 30). Thereafter we used the script "clumpify.sh" from the bbmap toolkits (version 33.94) to remove PCR duplicates following default settings, except changing the "scanlimit" from 5 to 10 to increase detection of inexact sequence duplicates. Read statistics after each step are supplied in Supplementary Figure 9.2.5.4 and Supplementary Data 2.

9.2 Reference database creation and taxonomic classification

The sensitivity and specificity of the taxonomic classification for a taxon is dependent on its genomic coverage in the reference database. Ideally a complete database of full genomes from all past and present organisms, that also reflects the inter-population variations of each species, would offer the optimal base for taxa identification. However, the currently available genomic references are far from complete and comprises whole and partial genomes as well as short and long genes. The genomic coverage offered by these databases varies drastically among taxa, and often skews towards scientifically interesting species and commercially important organisms. For instance, data available through the NCBI nt and RefSeq is massive for cereal plants and common animals such as rice (*Oryza sativa*), barley (*Hordeum vulgare*), cattle (*Bos taurus*), African elephant (*Loxodonta africana*), but few is available (if any) for extinct arctic megafauna such as the woolly mammoth (*Mammuthus primigenius*), woolly rhinoceros (*Coelodonta antiquitatis*) or arctic plants (for example 103 species in the PhyloNorway database had no entry in the NCBI databases).

The eDNA sequences originating from taxa with high genomic coverage are more likely to be correctly assigned. However, they can also attract reads from other taxa that share identical genes, but are not represented in the database. The likelihood that this mis-assignment occurs higher between genetically closely-related species and for sequences with short reads, as expected for fragmented sequences of ancient DNA. However, the magnitudes of this skew remain largely unknown. We therefore first set up a stepwise test to compare the resulting taxonomic classifications on different database compositions.

9.2.1 Database composition

We generated 4 databases (DB1 – DB4, Supplementary Table 9.2.2) by merging the following 4 sections stepwise.

(1) Initially, we created a fundamental database by combining two publicly available repositories from the National Center for Biotechnology Information (NCBI). The first was the NCBI nucleotide collection (nt), a database that consists of entries from all traditional divisions of GenBank, EMBL, and DDBJ, but excludes sequences longer than 100Mb.

This was downloaded on 21th November 2018 and divided into 9 equal-sized fasta files to reduce the downstream RAM (random access memory) requirement for alignment. The second was the NCBI RefSeq project (RefSeq release 90), which is a curated, non-redundant collection of reference sequences for major organisms, and was used to supplement the nt to increase coverage for eukaryotes. The RefSeq divisions were also divided into equal-sized fasta files for indexing, including vertebrate_other (9 files), vertebrate_mammalian (18 files), invertebrate (3 files), and a combination of mitochondria, plant, protozoa and plastid which were merged first, and then divided into 5 fasta files.

(2) Available arctic mammalian genomes, including: (i) 10 full genomes (Supplementary Table 9.2.1) marked as "reference genome" or "representative genome" in NCBI Genome-RefSeq, (ii) a woolly rhinoceros (*Coelodonta antiquitatis*) full genome[78] and 2 woolly mammoth (*Mammuthus primigenius*) full genomes[79]. We further added the representative human genome (GCA_000001405.28) in NCBI Genome-RefSeq to control the potential false-positive identifications caused by human contaminations.

(3) The assembled cpGenomes and nrDNA from PhyloNorway database (see Section 7.3). Taxa (mainly subspecies and variants) missing from the NCBI Taxonomy were assigned to the immediate parent nodes, i.e. subspecies/variants to species, and a few species to genus (see Supplementary Data 3 for details).

(4) The whole genome contigs of PhyloNorway database (see Section 7.5) that were merged first and then divided into 7 equal-sized fasta files.

A total of 53 fasta files were then converted into 53 bowtie databases, using "bowtie2-build" (version 2.3.2)[69] with following the default settings. Details for each database are shown in Supplementary Table 9.2.2.

Supplementary Table 9.2.1 | Mammalian genomes from NCBI Genome-RefSeq.

Common name	Scientific name	NCBI taxaID	assembly_accession
Bison	*Bison bison bison*	43346	GCA_000754665.1
Horse	*Equus caballus*	9796	GCA_002863925.1
Caribou	*Rangifer tarandus*	9870	GCA_004026565.1
Hare	*Lepus americanus*	48086	GCA_004026855.1
Red fox	*Vulpes vulpes*	9627	GCA_003160815.1
Arctic fox	*Vulpes lagopus*	494514	GCA_004023825.1
Black bear	*Ursus americanus*	9643	GCA_003344425.1
Polar bear	*Ursus maritimus*	29073	GCA_000687225.1
Ferret	*Mustela putorius furo*	9669	GCA_000215625.1
Wolverine	*Gulo gulo*	48420	GCA_900006375.2

Supplementary Table 9.2.2 | Contents, covered organism numbers, and summary for the 4 tested databases.

Database	Content	Organism	Entry (million)	Nucleotide base (billion)	Mean length
DB1	nt + RefSeq	1,472,432	63.81	993.29	15566.67
DB2	DB1 + Arctic Animal	1,472,432	68.76	1,031.31	14998.49
DB3	DB2 + PhyloNorway cpGenomes and nrDNA	1,472,489	68.77	1,031.49	14999.10
DB4	DB2 + PhyloNorway whole genome contigs	1,472,489	380.42	1,186.24	3118.21

9.2.2 Database coverage for arctic taxa

In order to quantify the genomic coverage for each taxon, we removed the genomic redundancy and duplicate genomes both within each single database (especially nt) and between the merged databases. This was achieved by a k-mer based approach.

First, all entries in each respective database were converted into all possible k-mers (k=32) with taxaID annotated. We then assigned all k-mers to different partitions based on a prefix consisting of the first six bases of each k-mer. This resulted in 4096 (4^6) different partitions, which thus allowed us to sort and merge identical k-mers into a single record. For k-mers shared by multiple taxa, taxaID of the lowest nodes on the taxonomic tree covering all these taxa were retained and annotated to the k-mer. With this we created a database listing

every unique k-mer and its corresponding taxonomic node. We then counted the number of k-mers at each node in the database, to reflect the database coverage for that given taxon.

To illustrate the improved coverage by adding arctic mammalian genomes (from DB1 to DB2), we extracted and plotted the k-mers counts of 12 selected taxa (see Supplementary Figure 9.2.2.1), including 6 arctic species and 6 commonly seen false-positives in our data. Based on DB1, the average k-mer count for the 6 false-positives is 1.81 billion, while the 6 arctic taxa on average have only 28,754 for each species except for the *Bison*, for which the RefSeq already contained a full genome. After compiling DB2, the average k-mer count for the 6 arctic taxa increased to 0.88 billion, while mean count for the 6 false-positives decreased to 1.46 billion, as some k-mers in DB1 that uniquely existed in a species' genome later also appeared in the attached arctic mammalian genomes, and thus were counted into higher taxonomic ranks (*e.g.* species to genus). Attaching arctic mammalian genomes hereby greatly alleviated the database coverage imbalance.

For a few arctic plants that also have a wide distribution in temperate regions such as *Prunus avium* and *Arabidopsis thaliana*, the NCBI databases already supply good coverage with 138.7 and 105.9 million k-mers in DB2, respectively. Attaching the plant genomes thus had limited influences for them, which is reflected by small increases of k-mer count by 2.31% and 7.27% respectively for the 2 species between DB2 to DB4. However, there are 1230 (80.28%) PhyloNorway covered arctic species having less than 10,000 k-mers in DB2, among them 122 species (7.96%) having no k-mer. By adding the PhyloNorway cpGenomes and nrDNA database (from DB2 to DB3), we increased the mean k-mer count for these 1230 species by circa 7.4 folds, but the coverage remained strikingly low compared to common plant species. We solved the issue after compiling the PhyloNorway whole genome contigs (from DB2 to DB4), which increased the average k-mer count to 66.6 million for the covered arctic plant species (See Supplementary Figure 9.2.2.2 and Supplementary Data 3).

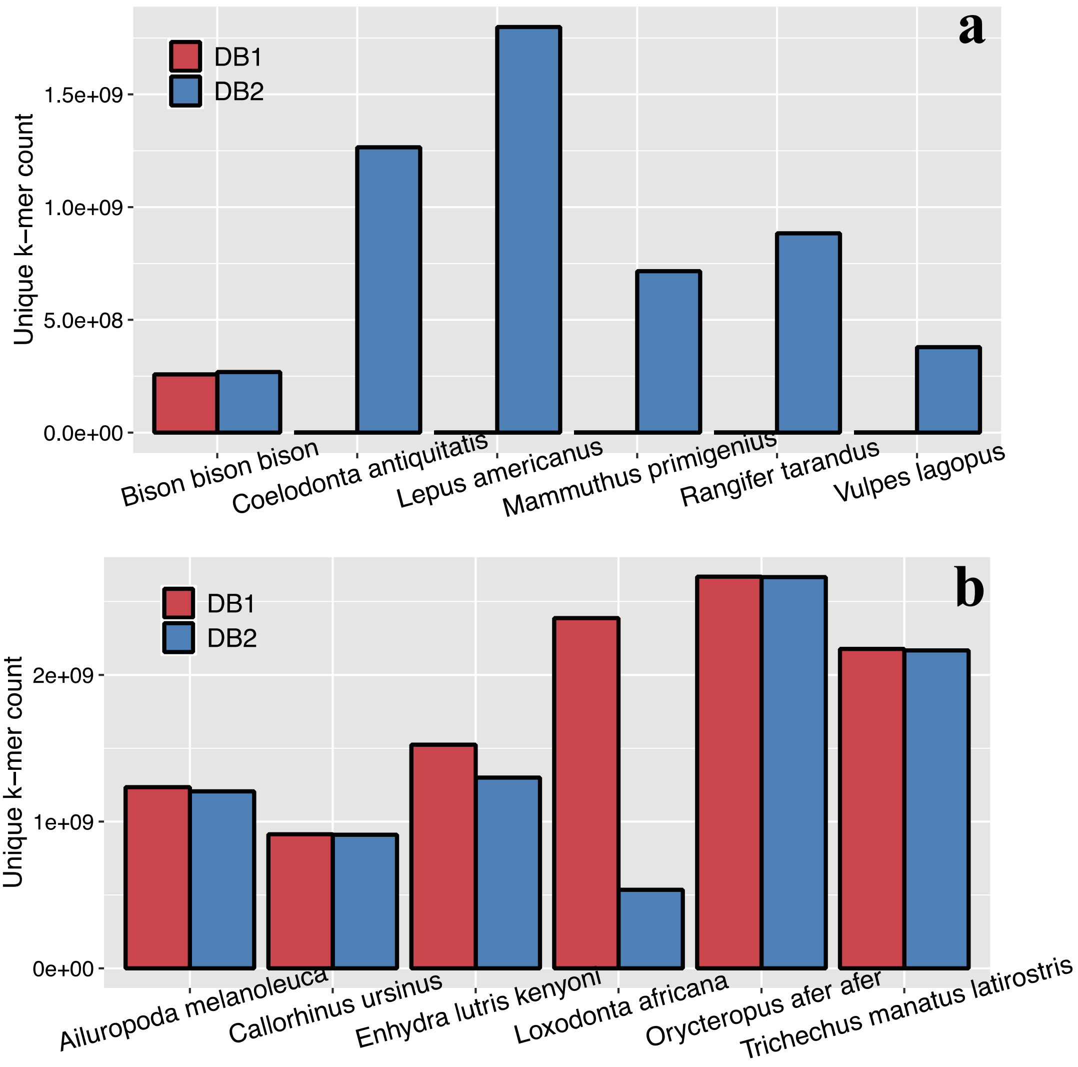

Supplementary Figure 9.2.2.1 | Database coverage for arctic faunas (a) and common false-positive animals (b), represented by k-mer counts in DB1 and DB2.

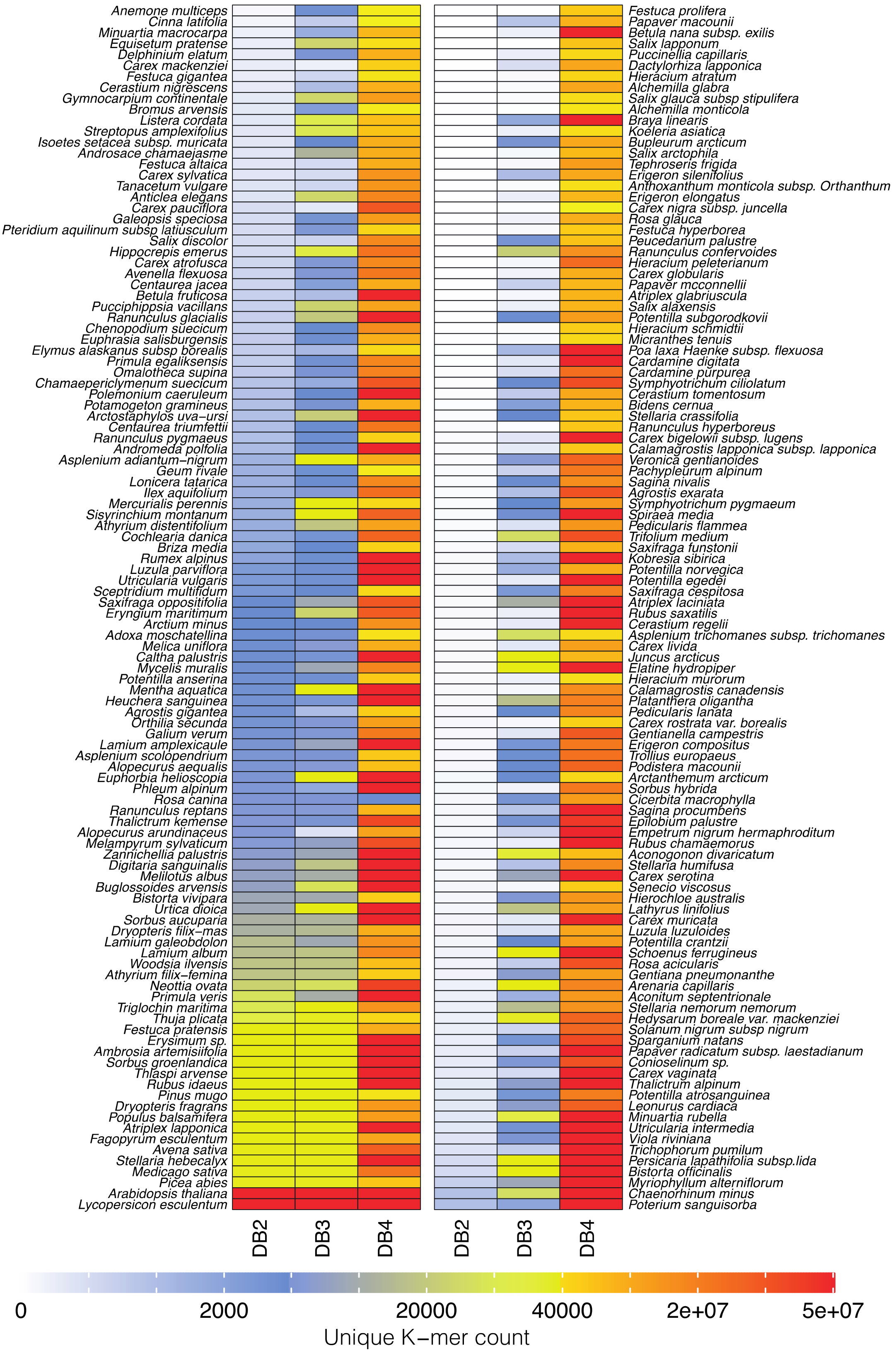

Supplementary Figure 9.2.2.2. | Database coverage for arctic plant species represented by k-mer count in DB2, DB3 and DB4.

9.2.3 Database coverage for the Asian steppe taxa

Some present-day Central Asian steppe plant taxa may extend their ranges into the Arctic during glacial times. Insufficient reference database coverage of these taxa can result in an underestimation of these taxa in our dataset. To investigate this, we compared the database coverage for the plant taxa covered by our Arctic plant checklist (Section 8), and the plant taxa of Central Asian steppe.

The compression was conducted at family level. We first downloaded all taxa within the *Plantae* Kingdom in a broader area (ranging from latitude 40º-60º N and longitude 50º - 140º E, Supplementary Figure 9.2.3.1) deposited at the GBIF Occurrence database as the Asian steppe plant list (downloaded on June 1st 2021). This list includes 19,755 taxa, most of which (92.6%) are species, subspecies, or variety. These taxa belong to 408 families. Among them only one algae family (*Characiaceae*) is not covered by NCBI taxonomy. We next applied the unique k-mer counts datasets generated in Section 9.2.2 for evaluating the reference database coverage for these families.

We found our reference database generally offers sufficient coverage for the Central Asian steppe plant taxa. As shown in Supplementary Figure 9.2.3.2, when the families are categorized based on the unique k-mer counts, there are more than 70% percent of the steppe families represented by more than 100,000 unique and exclusive k-mer. This ratio is higher than that for the arctic taxa, for which there are 66% families represented by at least 100,000 k-mer. Although the percentage of arctic taxa with very good coverage (>1 million unique k-mer) is higher than that for the steppe taxa, we found that, however, there are more steppe families across all categories (Supplementary Figure 9.2.3.3). This indicates that our reference database contains more data) for the Central Asian steppe plant taxa than for the arctic plant taxa. The potential bias of underestimating the steppe taxa in this dataset is therefore minor.

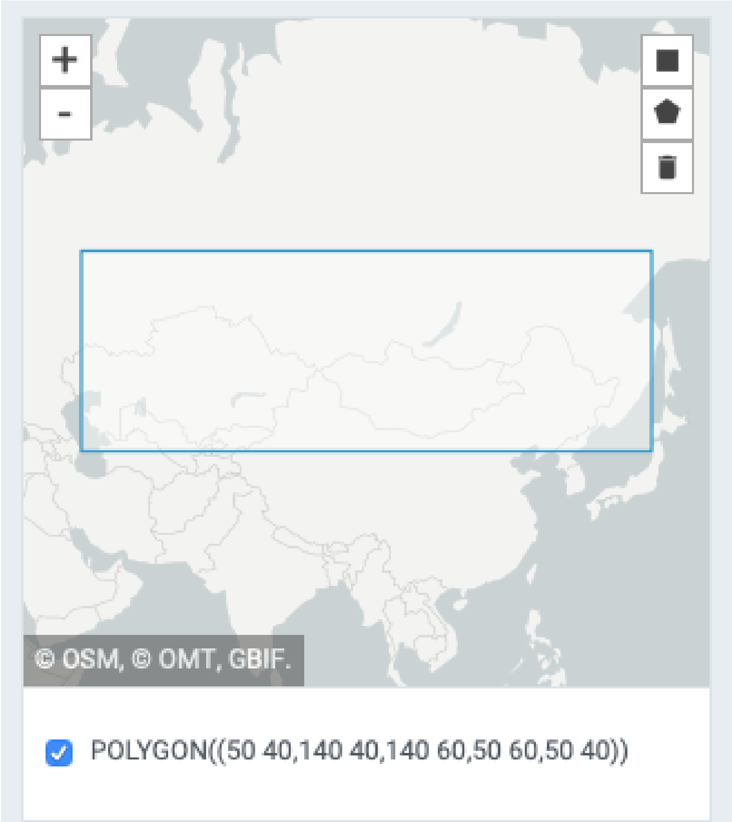

Supplementary Figure 9.2.3.1 | The area used for searching Central Asian steppe taxa.

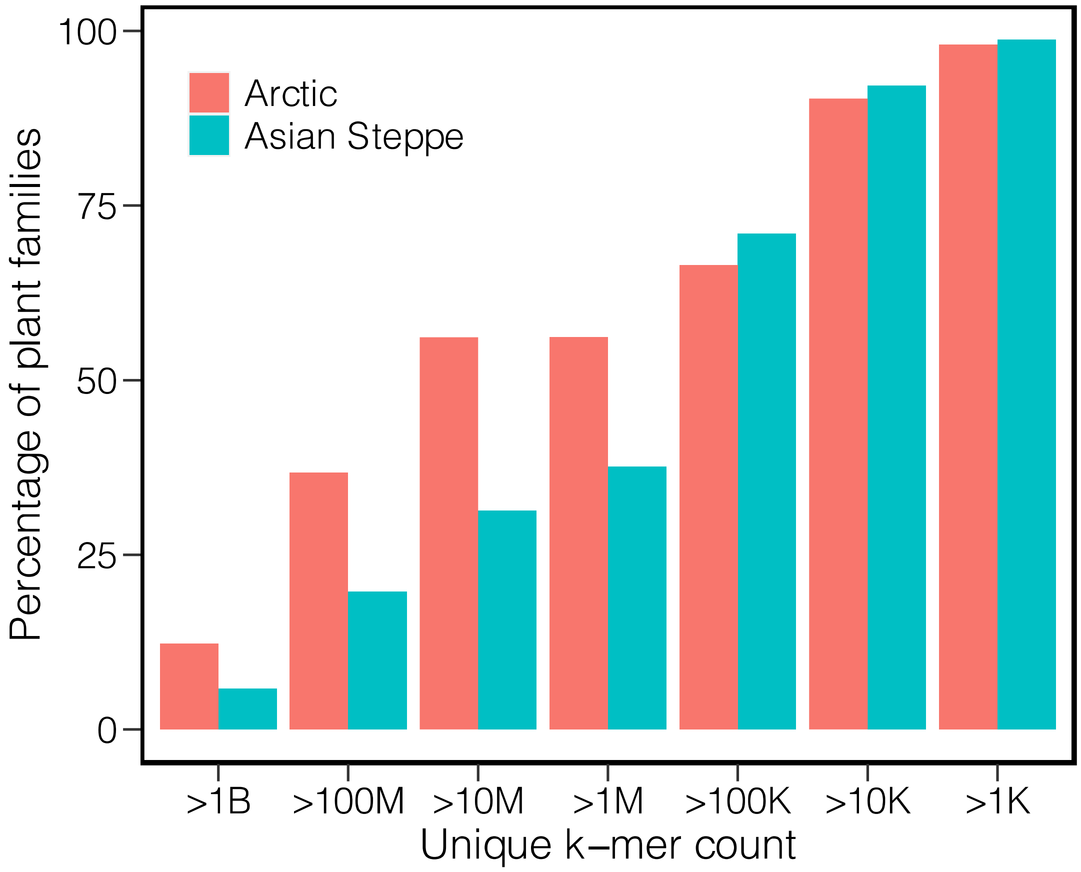

Supplementary Figure 9.2.3.2 | Percentage of the plant families represented by at least how many unique k-mer counts to the total plant family of a region. B stands for billion; M stands for million, K strands for thousand.

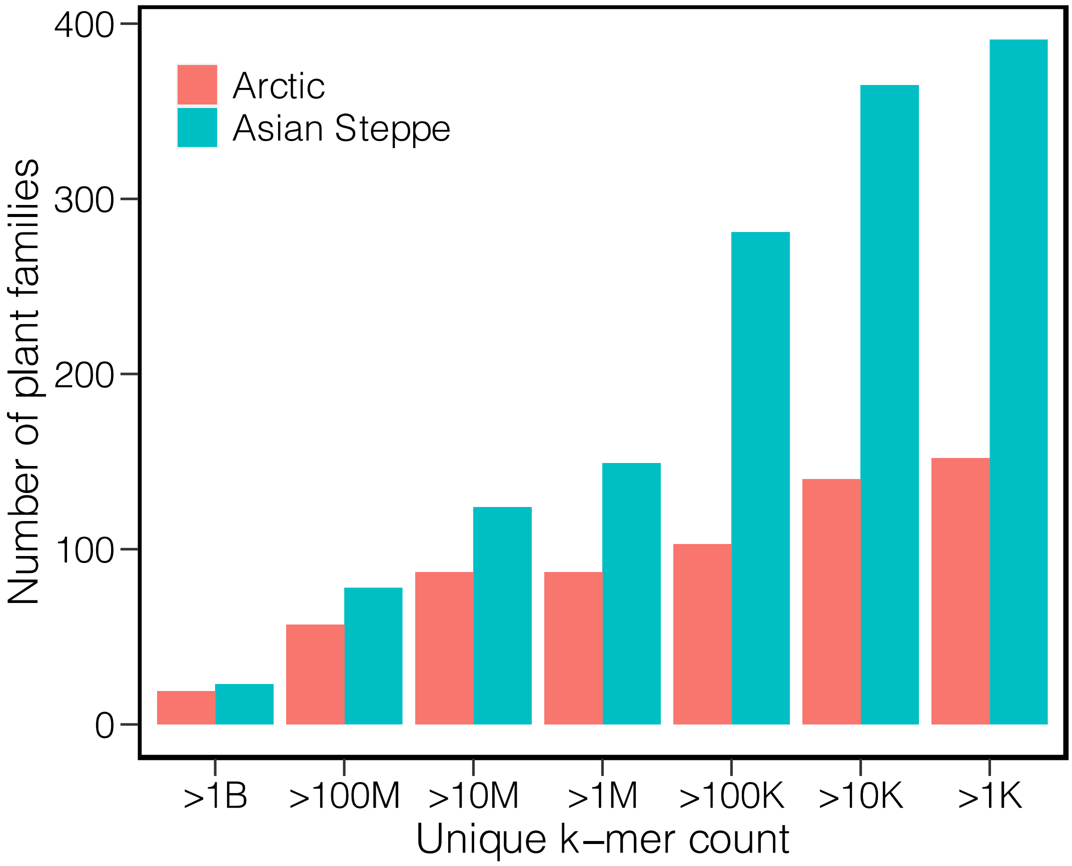

Supplementary Figure 9.2.3.3 | **Number of the plant families represented by at least how many unique k-mer counts.** B stands for billion; M stands for million, K strands for thousand.

9.2.4 Sequencing reads alignment

All quality-controlled reads were aligned against the 53 subdivided bowtie reference databases (Section 9.2.1) using bowtie2[69] (version 2.3.2), respectively. We kept 1000 hits (-k 1000) for each read against each database subdivision. This generated 53 bam files for each sample. The total number of entries in the combined database exceeded the size limit for header of bam format, and thus prevented us for merging the alignments for each sample. We therefore filtered all the bam files by parsing only alignments with 100% similarity to reference (flag AS:i:0), and kept only the reference headers (@SQ) appearing in alignments, using "samtools view"[80] and a R script. For each sample, the bam files corresponding with the compositions of DB1 to DB4 (Supplementary Table 9.1.2.) were merged respectively using "samtools merge", and then sorted by read ID using "samtools

sort”. This generated 4 final bam files for each sample that corresponded with the 4 tested databases.

We thereafter parsed alignments through a naïve lowest common ancestor algorithm using ngsLCA (https://github.com/miwipe/ngsLCA). A threshold of 5 unique reads per taxon (thr1=5) was set to remove ‘noise’ likely arising from PCR/sequencing errors, and/or mis-annotations of database[81]. This LCA analysis is based on the NCBI taxonomy (downloaded on 15th April 2019). For genomes in the database that were not covered by the NCBI taxonomy, we assigned a pseudo NCBI styled accession number to each entry, and modified the nucl_gb.accession2taxid file by attaching new lines containing the pseudo identifier and the corresponding NCBI taxaID. The identified taxa for each sample were profiled at species and genus level, by merging all nodes below the taxon.

9.2.5 Specificity and sensitivity for taxonomic classifications

After contaminants detected in laboratory controls were subtracted from the alignment results (Section 9.3), we compared the taxonomic profiles based on the 4 databases without further filtering to investigate the effects of different database composition on taxonomic classification.

On basis of DB1, we find a total 243,260 reads assigned to 654 metazoan taxa across all samples. By adding arctic mammalian genomes (from DB1 to DB2), additional 352,252 reads and 8 new taxa were increased under metazoan. We find remarkable improvements in the proportions of classified reads for mammalian identification as exemplified by three cases in Supplementary Figure 9.2.5.1. In the mammoth-elephant case, most mammoth reads were mis-assigned to African elephant (*Loxodonta africana*) on basis of DB1, and the mammoth signals were almost unseen. Based on DB2 that includes 2 mammoth genomes, sequences originating from the shared genome regions between mammoth and elephant were matched onto both species and hence classified to their common ancestor *Elephantidae*. Therefore we observe the proportion of elephant reads dramatically dropped (from 239,569 to 16,497), in which the majority were assigned to *Elephantidae* (increased from 1,129 to 224,024). In addition, sequences exclusively originating from the mammoth genome could be correctly assigned (from 145 to 88,854). Similarly, all rhinocerotic reads were mis-assigned to white rhinoceros (*Ceratotherium simum simum*) on the basis of on DB1. We were only able to identify woolly rhinoceros (*Coelodonta antiquitatis*) after

including the arctic mammalian genomes which contained a full woolly rhinoceros genome. Likewise, we find that 5904 out of 5947 (99.27%) *Leporidae* (rabbit family) sequences were assigned to the European rabbit (*Oryctolagus cuniculus*) when restricted to DB1, while the expected snow hare (*Lepus americanus*) was almost not identifiable. After switching from DB1 to DB2, majority of the previous European rabbit reads were classified to *Leporidae*, and 14,733 additional reads were identified as snow hare.

Ribosomal and chloroplast DNA is present as multicopy in plant cells, and therefore been considered as more abundant in ancient sediments and thus having a higher chance to be detected[82,83]. By attaching the PhyloNorway cpGenomes and nrDNA (DB2 to DB3), we find a 27.84% increase (7,230,378 to 9,243,667 reads) of reads assigned to plants at the family level or lower. However, by including the PhyloNorway whole genome contigs (DB4 to DB2), we find that this number increased to a total of 166,747,212 (a 23.06-fold increase, Supplementary Figure 9.2.5.2). This suggests that, although the organelle genomes are duplicated in plant cells, the larger size of the nuclear genome overpowers the multicopy of smaller organelle genomes, and hence remains the main source for plant DNA in sediments. The paleo-vegetation studies based on the widely-used metabarcoding method, which relies on amplifying and identifying the short DNA barcodes mostly distributed on chloroplast[84], exploits just a minor fraction of the plant DNA information preserved in sediments.

We see the identified plant genus and family decreased in numbers by attaching plant genomes (Supplementary Figure 9.2.5.2) while the increased species are mostly arctic plants that have no entries in DB2, fitting with the previously described expectation that improving the database coverage balance can reduce the false-positive identifications. To further confirm this, we compared the plant profiles generated on basis of DB2, DB3 and DB4 against the arctic taxa checklist assembled in Section 8 (Supplementary Figure 9.2.5.3). We see clear increases of arctic taxa proportions corresponding with improvements in the database coverage. Relative to DB1, we see improvements of 28.25% (DB2), 37.50% (DB3), and 47.35% (DB4) for identified genera covered by the checklist. The increases of read proportions assigned to arctic plants are more profound. There are 98.31% plant reads finally aligned to arctic species based on DB4, significantly better than DB2 (66.56%) and DB3 (77.07%).

In conclusion, adding the arctic mammalian genomes and PhyloNorway whole genome contigs database to the NCBI nt and RefSeq remarkably improved the database coverage and hence the specificity and sensitivity of the taxa classification for our data. The taxonomic profiles based on DB4 were therefore used for subsequent analysis. Mapped reads numbers are shown in Supplementary Figure 9.2.5.4, and for each sample in Supplementary Data 2.

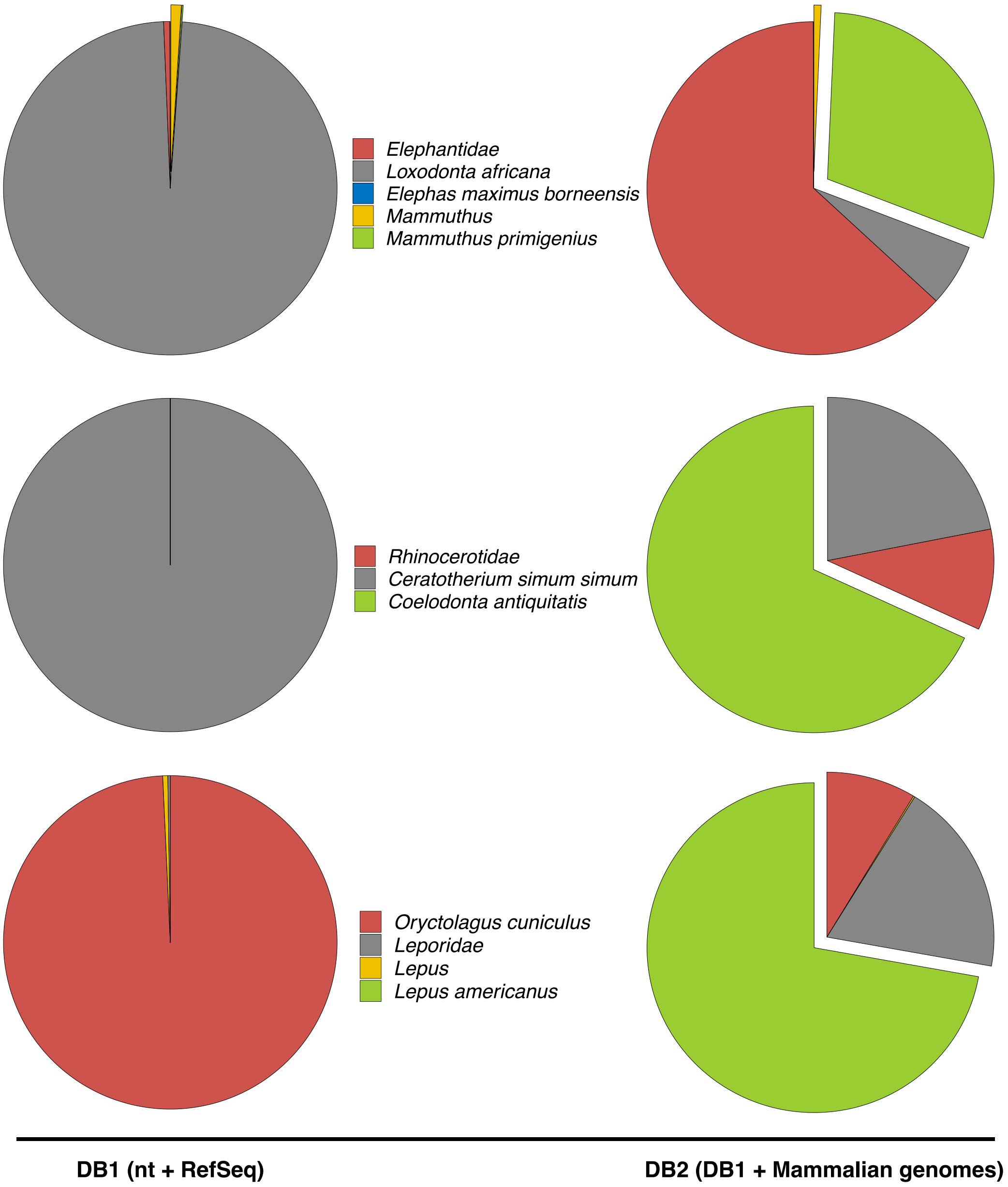

Supplementary Figure 9.2.5.1 | Three examples showing the effects of adding arctic mammalian genomes into reference database on megafauna identifications.

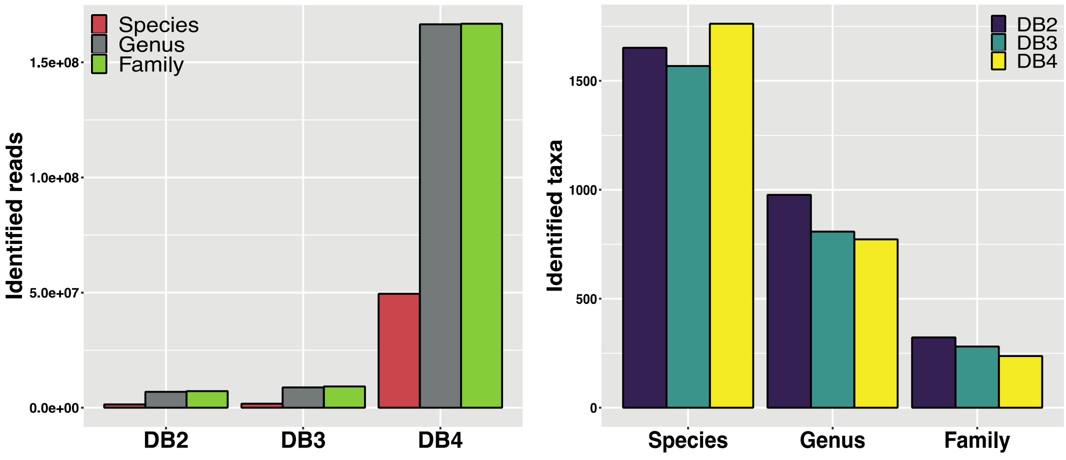

Supplementary Figure 9.2.5.2 | Numbers of identified plant reads and taxa, by adding the PhyloNorway cpGenomes and nrDNA (DB2 to DB3), and the PhyloNorway whole genome contigs (DB2 to DB4).

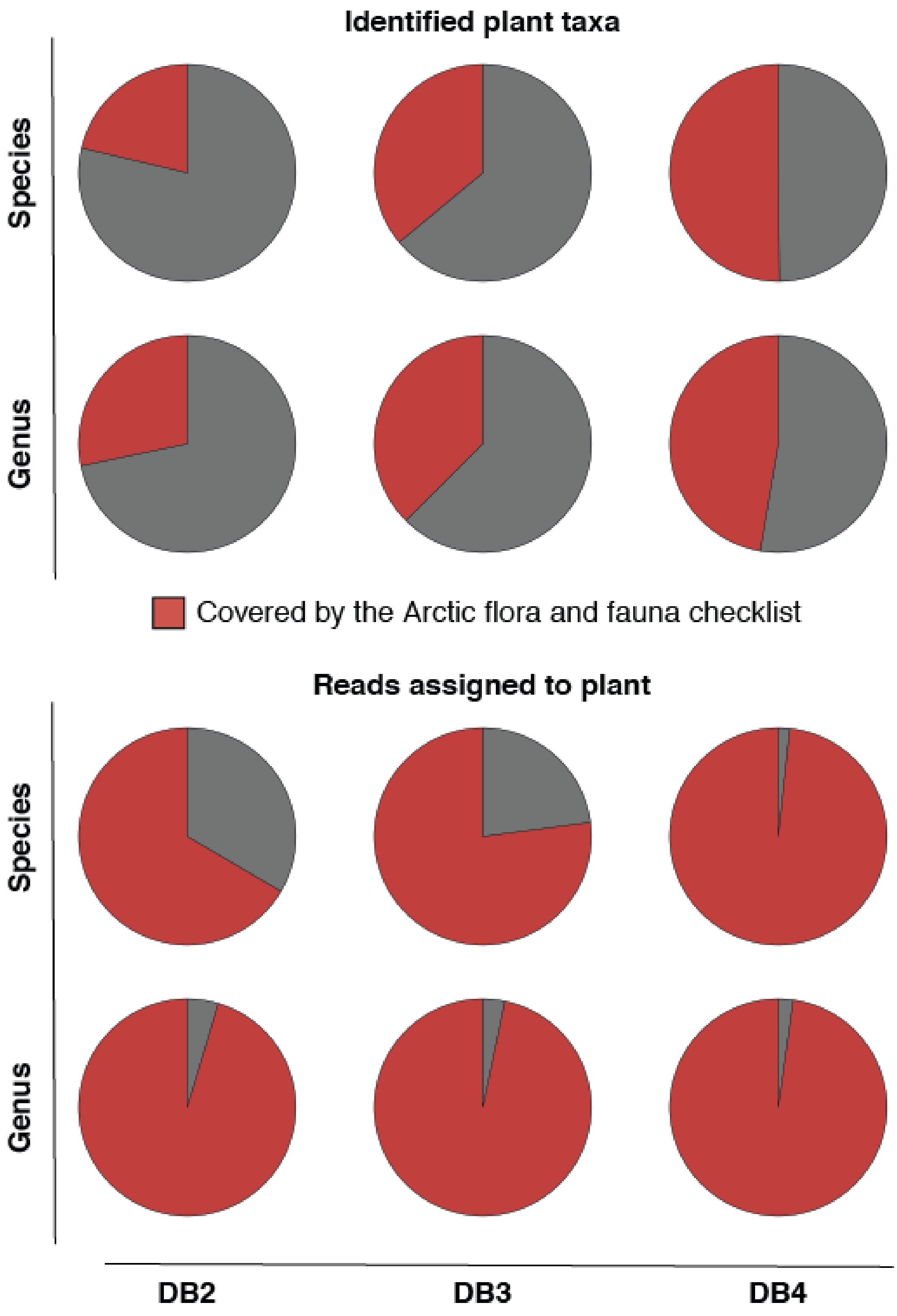

Supplementary Figure 9.2.5.3 | Proportions of the Arctic plant taxa and reads assigned to Arctic plants, by adding the PhyloNorway cpGenomes and nrDNA (DB2 to DB3), and the PhyloNorway whole genome contigs (DB2 to DB4).

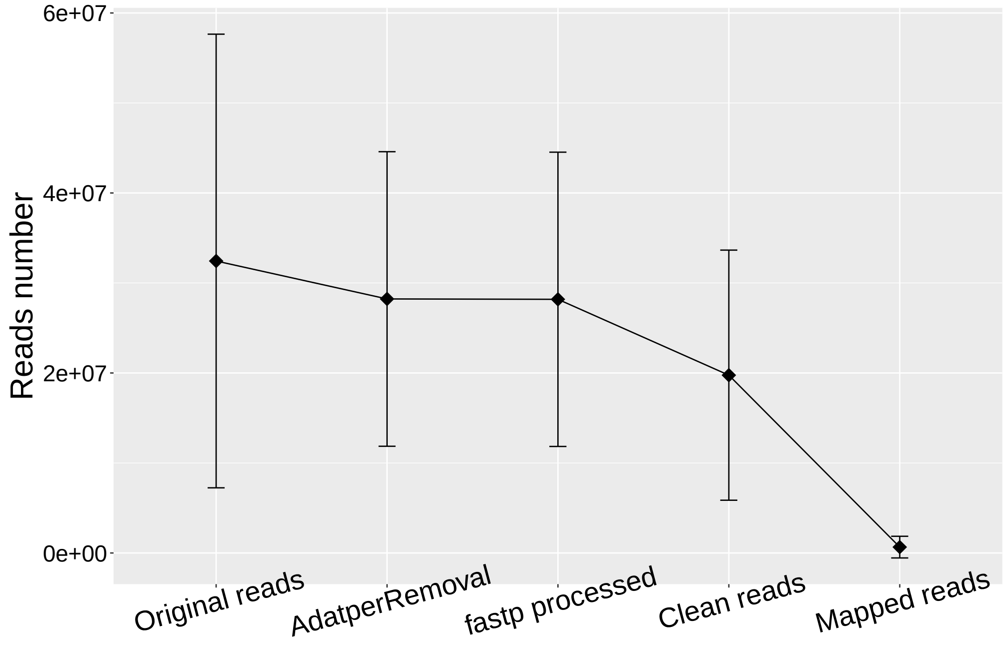

Supplementary Figure 9.2.5.4 | Average reads number per sample after each processing (n=535 samples). Error bars denote standard deviation and are cantered at the mean values. On average 65.99% of reads passed the quality controls, and 3.30% of the quality-controlled reads were successfully aligned to at least one entry in the database.

9.3 Laboratory controls

By merging all taxa identified in our 86 extraction and library controls we generated a combined list covering the potential contaminants. Given that fewer sequencing reads are contained in controls, we reduced the reads number threshold required for confirming a taxon from 5 to 2, to increase the sensitivity for contaminates detection.

It is known that index hopping in multiplex sequencing experiments is a crucial problem, particularly for single indexed libraries[85]. Although the rate of hopping is low (~0.3%), it possesses a great threat to taxonomic profiling of environmental DNA, as the method relies on identification of taxa based on a pool of DNA from mixed unknown organisms. This issue can generally be solved by noise-cancelling via setting a minimum reads number threshold to remove low-represented taxa[33], but it is amplified when the hopping occurred from sample to control that contains much fewer reads, and thus lead to false-positive

contaminants. We therefore first parsed the taxa listed in the 9 single-indexed controls to separate potential false-positives.

This was achieved by first eliminating the differences in sequencing depth across the 86 controls by normalizing the read number for each control using the “range” method in the function “Normalization” in R package IntClust. If the normalized read number for a given taxon in one of the 9 single-indexed controls was greater than 2.33% (1/86 * 2, i.e. 2 folds higher than its average reads number in all controls) of that taxon’s reads sum across all 86 negative controls, it was marked as a potential false-positive. We thereafter merged the data of all sediment samples sequenced in the same pool with each of these 9 single-indexed controls to form 9 datasets. If the proportion of reads for a potential false-positive in the control was close (≤ 0.1 proximity difference) to the taxon’s proportion of reads in the corresponding merged sediment sample dataset, reads of the taxon in the control were considered as an artifact of index hopping and thus removed from that control. In each of the 9 single-indexed controls, on average 13 out of the total 899 taxa were removed.

To ensure the taxa detected in controls were not raised from false-positive identifications owing to reads mis-assignment (see Section 9.2.5), sequencing and PCR errors, or contamination in the reference sequences[81], we applied the following 3 criteria to filter the taxa detected in each control in prior merging them.

(i) The taxon appears on the Arctic flora and fauna checklist (see Section 8). As arctic species are unlikely to contaminate reagents, consumables or laboratory facilities, there are high risk for them to be false-positive identifications given that the high reference database coverage on these arctic taxa we had supplied likely attracted short DNA fragments from other taxa.

(ii) There are at least two sediment samples processed in the same batch that have no reads identified to the taxon, indicating the taxon is not a systematic laboratory contamination for that batch.

(iii) The taxon’s coefficient of variation among all sediment samples in the same batch are greater than 0.5, indicating the taxon’s read frequencies are not correlative across samples, and thus identification of the taxon is unlikely to be a systematic contamination for that batch.

On average, in each control, 4.2 taxa conform all 3 criteria and were thus recognized as false-positives and removed. We next used a ‘greedy’ method to merge taxa from all 86 controls, by including all taxa appearing 3 or more times. This resulted in a list consisting of 1646 taxa. All listed taxa were then subtracted from the taxonomic profile of each sample.

9.4 Plant identification authentication

We applied a series of threshold-based filtering steps and verifications for authenticating the identified plants. All steps were performed on the genus and species profiles, respectively.

For each sample, we first removed the taxa represented by less than 1% of the total reads assigned to plant kingdom (*Viridiplantae*), as suggested by the test in ref. 33. In each sample, reads number of a taxon was then converted to its percentage of the plant reads sum, to eliminate difference of sequencing depth among samples. As discussed in Section 9.2, higher database coverage for a taxon attracts more reads assigning to it. Therefore, the thresholds for discriminating genuine signals and false-positives should vary based on the database coverage, which can be reflected by the unique k-mer count calculated in Section 9.2.2. We evaluated the combined plant taxonomic profile against the Arctic flora and fauna checklist assembled in Section 8, and found 153 out of the 187 identified plant genera were covered by this checklist, with an average of 0.65% reads percentage in each sample. The 34 non-arctic plant genera, however, were represented by only 0.034% reads on average. We thus standardized the k-mer counts for all identified plant genera to a range of 1% (the minimum threshold of reads percentage) to 1.97% (median of the non-arctic genera reads percentage across all samples) via the “rescale” function supplied by R package scales. For each sample, any genus with a lower reads percentage than its rescaled k-mer value was removed.

After these two filters, there were a total of 164,361,814 reads across all samples assigned to the 187 identified plant genera. Of them 163,844,375 reads (99.69%) were assigned to the 153 arctic genera. In the combined taxonomic profile, each arctic genus was represented by 1,070,878 reads on average, while each non-arctic genus was represented by only 15,218 reads. We thus summed the read percentage of each taxon across all samples, and set a minimum threshold of 5.39% (50% percentile of the reads percentage sums for the

non-arctic genus) for further removing the potential false-positives. Thereafter the genus *Zea* (maize*)* was removed as a common mis-identification[33], which appeared in 2 North America samples cr9_26 and cr9_29, and a North Atlantic sample cr9_41. The same filtering procedures were also performed on species rank.

After authentication, the final plant taxonomic profile contains 130 genera, of which 114 are covered by the arctic flora and fauna checklist. For the 16 genera not appeared in the checklist, 9 of them (*Auxenochlorella*, *Volvox*, *Chlorella*, *Scherffelia*, *Monoraphidium*, *Closterium*, *Choricystis*, *Coccomyxa*, *Nitella*) are algae, the other 7 (*Oreojuncus*, *Ficaria*, *Vigna*, *Scrophularia*, *Bromus*, *Ammi*, *Taraxacum*) are all of arctic or boreal distribution according to the GBIF (the Global Biodiversity Information Facility, https://www.gbif.org). There are 279 species in the authenticated plant taxonomic profile. Except the algae species, only 11 of them are not covered by the checklist, including *Oxytropis lapponica*, *Anemone narcissiflora*, *Myriophyllum spicatum*, *Carex magellanica*, *Potamogeton obtusifolius*, *Hordeum vulgare*, *Hedysarum boreale*, *Ammi majus*, *Nuphar lutea*, *Ficaria verna*, and *Scrophularia nodosa*. All of them are of arctic or boreal distributions based on the GBIF.

We further manually checked the plant taxonomic profile for each site against pollen/macrofossils records and DNA metabarcoding data when comparative datasets existed (Section 11). Additionally, we checked our DNA data against the biogeographical and ecological context inferred by the floristic composition and distribution investigation. This check was done for all samples from Svalbard and confirmed that the identified taxa fit with these records on genus level, but the identifications into species level are not always reliable (for example species under *Salix*). For sites where modern soil samples were sequenced, we compared the genera detected by DNA to the records of flora surveys (where available), and confirmed that the DNA-recovered plant taxa are of local distribution. For example, for site Varanger (Site ID 3, VA) where we sequenced 4 modern soil samples, 19 out of 20 identified genera are compromised in the record of a vegetation investigation published on 2012[27]. The only exception is *Hippuris*, an aquatic macrophyte habituating in shallow ponds, streams or muds. Aquatic plants can be very rapidly dispersed as soon as temperature allows in arctic and sub-arctic[86,87]. *Hippuris* therefore might occasionally appear around the sampling site during warmer summers. The same cases also occurred for Colesdalen 2009 site1 (Site ID 70, 09C1) and Endalen (Site ID 72,

ES), where we sequenced 6 and 3 modern or near-modern samples, respectively. These evidences indicate the authenticated plant taxonomic profiles are reliable on genus level.

9.5 Plant abundance estimation

In Arctic, the floristic eDNA preserved in sediment generally originated from local environment[30,51,88]. The relative abundance for plants can therefore be estimated based on the identified DNA sequence counts. We identified 5 factors that can potentially contribute the assigned reads number: (a) the taxon's abundance, (b) the degree of ancient DNA damage, (c) sequencing depth for the sample, (d) the reference database coverage for the taxon, and (e) the genomic distinctiveness for the taxon (more exclusive genes a taxon has, more of its DNA sequences can be correctly identified). In order to estimate the abundance (factor a), effects of the other 4 factors need to be eliminated.

9.5.1 Effect of DNA damage

It had been proved that in frozen samples, DNA damage could appear no different across the 50,000 years interval[14], and this is further confirmed by our data. The DNA fragmentation (illustrated by reads length distribution in Supplementary Figure 9.5.1) is not higher in older samples, and the deamination-caused nucleobase substitution rate randomly varies around a certain level in different age groups (Supplementary Figure 9.6.2.2). The effect of DNA damage (factor b) is therefore insignificant for our data.

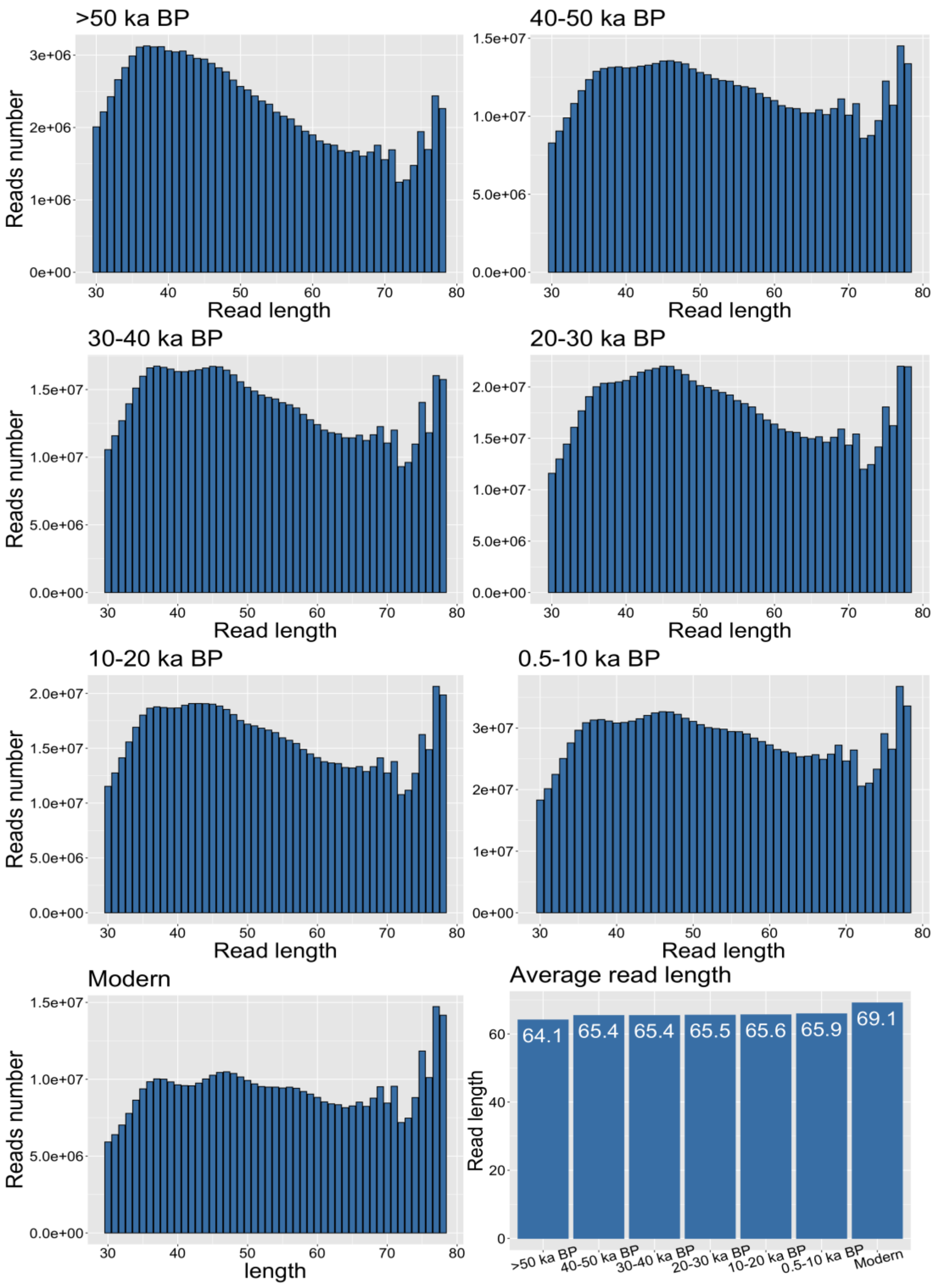

Supplementary Figure 9.5.1 | Reads length distribution, and average reads length of different age groups. DNA is highly fragmented, but only the read lengths of modern samples and samples older than 50 ka BP slightly differ from other age groups.

9.5.2 Elimination of the sequencing depth effect

We first removed samples without enough sequencing reads for validly investigating the plant community, via random rarefaction. For each sample, we required at least 100 reads assigned to plant kingdom (*Viridiplantae*) as input, and then randomly subset reads to investigate the accumulated diversities they represent. Through this we generated a rarefaction curve for each sample, and only the samples with the curve reaching a plateau asymptote (final escalating rate less than 0.001) were considered as sequenced to a sufficient depth. Out of 535 samples, 499 at genus rank and 493 at species rank passed the threshold and were included for subsequent analysis (see Supplementary Data 5).

We tested 6 different normalization methods for eliminating the effect of sequencing depth (factor c). Details for each method can be found in the normalization R script.

(i) Standardize abundances in each sample to the median of sequencing depth on plant across samples (hereafter Median).

(ii) Standardize abundances in each sample to the minimum sequencing depth on plant across samples (a common-scale, hereafter COM).

(iii) Cumulative sum scaling normalization (hereafter CSS) supplied by the R package metagenomeSeq[89].

(iv) Firstly apply the “poscounts” method supplied in R package DESeq2[90] to estimate the size factor, with a custom geometric function to deal with the frequent 0s in our data. The function “counts” in DESeq2 was then used to normalize the abundances in each sample on the basis of the sample’s size factor (hereafter Counts).

(v) Apply the first step in method iv, then normalize the abundances in each sample through the Variance Stabilizing Transformation supplied by R package DESeq2 (hereafter VST).

(vi) Apply the first step in method iv, then normalize the abundances in each sample through a log2 transformation supplied by R package DESeq2 (hereafter log2).

We found the COM method completely removed the sequencing depth differences among samples (Supplementary Table 9.5.2.1), and is the only method that well eliminated the variations introduced by sequencing depth (Supplementary Figure 9.5.2.1), yet retained

the variations of different genera among samples (Supplementary Figure 9.5.2.2). It was therefore used for the sequencing depth normalization.

Supplementary Table 9.5.2.1 | Mean sequencing depth and Standard deviation among samples after different normalization.

Method	Mean Sequencing depth	Standard deviation
Raw data	329112	644097
Median	6240	10263
COM	125	0
CSS	99	45
Counts	780282	6659057
VST	31	11
log2	91	38

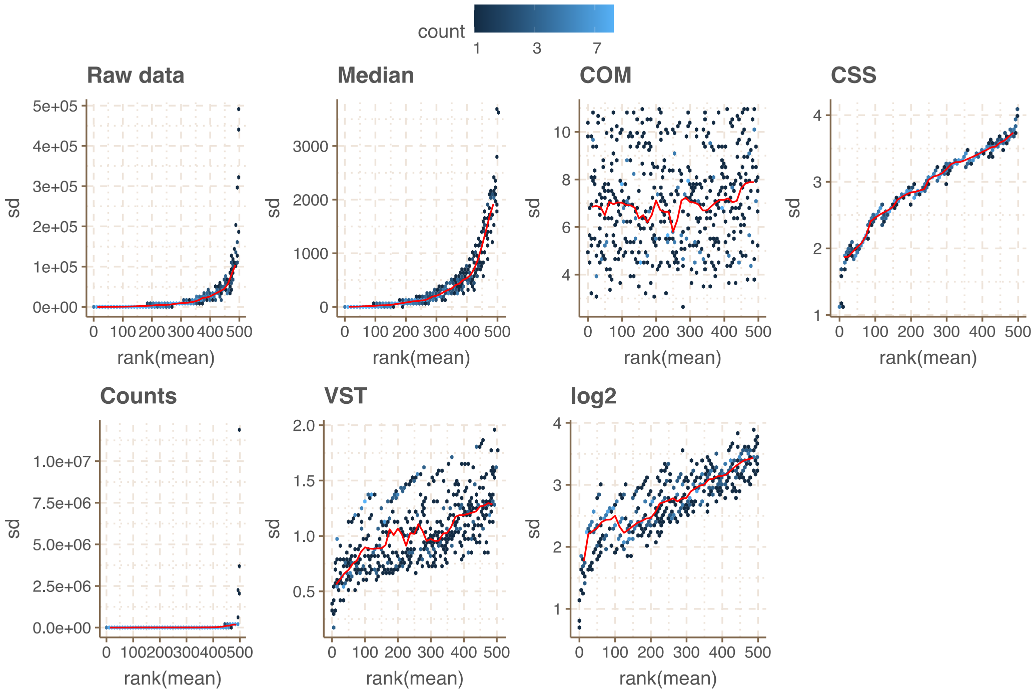

Supplementary Figure 9.5.2.1 | Standard deviations within each sample corresponded to rank of the mean sequencing depths of samples. COM method is the only one removed the dependence of standard deviation on the mean.

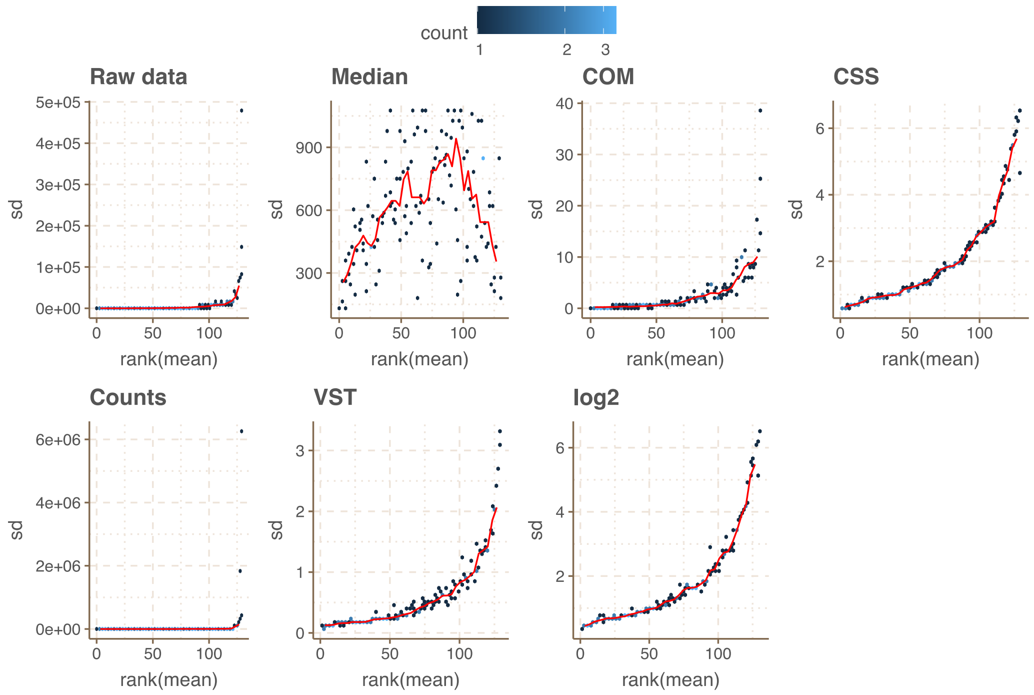

Supplementary Figure 9.5.2.2 | Standard deviation within each genus corresponded to rank of the mean abundances of genera.

9.5.3 Effect of taxa identification efficiency

The effects of reference database coverage (factor d) and the genomic distinctiveness (factor e) of a taxon can be reflected by the efficiency of the taxonomic classification for that taxon, i.e. how much proportion of the taxon's total input reads can be correctly identified through our taxa identification pipeline. We therefore mimicked 1000 mock metagenomics datasets with known reads composition for each comprised plant taxon, to examine the efficiencies of the taxa identification pipeline for different plant taxa.

Ninety-six plant full genome assemblies were downloaded from NCBI genomes ftp server (a full list shown in Supplementary Table 9.5.2.1). We generated 100,000 reads for each of the 96 plants in fastq format by randomly retrieving short sequences from its genome, using the "randomreads" script comprised in BBmap toolkit (https://github.com/BioInfoTools/BBMap/blob/master/sh/randomreads.sh) with requiring the reads length ranging from 30 to 81 and a minimum base quality of 30 for simulating our quality-controlled sequencing data (Section 9.1). Thereafter we randomly extracted 0 to 10,000 reads from the 100,000 reads for each plant, and combined the 96 subsets as a

mock metagenomics dataset. This process was repeated for 1000 times to generate 1000 mock datasets with each containing random amount of reads from the 96 plant genomes. We then parsed these 1000 datasets through our taxa identification pipeline without any filtering.

Supplementary Table 9.5.2.1 | Plant genome assemblies used for simulating the 1000 mock sequencing datasets.

Assembly accession	NCBI taxaID	Scientific name	Assembly level
GCF_000001735.4	3702	*Arabidopsis thaliana*	Chromosome
GCF_000002425.4	3218	*Physcomitrella patens*	Chromosome
GCF_000002595.1	3055	*Chlamydomonas reinhardtii*	Scaffold
GCF_000002775.4	3694	*Populus trichocarpa*	Chromosome
GCF_000003195.3	4558	*Sorghum bicolor*	Chromosome
GCF_000003745.3	29760	*Vitis vinifera*	Chromosome
GCF_000004075.3	3659	*Cucumis sativus*	Chromosome
GCF_000004255.2	81972	*Arabidopsis lyrata subsp. lyrata*	Scaffold
GCF_000004515.5	3847	*Glycine max*	Chromosome
GCF_000005005.2	4577	*Zea mays*	Chromosome
GCF_000005505.3	15368	*Brachypodium distachyon*	Chromosome
GCF_000143415.4	88036	*Selaginella moellendorffii*	Scaffold
GCF_000143455.1	3068	*Volvox carteri f. nagariensis*	Scaffold
GCF_000150535.2	3649	*Carica papaya*	Scaffold
GCF_000151685.1	3988	*Ricinus communis*	Scaffold
GCF_000184155.1	101020	*Fragaria vesca subsp. vesca*	Chromosome
GCF_000188115.4	4081	*Solanum lycopersicum*	Chromosome
GCF_000208745.1	3641	*Theobroma cacao*	Chromosome
GCF_000219495.3	3880	*Medicago truncatula*	Chromosome
GCF_000226075.1	4113	*Solanum tuberosum*	Scaffold
GCF_000231095.1	4533	*Oryza brachyantha*	Chromosome
GCF_000263155.2	4555	*Setaria italica*	Chromosome
GCF_000309985.1	3711	*Brassica rapa*	Chromosome
GCF_000313045.1	3656	*Cucumis melo*	Scaffold
GCF_000313855.2	214687	*Musa acuminata subsp. malaccensis*	Chromosome
GCF_000315295.1	225117	*Pyrus x bretschneideri*	Scaffold
GCF_000317415.1	2711	*Citrus sinensis*	Chromosome
GCF_000327365.1	29730	*Gossypium raimondii*	Chromosome
GCF_000331145.1	3827	*Cicer arietinum*	Chromosome
GCF_000340665.1	3821	*Cajanus cajan*	Chromosome
GCF_000346465.2	3760	*Prunus persica*	Chromosome
GCF_000346735.1	102107	*Prunus mume*	Chromosome
GCF_000365185.1	4432	*Nelumbo nucifera*	Scaffold

GCF_000375325.1	81985	*Capsella rubella*	Scaffold
GCF_000390325.2	4098	*Nicotiana tomentosiformis*	Scaffold
GCF_000393655.1	4096	*Nicotiana sylvestris*	Scaffold
GCF_000413155.1	42345	*Phoenix dactylifera*	Scaffold
GCF_000414095.1	981085	*Morus notabilis*	Scaffold
GCF_000442705.1	51953	*Elaeis guineensis*	Chromosome
GCF_000463585.1	28532	*Tarenaya hassleriana*	Scaffold
GCF_000471905.2	13333	*Amborella trichopoda*	Scaffold
GCF_000478725.1	72664	*Eutrema salsugineum*	Scaffold
GCF_000493195.1	85681	*Citrus clementina*	Scaffold
GCF_000495115.1	75702	*Populus euphratica*	Scaffold
GCF_000499845.1	3885	*Phaseolus vulgaris*	Chromosome
GCF_000504015.1	4155	*Erythranthe guttata*	Scaffold
GCF_000511025.2	3555	*Beta vulgaris subsp. vulgaris*	Chromosome
GCF_000512975.1	4182	*Sesamum indicum*	Chromosome
GCF_000611645.1	145388	*Monoraphidium neglectum*	Scaffold
GCF_000612285.1	29729	*Gossypium arboreum*	Chromosome
GCF_000612305.1	71139	*Eucalyptus grandis*	Scaffold
GCF_000633955.1	90675	*Camelina sativa*	Chromosome
GCF_000686985.2	3708	*Brassica napus*	Chromosome
GCF_000695525.1	109376	*Brassica oleracea var. oleracea*	Chromosome
GCF_000696525.1	180498	*Jatropha curcas*	Scaffold
GCF_000710875.1	4072	*Capsicum annuum*	Chromosome
GCF_000715135.1	4097	*Nicotiana tabacum*	Scaffold
GCF_000741045.1	3916	*Vigna radiata var. radiata*	Chromosome
GCF_000801105.1	3726	*Raphanus sativus*	Scaffold
GCF_000817695.2	130453	*Arachis duranensis*	Chromosome
GCF_000826755.1	326968	*Ziziphus jujuba*	Chromosome
GCF_000987745.1	3635	*Gossypium hirsutum*	Chromosome
GCF_001190045.1	3914	*Vigna angularis*	Chromosome
GCF_001263595.1	78828	*Phalaenopsis equestris*	Scaffold
GCF_001406875.1	28526	*Solanum pennellii*	Chromosome
GCF_001411555.1	51240	*Juglans regia*	Scaffold
GCF_001433935.1	39947	*Oryza sativa Japonica Group*	Chromosome
GCF_001531365.1	59895	*Cynara cardunculus var. scolymus*	Chromosome
GCF_001540865.1	4615	*Ananas comosus*	Chromosome
GCF_001605985.2	906689	*Dendrobium catenatum*	Scaffold
GCF_001625215.1	79200	*Daucus carota subsp. sativus*	Chromosome
GCF_001654055.1	3981	*Hevea brasiliensis*	Scaffold
GCF_001659605.1	3983	*Manihot esculenta*	Chromosome
GCF_001683475.1	63459	*Chenopodium quinoa*	Scaffold
GCF_001865875.1	3871	*Lupinus angustifolius*	Chromosome
GCF_001876935.1	4686	*Asparagus officinalis*	Chromosome
GCF_001879085.1	49451	*Nicotiana attenuata*	Chromosome
GCF_001879475.1	35883	*Ipomoea nil*	Scaffold

GCF_001957025.1	169297	*Aegilops tauschii subsp. tauschii*	Contig
GCF_001995035.1	3673	*Momordica charantia*	Scaffold
GCF_002007265.1	3562	*Spinacia oleracea*	Scaffold
GCF_002127325.1	4232	*Helianthus annuus*	Chromosome
GCF_002168275.1	108875	*Herrania umbratica*	Scaffold
GCF_002207925.1	42229	*Prunus avium*	Scaffold
GCF_002211085.1	206008	*Panicum hallii*	Chromosome
GCF_002303985.1	66656	*Durio zibethinus*	Scaffold
GCF_002738345.1	3661	*Cucurbita maxima*	Scaffold
GCF_002738365.1	3662	*Cucurbita moschata*	Scaffold
GCF_002742605.1	158386	*Olea europaea var. sylvestris*	Chromosome
GCF_002806865.1	3664	*Cucurbita pepo subsp. pepo*	Chromosome
GCF_002870075.1	4236	*Lactuca sativa*	Scaffold
GCF_002906115.1	58331	*Quercus suber*	Scaffold
GCF_002994745.1	74649	*Rosa chinensis*	Chromosome
GCF_003086295.2	3818	*Arachis hypogaea*	Chromosome
GCF_003573695.1	3469	*Papaver somniferum*	Chromosome
GCF_003576645.1	35885	*Ipomoea triloba*	Chromosome

We found that the number of identified reads for a taxon is linear correlated to its input reads number, but the correlation coefficient (ρ) varies for different plants (Supplementary Figure 9.5.2.1 and Supplementary Table 9.5.2.2). This indicates that the reads assigned to a taxon is directly determined by the taxon's reads existed in the input sequencing data, but the identification efficiency differs among taxa. To examine how the database coverage and the genomic distinctiveness of a taxon affecting its identification efficiency, we investigated the correlation between these two factors to the ρ value for each taxon. The unique k-mer counts in reference database (Section 9.2.2) were used to reflect the database coverage. The ratio of unique k-mer count for a taxon to its total k-mer count, i.e. proportion of the exclusive sequences for a taxon to all its available sequences in the database, was used to reflect its genomic distinctiveness. As shown in the Supplementary Figure 9.5.2.2, we found that the database coverage does not affect the identification efficiency, likely due to the target taxa in the mock datasets are generally well represented by the reference database (the minimum unique k-mer count for the 96 investigated plants is 66.77 million), while the identification efficiency is mainly determined by the genomic distinctiveness, which is linear correlated to ρ.

Given the reference database well covering the identified plant genera in our dataset (mean unique k-mer count for the 130 identified genera is 273.47 million), we therefore calculated the value of ρ for each plant genus as its taxonomic classification efficiency, and thereby

estimated the relative abundance by rescaling the normalized reads number on its ρ value through the simulated equation (Supplementary Figure 9.5.2.2). The final plant abundance table is supplied in Supplementary Data 5.

The abundance estimation was also performed on species level, but given the uncertainty of the identification into the species level for some taxa and the database coverage imbalance at species level, only the genus abundance was used for subsequent analysis.

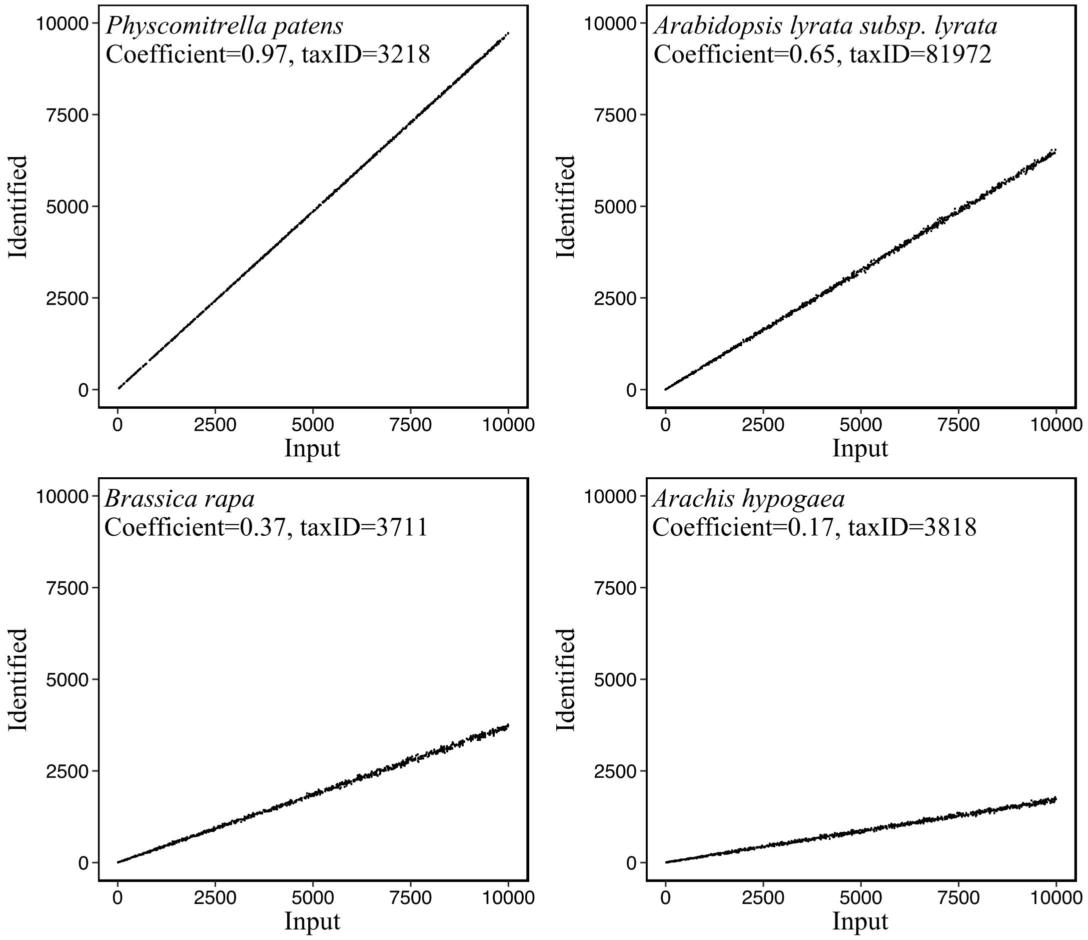

Supplementary Figure 9.5.2.1 | Four examples illustrating the correlation between the input and identified reads number of the 1000 mock datasets.

Supplementary Table 9.5.2.2 | The coefficient of the linear regression between input and identified reads number, the unique k-mer count in the reference database, and the estimated genomic distinctiveness for tested plant taxa.

NCBI taxaID	Linear correlation coefficient	R^2	Unique k-mer count	Genomic distinctiveness
3702	0.89	0.9999	113,590,034	0.84
3218	0.97	1.0000	357,833,475	1.00
3055	0.96	1.0000	94,828,109	0.99
3694	0.72	0.9998	228,487,143	0.66
4558	0.95	1.0000	382,846,616	0.97
29760	0.97	1.0000	452,043,351	0.99
3659	0.81	0.9999	164,941,685	0.90
81972	0.65	0.9997	80,155,388	0.56
3847	0.96	1.0000	656,674,966	0.98
4577	0.85	0.9999	825,439,027	0.98
15368	0.92	1.0000	219,070,596	0.92
88036	0.97	1.0000	129,613,096	1.00
3068	0.95	1.0000	106,305,053	0.99
3649	0.97	1.0000	234,525,601	0.99
3988	0.97	1.0000	254,640,781	0.99
101020	0.76	0.9998	120,108,935	0.68
4081	0.82	0.9999	500,937,061	0.76
3641	0.92	1.0000	223,726,941	0.88
3880	0.95	1.0000	305,102,896	0.96
4113	0.93	1.0000	506,571,043	0.92
4533	0.96	1.0000	220,873,319	0.97
4555	0.95	1.0000	315,474,196	0.97
3711	0.37	0.9991	92,361,736	0.33
3656	0.94	1.0000	275,734,294	0.93
214687	0.95	1.0000	320,637,064	0.99
225117	0.81	0.9999	245,232,223	0.74
2711	0.42	0.9993	79,988,375	0.34
29730	0.62	0.9997	302,347,581	0.50
3827	0.96	1.0000	336,335,120	0.98
3821	0.96	1.0000	407,086,709	0.97
3760	0.78	0.9998	116,769,729	0.65
102107	0.78	0.9998	119,929,528	0.66
4432	0.97	1.0000	606,917,920	1.00
81985	0.76	0.9998	80,377,552	0.67
4098	0.37	0.9990	327,988,746	0.29
4096	0.23	0.9980	224,541,342	0.17
42345	0.96	1.0000	404,770,809	0.97

981085	0.97	1.0000	236,005,810	0.99
51953	0.97	1.0000	851,657,205	0.99
28532	0.96	1.0000	159,342,401	0.99
13333	0.97	1.0000	588,468,541	1.00
72664	0.94	1.0000	164,262,983	0.92
85681	0.42	0.9992	81,611,314	0.35
75702	0.85	0.9999	286,898,424	0.81
3885	0.96	1.0000	355,532,675	0.96
4155	0.96	1.0000	199,432,805	0.99
3555	0.96	1.0000	404,410,197	0.99
4182	0.97	1.0000	234,927,328	0.99
145388	0.97	1.0000	66,776,475	1.00
29729	0.55	0.9996	415,820,446	0.46
71139	0.97	1.0000	493,964,709	1.00
90675	0.94	1.0000	383,451,803	0.94
3708	0.34	0.9989	163,746,751	0.31
109376	0.40	0.9992	113,384,636	0.33
180498	0.97	1.0000	216,274,727	0.99
4072	0.97	1.0000	2,233,660,182	0.99
4097	0.28	0.9987	518,208,926	0.22
3916	0.90	0.9999	309,978,494	0.86
3726	0.94	1.0000	268,076,336	0.92
130453	0.30	0.9987	151,965,195	0.26
326968	0.97	1.0000	282,803,016	0.99
3635	0.53	0.9995	624,272,694	0.47
3914	0.90	0.9999	317,510,243	0.86
78828	0.97	1.0000	779,590,578	1.00
28526	0.81	0.9999	527,699,161	0.76
51240	0.97	1.0000	475,566,942	0.99
39947	0.30	0.9986	93,298,780	0.31
59895	0.96	1.0000	477,512,631	0.98
4615	0.97	1.0000	291,575,373	1.00
906689	0.00	0.7801	818,906,866	1.00
79200	0.81	0.9999	258,371,865	0.91
3981	0.96	1.0000	657,502,786	0.99
3983	0.97	1.0000	348,266,799	0.98
63459	0.90	0.9999	724,919,914	0.96
3871	0.96	1.0000	477,321,804	0.98
4686	0.97	1.0000	686,459,661	1.00
49451	0.85	0.9999	1,027,544,599	0.84
35883	0.92	1.0000	418,211,218	0.92
169297	0.70	0.9998	360,133,025	0.23
3673	0.97	1.0000	230,336,161	0.99
3562	0.71	0.9998	391,047,456	0.93
4232	0.96	1.0000	1,216,205,494	0.99

108875	0.91	0.9999	173,263,302	0.85
42229	0.82	0.9999	141,959,773	0.71
206008	0.96	1.0000	320,957,503	0.97
66656	0.97	1.0000	554,667,158	0.99
3661	0.78	0.9998	134,196,332	0.66
3662	0.71	0.9998	128,956,024	0.60
158386	0.96	1.0000	739,178,523	0.99
3664	0.70	0.9998	119,503,451	0.58
4236	0.97	1.0000	1,300,416,142	0.99
58331	0.97	1.0000	665,656,388	0.99
74649	0.65	0.9997	219,974,813	0.63
3818	0.17	0.9973	244,059,130	0.19
3469	0.91	0.9999	1,397,182,454	0.99
35885	0.75	0.9998	187,142,615	0.67

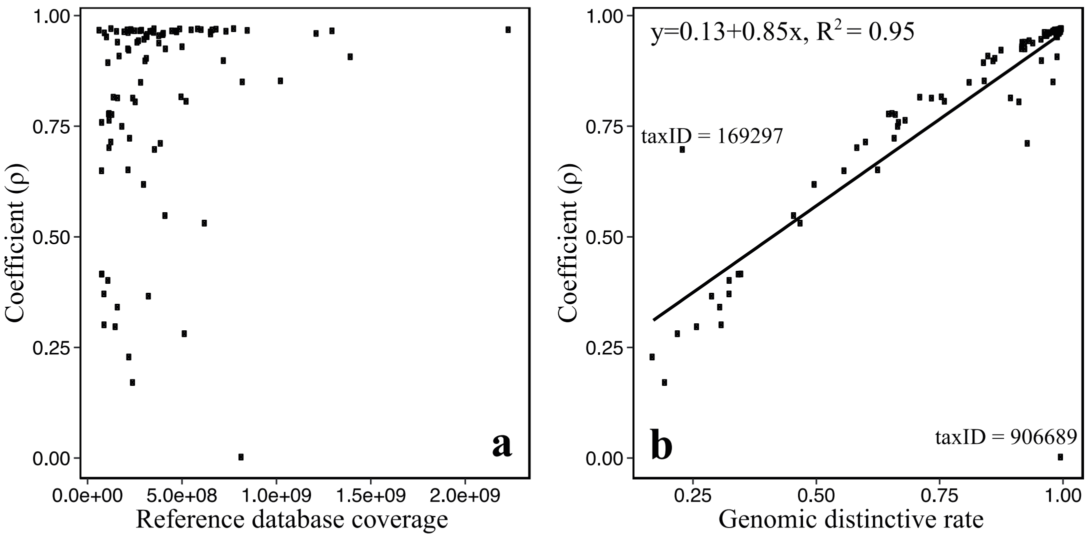

Supplementary Figure 9.5.2.2 | a, Correlation between the taxonomic classification efficiency to the database coverage. b, Correlation between the taxonomic classification efficiency to the genomic distinctiveness. Each dot represents a plant. The 2 outliersin b: *Dendrobium catenatum* (taxaID=906689) and *Aegilops tauschii subsp. tauschii* (taxaID=169297).

9.6 Megafauna authentication

9.6.1 Mammalian taxa grouping

Woolly mammoth (*Mammuthus primigenus*) are closely related to African elephant (*Loxodonta africana*) both genetically and phylogenetically[91], allowing the mammoth genomes to be assembled by aligning fragmented DNA sequences retrieved from mammoth skeletons against the African elephant genomes[79]. The resulting genomic similarities between the two species in the reference database raise a high chance of mis-assigning mammoth DNA to elephant. The test in ref. 33 evidenced that in the sub-Arctic ancient eDNA dataset, shotgun sequenced DNA that identified as elephant had been originated from mammoth, and hence required manually corrected. This is confirmed by our data, and had been proven also necessary for other species, such as the identifications of woolly rhinoceros and snow hare as discussed above (see Section 9.2.5 and Supplementary Figure 9.2.5.1).

Additionally, in this multiple-continental study covering 50,000 years, different species, subspecies, variants and populations of an animal can be distinct variations in distribution across different regions during different times. Genes of subdivisions of an animal are divergent (for example ref. [92] and the mitochondrial haplotypes discussed in Section 14). Efficiencies for identifying an animal from different times and regions based on matching its DNA to the reference genomes which have been reconstructed from a few individuals, will therefore be skewed. In order to investigate the Holarctic distributions of mammals and ensuring that they are comparable across time and space, instead of correcting the misidentifications, we clustered the identified mammalian taxa into genus and family levels, respectively, and use the family if there is no ambiguous arctic genus under the family, or the genus to reflecting arctic fauna distributions (see Supplementary Table 9.6.1).

Supplementary Table 9.6.1 | Taxonomic group for arctic faunas

Fauna	Taxa	Taxonomic rank
Bear	*Ursidae*	Family
Beaver	*Castor*	Genus
Bison	*Bison*	Genus
Camel	*Camelidae*	Family
Caribou	*Rangifer*	Genus
Deer	*Alces*	Genus
Deer	*Odocoileus*	Genus
Fox	*Vulpes*	Genus
Hare	*Leporidae*	Family
Horse	*Equus*	Genus
Lion	*Felidae*	Family
Mammoth	*Elephantidae*	Family
Muskox	*Bubalus*	Genus
Sheep	*Ovis*	Genus
Vole	*Microtus*	Genus
Wolf	*Canis*	Genus
Woolly rhinoceros	*Rhinocerotidae*	Family

9.6.2 DNA damage

We used MapDamage[93] (version 2.0.8) to confirm the presence of damage on DNA from animals that are abundant in DNA sequences and with good database coverage, i.e. taxa that have sufficient reads and reference genomes for generating reliable damage models. See Supplementary Table 9.6.2 for a list. A dedicated database for this purpose was constructed for each of the 11 tested animal, including (i) a subset of all entries for the animal in nt and RefSeq (The taxaIDs used for screening entries are supplied in Supplementary Table 9.6.2 and (ii) the complete genome(s) in the reference database section 2 (See Section 9.2.1), if available. The database for each animal was then indexed via "bwa index"[94] following default settings. A human (*Homo sapiens*) full genome was also included to control the potential human contaminates. A total of 12 databases were constructed.

All quality-controlled reads from each sample were then mapped against each of the 12 databases with "bwa aln -l 1000"[94,95], and then converted into bam format using "bwa samse" and "samtools view -F 4 -q 30 -bS"[96]. The resulting bam file contained only

mapping reads for each of the 12 mammals on each sample. We then merged the 12 bam files for each sample with "samtools merge", and sorted the merged bam file with "samtools sort". Program ngsLCA (https://github.com/miwipe/ngsLCA) was then applied to assign the lowest common ancestor of all hit taxa to each of the reads with multiple alignments (-editdistmin 0 -editdistmax 10). From each merged bam file, we then extracted the alignments that mapped exclusively to one of the 11 animals, which were used as input to calculate the damage patterns for that animal in each sample.

Frequencies of 5′ C to T and 3′ G to A were computed and the standard deviations were calculated in MapDamage, with reference sequence names ignored when tabulating reads (--merge-reference-sequences). Some damage patterns are shown in Supplementary Figure 9.6.2.1. In order to show the general damage patterns for different animals across samples, the average C to T and G to A mutation frequencies of the first position (F) and the mean of 4 to 25 positions (R) on identified DNA strands were calculated, and the F/R rates were used as a parameter to evaluate the ancient DNA post-mortem damage degree. The values of F/R rate for six animals are shown in Supplementary Figure 9.6.2.2, and the complete damage rate for all tested animals are supplied in Supplementary Data 6.

Supplementary Table 9.6.2 | NCBI TaxaID used for screening the entries for animals in nt and RefSeq

Fauna	NCBI TaxaID	Scientific name
Bear	9643	*Ursus americanus*
Bear	29073	*Ursus maritimus*
Bison	304335	*Bison antiquus*
Bison	9901	*Bison bison*
Bison	43347	*Bison bison athabascae*
Bison	43346	*Bison bison bison*
Bison	9902	*Bison bonasus*
Bison	1874132	*Bison bonasus caucasicus*
Bison	268291	*Bison priscus*
Caribou	9869	*Rangifer*
Caribou	9870	*Rangifer tarandus*
Deer	9851	*Alces*
Deer	9852	*Alces alces*
Deer	9853	*Alces alces alces*
Deer	341674	*Alces alces gigas*

Deer	162953	*Alces alces cameloides*
Deer	162954	*Alces alces pfitzmayeri*
Deer	225176	*Alces alces shirasi*
Deer	1574408	*Cervus canadensis*
Deer	227166	*Megaloceros giganteus*
Deer	227165	*Megaloceros*
Fox	9625	*Vulpes*
Fox	9627	*Vulpes vulpes*
Fox	494514	*Vulpes lagopus*
Fox	383736	*Vulpes lagopus beringensis*
Hare	9980	*Lepus*
Hare	62618	*Lepus arcticus*
Hare	62621	*Lepus timidus*
Hare	2059307	*Lepus timidus hibernicus*
Hare	48086	*Lepus americanus*
Hare	62620	*Lepus othus*
Horse	9789	*Equus*
Horse	9796	*Equus caballus*
Horse	1114792	*Equus ferus*
Human	9606	*Homo sapiens*
Mammoth	37348	*Mammuthus*
Mammoth	37349	*Mammuthus primigenius*
Sheep	9943	*Ovis dalli*
Sheep	72761	*Ovis dalli dalli*
Sheep	56194	*Ovis nivicola*
Sheep	1867111	*Ovis nivicola alleni*
Sheep	1867110	*Ovis nivicola koriakorum*
Sheep	1867112	*Ovis nivicola lydekkeri*
Sheep	1983725	*Ovis nivicola nivicola*
Sheep	1983726	*Ovis nivicola tschuktschorum*
Wolf	9612	*Canis lupus*
Wolf	1320375	*Canis lupus arctos*
Wolf	1341016	*Canis lupus campestris*
Wolf	443256	*Canis lupus lupus*
Wolf	9614	*Canis latrans*
Wolf	45781	*Canis rufus*
Woolly Rhinoceros	222862	*Coelodonta*
Woolly Rhinoceros	222863	*Coelodonta antiquitatis*

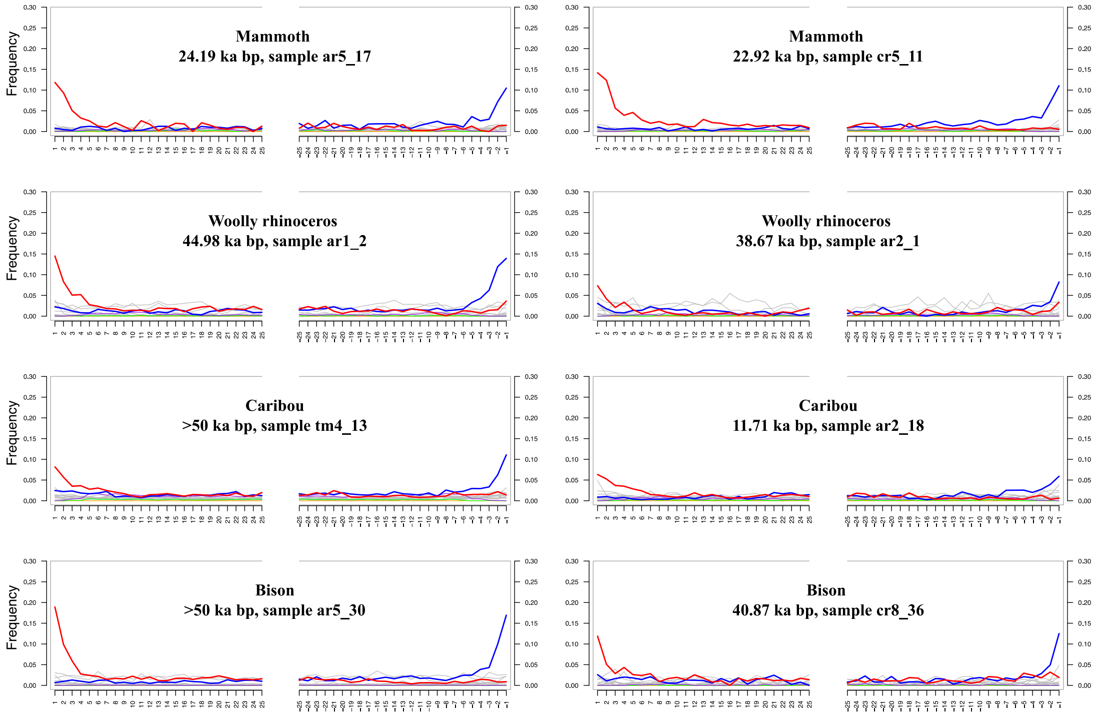

Supplementary Figure 9.6.2.1 | Examples for megafauna DNA damage patterns. In each plot, the substitution frequencies at 5′ end (left), and 3′ end (right) are illustrated for each position, with red line for C to T substitutions, blue line for G to A substitutions, and grey line for all other substitutions.

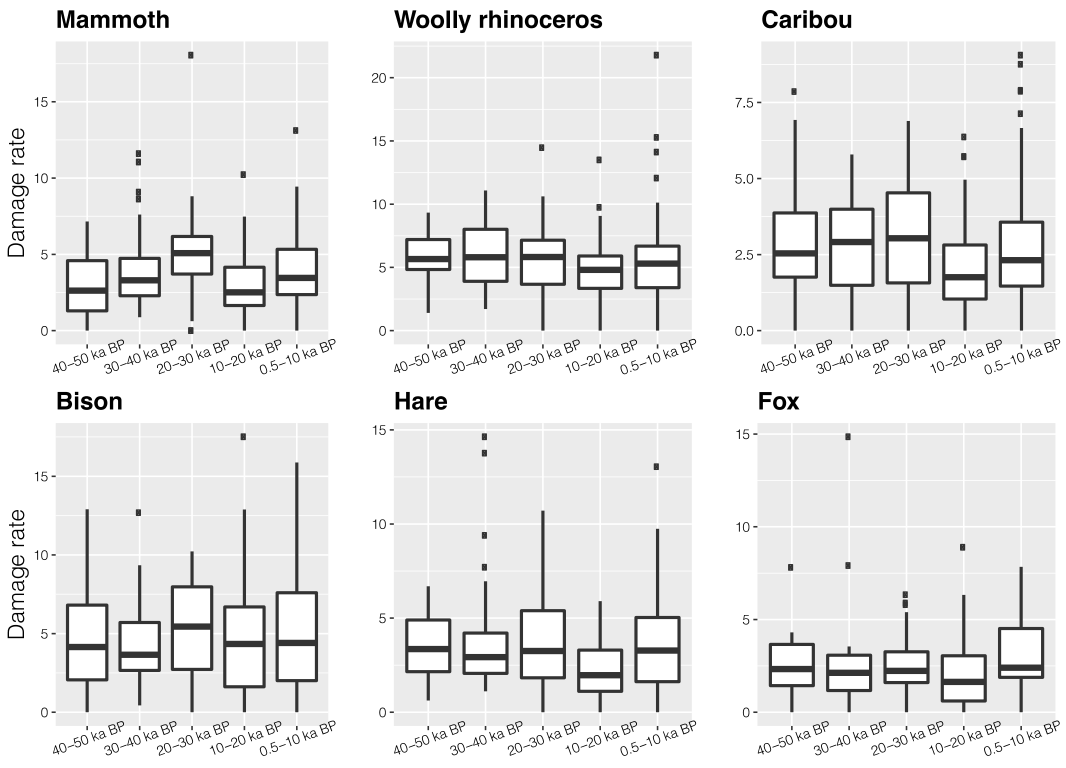

Supplementary Figure 9.6.2.2 | Fauna DNA damage degree (F/R rate). The bar denotes the median; the lower and upper hinges denote the 25th and 75th percentiles, respectively; the upper and lower whisker extended from the hinge denote the largest value no further than 1.5 * IQR (inter-quartile range) and the smallest value at most 1.5 * IQR of the hinge, respectively; data beyond the end of the whiskers are shown as outliers. Sample size (n) used for calculating damage degree: mammoth, 249; woolly rhinoceros, 335; caribou, 423; bison, 307; hare, 298; fox, 159; details are supplied in Supplementary Data 6. The first bases at each end on the DNA strands have significantly higher deamination substitution frequencies, confirming that the identified megafauna DNA is ancient-originated. The rates are not higher for older samples, indicating that the DNA deamination damages have no evidently difference among different age groups within the 50 kyr interval under the arctic frozen condition.

9.6.3 Thresholds for megafauna authentication

Compared to plants, animals are sporadically distributed across the landscape. The lack of identified mammalian DNA in some samples could therefore be ascribed to the absence of animal traces, rather than insufficient sequencing depth. The rarefaction method is therefore not capable for evaluating sequencing depth for the animal identification. We

therefore set a threshold requiring at least one million quality-controlled reads in a sample for a valid investigation of the megafaunal community. Out of 535 samples, 495 samples passed the filtering.

We then mapped all identified reads for each animal in every sample against the database DB2 constructed in Section 9.2.1 (the complete reference database excluding PhyloNorway) following the method described in Section 9.2.4, but parsed all alignments with allowing a maximum of 2 mismatches. Only the reads still exclusively mapped to the tested animal, *i.e.* perfectly matched to the tested animal and meanwhile not mapped to any other taxa even with 1 or 2 mismatches, were retained as the animal's identified reads. We required at least 5 exclusively mapped reads in a sample for an animal to confirm its existence. The extinct megafaunas (mammoth and woolly rhinoceros) were further authenticated by the DNA damage patterns with requiring the F/R rates greater than 2.

After these filters, some animals were rarely detected across the Arctic (*e.g.* deer and muskox, see Supplementary Table 9.6.3), despite they had been abundant regionally or globally, likely due to lack of proper reference genomes that resulted in unreliable identifications. We therefore removed animals with less than 20 occurrences from the subsequent analysis to be conservative. A full table of the animal distribution matrix can be found in Supplementary Data 6.

Supplementary Table 9.6.3 | The number of samples with animal identified.

Fauna	Number of samples
Camel	1
Bear	4
Beaver	1
Bison	38
Deer	7
Fox	1
Hare	192
Horse	162
Lion	11
Mammoth	159
Muskox	0
Caribou	110
Sheep	5

Vole	173
Wolf	64
Woolly rhinoceros	23

10. Effect of sample type on identified plant

The sampling sites come from localities across continents with varying topography, elevation, and glacial and climatic history. The landscapes could therefore be very distinctive in different regions, resulting in differences in the availability of material types that can be sampled across regions. The majority of the samples from Northwest and Central Siberia, for example, are from valley exposures and *yedoma*, in contrast to the North Atlantic where nearly half of the samples come from lake sediments. These differences are partly due to sample availability, but also to differences in glacial and climatic history, *i.e.* the ice-free landmass of Northwest and Central Siberia resulted in direr environment, whereas Northern Atlantic sites having fair temporal depth are mostly lakes distributed along the coastlines, as the deglaciation in this region began at the boundary between glacier and sea and consequently the sediment deposition commenced earliest in coastal areas[97,98].

In this section we investigate the effects of material types on the recovered vegetation. According to the formation of the sedimentary successions, samples were broadly divided into 2 types: permafrost (aeolian deposit, including *e.g.*, loess, *yedoma* and stratigraphic exposures in river valleys) and lake sediment (lacustrine deposit).

10.1 Sample type distribution and effect on vegetation diversity

Supplementary Figure 10.1.1 shows that there are more lake sediment samples in North Atlantic, and that also increased along the timeline until Mid-Holocene. However, the number of genera identified from lake sediment (averagely 8.35 genera per sample) is not significantly different (Supplementary Figure 10.1.2) to that from permafrost (averagely 8.07 genera per sample), indicating that there is no notable bias for the recovered vegetation diversity from the 2 sample types.

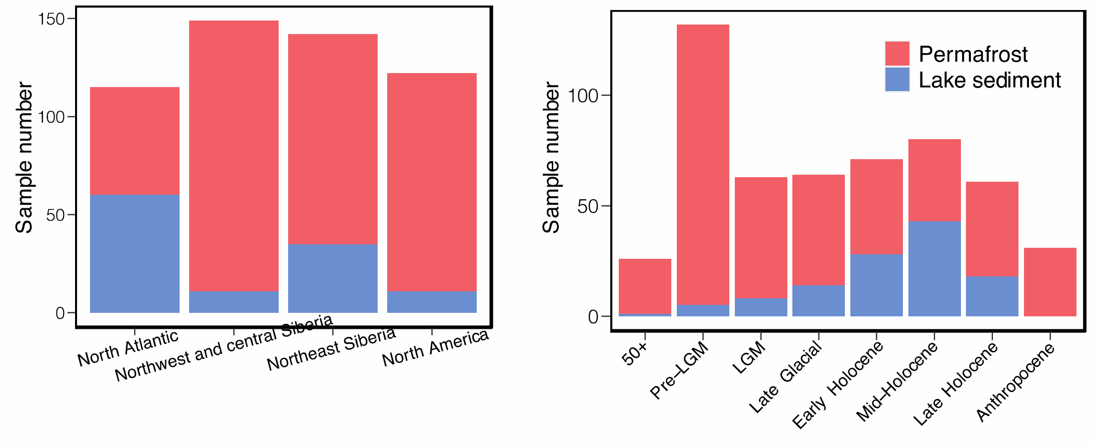

Supplementary Figure 10.1.1 | Sample types partitioned by regions and time intervals

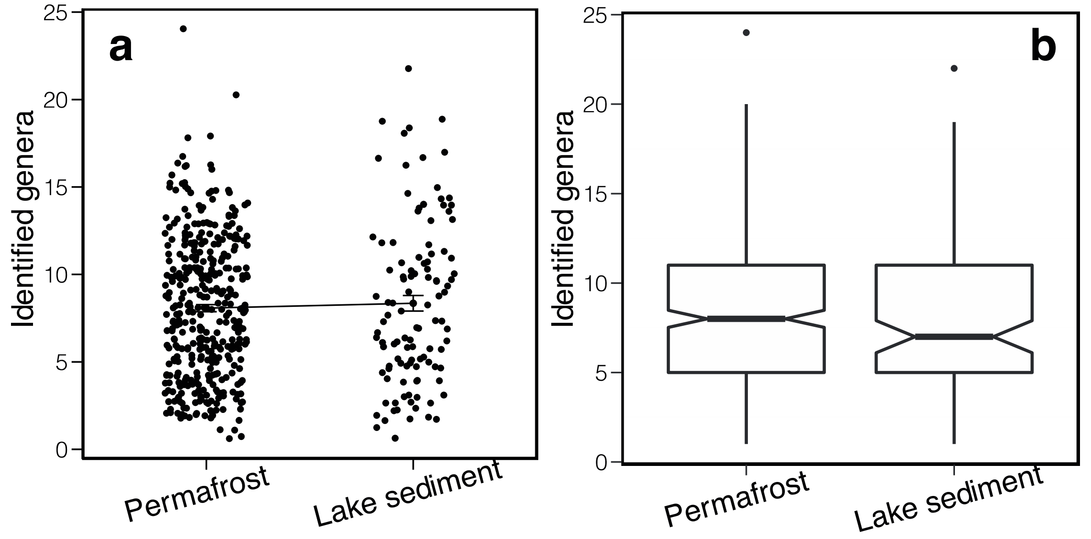

Supplementary Figure 10.1.2 | Plant genera identified in different sample types. A total of 499 samples passed the filtering (Section 9.5.2), of which 111 are lake sediment, and 388 are permafrost. **a,** each dot denotes a sample. Mean and the standard error are also shown. **b,** notched boxplot with outlier (one for each) excluded. The middle line denotes the median; the notches extend 1.58 * IQR / sqrt(n), indicating a roughly 95% confidence interval for comparing medians; the whisker extended from notches denote the largest the smallest value with outliers excluded.

10.2 Effect on algae and aquatic proportion

Lake sediment generated a higher proportion of algae (averagely 1.92%) and aquatic forbs (averagely 30.25%), compared to permafrost (0.02% and 1.20%, respectively). However, these differences can also be resulted from the regional floral differences, i.e. (i) there are

more lake sediment samples in North Atlantic (n=57) than the other 3 regions combined (n=54), and (ii) North Atlantic had been wetter than other regions (see Extended Data Figure 1d in the main text) (especially after the LGM due to deglaciation[97,99], for which all the North Atlantic lake sediment samples come from), and therefore its vegetation should have contained higher aquatic proportion. Given these two reasons, the aquatic rate is thus expected to be higher in lake sediments than other sample types regardless of the possible differences introduced by sample types.

To examine this, we compared the algae and aquatic forb proportion solely reflected by lake sediment in different regions (Supplementary Figure 10.2.1). It indicates that lake sediments in North Atlantic generated much higher proportion of algae (averagely 3.47%) and aquatic forbs (averagely 43.75%), compared to Northwest and central Siberia (0.00% and 10.48%, respectively), Northeast Siberia (0.39% and 17.88%, respectively), and North America (0.16% and 14.84%, respectively). This agrees the assumption in last paragraph, suggesting that it is mainly the floral differences contributing to the algae and aquatic proportion changes we observed in the overall floristic patterns. The aquatic proportion recovered by lake sediment also changed among time periods (Supplementary Figure 10.2.2), with further considering that the overall aquatic rate peaked at LGM and moved up and down after LGM (Figure 2a in the main text), while the proportion of lake sediment was very low in LGM and steadily increased thereafter (Supplementary Figure 10.1.1). The overall aquatic plant patterns therefore mostly reflect the ecological changes, rather than the bias of sample types.

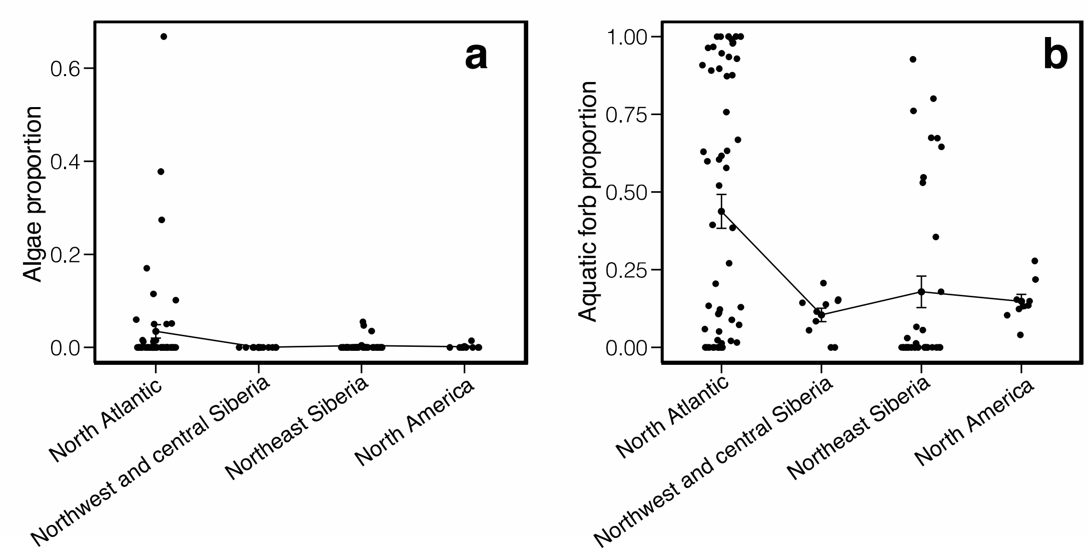

Supplementary Figure 10.2.1 | The Algae (a) and aquatic forb (b) proportion recovered from lake sediment in different regions. Each dot denotes a sample; mean and the standard error are also shown (n=111 lake sediment samples).

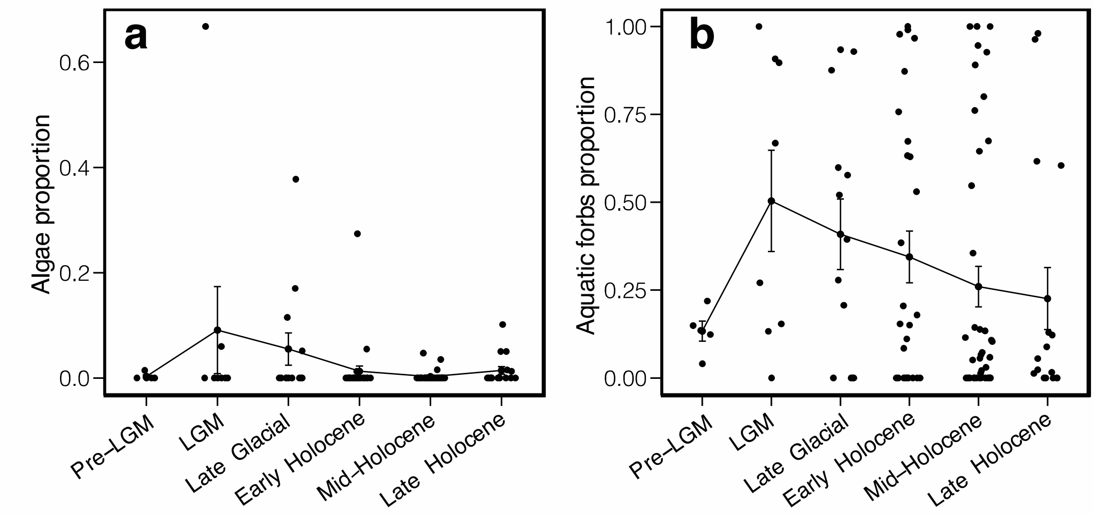

Supplementary Figure 10.2.2 | The Algae (a) and aquatic forb (b) proportion recovered from lake sediment within different time intervals. Each dot denotes a sample; mean and the standard error are also shown (n=111 lake sediment samples).

11. Metabarcoding *versus* shotgun metagenomics

Two modules were applied for comparing the capacities of metabarcoding and shotgun metagenomics in taxa identifications. (i) We conducted the 2 sequencing techniques in parallel on 14 DNA extracts (two processing batches tm1 and tm2), to directly compare

the retrieved taxonomic profiles. (ii) We compared the floristic profiles reconstructed by this study and the previous metabarcoding study[14] on the 131 overlapping samples of the 2 datasets.

11.1 Direct comparison

11.1.1 Data generation

We applied the trnL P6 loop primers[100] (GGGCAATCCTGAGCCAA, CCATTGAGTCTCTGCACCTATC) for plant, and 12S V5 primers (TAGAACAGGCTCCTCTAG, TTAGATACCCCACTATGC) for vertebrate barcodes amplifications, based on the primer comparison and evaluation conducted by ecoPrimers[84]. PCR reactions were set up using High-Fidelity DNA Polymerases kit (Thermo Fisher Scientific), with each 25 µL reaction containing 0.2 µL Hifi enzyme, 2.5 µL 10x Hifi buffer, 1 µL extracts, 1 µL forward primer (10mM), 1 µL reverse primer (10mM), 12.8 µL water, 1 µL Mg_2SO_4 (50mM), 0.5 µL DNTPs (10mM for each), 2 µL BSA (20mg/mL), and 3 µL DMSO. PCR was operated with a pre-incubation at 94 °C for 4 minutes, followed by 55 amplifying cycles of 94 °C denaturing for 30 seconds, 55 °C (vertebrate) or 52 °C (plant) annealing for 30 seconds, 68 °C elongation for 45 seconds, and a final elongation step at 72 °C for 7 minutes. For every 7 samples, one extraction control was introduced and amplified together with the samples.

Eight different primer tags (6 extra bases attaching on both forward and reverse primers) were added on all primers. For every PCR run, a primer negative control was introduced for each primer pair. The amplified PCR products were then visualized by 1% agarose gel electrophoresis. For reactions with positive bands appearing on the gel, we measured the DNA concentrations by Qubit 2.0, and pooled the reactions in equal DNA concentration. At least 5 successfully amplified reactions for both plant and vertebrate in a sample were required for pooling. The pools were then built into sequencing libraries[101] and sequenced on Illumina Hiseq 2500 (150BP pair-ended).

11.1.2 Data processing

We used OBItools[102] for metabarcoding data analysis. Read pairs in the original fastq files were first merged requiring a minimum alignment score of 40 (illuminapairedend --score-min=40). Unaligned singletons were removed by “obigrep”. All combined reads were

assigned to samples and divided into two separate datasets for vertebrate and plant, based on the PCR tags through “ngsfilter”. PCR duplicates were then removed using “obiuniq” to annotate the count of duplicates for each unique read. Thereafter reads were filtered, requiring more than 5 counts and read length in between 10–180 bases for plants and 60-150 bases for vertebrates. The “obiclean” was then used to classify reads into groups based on their similarity (1 mismatch allowed), and the less abundant reads in a group were considered as variants if their reads count ratios were less than 0.01. These variants were then merged into the read with highest count.

The reference database used for taxa identification includes three parts: (1) “pln” and “vrt” divisions of European Nucleotide Archive (EMBL release 142), which overlap with the NCBI genbank plants, vertebrates and mammal database, (2) The NCBI RefSeq mitochondrion database, and (3) The Arctic and Boreal plant trnL database in ref. 14. The first two databases were first converted into OBItools-fasta format by “obiconvert”. The trnL and V5 barcodes were then extracted by in-silico PCR simulation through program “ecoPCR”, allowing 3 mismatches between the primer and target sequence. Duplicated barcodes in databases were merged into a single record, assigning the lowest common ancestors of all taxa it represents. Only the records with taxonomic resolution higher than family level (including family) were kept. Sequencing reads that passed the filtering were then matched against the databases through “ecotag”, requiring a minimum identity rate 0.95.

We set the copy number (counts) threshold at 10 and applied the restrictive strategy for authenticating the identified taxa as suggested in ref. [103]. Three positive detections were required to confirm of the identification of a taxon. All taxa detected by the three reference databases for each sample were merged as the final taxonomic profile for that sample. Taxa detected in the blank controls (both extraction controls and primer controls) were subtracted from the corresponding taxonomic profiles.

11.1.3 Comparison

Taxa identified by the shotgun sequencing of batch tm1 and tm2 were extracted from the authenticated plant profile generated in Section 9.5. We merged all the taxa identified in the 7 samples of each processing batch and compared the accumulated taxonomic profiles, to alleviate the effects of sample-wise heterogeneities.

As illustrated in Supplementary Figure 11.1.3, shotgun sequencing retrieved much higher plant diversity than metabarcoding in these samples. There were only 1 species and 2 genera detected by metabarcoding across the 7 samples in batch tm1, indicating the method most likely been failed for these samples, despite the PCR had been replicated for more than 10 times for each sample in order to gain sufficient positive reactions. Further, all vertebrate taxa detected by metabarcoding in the 14 tested samples were common contaminants that also appeared in the corresponding negative controls. Whereas shotgun detected megafaunas including both extinct and extant species such as woolly mammoth, caribou, bison and horse. This comparison suggests that the shotgun metagenomics is more suitable for our samples and outperforms metabarcoding for the retrieved plant and animal diversities.

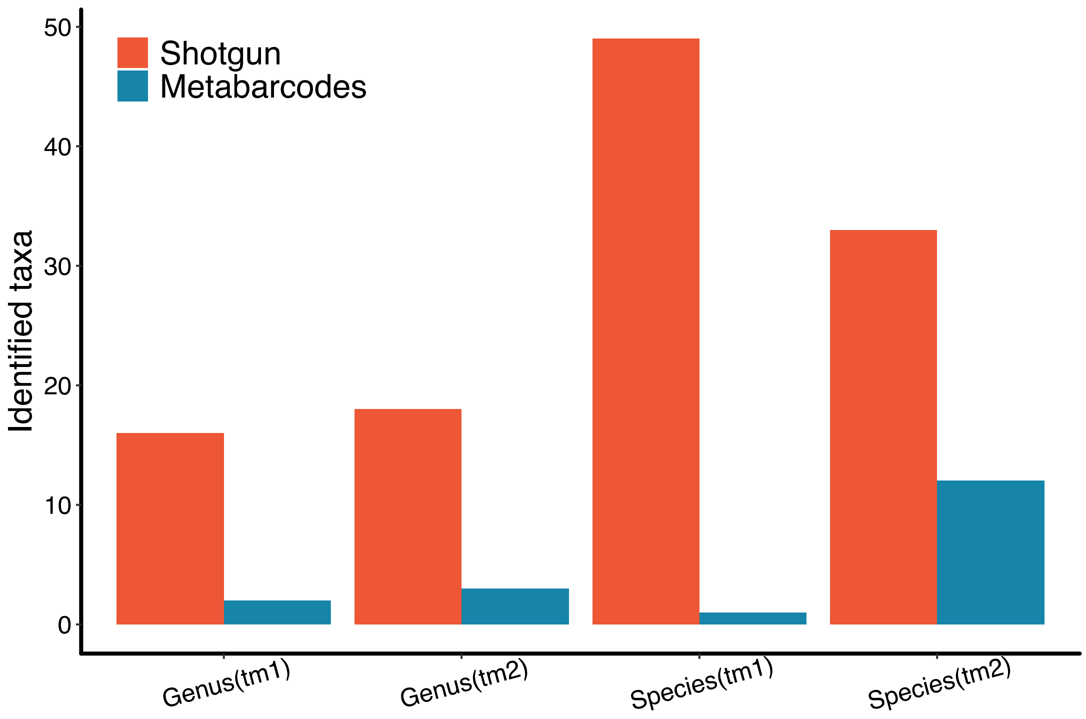

Supplementary Figure 11.1.3 | Plant identified by metabarcoding and shotgun metagenomics on two processing batches.

11.2 Comparison with the 2014 metabarcoding dataset

We sequenced 131 samples that had been metabarcoding sequenced in 2014[14] (referred as metabarcoding dataset in this section), offering a chance to extensively compare the two techniques. We first re-analyzed the metabarcoding dataset with applying a more advanced metabarcoding pipeline[104], against an updated reference database including the EMBL release 142 and a curated arctic, boreal and bryophyte database[14,105,106]. Filtering and authentication were carried out with a custom script (https://github.com/Y-Lammers/MergeAndFilter) and followed the same parameters used for the 2014 study, excepting changing the filtering of minimum PCR replicates from 1000 to 500 to increase the number of identified taxa. However, we found the reanalysis having no significant improvement over the 2014 results (the average identified genera in the new analysis is 2.43 *versus* 2.38 in the 2014 results). Therefore, the original metabarcoding result was used for the comparison.

We obtained the identified plant profiles of the 131 samples from both datasets. There are a total of 124 floristic taxa identified in the metabarcoding dataset across the 131 samples, but only 49 to species level. We therefore compared the two datasets at genus rank, for which we also have more confidence for the shotgun results (see Section 9.4).

On average, 8.4 genera were identified in each sample by shotgun sequencing, while only 2.7 genera by metabarcoding. In 128 out of 131 samples, shotgun recovered more plant genera (see Supplementary Figure 11.2.1), and the genera detected by metabarcoding in a sample were mostly a subset of its shotgun data.

We grouped the 131 samples into the pre-defined age intervals (Section 2) to investigate the diversity of floristic communities captured through the 2 sequencing techniques (Supplementary Figure 11.2.2). All genera for each age interval were combined as an assemblage to represent the plant diversity for that interval. The floristic community reconstructed by both shotgun and metabarcoding experienced similar trend, although shotgun recovered higher diversities at all intervals: on average 27.7 genera compared to 17.4 in metabarcoding. However, if the average number of genera detected in the samples within an age interval was used to represent the diversity (sample-wise), shotgun sequencing retrieves much higher diversities, and the variation trends also became divergent (Supplementary Figure 11.2.2). The combined assemblages for the 2 datasets

generated similar results, while the sample-wise diversities of metabarcoding are remarkably lower and varies distinctively, indicating that the metabarcoding dataset most likely introduced bias among different samples, where the plants identified in a sample were likely a subset of its genuine floristic community.

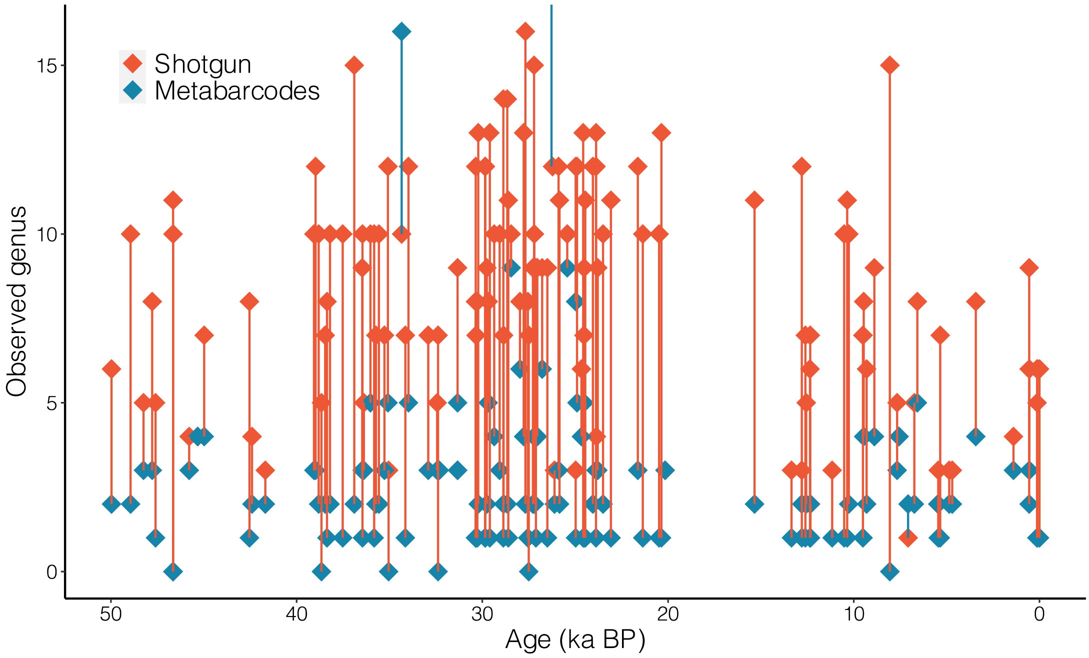

Supplementary Figure 11.2.1 | Number of genera identified in every sample by the shotgun sequencing of this study and the 2014 metabarcoding study.

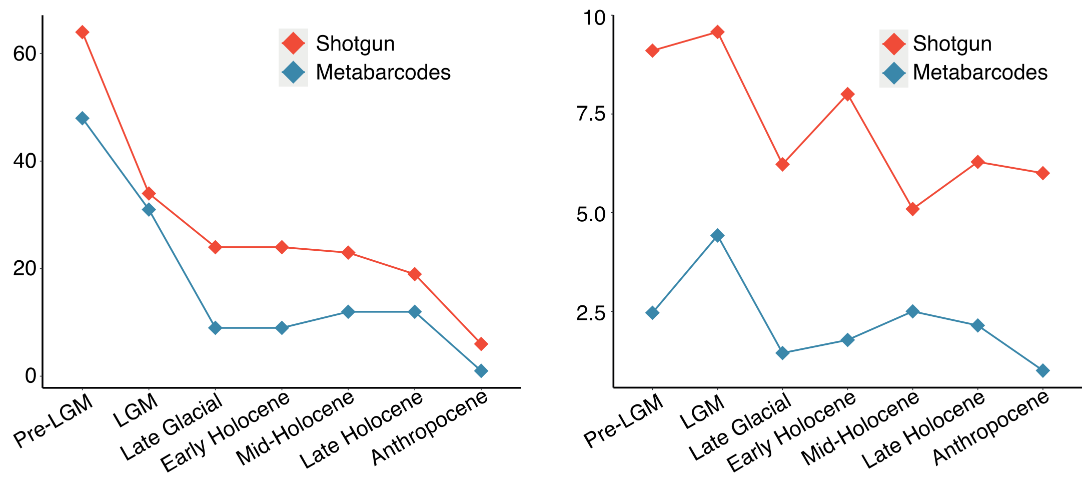

Supplementary Figure 11.2.2 | Trend of the accumulated genera count across the age intervals, and the average genera count across age intervals, retrieved by shotgun and metabarcoding.

12. Climate and human distribution modelling

12.1 Modelled paleoclimate

We generated climate variables using a paleoclimate emulator, precisely following the methodology in ref. 107. The approach applies a Gaussian process emulation of the singular value decomposition of ensembles of runs from the intermediate complexity atmosphere-ocean GCM PLASIM-GENIE[108] forced with time-varying input data. Spatial layers of monthly temperature and precipitation at the scale of 1 degree were emulated at 1,000 year intervals, forced by time-series of CO2[109], orbit[110] and ice volume[111], assuming the climate is in quasi-equilibrium with the forcing in each time interval. The emulated paleoclimate anomalies at climate model resolution (5°) were downscaled onto the observed modern (CHELSA; ref. 112) baseline climatology at 1° spatial resolution using bilinear interpolation. Emulated temperature anomalies were combined additively with baseline maximum and minimum monthly temperatures, assuming that temporal variability within each month is constant through time. Monthly precipitation was combined with baseline precipitation using a mixed multiplicative/additive approach[107].

Experimental set-up is detailed in ref. 107, including boundary conditions, and an extensive validation against model inter-comparisons of the mid-Holocene, the Last Glacial

Maximum, the Last Interglacial and the mid-Pliocene warm period, with glacial-interglacial variability validated against observationally based global temperature reconstructions. We have supplemented these earlier validations in Supplementary Figure 12.1.1, which plots the downscaled mean annual temperature and precipitation for comparison with the LGM PMIP4 ensemble[113]. Emulated mean LGM annual temperature change in the 60-90°N latitude band of 11.3°C compares to the PMIP4 ensemble mean of 13.5°C (range 7.5 to 18.9°C), illustrating that PLASIM GENIE is approximately centred within this highly uncertain envelope. Furthermore, the emulator reproduces large-scale regionally heterogeneous hydroclimatic behaviour observed in a compilation of proxy records spanning 130 to 70 thousand years ago[114].

We note that in ref. 107, it also includes a discussion section on model caveats targeted at end-users. This includes discussions of the limitations of low resolution and intermediate complexity, the restricted forcing (only CO2, orbit and ice sheets), the neglect of asymmetric ice sheet growth and decay, and the limitations of the downscaling approach. The land sea mask is not altered in our simulations, which could lead to a small underestimation of climate change in the region of the Fennoscandian ice sheet.

From the climate dataset, we used the BioCalc R package[115] to calculate four environmental variables considered likely drivers of ecological and human distributions changes: annual mean temperature, temperature seasonality, annual precipitation, and precipitation seasonality (bioclimatic variables bio1, bio4, bio 12, and bio 15, respectively).

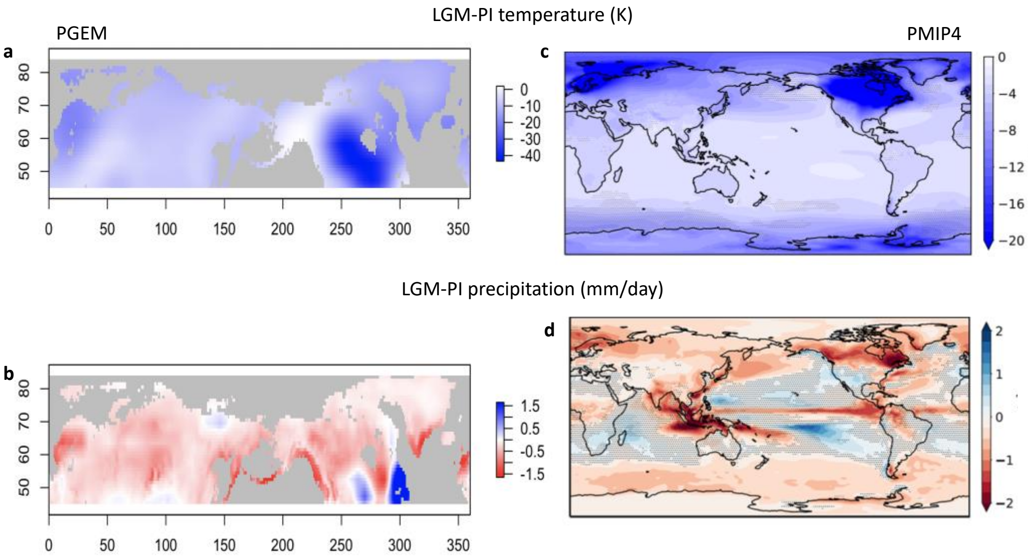

Supplementary Figure 12.1.1 | Comparison between PLASIM GENIE emulations (PGEM) and Paleoclimate Model Inter-Comparison Project 4 (PMIP4) simulation ensemble of the Last Glacial Maximum (LGM). Panels display the LGM change relative to preindustrial of (a) PGEM annual temperature, (b) PGEM annual precipitation, (c) PMIP4 annual temperature and (d) PMIP4 annual precipitation. Note the choice of a symmetrical precipitation legend masks an emulated precipitation change of up to 3mm/day over Newfoundland.

12.2 Human occurrence modeling

We developed distribution models to identify environmentally suitable conditions for paleolithic human occurrence in steps of 1,000 years from 5 to 31 ka and steps of 2,000 years from 32 to 47 ka, following the method described below.

First, we generated a human skeleton distribution database, by merging 3 souses. (i) We filtered the Canadian Archaeological Radiocarbon Database (CARD2.0)[116] by selecting the records with keyword “homo”, “human”, or “sapiens” in the columns “Taxa dated” or “Material dated”, then removed records without radiocarbon age or radiocarbon errors, out of the intCal20 calibrating curve, or with latitude lower than 45º N. (ii) We filtered the Radiocarbon Palaeolithic Europe Database (v27)[117] by removing records that are (a) with ambiguous sample or site information, (b) with latitude lower than 45º N, (c) without

radiocarbon ID, (d) with radiocarbon ages out of the intCal20 calibrating curve. (iii) Dated sample supplied in ref. [118-120].

Duplicates were removed and all ages were calibrated via the R package Bchron[38] based on the IntCal20[39]. Then, we filtered out all occurrences with a dating uncertainty of larger than +/- 500 years from 5 to 31ka, and larger than +/- 1,000 years from 32 to 47ka, resulting in 6,497 occurrences.

Occurrence data were assigned to grid cells with the same resolution as the climate dataset generated in Section 12.1, resulting in a final dataset of 1646 occurrences, after 2,000-years interval means calculated for each variable from 32 to 47 ka, to align with deep time dating uncertainty in the occurrence data. An ensemble of five different algorithms (Maxent[121]; generalized linear model, GLM; generalized additive model, GAM, as implemented by bam in the R package mgcv[122]; artificial neural network, ANN[123]; and random forest, RF[124]) was used to characterize the climatic niche of modern humans, using the R package biomod2[125]. Maxent was limited to 10,000 iterations; linear, product, and quadratic feature types were implemented with a beta regularization parameter of 0.95. GAM was limited to a maximum of 100 iterations. To calibrate models at each time step, five sets of pseudoabsences were generated by randomly sampling from a disk around occurrence points at a distance from 150 to 1250 km; each set included five times the number of occurrences as the time step of interest.

For validation purposes, we split the occurrences at each time step such that 80% were used for model training and the remaining 20% were used to validate them. This procedure was repeated 5 times for each time step dataset. We validated all models by assessing the Area Under the Receiver Operating Curve (AUC) and True Skill Score (TSS). We repeated the procedure from presence and pseudoabsence splitting through validation 5 times for each time step dataset. We then composed ensemble models at each time step by weighing the results of each algorithm using AUC. Given the reduced number of cases in our dataset from 5 to 11 ka, we generated a single ensemble model for time steps from models for 12 to 16 ka which was then projected at each time step from 5 to 11 ka and validated secondarily with available occurrence points for each time step.

We then projected models for each time step back into geographic space to infer the degree to which environmental conditions were suitable or unsuitable for paleolithic humans. We

inferred likely presence or absence of paleolithic humans by thresholding model projections to balance sensitivity and specificity of the resulting binary maps. Occurrences at each time slice used to train the models are mapped as “Occurrence”; eDNA sites are mapped for all time slices as “Paleo Site”. Maps are supplied in Supplementary Data 7. Further detail can be found in the script used to generate the models.

Finally, we extracted mean raw relative suitability and inferred human presence/absence at each time step at each eDNA site. Data is supplied in Supplementary Data 8. Further detail can be found in the script available in the GitHub repository (Section 15).

13. Spatiotemporal modelling of animal eDNA data

We were interested in modelling the presence or absence of animal eDNA reads using a model that could account for auto-correlation in space and time. We explain the models we used below, followed by the results obtained from fitting the model to our data.

13.1 The model

We took each eDNA site at each time point for which we have read data, and modeled it as the outcome of a Bernoulli(p) trial, where p is unknown. The Bernoulli probability parameter was, in turn, modeled as a standard logistic function of a linear combination of a global intercept, a spatiotemporal nugget and (possibly) some covariates. We used the data to infer the global intercept and the shape of the spatiotemporal field, and to learn which covariates were most informative of the present/absence data.

In formal notation, let h(s,t) denote the presence or absence of eDNA from a given animal at the sampling site in location s at time t (in years ago). We model h(s,t) as a Bernoulli random variable:

$$h(\boldsymbol{s},t) \sim Bernoulli\big(p(\boldsymbol{s},t)\big) \qquad (1)$$

In our case, s is a 2-element vector of polar coordinates with respect to the North Pole, and we use an azimuthal equidistant projection for all analyses below. We express the unknow Bernoulli probability parameter p(s,t) as a logistic function of a parameter q:

$$p(\boldsymbol{s}, t) = \frac{e^{q(\boldsymbol{s},t)}}{1 + e^{q(\boldsymbol{s},t)}} \tag{2}$$

The parameter q is then modeled using a linear mixed model, that might include a set of N external covariates:

$$q(\boldsymbol{s}, t) = \theta_0 + \sum_{l=1}^{N} \theta_l \, c_l \, (\boldsymbol{s}, t) + \gamma(\boldsymbol{s}, t) \tag{3}$$

Here, θ_0 is the model intercept, θ_l is the effect parameter for the lth covariate, and $\gamma(\boldsymbol{s}, t)$ is a spatiotemporal nugget that can be modeled using a spatiotemporal Gaussian random field (GF):

$$\gamma(\boldsymbol{s}, t) \mid \rho, \sigma, \omega \sim GF(\boldsymbol{0}, \boldsymbol{\Sigma}) \tag{4}$$

Here, $\boldsymbol{\Sigma}$ denotes a spatiotemporal covariance function. For this field, we chose a separable space-time model in which the spatial component is defined via the solution to a Matérn stochastic partial differential equation (SPDE)[126], while the temporal component is defined using an auto-regressive model of order 1[127]. The hyperparameters ρ, φ, ω control the shape of the covariance function: ρ and σ are the range and standard deviation parameters of the Matérn SPDE, and can be interpreted as the variation in the spatial field and as the typical distance between regions with low values and those with high values, respectively[128]. In turn, ω is the temporal autocorrelation parameter of the auto-regressive model. These hyperparameters were modeled using penalized-complexity priors[129].

We used the R package R-INLA[130-132], and followed recommended guidelines in ref. 133 in order to estimate the parameters of this model via the integrated nested Laplace approximation. To construct a spatiotemporal mesh to estimate the Gaussian field, we used a Delaunay triangulation with 200 spatial vertices on a hull defined by our data points, and 11 time knots (ranging from 0 to 50,000 years ago, in intervals of 5,000 years).

13.2 Predicting animal eDNA sampling probabilities

Given the eDNA data we have for a particular taxon in all our sampled sites, we were interested in estimating the probability that an unsampled site in a given location and time would also yield eDNA from that taxon. We filtered out animal data (Section 9.6.3) for which the ratio of presences over all sampled sites was less than 10%, and proceeded to estimate the probability of eDNA presence in the remaining taxa. Supplementary Figure 13.2.1 shows the inferred mean spatiotemporal field for each animal' probability of eDNA presence at 5 of the time knots, modeled without any external covariates. Supplementary Figure 13.2.2 shows an approximation to the variance of this estimate, computed by summing the marginal variance of the intercept and the marginal variance of the spatial-temporal nugget (without considering the covariance between these two parameters).

Some of the fields show large swaths of land with high presence probability, like mammoth, horse and hare. Certain animals show more concentrated probability distributions – especially wolf and vole eDNA – with spikes in the probability fields in particular locations of Siberia and North America

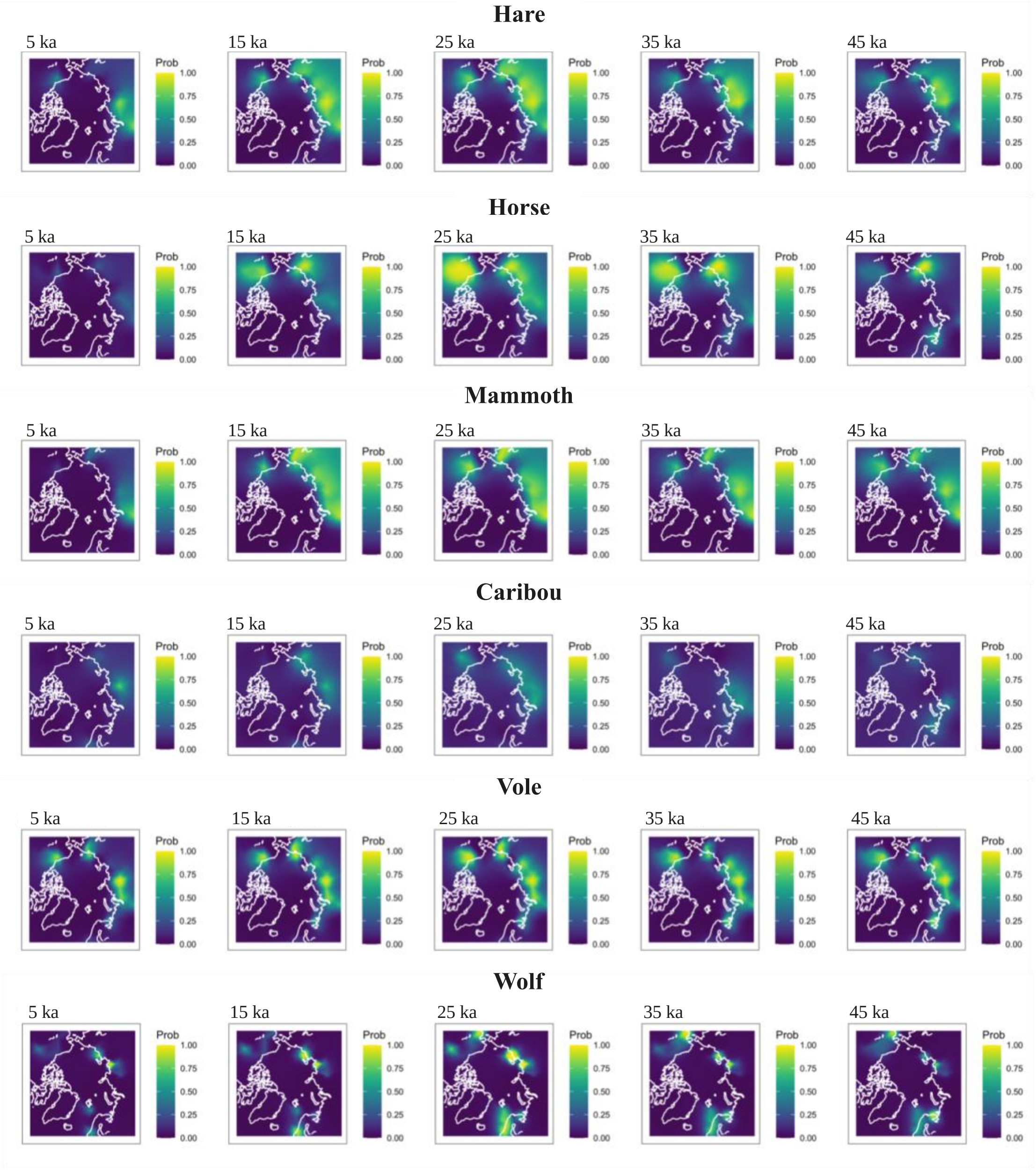

Supplementary Figure 13.2.1 | Inferred Bernoulli probability fields for the presence of eDNA from various animals living in the Arctic region. This was obtained after a logistic transformation of a fitted Gaussian spatiotemporal field.

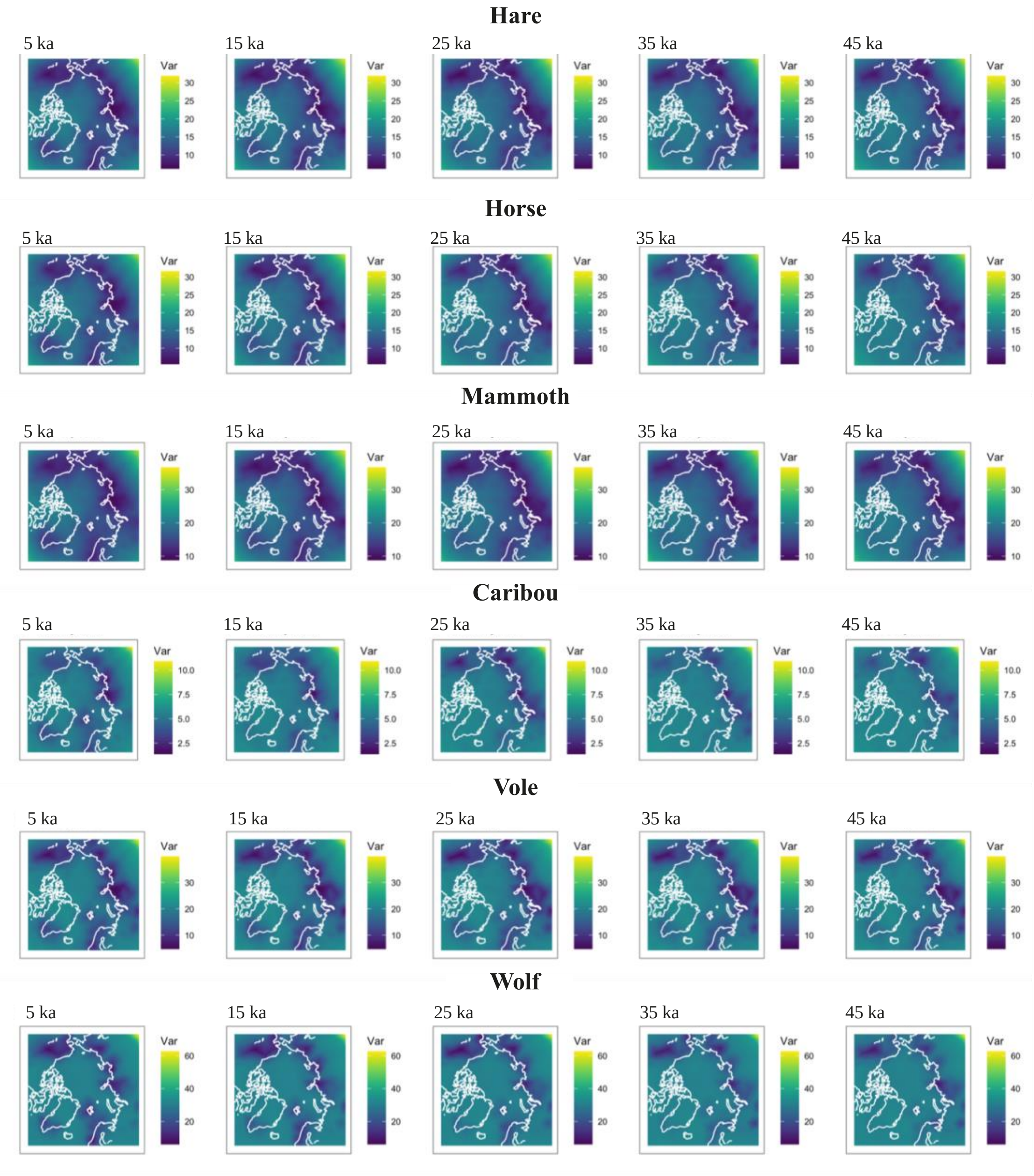

Supplementary Figure 13.2.2 | Approximation to the variance in the estimate of the spatiotemporal Gaussian random field for the presence of eDNA from various animals living in the Arctic region.

13.3 Modeling covariates

We aimed to find covariates that could contribute to predicting the presence/absence of eDNA from each of the remaining animal – caribou, hare, horse, mammoth, vole, and wolf - across the landscape through time.

Before inferring parameters, presence-absence animal and human covariates (Section 12.2) were min-max normalized so as to lie in [-1,1] (*i.e.* absences were recorded as -1 and presences as 1). As the plant diversity covariates (Section 9.5), we used the three sets of NMDS loadings for each site, from the plant NMDS analysis under K=3 (see main text). Climate covariates (mean annual temperature, mean annual precipitation, precipitation seasonality and temperature seasonality, see Section 12.1) were mean-centered and standardized, as were the plant NMDS covariates. Responses (animal presence/absence) were kept in their natural scale ([0,1]).

We used the Watanabe-Aikaike Information Criterion (WAIC)[134] and the negative sum of the logarithm of the conditional predictive ordinate for each observed site (hereby called CPO)[135], in order to compare models with different numbers of parameters[136]. The WAIC is a goodness-of-fit measure that includes a penalty term for the complexity of the model. The conditional predictive ordinate of a particular observation is equal to the posterior probability of that observation when the model is fit using all data except for that observation.

For each animal, we tested 10 different models with different sets of covariates (Supplementary Figure 13.3.1). We observe a strong drop in both these measures when including information about the presence or absence of eDNA from other animals into the model, for almost all the animals we considered, except for wolf. Drops of smaller magnitude are also observed when including plant information into the model, especially in the case of horse eDNA.

For each animal, we selected the model with the lowest WAIC, and estimated the regression parameters for each covariate included in the model (Figure 6a in main text). In general, the presence of an animals' eDNA tends to have positive effects on the presence of another animals' eDNA. This suggests that an animal's eDNA clusters with other animals' eDNA in space and time: samples where, e.g. one finds hare eDNA will tend to also contain vole and horse eDNA, while samples where hare eDNA is absent will also tend to lack these other animals' eDNA. We also find a positive effect of temperature seasonality on vole eDNA presence, and of caribou presence on wolf presence, among other interesting effects. Finally, the first dimension of the plant diversity NMDS analysis

has a negative effect on mammoth and horse presence, and the third dimension has a negative effect on hare presence.

We were interested in what would happen if we removed other animals as explanatory variables, and only used climate, humans or plants to fit the animal presence predictions (Figure 6b in main text). In this case, we find a positive effect of temperature seasonality on mammoth and vole presence and a negative effect of precipitation seasonality on hare and vole presence. The first dimension of the plant NMDS analysis has a negative effect on hare, horse, mammoth and vole, while the third dimension has a negative effect on hare, horse and vole. Additionally, we find a positive effect of human eDNA presence on hare eDNA presence.

Given that herbivores appear to co-occur quite strongly together, we also modeled herbivore presence as a response variable (recorded as 1 if any herbivore is present at a sample, and 0 if it is not). This time, we also considered other herbivores whose ratio of presences over total samples may be below 10%. We then used climate, human presence and private diversity as candidate explanatory variables for herbivore presence. In this case, though the fit scores do not show strong differences across models, the best model jointly includes only climate variables as covariates (Supplementary Figure 13.3.2). In particular, we observe that precipitation seasonality and annual precipitation have negative effects on herbivore presence (Figure 6d in main text).

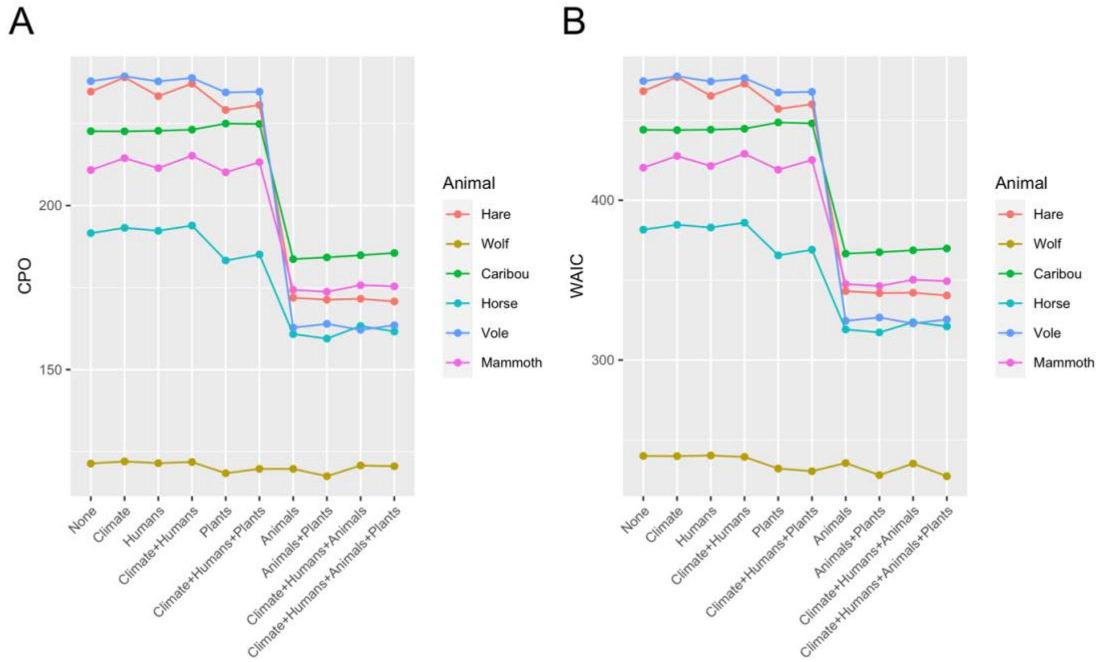

Supplementary Figure 13.3.1 | Model comparison for fitting presence/absence of animal eDNA. We computed two types of scores to compare model fit with varying numbers of covariates. Panel A: negative sum of the logarithm of the conditional predictive ordinate for each observed site (CPO). Panel B: Watanabe-Aikaike information criterion (WAIC). In both cases, smaller scores represent better model fits.

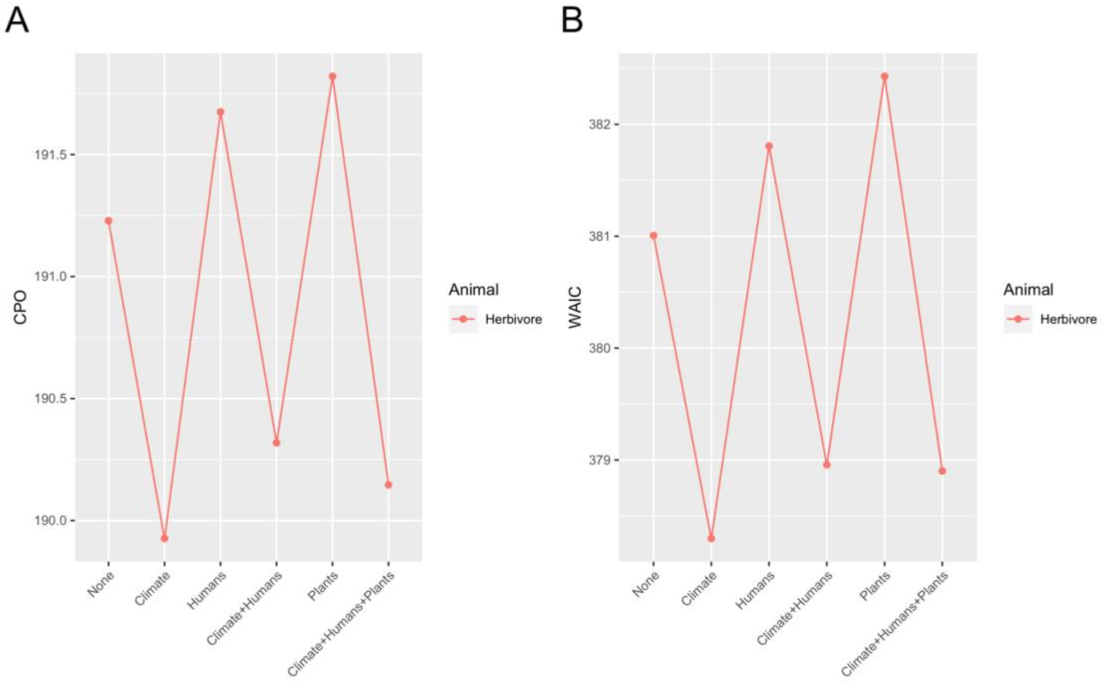

Supplementary Figure 13.3.2 | Model comparison for fitting presence/absence of herbivore eDNA. We computed two types of scores to compare model fit with varying numbers of covariates. Panel A: negative sum of the logarithm of the conditional predictive ordinate for each observed site (CPO). Panel B: Watanabe-Aikaike information criterion (WAIC). In both cases, smaller scores represent better model fits.

13.4 Partitions in time and space

To determine whether regional and/or temporal differences could be detected, we also ran the models partitioned by time periods and geographic regions. We partitioned the data into 3 regions of time - P1 (pre-LGM), P2 (LGM and Late Glacial), and P3 (Holocene) - and 4 regions of space (see Section 2). We show WAIC and CPO plots and effect size plots for the lowest-WAIC models fitted only on data from period P1 (Supplementary Figure 13.4.1 and 2), period P2 (Supplementary Figure 13.4.3 and 4) or periods P3 (Supplementary Figure 13.4.5 and 6). Within each of these temporal partitions, we used 4 time knots, and the same spatial grid as before. For the 4 spatial regions, we show WAIC and CPO plots and effect size plots for the lowest-WAIC models fitted only on data from North America (Supplementary Figure 13.4.7 and 8), from the North Atlantic (Supplementary Figure 13.4.9 and 10), from Northeast Siberia (Supplementary Figure 13.4.11 and 12) and from Northwest and central Siberia (Supplementary Figure 13.4.13 and 14). Here, we used 11 time knots and created a new spatial grid for each of the regions.

We note that the data for each these temporal and spatial partitions is smaller than in the previous analyses, leading to often very high WAIC or CPO scores, or very wide credible intervals for the effect size estimates (dominated by the wide priors on the Bayesian model), when covariates are scarce or highly imbalanced, so we urge caution in the interpretation of results from these subsets of the data.

Some of the previously observed effects may not be detectable in a particular partition simply due to lack of power. Focusing on the extinct fauna, we find that mammoth eDNA distribution is consistently and negatively associated with Plant NMDS1, and this inverse relationship continued from the LGM throughout the Holocene in all regions. Plant NMDS2 has a positive correlation with mammoth occurrence, but only during the LGM and Late Glacial, and in North America (Supplementary Figure 13.4.4 and 13.4.8). For horses, the only significant effects are seen in Northwest and Central Siberia, where their distribution, as with mammoths, is negative with respect to Plant NMDS1 and Plant NMDS3 (Supplementary Figure 13.4.14). These results suggest that the patterns seen more broadly are not being disproportionately influenced by specific regions or time periods.

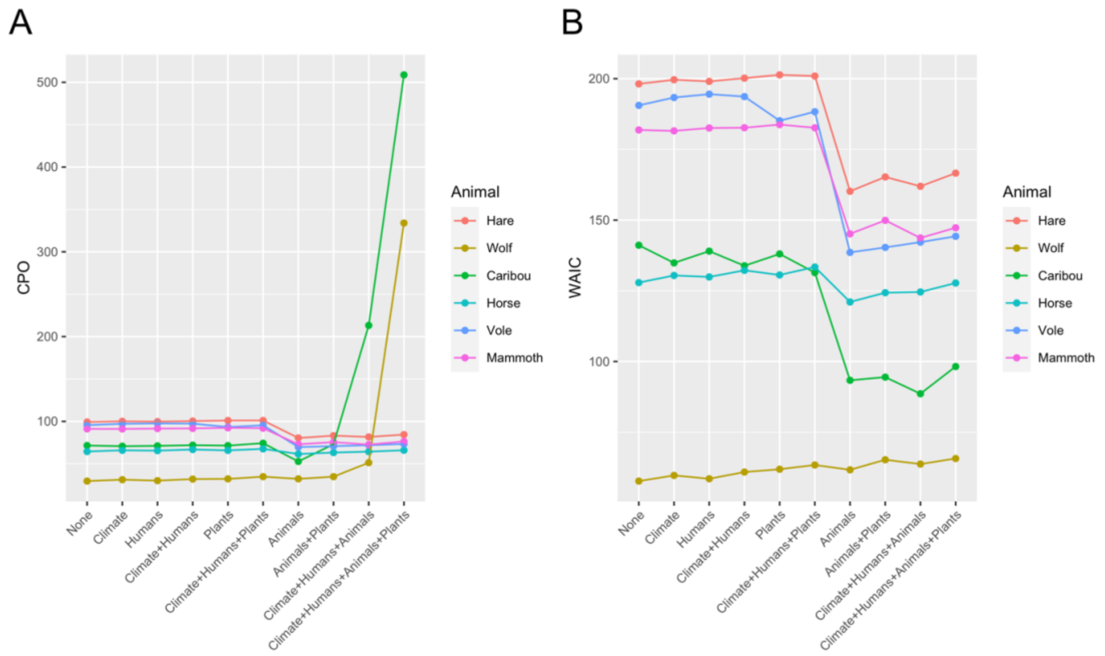

Supplementary Figure 13.4.1 | Model comparison for fitting presence/absence of animal eDNA in period P1. We computed two types of scores to compare model fit with varying numbers of covariates. Panel A: negative sum of the logarithm of the conditional

predictive ordinate for each observed site (CPO). Panel B: Watanabe-Aikaike information criterion (WAIC). In both cases, smaller scores represent better model fits.

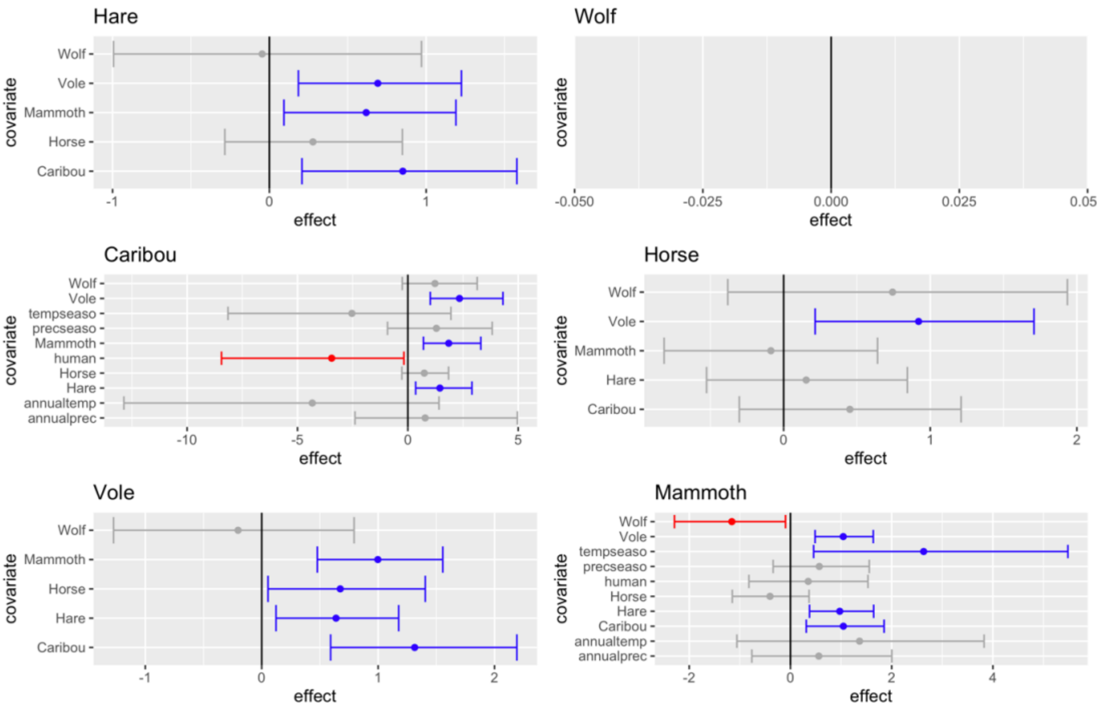

Supplementary Figure 13.4.2 | Posterior parameter estimates of covariate effects for the models explaining the presence/absence of each animal's eDNA in period P1 (n=126 samples). Only covariates included in the model with lowest WAIC are shown. The dots represent the posterior means, and the whiskers represent the posterior 2.5% and 97.5% quantiles. The colour red denotes covariate effects whose 2.5% and 97.5% quantiles are both negative, while the colour blue denotes covariate effects 2.5% and 97.5% quantiles are both positive.

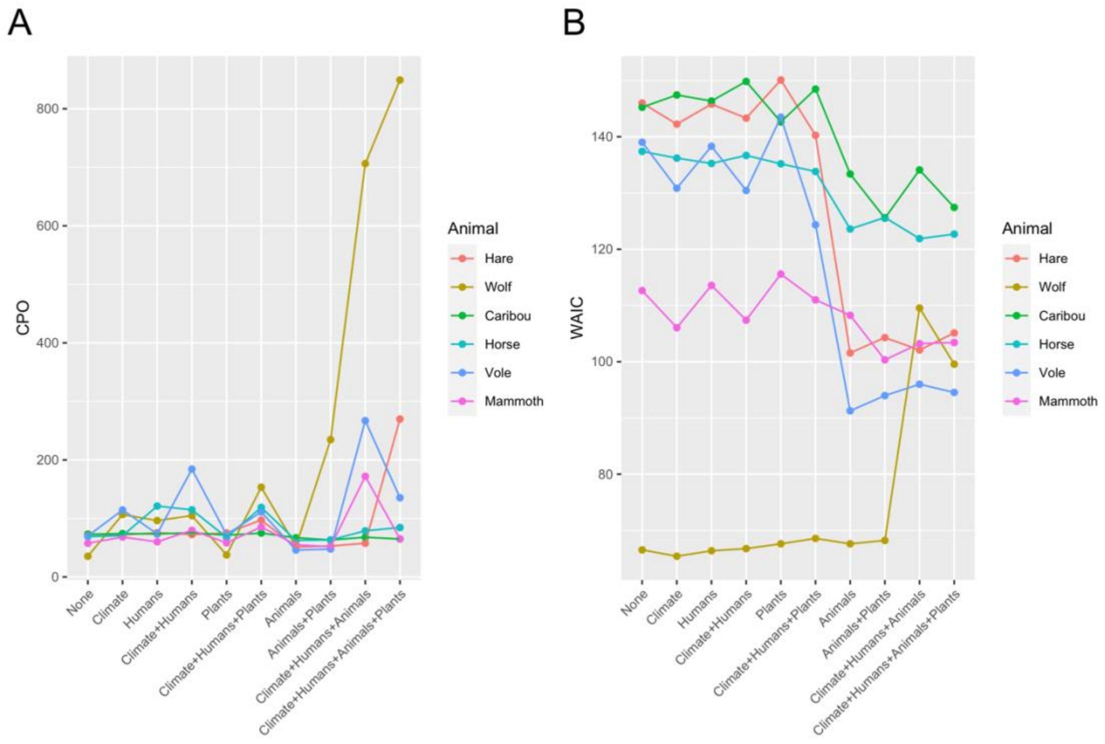

Supplementary Figure 13.4.3| Model comparison for fitting presence/absence of animal eDNA in period P2. We computed two types of scores to compare model fit with varying numbers of covariates. Panel A: negative sum of the logarithm of the conditional predictive ordinate for each observed site (CPO). Panel B: Watanabe-Aikaike information criterion (WAIC). In both cases, smaller scores represent better model fits.

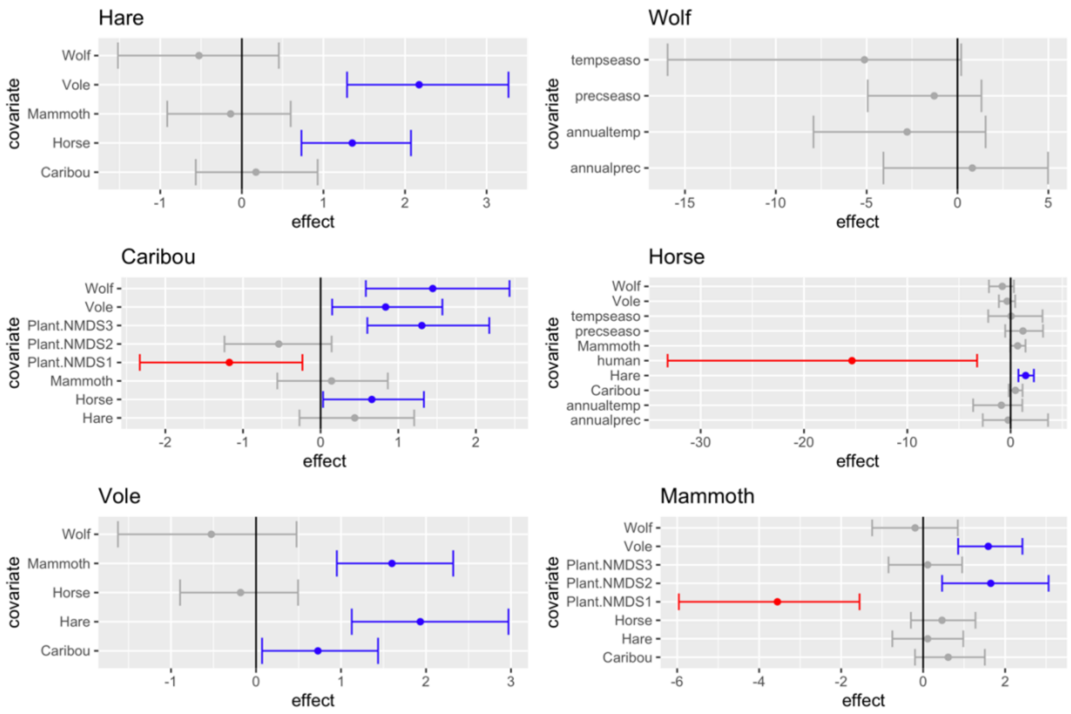

Supplementary Figure 13.4.4 | Posterior parameter estimates of covariate effects for the models explaining the presence/absence of each animal's eDNA in period P2 (n=127 samples). Only covariates included in the model with lowest WAIC are shown. The dots represent the posterior means, and the whiskers represent the posterior 2.5% and 97.5% quantiles. The colour red denotes covariate effects whose 2.5% and 97.5% quantiles are both negative, while the colour blue denotes covariate effects 2.5% and 97.5% quantiles are both positive.

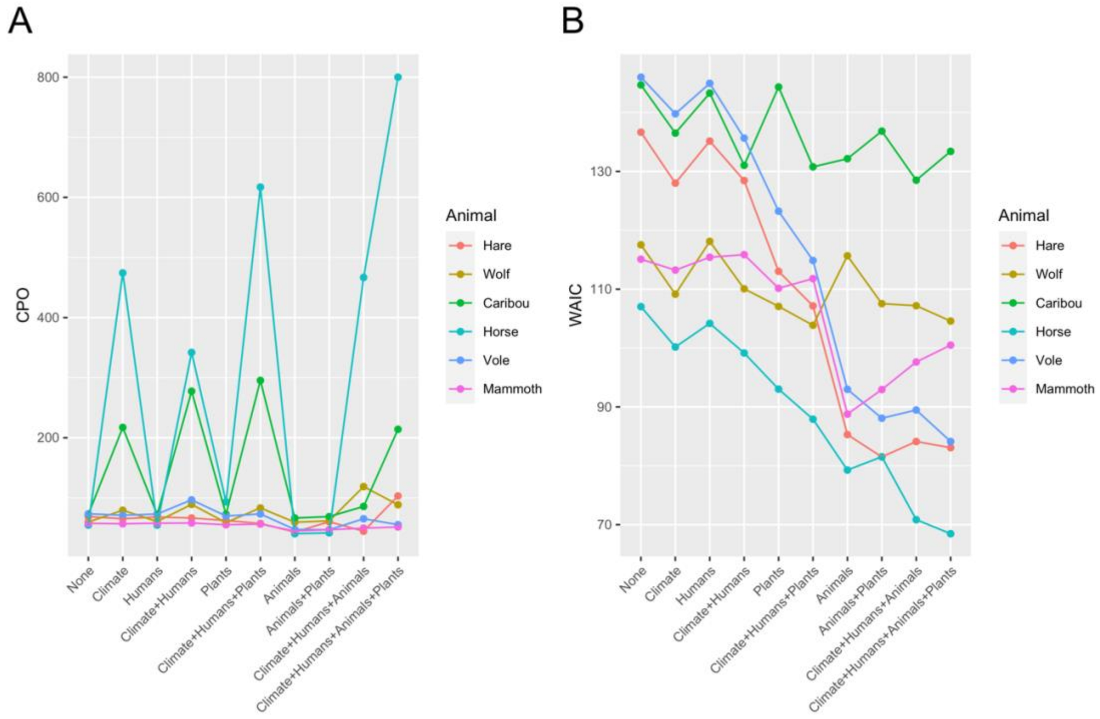

Supplementary Figure 13.4.5 | Model comparison for fitting presence/absence of animal eDNA in period P3. We computed two types of scores to compare model fit with varying numbers of covariates. Panel A: negative sum of the logarithm of the conditional predictive ordinate for each observed site (CPO). Panel B: Watanabe-Aikaike information criterion (WAIC). In both cases, smaller scores represent better model fits.

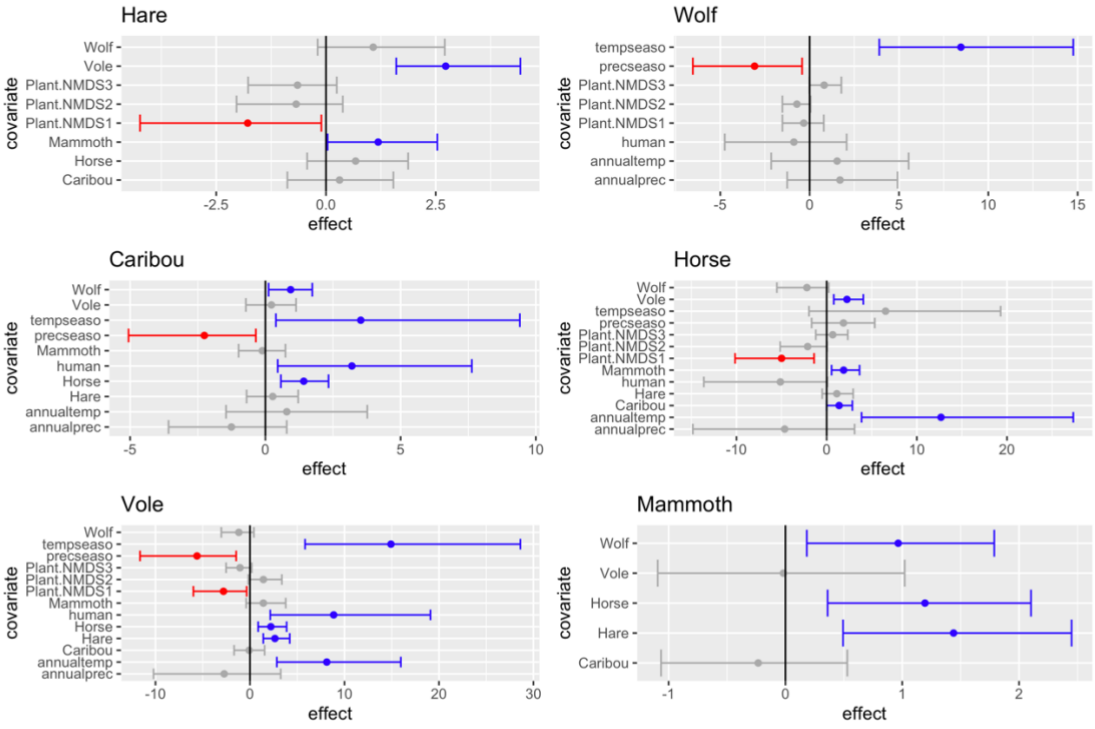

Supplementary Figure 13.4.6 | Posterior parameter estimates of covariate effects for the models explaining the presence/absence of each animal's eDNA in periods P3 (n=192 samples). Only covariates included in the model with lowest WAIC are shown. The dots represent the posterior means, and the whiskers represent the posterior 2.5% and 97.5% quantiles. The colour red denotes covariate effects whose 2.5% and 97.5% quantiles are both negative, while the colour blue denotes covariate effects 2.5% and 97.5% quantiles are both positive.

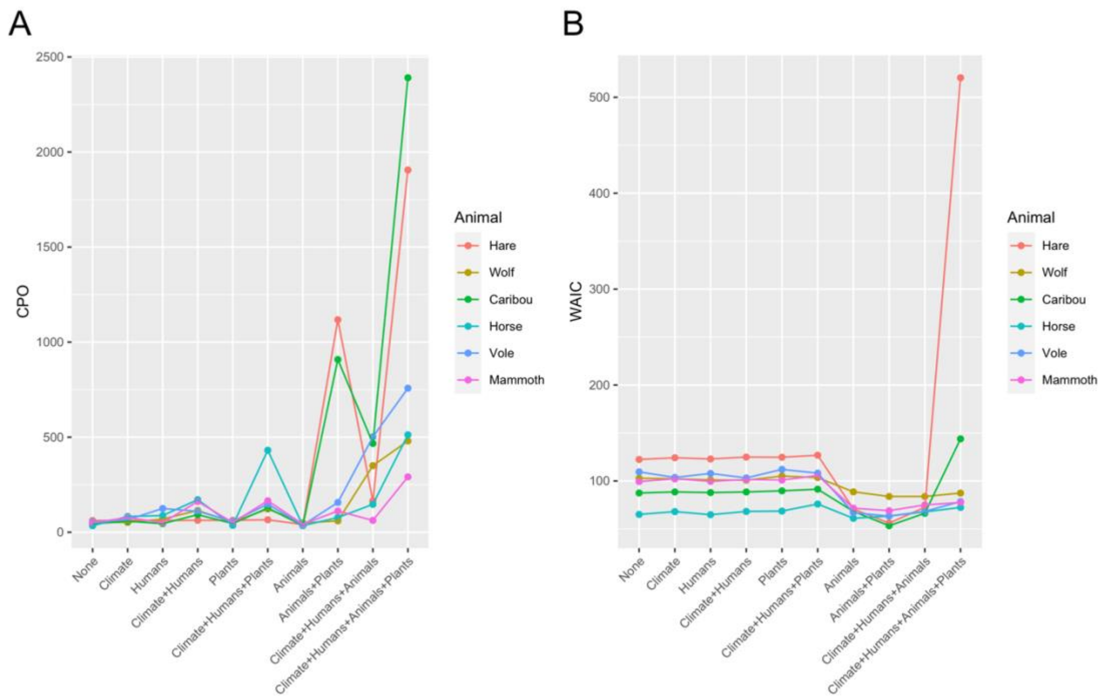

Supplementary Figure 13.4.7 | Model comparison for fitting presence/absence of animal eDNA in North America. We computed two types of scores to compare model fit with varying numbers of covariates. Panel A: negative sum of the logarithm of the conditional predictive ordinate for each observed site (CPO). Panel B: Watanabe-Aikaike information criterion (WAIC). In both cases, smaller scores represent better model fits.

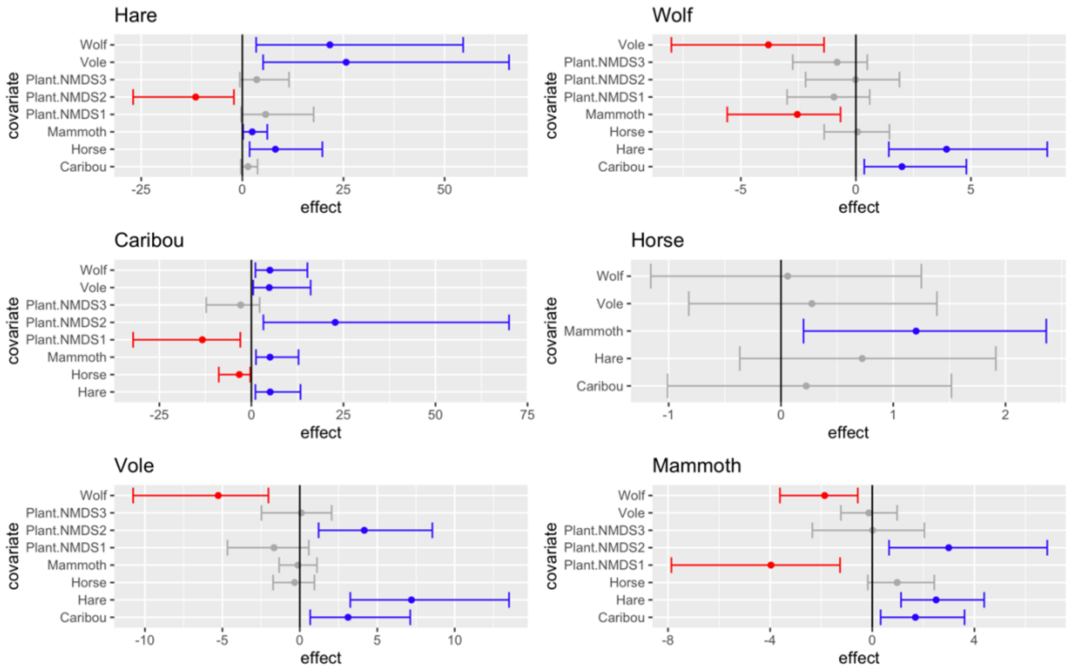

Supplementary Figure 13.4.8 | Posterior parameter estimates of covariate effects for the models explaining the presence/absence of each animal's eDNA in North America (n=105 samples). Only covariates included in the model with lowest WAIC are shown. The dots represent the posterior means, and the whiskers represent the posterior 2.5% and 97.5% quantiles. The colour red denotes covariate effects whose 2.5% and 97.5% quantiles are both negative, while the colour blue denotes covariate effects 2.5% and 97.5% quantiles are both positive.

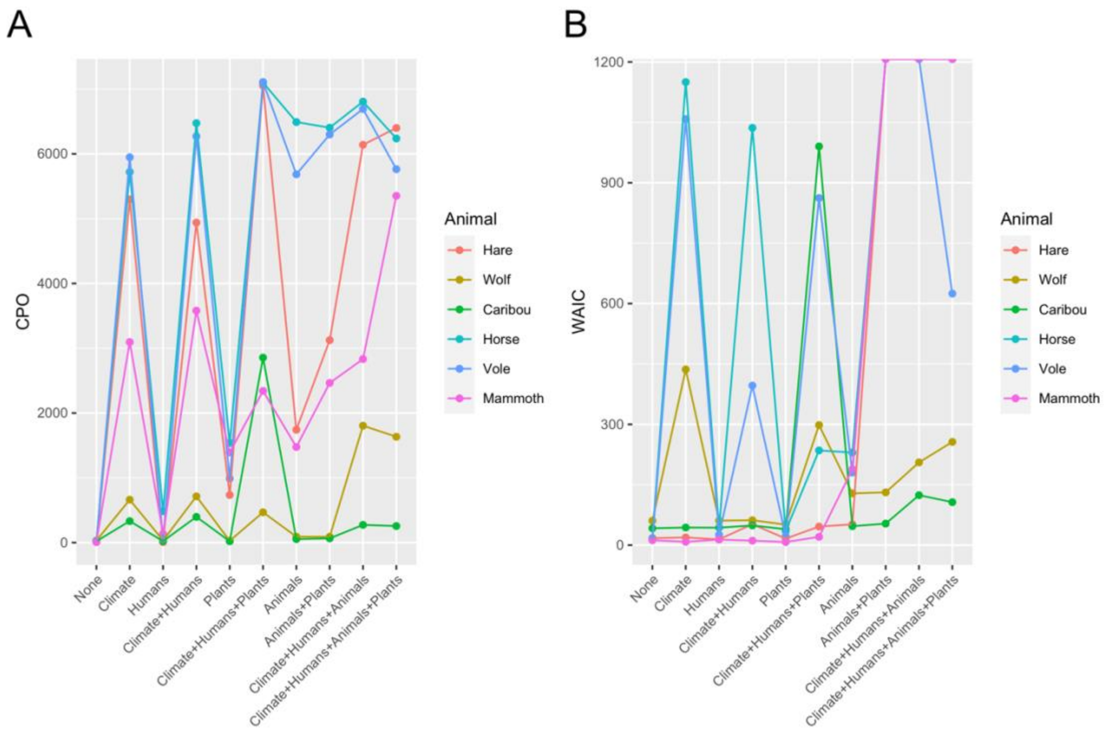

Supplementary Figure 13.4.9 | Model comparison for fitting presence/absence of animal eDNA in North Atlantic. We computed two types of scores to compare model fit with varying numbers of covariates. Panel A: negative sum of the logarithm of the conditional predictive ordinate for each observed site (CPO). Panel B: Watanabe-Aikaike information criterion (WAIC). In both cases, smaller scores represent better model fits.

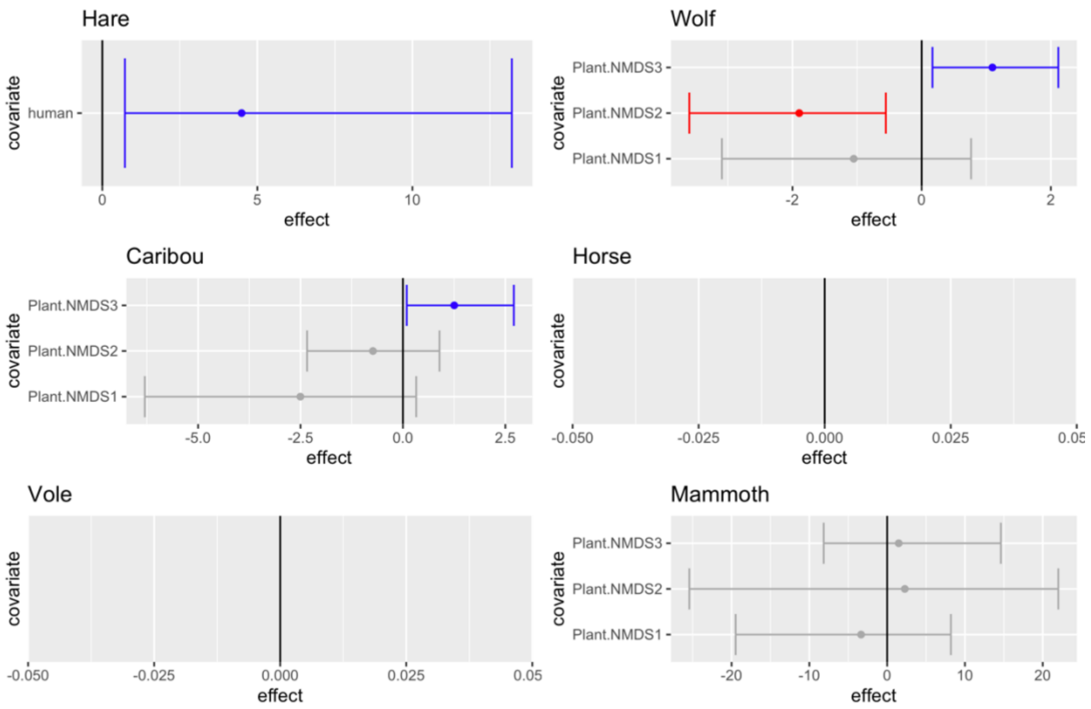

Supplementary Figure 13.4.10 | Posterior parameter estimates of covariate effects for the models explaining the presence/absence of each animal's eDNA in North Atlantic (n=104 samples). Only covariates included in the model with lowest WAIC are shown. The dots represent the posterior means, and the whiskers represent the posterior 2.5% and 97.5% quantiles. The colour red denotes covariate effects whose 2.5% and 97.5% quantiles are both negative, while the colour blue denotes covariate effects 2.5% and 97.5% quantiles are both positive.

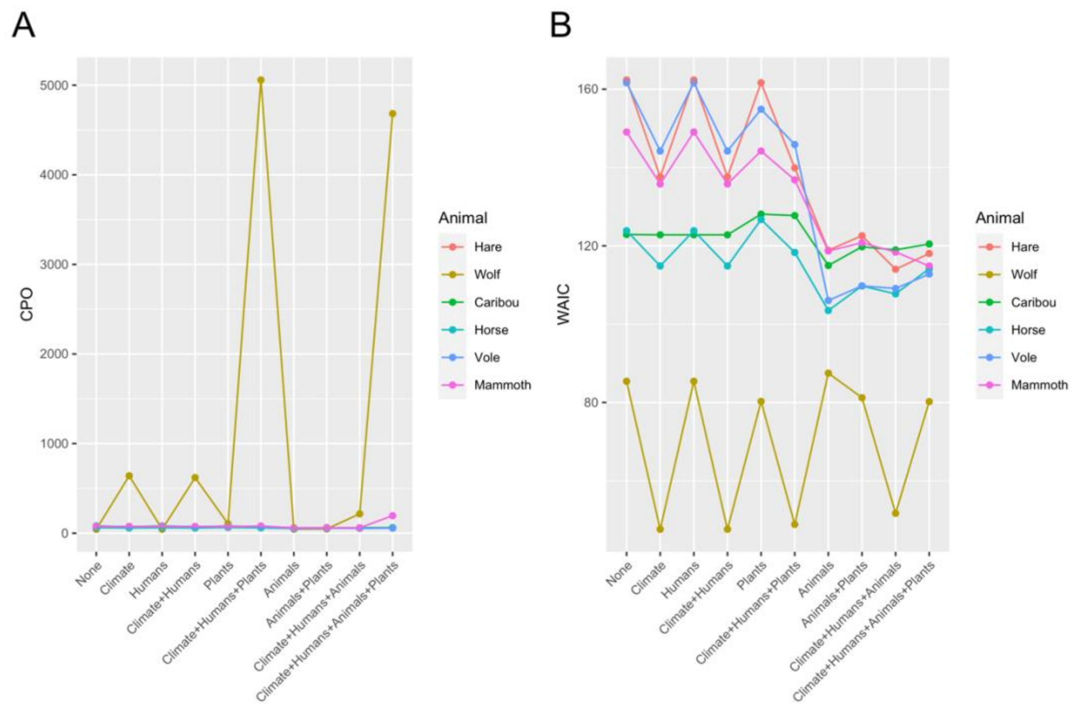

Supplementary Figure 13.4.11 | Model comparison for fitting presence/absence of animal eDNA in Northeast Siberian. We computed two types of scores to compare model fit with varying numbers of covariates. Panel A: negative sum of the logarithm of the conditional predictive ordinate for each observed site (CPO). Panel B: Watanabe-Aikaike information criterion (WAIC). In both cases, smaller scores represent better model fits.

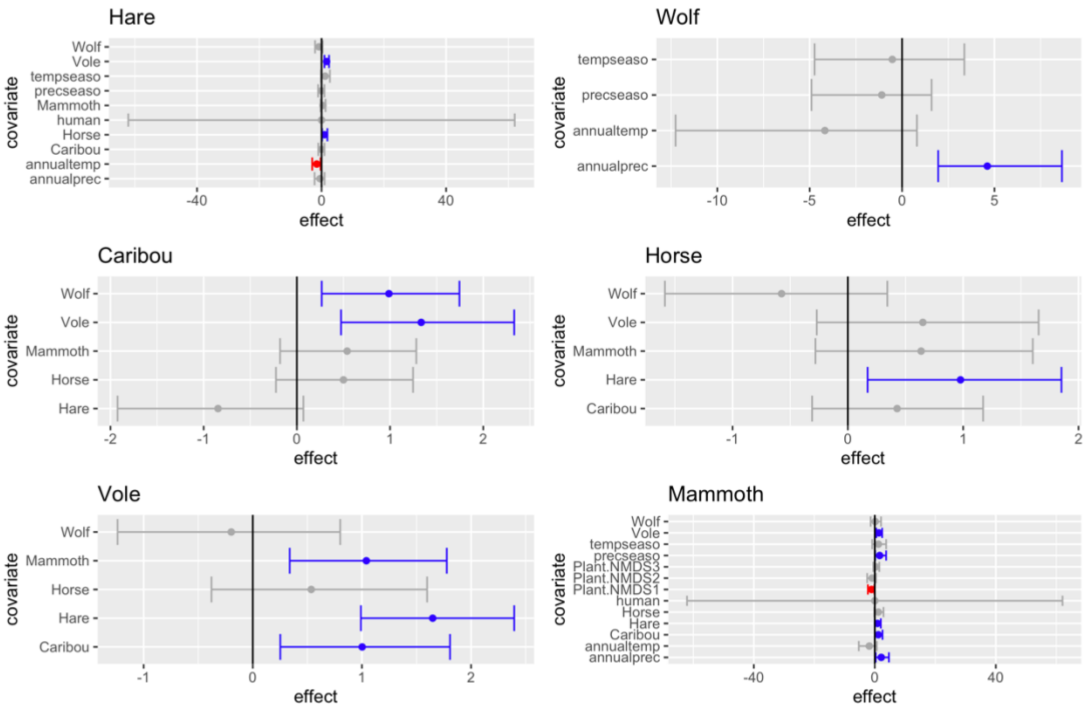

Supplementary Figure 13.4.12 | Posterior parameter estimates of covariate effects for the models explaining the presence/absence of each animal's eDNA in Northeast Siberian (n=141 samples). Only covariates included in the model with lowest WAIC are shown. The dots represent the posterior means, and the whiskers represent the posterior 2.5% and 97.5% quantiles. The colour red denotes covariate effects whose 2.5% and 97.5% quantiles are both negative, while the colour blue denotes covariate effects 2.5% and 97.5% quantiles are both positive.

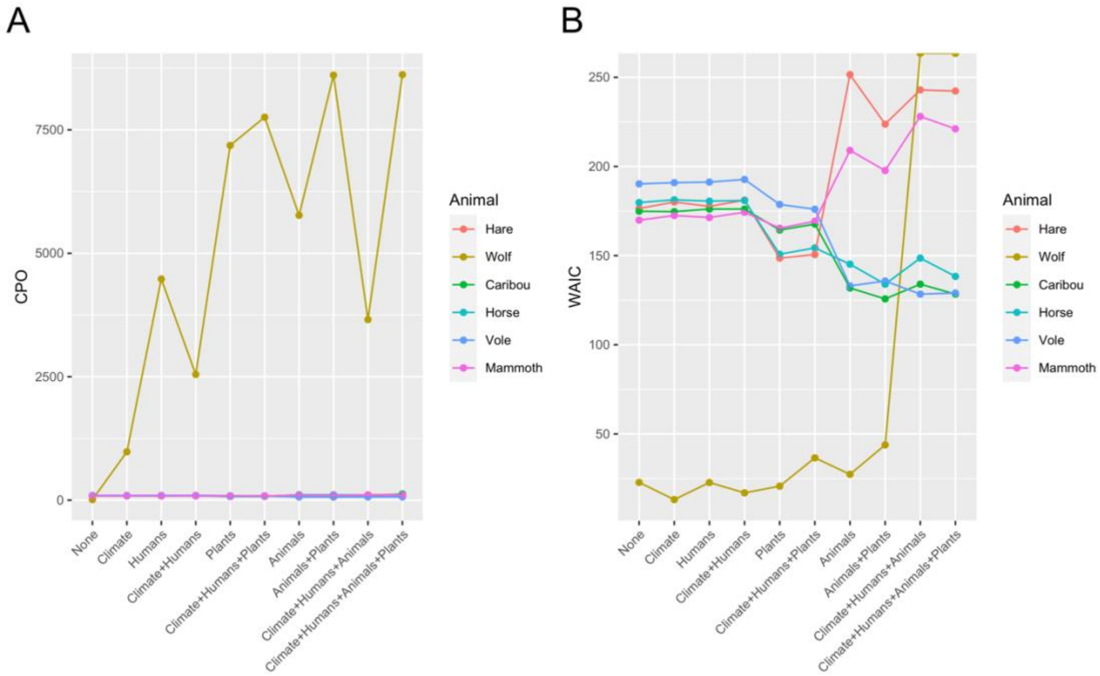

Supplementary Figure 13.4.13 | Model comparison for fitting presence/absence of animal eDNA in Northwest and central Siberian. We computed two types of scores to compare model fit with varying numbers of covariates. Panel A: negative sum of the logarithm of the conditional predictive ordinate for each observed site (CPO). Panel B: Watanabe-Aikaike information criterion (WAIC). In both cases, smaller scores represent better model fits.

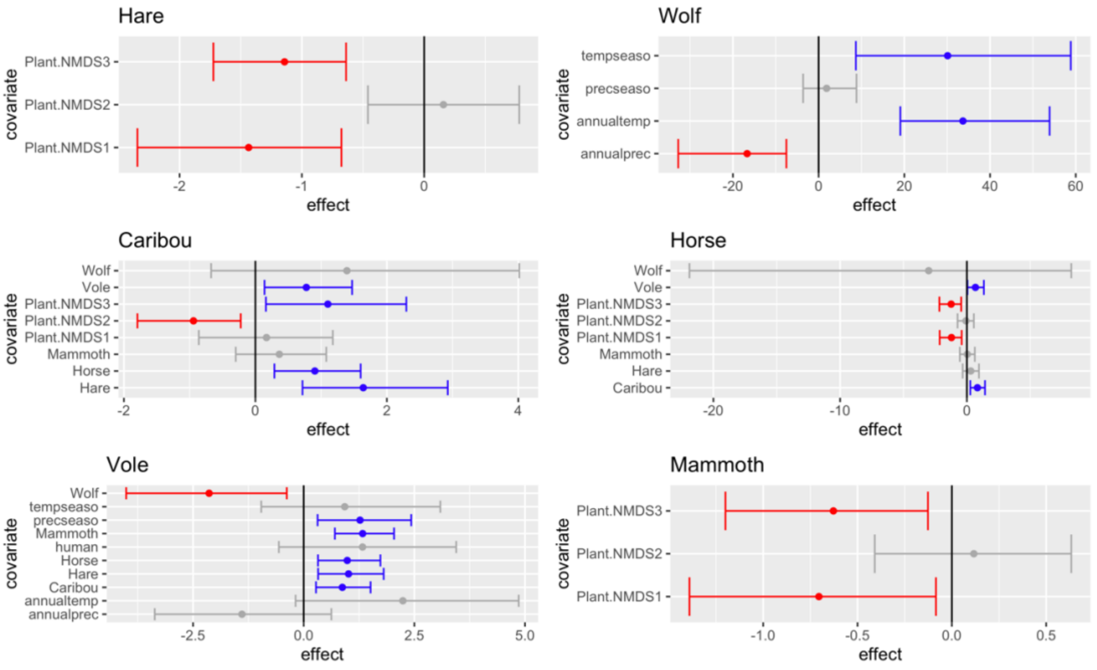

Supplementary Figure 13.4.14 | Posterior parameter estimates of covariate effects for the models explaining the presence/absence of each animal's eDNA in Northwest and central Siberian (n=145 samples). Only covariates included in the model with lowest WAIC are shown. The dots represent the posterior means, and the whiskers represent the posterior 2.5% and 97.5% quantiles. The colour red denotes covariate effects whose 2.5% and 97.5% quantiles are both negative, while the colour blue denotes covariate effects 2.5% and 97.5% quantiles are both positive.

14. Mammoth and horse mitochondrial haplotype

We applied a recently developed method (pathPhynder)[137] to assign species identified from eDNA into mitochondrial haplotypes, and successfully applied it on mammoth and horse, the best two represented animals in our data. Method and results are described in below.

14.1 Mammoth

We identified mammoth DNA in a total of 159 samples, with some samples containing thousands mammoth reads. This created the possibility to study the genetic structure of the mammoths appearing in different samples. As the mitochondrial genome reference of woolly mammoth is 16,506 bp long[138] and the whole genome is around 3.3 billion bases pairs[79], the majority of the mammoth DNA in sediment samples may be expected to have

originated from its nuclear genome. However, the mitochondrial genome has multiple copies per cell and a smaller size, and the mammoth DNA endogenous rates in sediment samples are extremely low. Therefore, the recovered coverage on mitochondria genome should be higher than that of the nuclear genome, and thus more comparable single nucleotide polymorphisms (SNPs) are available cross mitochondria genomes. The first mammoth mitochondrion was sequenced in 2006, marking it the oldest mitochondrial sequence of any species at the time[139]. Since then, over 100 partial or complete mitochondrial genomes from woolly mammoths have been published, all from fossil remains representing different regions and times. However, only 2 complete genomes have been sequenced so far. The mammoth mitochondrial tree is therefore significantly more complete and well understood than the nuclear tree. Given these 2 reasons, we focused on mammoth mitochondrial genome in this section.

Like elephants in general, mammoths first evolved in Africa, moved North into Asia and Europe, and then made their way into North America via the Bering Land Bridge. The main mammoth species include Southern mammoths (*Mammuthus meridionalis*), Steppe mammoths (*M. trogontherii*), Columbian mammoths (*M. columbi*), and woolly mammoths (*M. primigenius*). Sometimes the North American woolly mammoths are separated from woolly mammoths in the literature and known as *M. americanus*. Until recently, it was thought that these species essentially gave rise to each other, in a linear fashion in the order presented above, with the transition to the woolly mammoth occurring approximately 700,000 years ago. These findings relied primarily on fossil and morphological studies. However, this view has been challenged by progress in the field of mammoth genetics, such as in ref. [140]. The evolutionary history now appears more complicated, with possible long-term coexistence of different mammoth species and multiple hybridization events[141]. It has also been suggested that earlier mammoths species such as the Steppe mammoth migrated to North America and evolved there, resulting in a separate species, but this now seems unlikely, given recent phylogenetic studies[142].

The five currently known mammoth mitochondrial haplogroups (labeled A to E) fit within three major clades (labeled 1 to 3)[140]. In a somewhat confusing labeling scheme, clade 3 is equivalent to haplogroup B, and clade 2 is equivalent to haplogroup A. Clade 1 contains haplogroups C, D and E, although the latter two are often grouped together and labeled as 1DE. Both clades 2 and 3 are known to be older, dying out around 30,000 years ago, while

the last persisting mammoth populations fell within clade 1. Clade 1C is often called the American clade (the previously mentioned *Mammuthus Americanus*) because of its presence in the Americas. The clades diverged around 1 million years ago, with the MRCA of clade 1 estimated to be around 500 ka[140].

Mammoth DNA retrieved from sediment samples are most likely from multiple individuals of a population who inhabited the location during a certain time frame. Thus the DNA or genomes can be seen as metagenomic-genomes, containing genetic information from different individuals sharing similar genomic structures. Therefore, instead of the whole genome-based methods, here we developed a new approach to directly place the mitochondrial reads from each sample on the mammoth mitochondrial tree re-built from the published mitochondrial genomes. In the majority of cases, we achieved at least a clade-level placement, with some achieving a surprising level of depth. Our assignments are consistent with previous findings from skeletal material, but also suggest a previously unseen clade.

14.1.1 Reference tree

We downloaded 78 mitochondrial genomes (as fasta format) from NCBI for building the mammoth mitochondrial phylogenetic tree. The list of NCBI accession IDs for these 78 samples, along with their latitude, longitude, age and reference (if available) is shown in Supplementary Table 14.1.1. The publications containing the original data are ref. [138,140,143-149]. We concatenated these genomes to create a single file and standardized the sequences, by changing all bases to uppercase and replacing all instances of nucleotides that were not A, C, T, G or N into an N. Hereafter we aligned all sequences with Muscle (version 3.81)[150] following the default parameters. SNPSites[151] was then used to turn the multiple sequence alignment into a VCF file, with a total of 860 variable sites identified. In the VCF file, there were many 667 lines with asterisks in the “alt allele” field, which is intended to represent a deletion in a newer release of GATK. However, due to the nature of the data and the high frequency of the occurrence of asterisks, it is most likely that this is an artifact of missing data. The VCF was thus transformed to reflect this. Additionally, there was one triallelic site, where the third allele was a C to T in a single sample. This appears to reflect deamination, so the T was replaced with a missing data marker in this sample. The final VCF file contained had 860 SNPs.

We used the BEAST[152] software suite to build a tree based on the VCF file. Specifically, "Beauti" was first used with default parameters to produce an xml file. We then ran "BEAST" using default parameters, including Beagle. Thereafter "TreeAnnotator" was applied to call a consensus MCC (maximum clade credibility) tree in NEXUS format, which was then converted into newick format via "Figtree".

Since the VCF had a significant amount of missing data, we performed an imputation step with using the algorithm included in PathPhynder (https://github.com/ruidlpm/pathPhynder). Using the newick format tree as input, and PathPhynder subsequently used the tree in the imputation process. If a site was not imputed, we left it as missing data, and not including it in downstream analysis. The algorithm failed to fully impute a total of 188 SNPs.

Supplementary Table 14.1.1 | Published mammoth mitochondrial genomes used for building the phylogenetic tree

NCBI accession ID	Age (cal. yr BP)	Lat.	Lon.
EU153446.1	13995	69.8	169
DQ316067.1	32850	68.17	165.93
EU153445.1	35800	72.5	127.5
EU153453.1	>55200	69.79	157.7
EU153450.1	>58000	73.64	142.89
EU153451.1	>63500	73.21	143.6
EU153452.1	50200	73.64	142.67
EU153458.1	46900	62.67	142.93
EU153454.1	24740	68.6	147.06
EU153448.1	18560	71.87	140.58
EU153444.1	-	-	-
EU153456.1	18545	67.83	124.29
EU153457.1	-	-	-
AP008987.1	-	-	-
JF912200.1	41510	69.37	-154.67
EU153455.1	20620	74.15	99.59
EU153449.1	20380	73.32	105.4
EU153447.1	17125	72.09	79.35
KX027489.1	>48800	74.42	107.75
KX027490.1	27740	73.75	102
KX027491.1	42764	65.17	-147.5
KX027492.1	16789	64.83	-148

KX027495.1	12125	70.4	143.95
KX027508.1	-	68.06	-139.78
KX027534.1	17510	40.52	-89.72
KX027507.1	20550	39.82	-89.53
KX027526.1	41910	68.9	69.5
KX027531.1	37920	64.05	-139.42
KX027532.1	38600	64.05	-139.42
KX027533.1	41300	63.5	142.75
KX027536.1	10350	42.15	-78.93
KX027498.1	13985	38.88	-84.75
KX027499.1	12930	38.88	-84.75
KX027500.1	13215	38.88	-84.75
KX027501.1	13950	38.88	-84.75
KX027502.1	13860	38.88	-84.75
KX027564.1	-	63.73	-138.83
KX027565.1	>45400	67.48	-139.92
KX027566.1	-	63.83	-138.25
KX027567.1	28960	63.83	-138.25
KX027560.1	-	68.06	-139.78
KX027561.1	-	68.06	-139.78
DQ188829.2	12170	71	145
KX176757.1	40700	72.68	143.52
KX176751.1	43600	73.34	141.31
KX176755.1	42960	68.733	161.383
KX176773.1	40100	56.51	3.52
KX176793.1	-	62	58.73
KX176767.1	45700	51.08	83.03
KX176770.1	-	51.54	7.2
KX176769.1	45180	47.82	12.64
KX176768.1	-	50.92	84.78
KX176785.1	-	44.8	34.29
EU155210.1	27740	73.45	102
MG334278.1	8318	71.2489	-179.9789
MG334266.1	4643	71.2489	-179.9789
MG334279.1	7470	71.2489	-179.9789
MG334270.1	4024	71.2489	-179.9789
MG334269.1	4079	71.2489	-179.9789
MG334265.1	4726	71.2489	-179.9789
MG334274.1	7336	71.2489	-179.9789
MG334264.1	4969	71.2489	-179.9789
MG334281.1	6380	71.2489	-179.9789
MG334280.1	7194	71.2489	-179.9789

MG334276.1	7060	71.2489	-179.9789
MG334285.1	11972	74	98
MG334277.1	8491	71.2489	-179.9789
MG334267.1	4354	71.2489	-179.9789
MG334268.1	4336	71.2489	-179.9789
MG334283.1	12775	75.16	145.15
MG334273.1	16901	66.4	171
MG334272.1	14431	66.4	171
MG334275.1	14408	71.2489	-179.9789
MG334271.1	41632	71.2489	-179.9789
MG334284.1	15602	71.2489	-179.9789
YW890206.1	44828	66.76	124.12
YW890205.1	4436	71.2489	-179.9789
MF770243.1	32480	74.13	141.03

14.1.2 Phylogenetic assignment

The multiple sequence alignment did not have the same coordinates as the original reference genomes. Because we required a reference sequence for PathPhynder whose coordinates matched our multiple sequence alignment, however, we instead created a consensus sequence from the multiple sequence alignment via a Python script. We then aligned the quality-controlled reads of each eDNA sample (Section 9.1) against the two woolly mammoth genomes (Section 9.2.1) and then subtracted reads that also mapped to any other taxa following the procedures described in Section 9.6.2. All mapped reads were then extracted from the bam file and mapped against this new consensus file via BWA toolkits, requiring a quality score higher than 10. A total of 104 samples had at least one read mapped onto the mitochondrial genomes. For this subset of samples, the mean number of reads that mapped onto the mitochondrion was 8.7, and the mean read length was 72 (distributions shown in Supplementary Figure 14.1.2.1). These mapped reads were used afterwards as inputs for PathPhynder.

We first assigned SNPs to branches of the tree via PathPhynder. It reported 188 SNPs with missing data, 729 branch defining alleles, and 658 informative positions. There were 202 positions not used. At this point, we also made sure that the new consensus genome and the bam files had the same contig name. We then followed a standard PathPhynder workflow to place samples on the tree. Once SNPs had been assigned to the tree from the data, the software determined at which branches the sample matched (labelled “supporting”

or “derived” alleles) and conflicted (labelled “conflicting” or “ancestral” alleles) with these SNPs. The best path was determined by the number of supporting and conflicting alleles. Three examples are shown in Supplementary Figure 14.1.2.2 – 14.1.2.4. Sum of the supporting SNPs number along the best path was calculated as an approximation for the confidence of the placement.

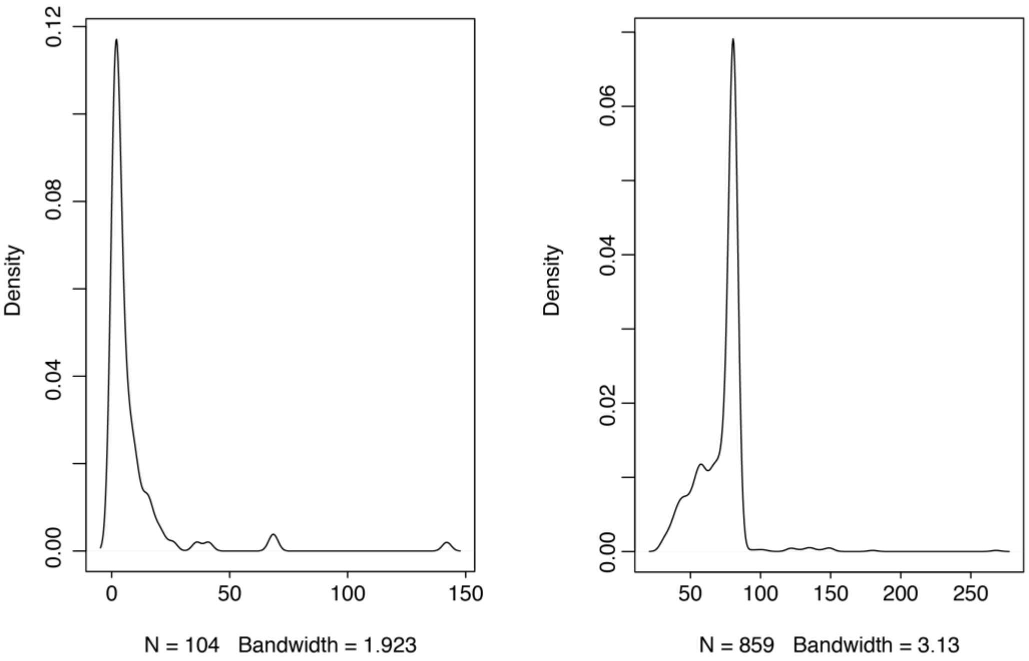

Supplementary Figure 14.1.2.1 | Read counts (how many reads per sample mapped to the mammoth mitochondrial genome) per each of the 104 samples and read length distribution for the 859 mapped reads.

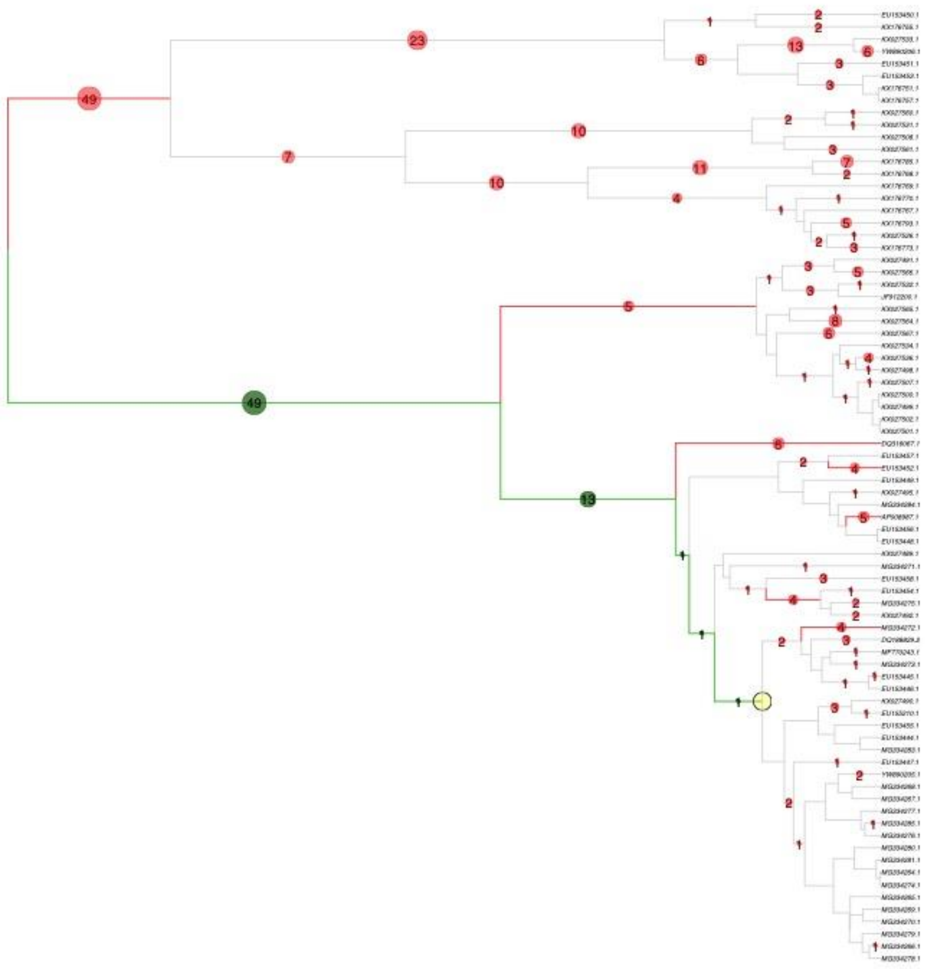

Supplementary Figure 14.1.2.2 | Mammoth phylogenetic assignment for sample ar5_18 (25.23 ka, Northeast Siberia). The green dots represent SNPs assigned to branches on the tree that are in support of placing the sample on that branch (i.e. the SNPs match), and the red dots represent those that are in conflict of placing the sample on that branch (i.e. the SNPs do not match). The best path is shown in green.

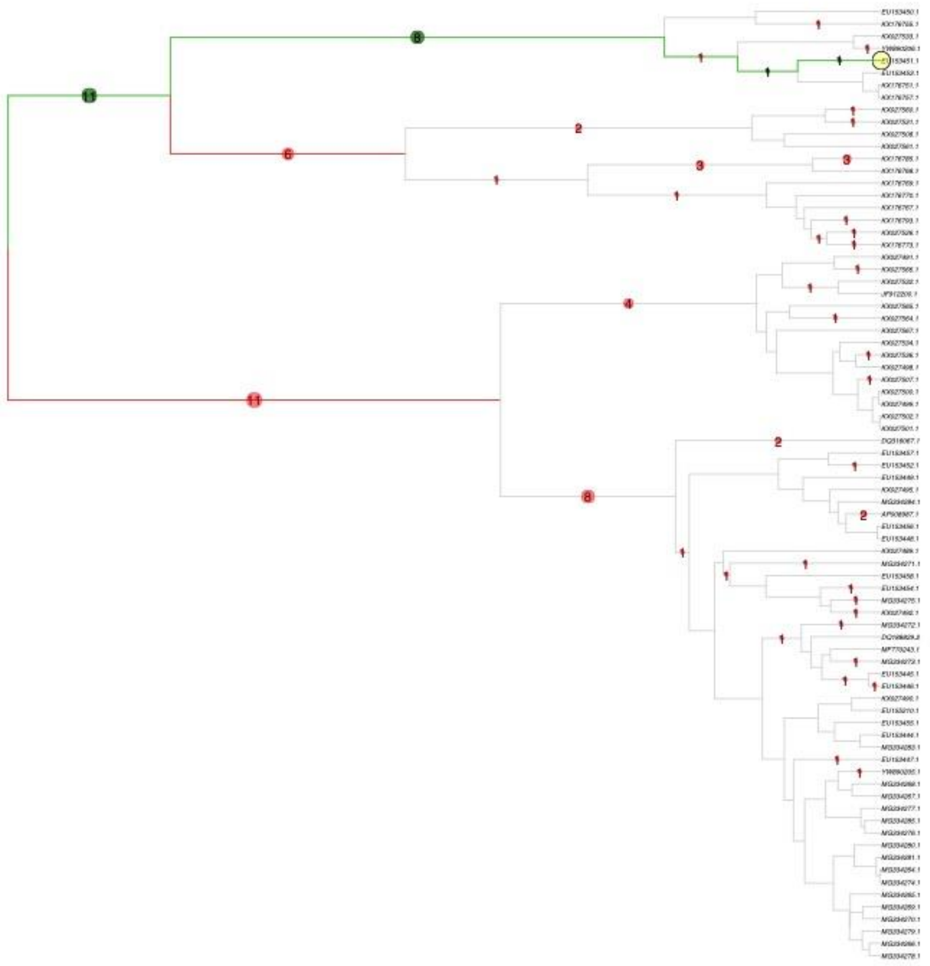

Supplementary Figure 14.1.2.3 | Mammoth phylogenetic assignment for sample cr8_39 (43.86 ka, Northeast Siberia). The green dots represent SNPs assigned to branches on the tree that are in support of placing the sample on that branch (i.e. the SNPs match), and the red dots represent those that are in conflict of placing the sample on that branch (i.e. the SNPs do not match). The best path is shown in green.

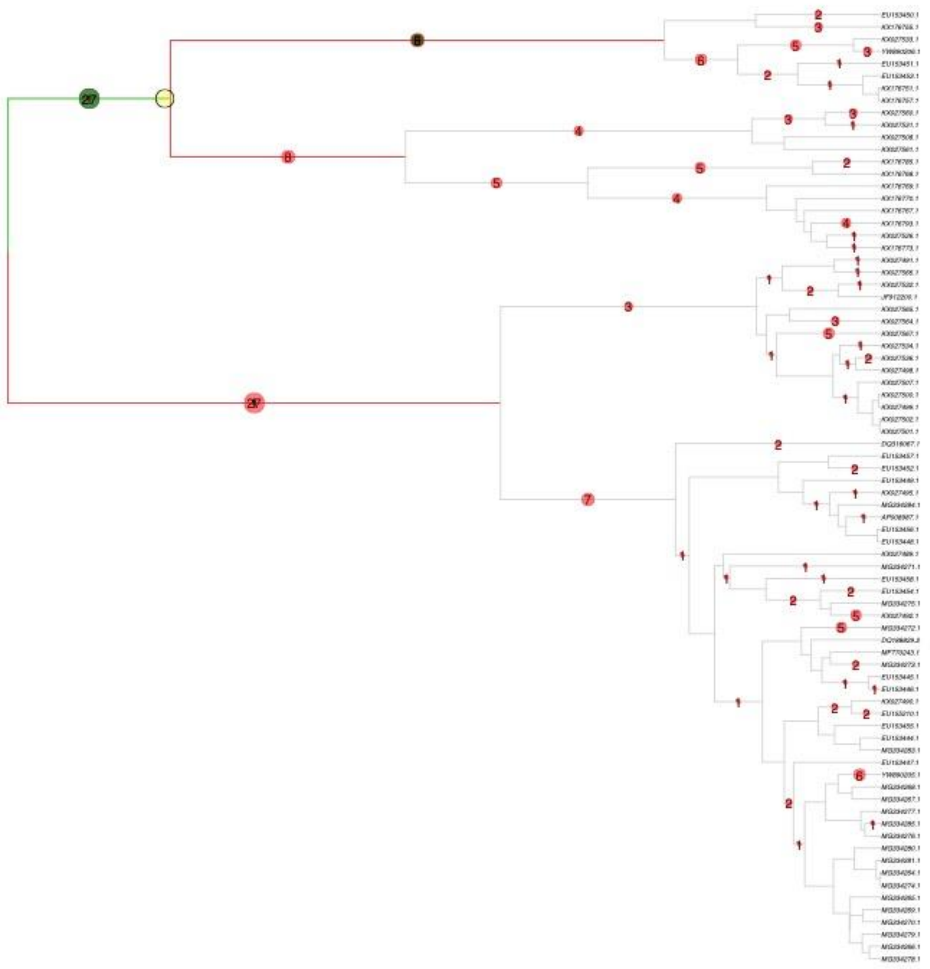

Supplementary Figure 14.1.2.4 | Mammoth phylogenetic assignment for sample cr5_11 (22.92 ka, Northeast Siberia). The green dots represent SNPs assigned to branches on the tree that are in support of placing the sample on that branch (i.e. the SNPs match), and the red dots represent those that are in conflict of placing the sample on that branch (i.e. the SNPs do not match). The best path is shown in green.

14.1.3 Result

Among the 159 eDNA samples identified with mammoth eDNA (Section 9.6.3), we were able to place a total of 79 samples on the tree at some levels, with a mean supporting value of 6.1. Supplementary Figure 14.1.3.1 shows a distribution of supports, stratified by clade. The most supported placements were in Clade 1DE, with the highest ar5_18 reaching 65 (Supplementary Figure 14.1.2.2). Most samples did not achieve a maximum depth

placement. Samples were often placed on an internal node in the tree. This is expected for because of the low coverage data which could lead to few or no matching SNPs on lower branches, but it could also result from the sample being divergent from the other samples in the tree at that internal node, which would imply that this sample represents a new clade that have not been found.

Based on our data, clade 3 is mostly in Europe and NW Siberia. Clade 2 is in Central to NE Russia. Clade 1C is scattered across America, while clade 1DE is in Central and NE Russia, but primarily along the coastline.

Eight samples were placed within Clade 2. Some had very good support, with cr8_39 even achieving maximum depth in the tree (Supplementary Figure 14.1.2.3). These results are in close congruence with the known geographical and age distributions of the clade, but also offer some new information about mammoth population dynamics and increase the known diversity of the clade. Within Clade 1C (the known American clade) there are a series of samples indicating that the previously published genomes are from the middle Americas. We successfully placed 6 sediment samples from Alaska into Clade 1C, all of which agreed well with both the known location and age of this clade. These two clades provide support to the effectiveness and the reliability of our method.

Very little is known about Clade 3, with the locations of the previously published mammoths broadly scattered and many of the ages remaining unknown. None of our samples were conclusively placed into Clade 3. The sample cr5_11 was placed at the shared root of Clades 2 and 3 with very good support. It is also the youngest mammoths (22.92 ka BP) in Clades 2 and 3, and has quite a few conflicting SNPs internal to both Clades 2 and 3 (Supplementary Figure 14.1.2.4). Although this signal could result from a sample containing both Clade 2 and Clade 3 genomes, as all other results indicate that all clade 2 and 3 mammoths died out after 30 ka BP, it is unlikely that the sample results from a region that had both two clades living there. . An more likely alternative explanation is that another clade branching off from the common ancestor of Clades 2 and 3 is represented by this sample.

We placed 14 samples from Siberia to Clade 1DE. They were scattered in regards to both age and location. Some samples were placed at maximum depth and found neighbours halfway across the world, such as sample ar5_17, indicating either a highly mobile

population, or a wide spread large mammoth population with relatively conserved mitochondrial genome.

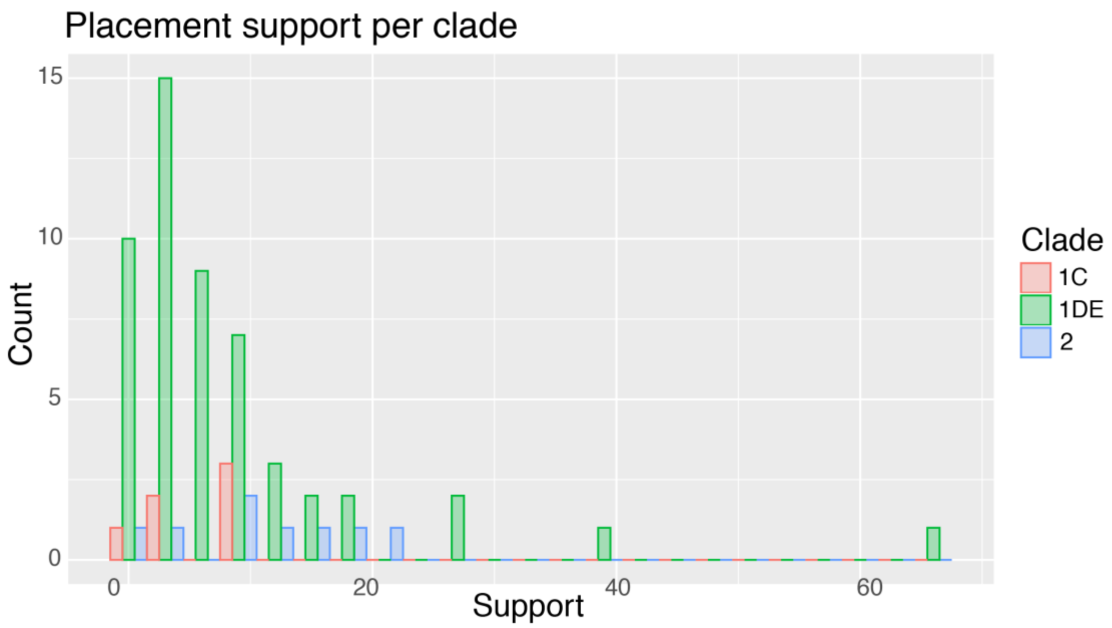

Supplementary Figure 14.1.3.1 | Mammoth placement supporting values distribution.

14.2 Horse

Current standard taxonomy depicts 2 living species of horse, including the modern domestic horse [*Equus (ferus) caballus*] and Przewalski's horse (*E. przewalski*). However, these have been suggested as representing members of a single species based on the fertility of their F1-hybrids, the recent estimates of population split times[153] and the genetic continuity found between the earliest domestic horses[154]. Multiple other equine species of debated taxonomic status have gone extinct recently (*e.g.*, *E. lambei*, *E. scotti*). Domestication of the modern horse likely began ~4000-5000 years ago in Eurasia and became widespread thereafter. Recent breeding practices have led to increased genetic influence of specific bloodlines, and therefore a substantial decline in genetic diversity[155,156].

14.2.1 Reference tree and eDNA sample assignment

The horse mitochondrial phylogeny has at least 18 different haplogroups and is not generally structured with respect to geography or age as in the case of the mammoth phylogeny[157]. In fact, in the mitochondrial phylogeny, *E. caballus*, *E. lambei*, *E. scotti* and

E. przewalski are not represented by distinct clades. It has been suggested that although both *E. lambei* and *E. scotti* appear to be morphologically distinct from caballus, they may not be genetically well-defined species. Therefore, we use the term "horse" to refer to any of these four species.

One of the oldest full genomes of any species is of a horse, dated to between 780 and 560 ka, and comes from a fossilized bone in Yukon, Canada[24]. This genome, along with a few others from the same region but with a wide range of ages, represents the most diverged outgroup in the horse mitochondrial phylogeny.

The reference panel includes a total of 818 horse mitochondrial genomes from 2 souses. (i) 437 annotated mitochondrial reference genomes collected from a bored range of referencesi[24,153,154,157-179], representing a diverse subset meant to cover the relevant regions and time periods of our eDNA samples (details supplied in Supplementary Data 9). This included 198 modern and 239 ancient samples with outgroups (*Haringtonhippus*, *Hippidion*, and *E. ovodovi*). (2) All available horse mitochondrial genomes at NCBI (n= 432, downloaded on 28th Feb. 2021).

The eDNA reads alignment was done differently from the mammoths because of the high mitochondrial diversity in horse, and that the analysis yielded very few alignments when against a single horse genome. We therefore mapped the quality-controlled eDNA reads (Section 9.1) against the whole horse mitochondrial panel and then subtracted reads that also mapped to any other taxa following the method in Section 9.6.2, resulting in 135 samples with reads mapped, with a mean reads number of 3.6 for each sample and a mean read length of 62.

We next trimmed the outgroups from the first part of the reference panel and used the remaining 403 genomes to construct a reference phylogeny. Based on this reference phylogeny, we placed our eDNA samples following the same method as for the mammoth.

14.2.2 Results

Of the 162 eDNA samples identified with horse (Section 9.6.3), we placed 43 samples to the horse mitochondrial reference. In order to extract meaningful results and better represent the information, we thinned the tree by more than half of the reference samples, taking care to leave them in if they were neighbouring a placed eDNA sample. A list of the

references contained in the thinned tree can be found in Supplementary Data 9. The final tree is shown in the Supplementary Figure 14.2.2.1.

Most of the horse references are from the late Holocene or modern times, and that very few of these are from North America. However, a single ancient North American clade is evident, and is highly diverged from the rest of the tree. The reference samples in this clade are all from a small region of Yukon, Canada[24,164], therefore referred as Yukon Clade hereafter. Surprisingly, we placed 15 samples into this clade (Supplementary Table 14.2.2), which consists of 7 reference horse genomes including the three oldest reference genomes, KT757751, KT757752, and KT757763, representing the oldest known *E. caballus* from North America, all dating from around or before 100 ka. The other 4 reference genomes in this clade include two *E. lambei*, from approximately 26 and 38 ka, and two *E. scotti* that are older than 50 ka. The Yukon Clade is divergent from the main horse clade, although it's likely that these horses were in the same region, at the same time as other horses from the main clade. The eDNA samples that placed into this clade range from 23 to 30 ka, which might suggest they represent *E. lambei* individuals, as the reference *E. lambei* samples in this clade are from this same age range.

Of the 15 eDNA sample placed into the Yukon Clade, 11 are with more than 2 supports, and 9 of the 11 are from the site GS (Goldbottom Site, Site ID 61), which is only ~10 km away from the location where two skeletons of the clade were found, further confirming the reliability of our method. A further 2 eDNA samples placed were from site QC (Quartz Creek, Site ID 56) which is also in Yukon. These potential new members of the Yukon clade indicate a long-term continuity of the lineage. None of the other placed samples had substantial support or were placed in groups, and we cannot draw meaningful conclusions from them.

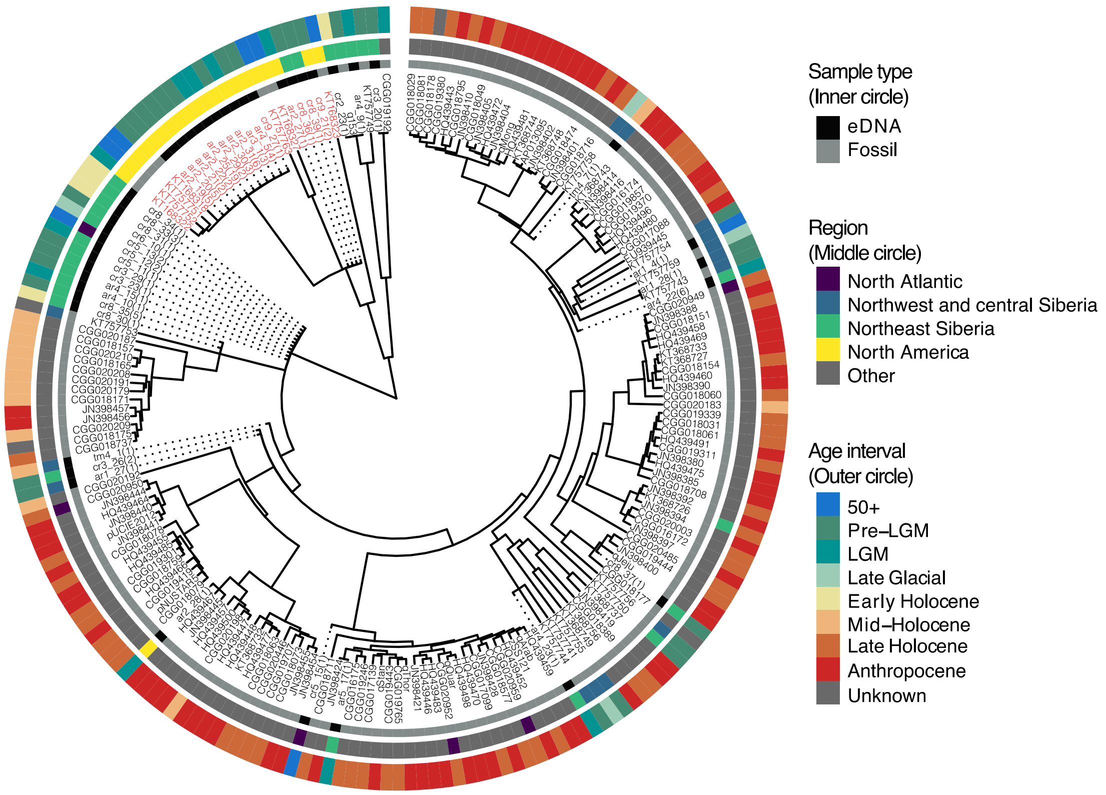

Supplementary Figure 14.2.2.1 | Horse mitochondrial phylogenetic tree. For placed eDNA samples the number of supporting SNPs is given in braces (Supplementary Section 14.2). IDs for the Yukon clade (both eDNA and fossil) are marked in red.

Supplementary Table 14.2.2 | eDNA samples placed into the Yukon clade of horse.

Sample ID	Support	Age (ka BP)	Age interval	Rigion	Site ID	Site Abbreviation
ar2_19	5	30.22	Pre-LGM	North America	61	GS
ar2_20	5	29.54	Pre-LGM	North America	61	GS
ar2_21	2	28.87	Pre-LGM	North America	61	GS
ar2_22	2	30.30	Pre-LGM	North America	61	GS
ar2_25	19	27.11	Pre-LGM	North America	61	GS
ar2_27	1	24.52	LGM	North America	61	GS
ar2_29	2	24.57	LGM	North America	61	GS
ar2_30	3	23.21	LGM	North America	61	GS
ar2_31	6	27.27	Pre-LGM	North America	61	GS
ar3_1	3	23.50	LGM	North America	61	GS
ar4_5	4	29.84	Pre-LGM	North America	56	QC

cr8_36	1	40.87	Pre-LGM	Northeast Siberia	41	PP
cr8_39	1	43.86	Pre-LGM	Northeast Siberia	41	PP
cr9_21	2	30.35	Pre-LGM	North America	56	QC
cr9_27	1	29.07	Pre-LGM	North America	56	QC

15. Code and script archive

All code and script used in this study can be found at the GitHub repository: https://github.com/wyc661217/Arctic_eDNA_2021.

References

1 Paus, A., Velle, G. & Berge, J. The Lateglacial and early Holocene vegetation and environment in the Dovre mountains, central Norway, as signalled in two Lateglacial nunatak lakes. *Quaternary Sci Rev* **30**, 1780-1796, doi:10.1016/j.quascirev.2011.04.010 (2011).

2 Parducci, L. *et al.* Glacial survival of boreal trees in northern Scandinavia. *Science* **335**, 1083-1086, doi:10.1126/science.1216043 (2012).

3 Yoccoz, N. G. *et al.* DNA from soil mirrors plant taxonomic and growth form diversity. *Molecular Ecology* **21**, 3647-3655, doi:10.1111/j.1365-294X.2012.05545.x (2012).

4 Willerslev, E. *et al.* Diverse plant and animal genetic records from Holocene and Pleistocene sediments. *Science* **300**, 791-795, doi:10.1126/science.1084114 (2003).

5 Svendsen, J. I. *et al.* Geo-archaeological investigations of Palaeolithic sites along the Ural Mountains - On the northern presence of humans during the last Ice Age. *Quaternary Sci Rev* **29**, 3138-3156, doi:10.1016/j.quascirev.2010.06.043 (2010).

6 Astakhov, V. I. & Mangerud, J. The age of the Karginsky interglacial strata on the lower Yenisei. *Dokl Earth Sci* **403**, 673-676 (2005).

7 Astakhov, V. & Mangerud, J. The geochronometric age of Late Pleistocene terraces on the lower Yenisei. *Dokl Earth Sci* **416**, 1022-1026, doi:10.1134/S1028334x07070094 (2007).

8 Astakhov, V. I. & Isayeva, L. L. The Ice Hill - an Example of Retarded Deglaciation in Siberia. *Quaternary Sci Rev* **7**, 29-40, doi:Doi 10.1016/0277-3791(88)90091-1 (1988).

9 Möller, P. *et al.* Data set on sedimentology, palaeoecology and chronology of Middle to Late Pleistocene deposits on the Taimyr Peninsula, Arctic Russia. *Data in Brief*, doi:10.1016/j.dib.2019.104267 (2019).

10 Möller, P. *et al.* Glacial history and palaeo-environmental change of southern Taimyr Peninsula, Arctic Russia, during the Middle and Late Pleistocene. *Earth-Science Reviews* **196**, doi:10.1016/j.earscirev.2019.04.004 (2019).

11 Jørgensen, T. *et al.* A comparative study of ancient sedimentary DNA, pollen and macrofossils from permafrost sediments of northern Siberia reveals long-

term vegetational stability. *Molecular Ecology* **21**, 1989-2003, doi:10.1111/j.1365-294X.2011.05287.x (2012).

12 Möller, P., Alexanderson, H., Funder, S. & Hjort, C. The Taimyr Peninsula and the Severnaya Zemlya archipelago, Arctic Russia: a synthesis of glacial history and palaeo-environmental change during the Last Glacial cycle (MIS 5e–2). *Quaternary Sci Rev* **107**, 149-181, doi:10.1016/j.quascirev.2014.10.018 (2015).

13 Möller, P., Bolshiyanov, D. Y. & Bergsten, H. Weichselian geology and palaeoenvironmental history of the central Taymyr Peninsula, Siberia, indicating no glaciation during the last global glacial maximum. *Boreas* **28**, 92-114, doi:10.1111/j.1502-3885.1999.tb00208.x (1999).

14 Willerslev, E. *et al.* Fifty thousand years of Arctic vegetation and megafaunal diet. *Nature* **506**, 47-51, doi:10.1038/nature12921 (2014).

15 Cherezova, A. A. *et al.* Lateglacial and Holocene palaeoenvironments on Bolshevik Island (Severnaya Zemlya), Russian High Arctic. *Boreas* **49**, 375-388, doi:10.1111/bor.12428 (2020).

16 Arnold, L. J. *et al.* Paper II - Dirt, dates and DNA: OSL and radiocarbon chronologies of perennially frozen sediments in Siberia, and their implications for sedimentary ancient DNA studies. *Boreas* **40**, 417-445, doi:10.1111/j.1502-3885.2010.00181.x (2011).

17 Pisaric, M. F. J., MacDonald, G. M., Velichko, A. A. & Cwynar, L. C. The Lateglacial and Postglacial vegetation history of the northwestern limits of Beringia, based on pollen, stomate and tree stump evidence. *Quaternary Sci Rev* **20**, 235-245, doi:Doi 10.1016/S0277-3791(00)00120-7 (2001).

18 Murton, J. B. *et al.* Palaeoenvironmental Interpretation of Yedoma Silt (Ice Complex) Deposition as Cold-Climate Loess, Duvanny Yar, Northeast Siberia. *Permafrost and Periglacial Processes* **26**, 208-288, doi:10.1002/ppp.1843 (2015).

19 Kuzmina, S. A. *et al.* The late Pleistocene environment of the Eastern West Beringia based on the principal section at the Main River, Chukotka. *Quaternary Sci Rev* **30**, 2091-2106, doi:10.1016/j.quascirev.2010.03.019 (2011).

20 Haile, J. *et al.* Ancient DNA reveals late survival of mammoth and horse in interior Alaska. *Proc Natl Acad Sci U S A* **106**, 22352-22357, doi:10.1073/pnas.0912510106 (2009).

21 Westgate, J. A. *et al.* Changing ideas on the identity and stratigraphic significance of the Sheep Creek tephra beds in Alaska and the Yukon Territory, northwestern North America. *Quatern Int* **178**, 183-209, doi:10.1016/j.quaint.2007.03.009 (2008).

22 Demuro, M., Arnold, L. J., Froese, D. G. & Roberts, R. G. OSL dating of loess deposits bracketing Sheep Creek tephra beds, northwest Canada: Dim and problematic single-grain OSL characteristics and their effect on multi-grain age estimates. *Quaternary Geochronology* **15**, 67-87, doi:10.1016/j.quageo.2012.11.003 (2013).

23 Froese, D. G., Westgate, J. A., Reyes, A. V., Enkin, R. J. & Preece, S. J. Ancient permafrost and a future, warmer Arctic. *Science* **321**, 1648, doi:10.1126/science.1157525 (2008).

24 Orlando, L. *et al.* Recalibrating Equus evolution using the genome sequence of an early Middle Pleistocene horse. *Nature* **499**, 74-78, doi:10.1038/nature12323 (2013).
25 Froese, D. G., Zazula, G. D. & Reyes, A. V. Seasonality of the late Pleistocene Dawson tephra and exceptional preservation of a buried riparian surface in central Yukon Territory, Canada. *Quaternary Sci Rev* **25**, 1542-1551, doi:10.1016/j.quascirev.2006.01.028 (2006).
26 Briner, J. P. *et al.* A multi-proxy lacustrine record of Holocene climate change on northeastern Baffin Island, Arctic Canada. *Quaternary Res* **65**, 431-442, doi:10.1016/j.yqres.2005.10.005 (2006).
27 Pedersen, M. W. *et al.* A comparative study of ancient environmental DNA to pollen and macrofossils from lake sediments reveals taxonomic overlap and additional plant taxa. *Quaternary Sci Rev* **75**, 161-168, doi:10.1016/j.quascirev.2013.06.006 (2013).
28 Landvik, J. Y. *et al.* Northwest Svalbard during the last glaciation: Ice-free areas existed. *Geology* **31**, 905-908, doi:Doi 10.1130/G19703.1 (2003).
29 Charman, D. J. *et al.* Climate-related changes in peatland carbon accumulation during the last millennium. *Biogeosciences* **10**, 929-944, doi:10.5194/bg-10-929-2013 (2013).
30 Edwards, M. E. *et al.* Metabarcoding of modern soil DNA gives a highly local vegetation signal in Svalbard tundra. *Holocene* **28**, 2006-2016, doi:10.1177/0959683618798095 (2018).
31 Christner, B. C., Mikucki, J. A., Foreman, C. M., Denson, J. & Priscu, J. C. Glacial ice cores: A model system for developing extraterrestrial decontamination protocols. *Icarus* **174**, 572-584, doi:10.1016/j.icarus.2004.10.027 (2005).
32 Willerslev, E. *et al.* Ancient biomolecules from deep ice cores reveal a forested southern Greenland. *Science* **317**, 111-114, doi:10.1126/science.1141758 (2007).
33 Pedersen, M. W. *et al.* Postglacial viability and colonization in North America's ice-free corridor. *Nature* **537**, 45-49, doi:10.1038/nature19085 (2016).
34 Lewis, S. L. & Maslin, M. A. Defining the anthropocene. *Nature* **519**, 171-180, doi:10.1038/nature14258 (2015).
35 Clark, P. U. *et al.* The Last Glacial Maximum. *Science* **325**, 710-714, doi:10.1126/science.1172873 (2009).
36 Gradstein, F. M., Ogg, J. G., Schmitz, M. D. & Ogg, G. M. *Geologic Time Scale 2020*. 1217-1255 (Elsevier, 2020).
37 Blaauw, M. & Christen, J. A. Flexible paleoclimate age-depth models using an autoregressive gamma process. *Bayesian Analysis* **6**, 457-474, doi:10.1214/11-ba618 (2011).
38 Haslett, J. & Parnell, A. A simple monotone process with application to radiocarbon-dated depth chronologies. *Journal of the Royal Statistical Society: Series C (Applied Statistics)* **57**, 399-418, doi:10.1111/j.1467-9876.2008.00623.x (2008).
39 Reimer, P. J. *et al.* The IntCal20 Northern Hemisphere Radiocarbon Age Calibration Curve (0–55 cal kBP). *Radiocarbon* **62**, 725-757, doi:10.1017/rdc.2020.41 (2020).
40 Blaauw, M. *et al.* *rbacon: Age-Depth Modelling using Bayesian Statistics*, <https://cran.r-project.org/web/packages/rbacon/index.html> (2021).

41 Willerslev, E., Hansen, A. J. & Poinar, H. N. Isolation of nucleic acids and cultures from fossil ice and permafrost. *Trends in Ecology & Evolution* **19**, 141-147, doi:10.1016/j.tree.2003.11.010 (2004).
42 Lydolph, M. C. *et al.* Beringian paleoecology inferred from permafrost-preserved fungal DNA. *Appl Environ Microb* **71**, 1012-1017, doi:10.1128/AEM.71.2.1012-1017.2005 (2005).
43 Willerslev, E. & Cooper, A. Ancient DNA. *Proceedings of the Royal Society B: Biological Sciences* **272**, 3-16, doi:10.1098/rspb.2004.2813 (2005).
44 Pedersen, M. W. *et al.* Ancient and modern environmental DNA. *Philosophical Transactions of the Royal Society B: Biological Sciences* **370**, 20130383, doi:10.1098/rstb.2013.0383 (2015).
45 Thomsen, P. F. & Willerslev, E. Environmental DNA – An emerging tool in conservation for monitoring past and present biodiversity. *Biological Conservation* **183**, 4-18, doi:10.1016/j.biocon.2014.11.019 (2015).
46 Gilbert, M. T. P. *et al.* DNA from pre-Clovis human coprolites in Oregon, North America. *Science* **320**, 786-789, doi:10.1126/science.1154116 (2008).
47 Hebsgaard, M. B. *et al.* 'The Farm Beneath the Sand' - an archaeological case study on ancient 'dirt' DNA. *Antiquity* **83**, 430-444, doi:Doi 10.1017/S0003598x00098537 (2009).
48 Crecchio, C. & Stotzky, G. Binding of DNA on humic acids: Effect on transformation of Bacillus subtilis and resistance to DNase. *Soil Biology and Biochemistry* **30**, 1061-1067, doi:10.1016/s0038-0717(97)00248-4 (1998).
49 Blum, S. A. E., Lorenz, M. G. & Wackernagel, W. Mechanism of retarded DNA degradation and prokaryotic origin of DNases in nonsterile soils. *Syst Appl Microbiol* **20**, 513-521, doi:Doi 10.1016/S0723-2020(97)80021-5 (1997).
50 Nielsen, K. M., Johnsen, P. J., Bensasson, D. & Daffonchio, D. Release and persistence of extracellular DNA in the environment. *Environmental Biosafety Research* **6**, 37-53, doi:10.1051/ebr:2007031 (2007).
51 Parducci, L. *et al.* Ancient plant DNA in lake sediments. *New Phytol* **214**, 924-942, doi:10.1111/nph.14470 (2017).
52 Haile, J. *et al.* Ancient DNA chronology within sediment deposits: are paleobiological reconstructions possible and is DNA leaching a factor? *Molecular Biology and Evolution* **24**, 982-989, doi:10.1093/molbev/msm016 (2007).
53 Sjogren, P. *et al.* Lake sedimentary DNA accurately records 20(th) Century introductions of exotic conifers in Scotland. *New Phytol* **213**, 929-941, doi:10.1111/nph.14199 (2017).
54 Leonard, J. A. *et al.* Animal DNA in PCR reagents plagues ancient DNA research. *J Archaeol Sci* **34**, 1361-1366, doi:10.1016/j.jas.2006.10.023 (2007).
55 Smith, O. *et al.* Sedimentary DNA from a submerged site reveals wheat in the British Isles 8000 years ago. *Science* **347**, 998-1001, doi:10.1126/science.1261278 (2015).
56 Rohland, N. & Hofreiter, M. Comparison and optimization of ancient DNA extraction. *Biotechniques* **42**, 343-352, doi:10.2144/000112383 (2007).
57 Seersholm, F. V. *et al.* DNA evidence of bowhead whale exploitation by Greenlandic Paleo-Inuit 4,000 years ago. *Nat Commun* **7**, 13389, doi:10.1038/ncomms13389 (2016).

58 Giguet-Covex, C. *et al.* Long livestock farming history and human landscape shaping revealed by lake sediment DNA. *Nat Commun* **5**, 3211, doi:10.1038/ncomms4211 (2014).
59 Slon, V. *et al.* Neandertal and Denisovan DNA from Pleistocene sediments. *Science* **356**, 605-608, doi:10.1126/science.aam9695 (2017).
60 Lorenz, M. G. & Wackernagel, W. Adsorption of DNA to sand and variable degradation rates of adsorbed DNA. *Appl Environ Microb* **53**, 2948-2952 (1987).
61 Norén, L., Hedell, R., Ansell, R. & Hedman, J. Purification of crime scene DNA extracts using centrifugal filter devices. *Investigative Genetics* **4**, doi:10.1186/2041-2223-4-8 (2013).
62 Doran, A. E. & Foran, D. R. Assessment and mitigation of DNA loss utilizing centrifugal filtration devices. *Forensic Sci Int-Gen* **13**, 187-190, doi:10.1016/j.fsigen.2014.08.001 (2014).
63 Garvin, A. M. & Fritsch, A. Purifying and Concentrating Genomic DNA from Mock Forensic Samples Using Millipore Amicon Filters. *J Forensic Sci* **58**, S173-S175, doi:10.1111/1556-4029.12002 (2013).
64 King, C., Debruyne, R., Kuch, M., Schwarz, C. & Poinar, H. A quantitative approach to detect and overcome PCR inhibition in ancient DNA extracts. *Biotechniques* **47**, 941-949, doi:10.2144/000113244 (2009).
65 Cappellini, E. *et al.* Ancient Biomolecules and Evolutionary Inference. *Annu Rev Biochem* **87**, 1029-1060, doi:10.1146/annurev-biochem-062917-012002 (2018).
66 Schwarz, C. *et al.* New insights from old bones: DNA preservation and degradation in permafrost preserved mammoth remains. *Nucleic Acids Research* **37**, 3215-3229, doi:10.1093/nar/gkp159 (2009).
67 Meyer, M. & Kircher, M. Illumina sequencing library preparation for highly multiplexed target capture and sequencing. *Cold Spring Harbor Protocols* **2010**, pdb prot5448, doi:10.1101/pdb.prot5448 (2010).
68 Alsos, I. G. *et al.* The Treasure Vault Can be Opened: Large-Scale Genome Skimming Works Well Using Herbarium and Silica Gel Dried Material. *Plants* **9**, doi:10.3390/plants9040432 (2020).
69 Langmead, B. & Salzberg, S. L. Fast gapped-read alignment with Bowtie 2. *Nature Methods* **9**, 357-359, doi:10.1038/nmeth.1923 (2012).
70 Li, D., Liu, C. M., Luo, R., Sadakane, K. & Lam, T. W. MEGAHIT: an ultra-fast single-node solution for large and complex metagenomics assembly via succinct de Bruijn graph. *Bioinformatics* **31**, 1674-1676, doi:10.1093/bioinformatics/btv033 (2015).
71 Luo, R. *et al.* SOAPdenovo2: an empirically improved memory-efficient short-read de novo assembler. *GigaScience* **1**, doi:10.1186/2047-217x-1-18 (2012).
72 Schubert, M., Lindgreen, S. & Orlando, L. AdapterRemoval v2: rapid adapter trimming, identification, and read merging. *BMC Research Notes* **9**, 88, doi:10.1186/s13104-016-1900-2 (2016).
73 Shen, W., Le, S., Li, Y. & Hu, F. SeqKit: A Cross-Platform and Ultrafast Toolkit for FASTA/Q File Manipulation. *PLoS One* **11**, e0163962, doi:10.1371/journal.pone.0163962 (2016).

74 Schmieder, R. & Edwards, R. Quality control and preprocessing of metagenomic datasets. *Bioinformatics* **27**, 863-864, doi:10.1093/bioinformatics/btr026 (2011).
75 Elven, R., Murray, D., Razzhivin, V. Y. & Yurtsev, B. *Annotated checklist of the Panarctic Flora (PAF)*, <http://panarcticflora.org> (2011).
76 Chamberlain, S. A. & Szocs, E. taxize: taxonomic search and retrieval in R. *F1000Res* **2**, 191, doi:10.12688/f1000research.2-191.v2 (2013).
77 Chen, S., Zhou, Y., Chen, Y. & Gu, J. fastp: an ultra-fast all-in-one FASTQ preprocessor. *Bioinformatics* **34**, i884-i890, doi:10.1093/bioinformatics/bty560 (2018).
78 Liu, S., Gilbert, M. T. P. & Dalén, L. in *Plant and Animal Genome XXVII Conference (January 12-16, 2019).* (PAG).
79 Palkopoulou, E. *et al.* Complete genomes reveal signatures of demographic and genetic declines in the woolly mammoth. *Current Biology* **25**, 1395-1400, doi:10.1016/j.cub.2015.04.007 (2015).
80 Li, H. *et al.* The Sequence Alignment/Map format and SAMtools. *Bioinformatics* **25**, 2078-2079, doi:10.1093/bioinformatics/btp352 (2009).
81 Steinegger, M. & Salzberg, S. L. Terminating contamination: large-scale search identifies more than 2,000,000 contaminated entries in GenBank. *Genome Biology* **21**, 115, doi:10.1186/s13059-020-02023-1 (2020).
82 Epp, L. S. *et al.* New environmental metabarcodes for analysing soil DNA: potential for studying past and present ecosystems. *Molecular Ecology* **21**, 1821-1833, doi:10.1111/j.1365-294X.2012.05537.x (2012).
83 Fahner, N. A., Shokralla, S., Baird, D. J. & Hajibabaei, M. Large-Scale Monitoring of Plants through Environmental DNA Metabarcoding of Soil: Recovery, Resolution, and Annotation of Four DNA Markers. *PLoS One* **11**, e0157505, doi:10.1371/journal.pone.0157505 (2016).
84 Riaz, T. *et al.* ecoPrimers: inference of new DNA barcode markers from whole genome sequence analysis. *Nucleic Acids Research* **39**, e145, doi:10.1093/nar/gkr732 (2011).
85 Kircher, M., Sawyer, S. & Meyer, M. Double indexing overcomes inaccuracies in multiplex sequencing on the Illumina platform. *Nucleic Acids Research* **40**, e3, doi:10.1093/nar/gkr771 (2012).
86 Birks, H. H. The importance of plant macrofossils in the reconstruction of Lateglacial vegetation and climate: examples from Scotland, western Norway, and Minnesota, USA. *Quaternary Sci Rev* **22**, 453-473, doi:10.1016/s0277-3791(02)00248-2 (2003).
87 Birks, H. H. Holocene vegetational history and climatic change in west Spitsbergen - plant macrofossils from Skardtjørna, an Arctic lake. *The Holocene* **1**, 209-218, doi:10.1177/095968369100100303 (2016).
88 Edwards, M. E. The maturing relationship between Quaternary paleoecology and ancient sedimentary DNA. *Quaternary Res* **96**, 39-47, doi:10.1017/qua.2020.52 (2020).
89 Paulson, J. N., Stine, O. C., Bravo, H. C. & Pop, M. Differential abundance analysis for microbial marker-gene surveys. *Nature Methods* **10**, 1200-1202, doi:10.1038/nmeth.2658 (2013).

90 Love, M. I., Huber, W. & Anders, S. Moderated estimation of fold change and dispersion for RNA-seq data with DESeq2. *Genome Biology* **15**, doi:10.1186/s13059-014-0550-8 (2014).
91 Miller, W. *et al.* Sequencing the nuclear genome of the extinct woolly mammoth. *Nature* **456**, 387-390, doi:10.1038/nature07446 (2008).
92 Barnett, R. *et al.* Phylogeography of lions (Panthera leo ssp.) reveals three distinct taxa and a late Pleistocene reduction in genetic diversity. *Molecular Ecology* **18**, 1668-1677, doi:10.1111/j.1365-294X.2009.04134.x (2009).
93 Jonsson, H., Ginolhac, A., Schubert, M., Johnson, P. L. & Orlando, L. mapDamage2.0: fast approximate Bayesian estimates of ancient DNA damage parameters. *Bioinformatics* **29**, 1682-1684, doi:10.1093/bioinformatics/btt193 (2013).
94 Li, H. & Durbin, R. Fast and accurate short read alignment with Burrows-Wheeler transform. *Bioinformatics* **25**, 1754-1760, doi:10.1093/bioinformatics/btp324 (2009).
95 Schubert, M. *et al.* Improving ancient DNA read mapping against modern reference genomes. *Bmc Genomics* **13**, 178, doi:10.1186/1471-2164-13-178 (2012).
96 McColl, H. *et al.* The prehistoric peopling of Southeast Asia. *Science* **361**, 88-92, doi:10.1126/science.aat3628 (2018).
97 Stroeven, A. P. *et al.* Deglaciation of Fennoscandia. *Quaternary Sci Rev* **147**, 91-121, doi:10.1016/j.quascirev.2015.09.016 (2016).
98 Hughes, A. L. C., Gyllencreutz, R., Lohne, Ø. S., Mangerud, J. & Svendsen, J. I. The last Eurasian ice sheets - a chronological database and time-slice reconstruction, DATED-1. *Boreas* **45**, 1-45, doi:10.1111/bor.12142 (2016).
99 Fredin, O. Glacial inception and Quaternary mountain glaciations in Fennoscandia. *Quatern Int* **95-96**, 99-112, doi:10.1016/s1040-6182(02)00031-9 (2002).
100 Taberlet, P. *et al.* Power and limitations of the chloroplast trnL (UAA) intron for plant DNA barcoding. *Nucleic Acids Research* **35**, e14, doi:10.1093/nar/gkl938 (2007).
101 Thomsen, P. F. *et al.* Environmental DNA from Seawater Samples Correlate with Trawl Catches of Subarctic, Deepwater Fishes. *PLoS One* **11**, e0165252, doi:10.1371/journal.pone.0165252 (2016).
102 Boyer, F. *et al.* obitools: a unix-inspired software package for DNA metabarcoding. *Molecular Ecology Resources* **16**, 176-182, doi:10.1111/1755-0998.12428 (2016).
103 Alberdi, A., Aizpurua, O., Gilbert, M. T. P., Bohmann, K. & Mahon, A. Scrutinizing key steps for reliable metabarcoding of environmental samples. *Methods in Ecology and Evolution*, doi:10.1111/2041-210x.12849 (2017).
104 Alsos, I. G. *et al.* Sedimentary ancient DNA from Lake Skartjørna, Svalbard: Assessing the resilience of arctic flora to Holocene climate change. *The Holocene* **26**, 627-642, doi:10.1177/0959683615612563 (2015).
105 Sønstebø, J. H. *et al.* Using next-generation sequencing for molecular reconstruction of past Arctic vegetation and climate. *Molecular Ecology Resources* **10**, 1009-1018, doi:10.1111/j.1755-0998.2010.02855.x (2010).

106 Soininen, E. M. *et al.* Highly overlapping winter diet in two sympatric lemming species revealed by DNA metabarcoding. *PLoS One* **10**, e0115335, doi:10.1371/journal.pone.0115335 (2015).
107 Holden, P. B. *et al.* PALEO-PGEM v1.0: a statistical emulator of Pliocene–Pleistocene climate. *Geoscientific Model Development* **12**, 5137-5155, doi:10.5194/gmd-12-5137-2019 (2019).
108 Holden, P. B. *et al.* PLASIM–GENIE v1.0: a new intermediate complexity AOGCM. *Geoscientific Model Development* **9**, 3347-3361, doi:10.5194/gmd-9-3347-2016 (2016).
109 Luthi, D. *et al.* High-resolution carbon dioxide concentration record 650,000-800,000 years before present. *Nature* **453**, 379-382, doi:10.1038/nature06949 (2008).
110 Berger, A. & Loutre, M. F. Parameters of the Earths orbit for the last 5 Million years in 1 kyr resolution. *PANGAEA* **10**, doi:10.1594/PANGAEA.56040 (1999).
111 Stap, L. B., van de Wal, R. S. W., de Boer, B., Bintanja, R. & Lourens, L. J. The influence of ice sheets on temperature during the past 38 million years inferred from a one-dimensional ice sheet–climate model. *Clim Past* **13**, 1243-1257, doi:10.5194/cp-13-1243-2017 (2017).
112 Karger, D. N. *et al.* Climatologies at high resolution for the earth's land surface areas. *Scientific Data* **4**, 170122, doi:10.1038/sdata.2017.122 (2017).
113 Kageyama, M. *et al.* The PMIP4 Last Glacial Maximum experiments: preliminary results and comparison with the PMIP3 simulations. *Clim Past* **17**, 1065-1089, doi:10.5194/cp-17-1065-2021 (2021).
114 Nilsson-Kerr, K., Anand, P., Holden, P. B., Clemens, S. C. & Leng, M. J. Dipole patterns in tropical precipitation were pervasive across landmasses throughout Marine Isotope Stage 5. *Communications Earth & Environment* **2**, doi:10.1038/s43247-021-00133-7 (2021).
115 Ramirez, J. *BioCalc: Bioclim Variables Calculator. R package version 0.1/r19*, <https://R-Forge.R-project.org/projects/bcvars/> (2009).
116 Martindale, A. *et al.* Canadian Archaeological Radiocarbon Database (CARD 2.1), accessed 6, 2, 2020.
117 Vermeersch, P. M. Radiocarbon Palaeolithic Europe database: A regularly updated dataset of the radiometric data regarding the Palaeolithic of Europe, Siberia included. *Data in Brief* **31**, 105793, doi:10.1016/j.dib.2020.105793 (2020).
118 Bever, M. R. Too Little, Too Late? The Radiocarbon Chronology of Alaska and the Peopling of the New World. *American Antiquity* **71**, 595-620, doi:10.2307/40035881 (2017).
119 Graf, K. E., Ketron, C. V. & Waters, M. R. *Paleoamerican odyssey*. 83-97 (Texas A&M University Press, 2014).
120 Goebel, T. & Potter, B. in *The Oxford Handbook of the Prehistoric Arctic* 223 (Oxford University Press, 2016).
121 Phillips, S. J., Dudík, M. & Schapire, R. E. *Maxent software for modeling species niches and distributions version 3.4.1*, <http://biodiversityinformatics.amnh.org/open_source/maxent/> (2020).
122 Wood, S. *mgcv: Mixed GAM Computation Vehicle with Automatic Smoothness Estimation*, <https://cran.r-project.org/web/packages/mgcv/index.html> (2021).

123 Ripley, B. & Venables, W. *nnet: Feed-Forward Neural Networks and Multinomial Log-Linear Models*, <https://cran.r-project.org/web/packages/nnet/index.html> (2021).
124 Liaw, A. & Wiener, M. *randomForest: Breiman and Cutler's random forests for classification and regression*, <https://CRAN.R-project.org/package=randomForest> (2018).
125 Thuiller, W., Georges, D., Engler, R. & Breiner, F. *biomod2: Ensemble Platform for Species Distribution Modeling*, <https://cran.r-project.org/web/packages/biomod2/index.html> (2020).
126 Lindgren, F., Rue, H. & Lindström, J. An explicit link between Gaussian fields and Gaussian Markov random fields: the stochastic partial differential equation approach. *Journal of the Royal Statistical Society: Series B (Statistical Methodology)* **73**, 423-498, doi:10.1111/j.1467-9868.2011.00777.x (2011).
127 Cameletti, M., Lindgren, F., Simpson, D. & Rue, H. Spatio-temporal modeling of particulate matter concentration through the SPDE approach. *AStA Advances in Statistical Analysis* **97**, 109-131, doi:10.1007/s10182-012-0196-3 (2012).
128 Bakka, H. *et al.* Spatial modeling with R - INLA: A review. *Wiley Interdisciplinary Reviews: Computational Statistics* **10**, e1443 (2018).
129 Fuglstad, G.-A., Simpson, D., Lindgren, F. & Rue, H. Constructing Priors that Penalize the Complexity of Gaussian Random Fields. *Journal of the American Statistical Association* **114**, 445-452, doi:10.1080/01621459.2017.1415907 (2018).
130 Rue, H. *et al.* Bayesian Computing with INLA: A Review. *Annual Review of Statistics and Its Application* **4**, 395-421, doi:10.1146/annurev-statistics-060116-054045 (2017).
131 Lindgren, F. & Rue, H. Bayesian spatial modelling with R-INLA. *J Stat Softw* **63**, 1-25 (2015).
132 Rue, H., Martino, S. & Chopin, N. Approximate Bayesian inference for latent Gaussian models by using integrated nested Laplace approximations. *Journal of the Royal Statistical Society: Series B (Statistical Methodology)* **71**, 319-392, doi:10.1111/j.1467-9868.2008.00700.x (2009).
133 Krainski, E. T. *et al. Advanced spatial modeling with stochastic partial differential equations using R and INLA*. (CRC Press, 2018).
134 Watanabe, S. A widely applicable Bayesian information criterion. *Journal of Machine Learning Research* **14**, 867-897 (2013).
135 Pettit, L. I. The Conditional Predictive Ordinate for the Normal Distribution. *Journal of the Royal Statistical Society: Series B (Methodological)* **52**, 175-184, doi:10.1111/j.2517-6161.1990.tb01780.x (1990).
136 Gómez-Rubio, V. *Bayesian inference with INLA*. (CRC Press, 2020).
137 Martiniano, R., De Sanctis, B., Hallast, P. & Durbin, R. Placing ancient DNA sequences into reference phylogenies. *bioRxiv* (2020).
138 Pecnerova, P. *et al.* Mitogenome evolution in the last surviving woolly mammoth population reveals neutral and functional consequences of small population size. *Evolution Letters* **1**, 292-303, doi:10.1002/evl3.33 (2017).
139 Penny, D. *et al.* Complete Mitochondrial Genome and Phylogeny of Pleistocene MammothMammuthus primigenius. *PLoS Biology* **4**, doi:10.1371/journal.pbio.0040073 (2006).

140 Chang, D. *et al.* The evolutionary and phylogeographic history of woolly mammoths: a comprehensive mitogenomic analysis. *Scientific Reports* **7**, 44585, doi:10.1038/srep44585 (2017).
141 Roca, A. L. *et al.* Elephant natural history: a genomic perspective. *Annual Review of Animal Biosciences* **3**, 139-167, doi:10.1146/annurev-animal-022114-110838 (2015).
142 Lister, A. M. & Sher, A. V. Evolution and dispersal of mammoths across the Northern Hemisphere. *Science* **350**, 805-809, doi:10.1126/science.aac5660 (2015).
143 Palkopoulou, E. *et al.* Holarctic genetic structure and range dynamics in the woolly mammoth. *Proceedings of the Royal Society B: Biological Sciences* **280**, doi:10.1098/rspb.2013.1910 (2013).
144 Gillette, D. D. & Madsen, D. B. The Columbian mammoth, Mammuthus columbi, from the Wasatch Mountains of central Utah. *Journal of Paleontology* **67**, 669-680, doi:10.1017/s0022336000024999 (2016).
145 Debruyne, R. *et al.* Out of America: ancient DNA evidence for a new world origin of late quaternary woolly mammoths. *Current Biology* **18**, 1320-1326, doi:10.1016/j.cub.2008.07.061 (2008).
146 Enk, J. *et al.* Mammuthus Population Dynamics in Late Pleistocene North America: Divergence, Phylogeography, and Introgression. *Frontiers in Ecology and Evolution* **4**, doi:10.3389/fevo.2016.00042 (2016).
147 Krause, J. *et al.* Multiplex amplification of the mammoth mitochondrial genome and the evolution of Elephantidae. *Nature* **439**, 724-727, doi:10.1038/nature04432 (2005).
148 Poinar, H. N. *et al.* Metagenomics to paleogenomics: large-scale sequencing of mammoth DNA. *Science* **311**, 392-394, doi:10.1126/science.1123360 (2006).
149 Kornienko, I. V. *et al.* Complete mitochondrial genome of a woolly mammoth (Mammuthus primigenius) from Maly Lyakhovsky Island (New Siberian Islands, Russia) and its phylogenetic assessment. *Mitochondrial DNA Part B* **3**, 596-598, doi:10.1080/23802359.2018.1473721 (2018).
150 Edgar, R. C. MUSCLE: multiple sequence alignment with high accuracy and high throughput. *Nucleic Acids Research* **32**, 1792-1797, doi:10.1093/nar/gkh340 (2004).
151 Page, A. J. *et al.* SNP-sites: rapid efficient extraction of SNPs from multi-FASTA alignments. *Microbial Genomics* **2**, e000056, doi:10.1099/mgen.0.000056 (2016).
152 Bouckaert, R. *et al.* BEAST 2.5: An advanced software platform for Bayesian evolutionary analysis. *PLoS Computational Biology* **15**, e1006650, doi:10.1371/journal.pcbi.1006650 (2019).
153 Der Sarkissian, C. *et al.* Evolutionary Genomics and Conservation of the Endangered Przewalski's Horse. *Current Biology* **25**, 2577-2583, doi:10.1016/j.cub.2015.08.032 (2015).
154 Gaunitz, C. *et al.* Ancient genomes revisit the ancestry of domestic and Przewalski's horses. *Science* **360**, 111-114, doi:10.1126/science.aao3297 (2018).
155 MacHugh, D. E., Larson, G. & Orlando, L. Taming the Past: Ancient DNA and the Study of Animal Domestication. *Annual Review of Animal Biosciences* **5**, 329-351, doi:10.1146/annurev-animal-022516-022747 (2017).

156 Anthony, D. W. *The horse, the wheel, and language: how Bronze-Age riders from the Eurasian steppes shaped the modern world*. (Princeton University Press, 2010).
157 Achilli, A. *et al.* Mitochondrial genomes from modern horses reveal the major haplogroups that underwent domestication. *Proc Natl Acad Sci U S A* **109**, 2449-2454, doi:10.1073/pnas.1111637109 (2012).
158 Andersson, L. S. *et al.* Mutations in DMRT3 affect locomotion in horses and spinal circuit function in mice. *Nature* **488**, 642-646, doi:10.1038/nature11399 (2012).
159 Der Sarkissian, C. *et al.* Mitochondrial genomes reveal the extinct Hippidion as an outgroup to all living equids. *Biology Letters* **11**, doi:10.1098/rsbl.2014.1058 (2015).
160 Druzhkova, A. S. *et al.* Complete mitochondrial genome of an extinct Equus (Sussemionus) ovodovi specimen from Denisova cave (Altai, Russia). *Mitochondrial DNA Part B* **2**, 79-81, doi:10.1080/23802359.2017.1285209 (2017).
161 Fages, A. *et al.* Tracking Five Millennia of Horse Management with Extensive Ancient Genome Time Series. *Cell* **177**, 1419-1435 e1431, doi:10.1016/j.cell.2019.03.049 (2019).
162 Finno, C. J. *et al.* SERPINB11 frameshift variant associated with novel hoof specific phenotype in Connemara ponies. *PLoS Genetics* **11**, e1005122, doi:10.1371/journal.pgen.1005122 (2015).
163 Goto, H. *et al.* A massively parallel sequencing approach uncovers ancient origins and high genetic variability of endangered Przewalski's horses. *Genome Biology and Evolution* **3**, 1096-1106, doi:10.1093/gbe/evr067 (2011).
164 Heintzman, P. D. *et al.* A new genus of horse from Pleistocene North America. *Elife* **6**, e29944 (2017).
165 Jun, J. *et al.* Whole genome sequence and analysis of the Marwari horse breed and its genetic origin. *Bmc Genomics* **15 Suppl 9**, S4, doi:10.1186/1471-2164-15-S9-S4 (2014).
166 Jagannathan, V. *et al.* Comprehensive characterization of horse genome variation by whole-genome sequencing of 88 horses. *Animal Genetics* **50**, 74-77, doi:10.1111/age.12753 (2019).
167 Jiang, Q. *et al.* The complete mitochondrial genome and phylogenetic analysis of the Debao pony (Equus caballus). *Molecular Biology Reports* **38**, 593-599, doi:10.1007/s11033-010-0145-8 (2011).
168 Kim, H. *et al.* Peeling back the evolutionary layers of molecular mechanisms responsive to exercise-stress in the skeletal muscle of the racing horse. *DNA Research* **20**, 287-298, doi:10.1093/dnares/dst010 (2013).
169 Do, K.-T. *et al.* Genomic characterization of the Przewalski׳s horse inhabiting Mongolian steppe by whole genome re-sequencing. *Livestock Science* **167**, 86-91, doi:10.1016/j.livsci.2014.06.020 (2014).
170 Leegwater, P. A. *et al.* Dwarfism with joint laxity in Friesian horses is associated with a splice site mutation in B4GALT7. *Bmc Genomics* **17**, 839, doi:10.1186/s12864-016-3186-0 (2016).
171 Librado, P. *et al.* Tracking the origins of Yakutian horses and the genetic basis for their fast adaptation to subarctic environments. *Proc Natl Acad Sci U S A* **112**, E6889-6897, doi:10.1073/pnas.1513696112 (2015).

172 Librado, P. *et al.* Ancient genomic changes associated with domestication of the horse. *Science* **356**, 442-445, doi:10.1126/science.aam5298 (2017).
173 Lippold, S., Matzke, N. J., Reissmann, M. & Hofreiter, M. Whole mitochondrial genome sequencing of domestic horses reveals incorporation of extensive wild horse diversity during domestication. *BMC Evolutionary Biology* **11**, 328, doi:10.1186/1471-2148-11-328 (2011).
174 Metzger, J. *et al.* Runs of homozygosity reveal signatures of positive selection for reproduction traits in breed and non-breed horses. *Bmc Genomics* **16**, 764, doi:10.1186/s12864-015-1977-3 (2015).
175 Sawyer, S., Krause, J., Guschanski, K., Savolainen, V. & Paabo, S. Temporal patterns of nucleotide misincorporations and DNA fragmentation in ancient DNA. *PLoS One* **7**, e34131, doi:10.1371/journal.pone.0034131 (2012).
176 Schrimpf, R. *et al.* Screening of whole genome sequences identified high-impact variants for stallion fertility. *Bmc Genomics* **17**, 288, doi:10.1186/s12864-016-2608-3 (2016).
177 Vilstrup, J. T. *et al.* Mitochondrial phylogenomics of modern and ancient equids. *PLoS One* **8**, e55950, doi:10.1371/journal.pone.0055950 (2013).
178 Xu, S. *et al.* High Altitude Adaptation and Phylogenetic Analysis of Tibetan Horse Based on the Mitochondrial Genome. *Journal of Genetics and Genomics* **34**, 720-729, doi:10.1016/s1673-8527(07)60081-2 (2007).
179 Yuan, J. X. *et al.* Molecular identification of late and terminal Pleistocene Equus ovodovi from northeastern China. *PLoS One* **14**, e0216883, doi:10.1371/journal.pone.0216883 (2019).
