# Supplementary material for: Late Quaternary dynamics of Arctic biota from ancient environmental genomics
Source: Nature. 2021 Oct 20;600(7887):86–92. doi: 10.1038/s41586-021-04016-x (PMC8636272; doi:10.1038/s41586-021-04016-x)
Supplement: Supplementary file 2 — Reporting Summary [file 41586_2021_4016_MOESM2_ESM.pdf]

nature research

Corresponding author(s): Eske Willerslev

Last updated by author(s): Aug 9, 2021

Reporting Summary

Nature Research wishes to improve the reproducibility of the work that we publish. This form provides structure for consistency and transparency in reporting. For further information on Nature Research policies, see our Editorial Policies and the Editorial Policy Checklist.

Statistics

For all statistical analyses, confirm that the following items are present in the figure legend, table legend, main text, or Methods section.

n/a	Confirmed	
☐	☒	The exact sample size (*n*) for each experimental group/condition, given as a discrete number and unit of measurement
☐	☒	A statement on whether measurements were taken from distinct samples or whether the same sample was measured repeatedly
☐	☒	The statistical test(s) used AND whether they are one- or two-sided<br>*Only common tests should be described solely by name; describe more complex techniques in the Methods section.*
☐	☒	A description of all covariates tested
☐	☒	A description of any assumptions or corrections, such as tests of normality and adjustment for multiple comparisons
☐	☒	A full description of the statistical parameters including central tendency (e.g. means) or other basic estimates (e.g. regression coefficient) AND variation (e.g. standard deviation) or associated estimates of uncertainty (e.g. confidence intervals)
☐	☒	For null hypothesis testing, the test statistic (e.g. *F*, *t*, *r*) with confidence intervals, effect sizes, degrees of freedom and *P* value noted<br>*Give P values as exact values whenever suitable.*
☐	☒	For Bayesian analysis, information on the choice of priors and Markov chain Monte Carlo settings
☒	☐	For hierarchical and complex designs, identification of the appropriate level for tests and full reporting of outcomes
☐	☒	Estimates of effect sizes (e.g. Cohen's *d*, Pearson's *r*), indicating how they were calculated

Our web collection on statistics for biologists contains articles on many of the points above.

Software and code

Policy information about availability of computer code

Data collection	No software used for data collection.
Data analysis	Software: FastX (0.0.13), ORG.Annot (1.0.0), Maxent (3.4.1), BEAST (2.5), obitools (1.2.11), BWA (0.7.15), bowtie (1.2.3), samtools (1.10);<br>R packages: vegan (2.5.7), scales (1.1.1), ggplot2 (3.3.3), ComplexHeatmap (2.4.3), Bchron (4.7.4), rbacon (2.5.5), taxize (0.9.99), IntClust (0.1.0), metagenomeSeq (1.30.0), DESeq2 (1.28.1), BioCalc (0.1), mgcv (1.8.36), biomod2 (1.2.5), R-INLA (20.07.09), nnet (7.3.16), randomForest (4.6.14), biomod2 (3.5.1);<br>The custom scripts and code are archived at GitHub repository https://github.com/wyc661217/Arctic_eDNA_2021.

For manuscripts utilizing custom algorithms or software that are central to the research but not yet described in published literature, software must be made available to editors and reviewers. We strongly encourage code deposition in a community repository (e.g. GitHub). See the Nature Research guidelines for submitting code & software for further information.

Data

Policy information about availability of data

All manuscripts must include a data availability statement. This statement should provide the following information, where applicable:

- Accession codes, unique identifiers, or web links for publicly available datasets
- A list of figures that have associated raw data
- A description of any restrictions on data availability

Adapter-removed plant or animal eDNA data is deposited at EMBL-ENA with project accession PRJEB43822. The raw data of PhyloNorway plant genome database is available at EMBL-ENA with project accession PRJEB43865 Assembled plant genome contigs of the PhyloNorway database are available at https://doi.org/10.18710/3CVQAG. NCBI databases are available at the NCBI ftp server https://ftp.ncbi.nlm.nih.gov. Canadian Archaeological Radiocarbon Database

(CARD2.0) is available at https://www.canadianarchaeology.ca. Radiocarbon Palaeolithic Europe Database is available at https://ees.kuleuven.be/geography/projects/14c-palaeolithic. All other data is supplied in Supplementary Information and Supplementary Data tables.

Field-specific reporting

Please select the one below that is the best fit for your research. If you are not sure, read the appropriate sections before making your selection.

☐ Life sciences ☐ Behavioural & social sciences ☒ Ecological, evolutionary & environmental sciences

For a reference copy of the document with all sections, see nature.com/documents/nr-reporting-summary-flat.pdf

Ecological, evolutionary & environmental sciences study design

All studies must disclose on these points even when the disclosure is negative.

Study description

We shotgun sequenced the ancient environmental DNA from sediment across the Arctic, and reconstructed the Arctic biota dynamics in the last 50,000 years using the generated sequencing data.

Research sample

A total of 535 permafrost and lake sediment samples and 1,541 arctic and boreal herbaria specimens were used for data generation.

Sediment samples were selected from the circumpolar sediment sample repository at Centre for GeoGenetics, which contains more than 5,000 samples, following 5 5 criteria. (i) The site (or section) exhibits no sign of reworking or leaching, preferable to be lacustrine or permafrost sedimentary profile (see Section 5 and the corresponding references). (ii) The sampling method should comply with ancient DNA standards (see Section 1.2). (iii) Samples should come from well-characterized sedimentary profiles with explicit documentation on the stratigraphy. (iv) The age of the samples can be precisely determined (see Section 3). (v) The age frame of the sedimentary section is largely younger than 50 kilo annum Before Present (ka BP).

Leaf materials were sampled for the PhyloNorway database from herbarium specimens at Tromsø Museum (herbarium TROM). Specimens were selected by 4 criteria: (i) The species is native in arctic and/or boreal regions; (ii) The specimen appeared healthy, without any visible signs of fungal infection; (iii) Collection date for the specimen is as early in the growing season as possible; (iv) The sampling had proper documentation and reliable taxonomic identification. Common invasive plant species in the Arctic and sub-Arctic were also included.

Sampling strategy

Owing to site-specific sedimentological differences and different sampling strategies from various research groups involved, samples were collected using different methods, but all with deliberate precautions to avoid DNA-sensitive contamination. In general, sampling was either performed by directly withdrawing samples from the profile in situ, or by taking out larger bulk samples that later were subsampled under clean-controlled conditions in the dedicated laboratory at the Centre for GeoGenetics, University of Copenhagen. More detailed description of the two methods can be found in SI section 1.2.

No sample size calculation was performed. This is the first large-scale eDNA metagenomics study and we meant to collect samples evenly covering the Arctic in last 50 kyr at a best possibility (SI section 2).

Data collection

Sediment DNA extraction was performed at Centre for GeoGenetics by Yucheng Wang, and sequenced at the Danish National Sequencing Centre on Illumina platforms (HiSeq 2500, HiSeq 4000, HiSeq X Ten). Herbaria DNA extraction and sequencing were performed by the PhyloNorway team at The Arctic University Museum and Genoscope on Illumina HiSeq 2000. Raw data for both will be published together with the manuscript.

Timing and spatial scale

DNA Data were collected from sediment samples from across the Arctic and spanning the last 50,000 years. The PhyloNorway plant genome reference database was sampled form herbarium specimens from Norway and polar regions deposited at Tromsø University Museum.

Sequencing was performed from 2016-09 to 2018-10. The frequency of sequencing does not affect the data and therefore the results.

Data exclusions

In total, 7 sediment and 12 herbaria samples were excluded, due to possible disturbance of the stratum or contaminations. Details are suppled in Supplementary Information Section 5 and 7.4.

Reproducibility

This is a large scale (both spatial and temporal) study aiming at finding the general patterns of Arctic past biota dynamics. Therefore this is no repeat attempted, but the observed dynamics confirmed by both samples from adjacent sites and from site in different regions. Sediment samples are archived at Centre for GenoGenetics whereas herbarium specimens used for PhyloNorway database are deposited at the herbarium at Tromsø Museum (herbarium code TROM). Both sample types are available on request for anyone who would like to reproduce the study.

Randomization

Randomization is not relevant. Sampling sites were chosen based on a series thresholds, and to offer the best resolution in time and space.

Blinding

Blinding is not relevant, as there is no presupposed hypothesis.

Did the study involve field work? ☒ Yes ☐ No

Field work, collection and transport

Field conditions	Field works were performed by different groups under different conditions. Details are supplied in SI section 1 and the corresponding references. Since all samples were either collected under a clean-controlled condition, or later subsampled in the dedicated clean laboratory at the Centre for GeoGenetics, the filed work conditions do not affect the data and study question.
Location	A total of 74 localities across the Arctic, including: 1, 06D1 (62.383239,9.674164); 2, ANL (69.2544,16.06); 3, VA (70.3167,30.0167); 4, CAS (68.147817,39.758698); 5, GAS (59.066667,56.116667); 6, ZAS (58.15,56.9333); 7, YUB (60.6009,71.9263); 8, MarR1 (68.6557,71.9225); 9, MarR2 (68.656471,71.966054); 10, MK2 (69.7397,84.8181); 11, PO1 (66.8719,86.6269); 12, PO2 (66.7267,86.6391); 13, IH4 (66.758,86.6804); 14, LUR10 (73.1565,93.4072); 15, LoR3 (73.3504,96.9746); 16, UTRD4 (74.2664,99.8264); 17, OVR (74.1464,100.1264); 18, BBR1 (72.5397,100.4312); 19, CS1 (74.5477,100.5358); 20, TLH1 (74.64083333,100.7311111); 21, FI (74.6225,100.828); 22, BBR6 (73.5261667,101.0085); 23, BBR7 (73.5168,101.0089); 24, BAP (74.4936,101.2761); 25, LT (79.2453,101.8153); 26, BBR9 (73.6481,102.0177); 27, BBR10 (73.6481,102.1078); 28, BBS5 (73.65285,102.1207); 29, BBS6 (73.6989,102.1969); 30, KS1 (72.0967,102.3281); 31, KS2 (72.0886,102.2872); 32, DO (71.8667,127.066667); 33, CAB (71.6667,129.5); 34, BK1 (71.9062,132.7864); 35, BK2 (72.0028,132.8336); 36, BK3 (71.9056,132.7853); 37, CHR (69.4833,156.983333); 38, KK (69.3833,158.4667); 39, DY (68.6667,159.08333); 40, PJ (68.6667,160.8333); 41, PP (68.4992,162.4068); 42, MR1 (64.2833,171.25); 43, MR2 (64.2833,171.25); 44, MR3 (64.2833,171.25); 45, MR4 (64.2833,171.25); 46, MR5 (64.2833,171.25); 47, MR6 (64.2833,171.25); 48, AC (64.7352,177.30732); 49, PS (66.2333,-148.2667); 50, SV1 (65.9833,-148.95); 51, SV2 (65.9833,-148.95); 52, AMR (67.7438,-156.1921); 53, RBS (68.3535,-158.8874); 54, ZL (63.471,-162.0532); 55, TH (68.1934,-162.5804); 56, QC (60.985317,-130.501282); 57, RS (63.69,-138.58); 58, CM (63.67,-138.64245); 59, GR (63.683333,-138.6); 60, TC (63.097244,-139.538727); 61, GS (63.9333,-138.9667); 62, NP (60.578887,-139.005478); 63, BS (67.609221,-76.245117); 64, LI (64.398201,-50.201302); 65, K608 (64.60217,-50.5013); 66, LC (61.1399,-45.5347); 67, LS (65.6833333,-37.9166667); 68, DA (79.7216,10.9471); 69, CL10 (78.0925,14.9787); 70, 09C1 (78.0486,15.0909); 71, 09C2 (78.0476,15.0924); 72, ES (78.0329,15.1134); 73, RS1 (78.470996,16.2153); 74, RS2 (78.5584,16.4348).
Access & import/export	Sediment samples were collected and exported by different research groups from different countries, in agreement with the rules of the specific countries. All sediment samples were imported to Denmark as geological sediment samples for research, for which there is no specific permit required by the authorities.
Disturbance	The fieldwork and sampling was carried out exerting minimal disturbance to the areas and geological deposits.

Reporting for specific materials, systems and methods

We require information from authors about some types of materials, experimental systems and methods used in many studies. Here, indicate whether each material, system or method listed is relevant to your study. If you are not sure if a list item applies to your research, read the appropriate section before selecting a response.

Materials & experimental systems

n/a	Involved in the study	
☒	☐	Antibodies
☒	☐	Eukaryotic cell lines
☒	☐	Palaeontology and archaeology
☒	☐	Animals and other organisms
☒	☐	Human research participants
☒	☐	Clinical data
☒	☐	Dual use research of concern

Methods

n/a	Involved in the study	
☒	☐	ChIP-seq
☒	☐	Flow cytometry
☒	☐	MRI-based neuroimaging
