# Supplementary material for: Late Quaternary dynamics of Arctic biota from ancient environmental genomics
Source: Nature. 2021 Oct 20;600(7887):86–92. doi: 10.1038/s41586-021-04016-x (PMC8636272; doi:10.1038/s41586-021-04016-x)

Human Presence/Absence, 05 ka BP

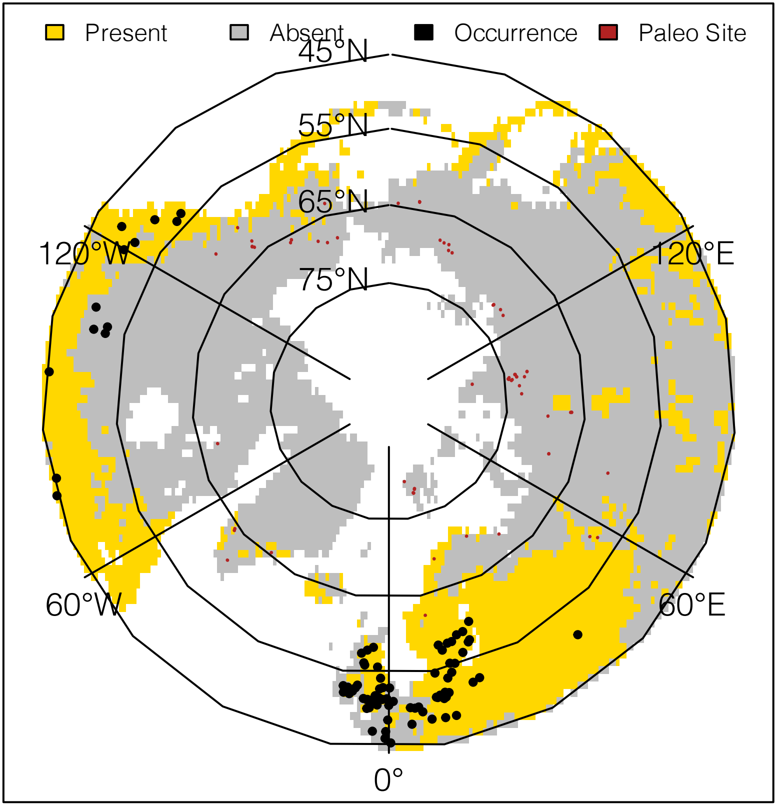

Human Presence/Absence, 06 ka BP

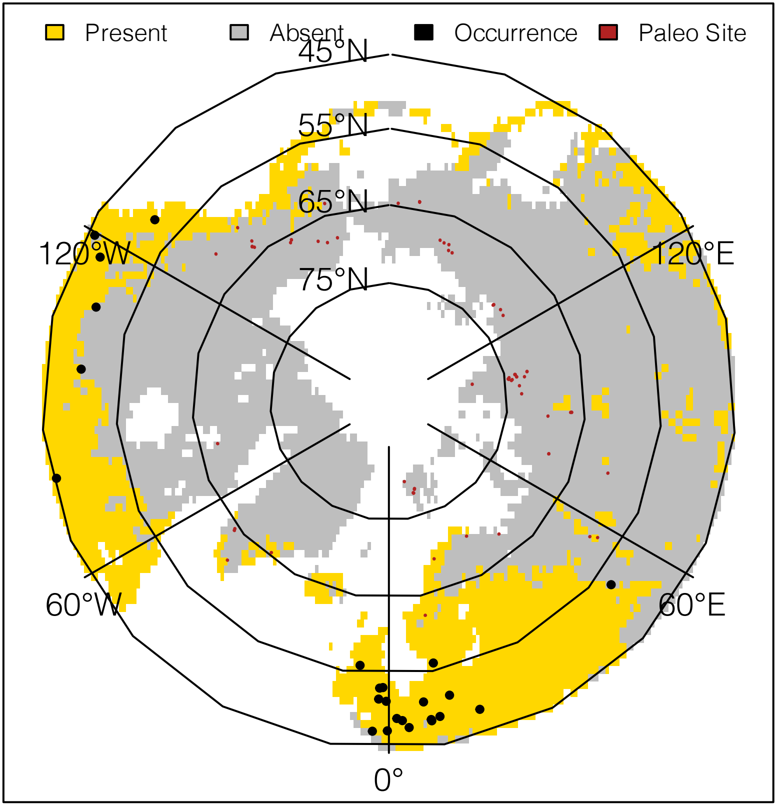

Human Presence/Absence, 07 ka BP

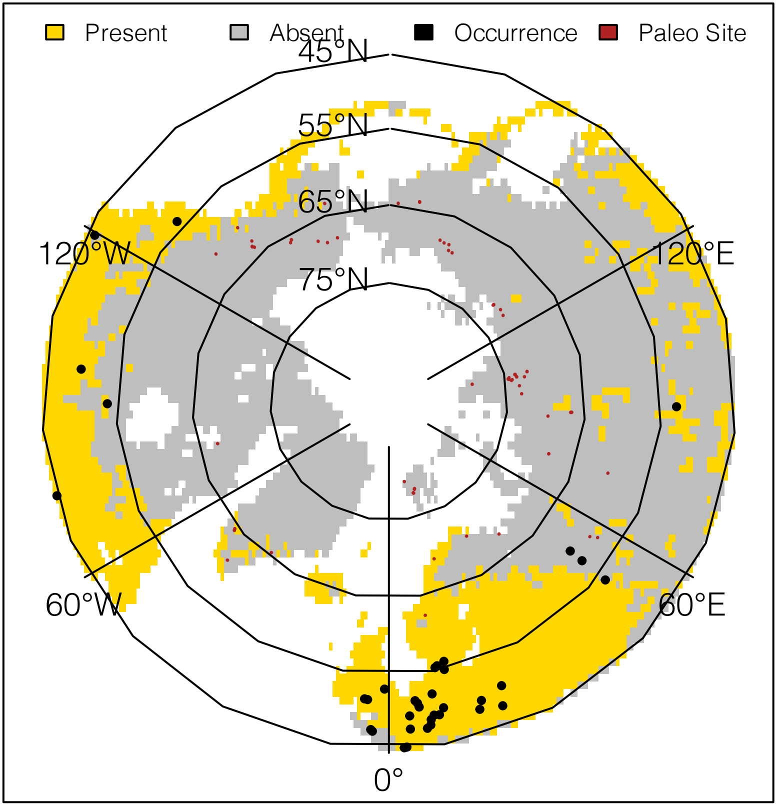

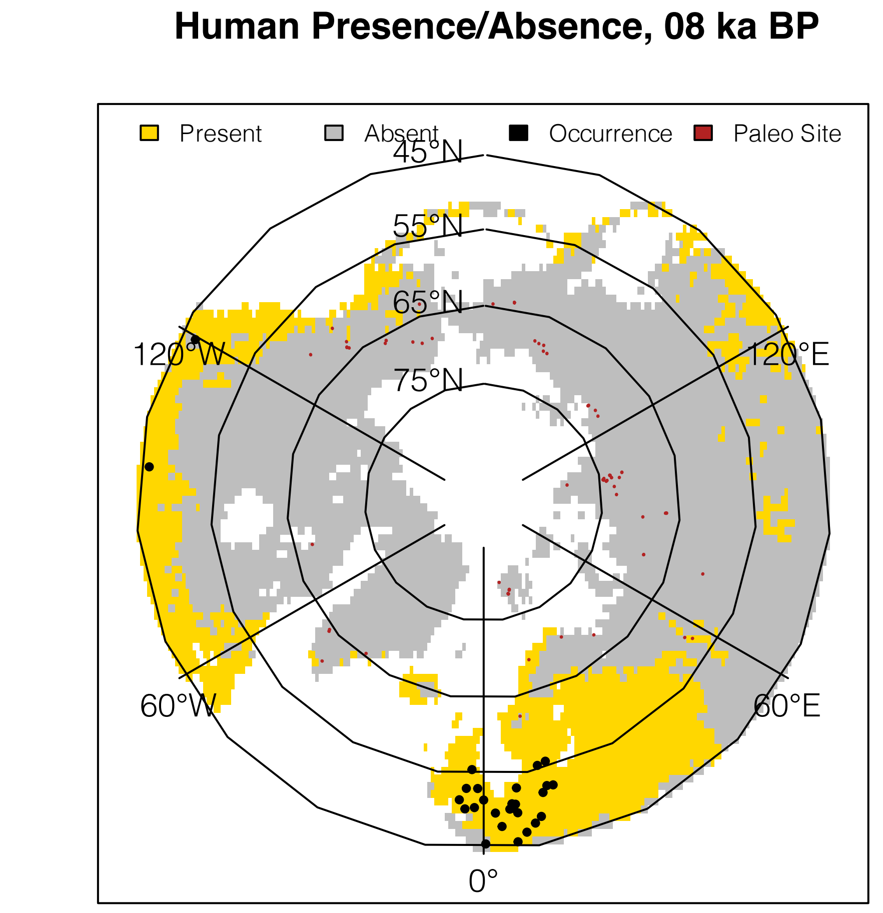
Human Presence/Absence, 08 ka BP
Present
Absent
Occurrence
Paleo Site
45°N
55°N
65°N
75°N
120°W
120°E
60°W
60°E
0°

Human Presence/Absence, 09 ka BP

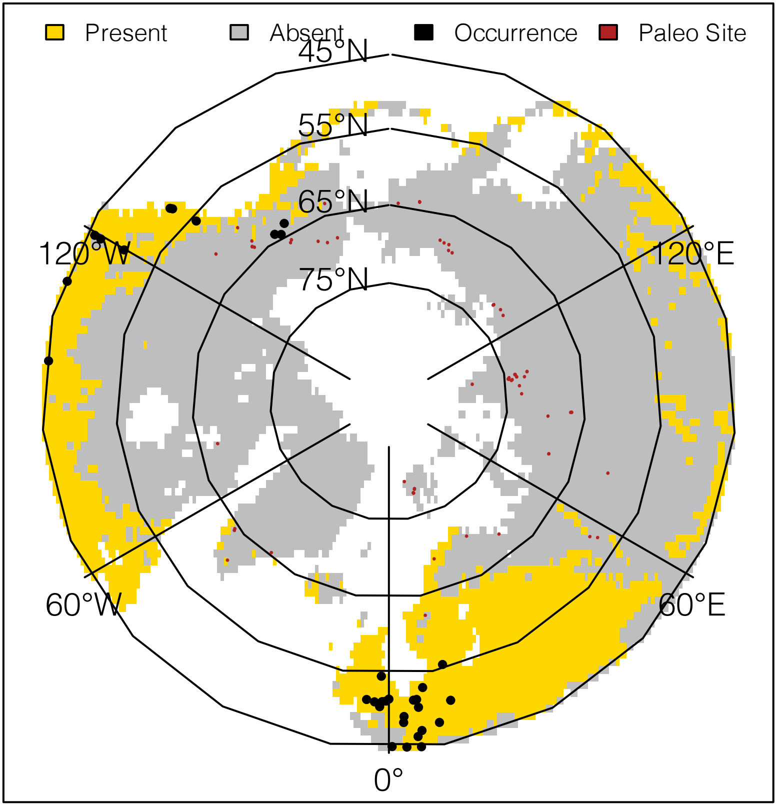

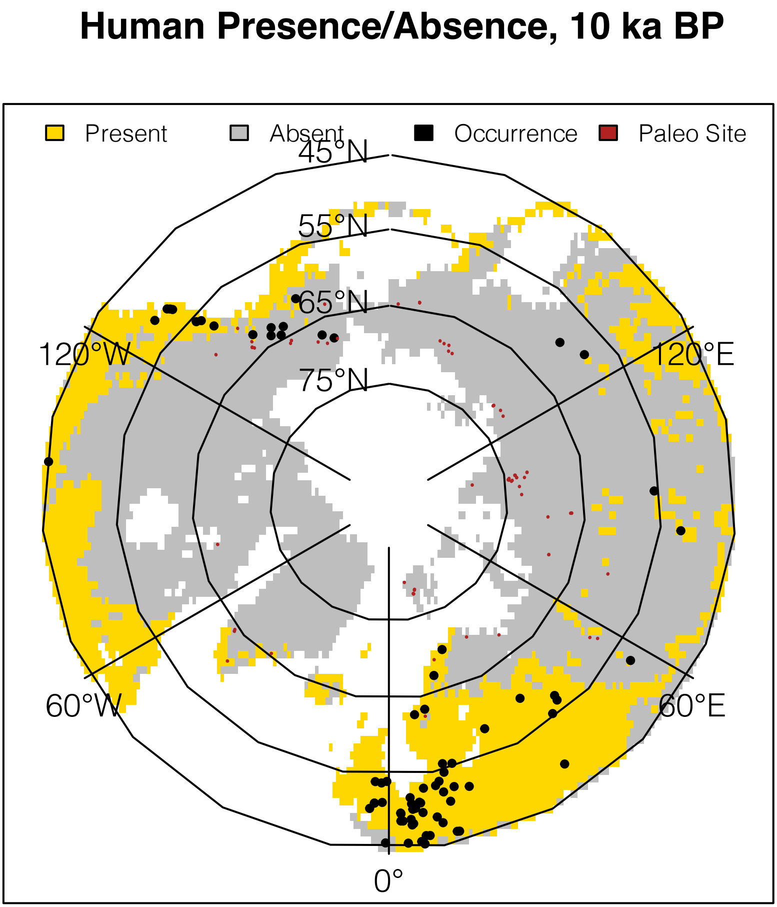

Human Presence/Absence, 10 ka BP
Present
Absent
Occurrence
Paleo Site
45°N
55°N
65°N
75°N
120°W
120°E
60°W
60°E
0°

Human Presence/Absence, 11 ka BP

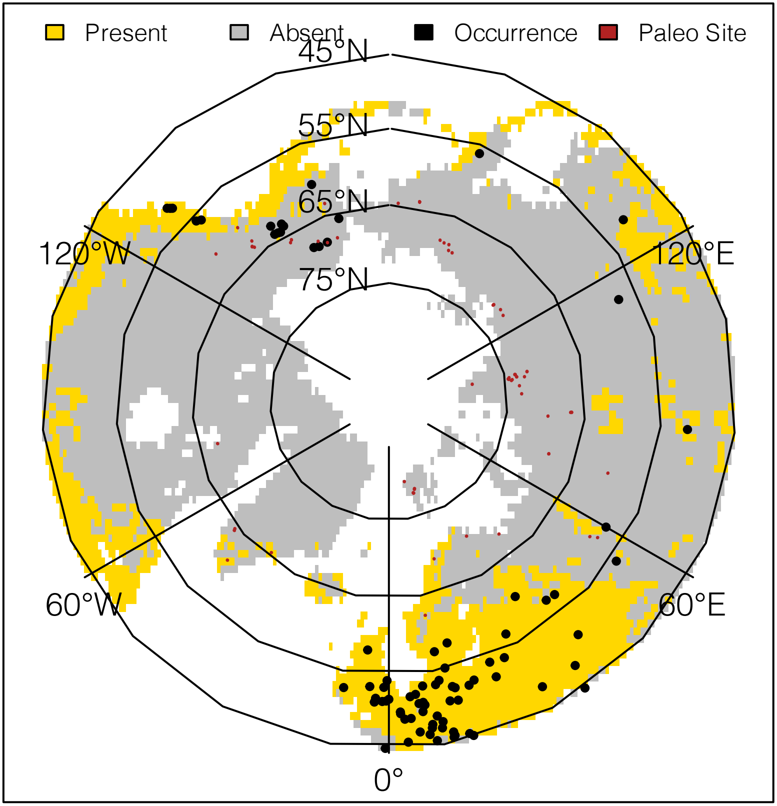

Human Presence/Absence, 12 ka BP

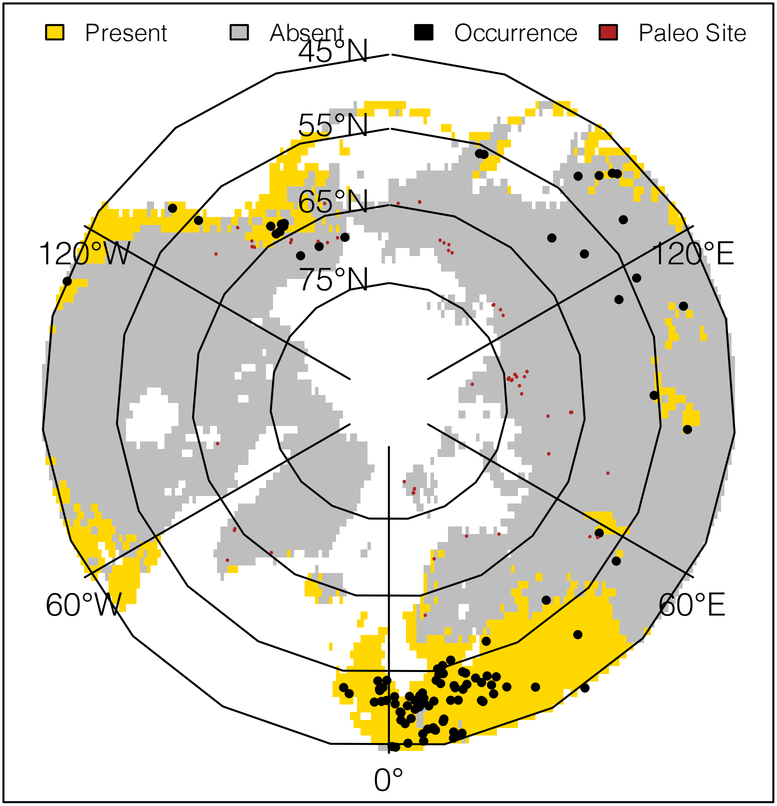

Human Presence/Absence, 13 ka BP

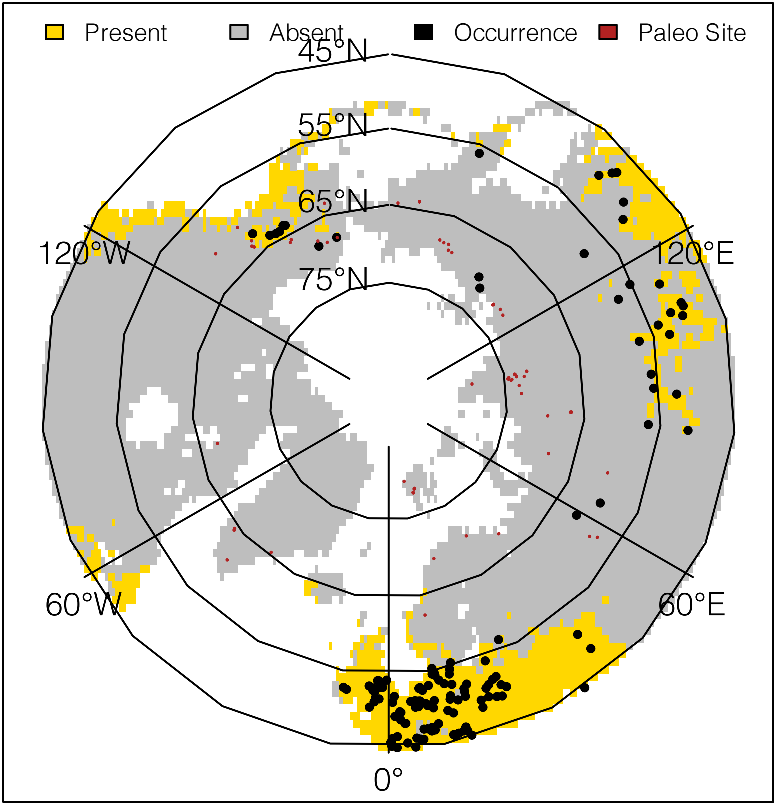

Human Presence/Absence, 14 ka BP

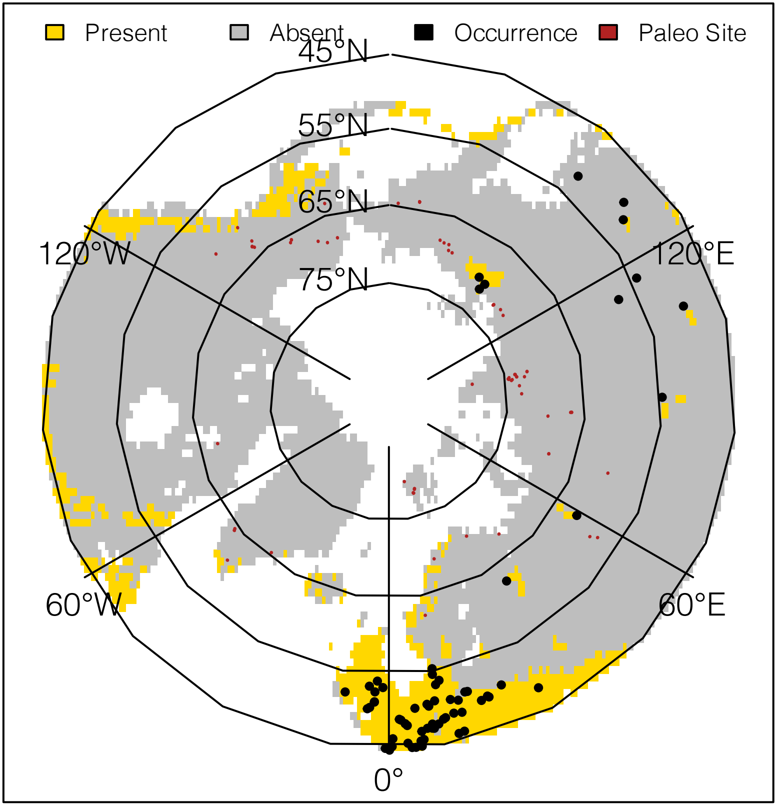

Human Presence/Absence, 15 ka BP

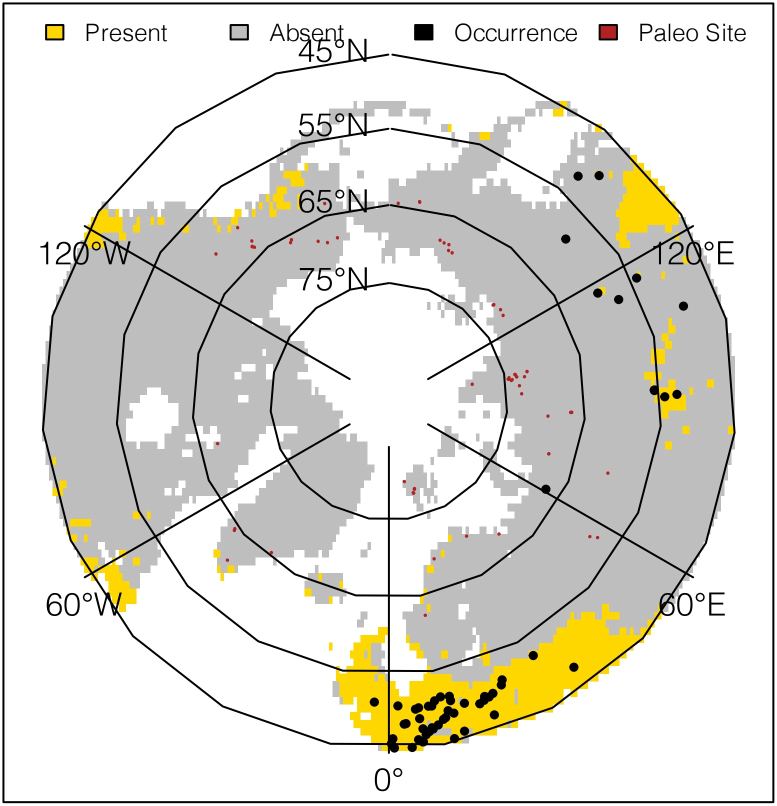

Human Presence/Absence, 16 ka BP

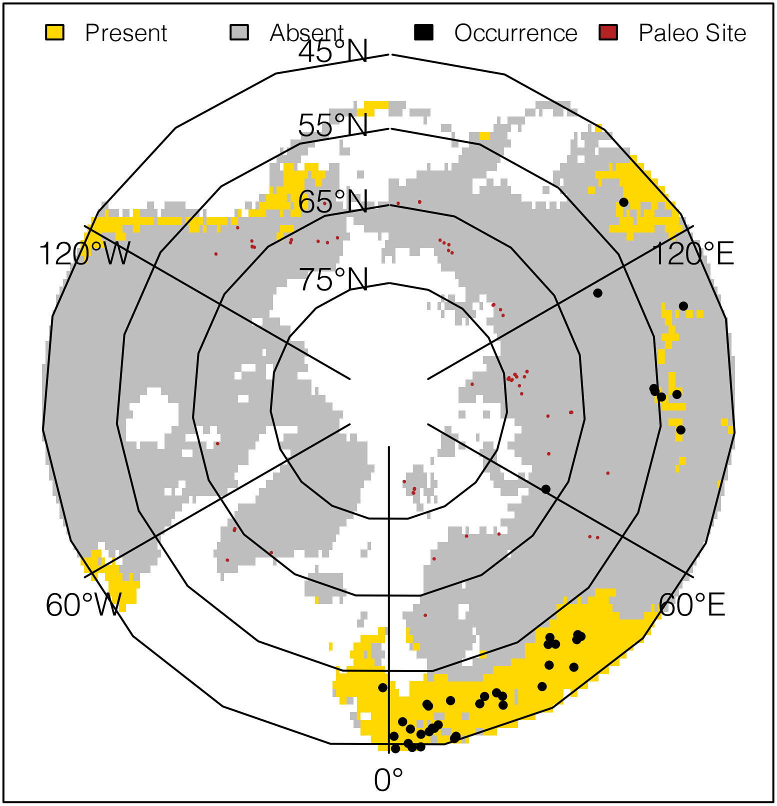

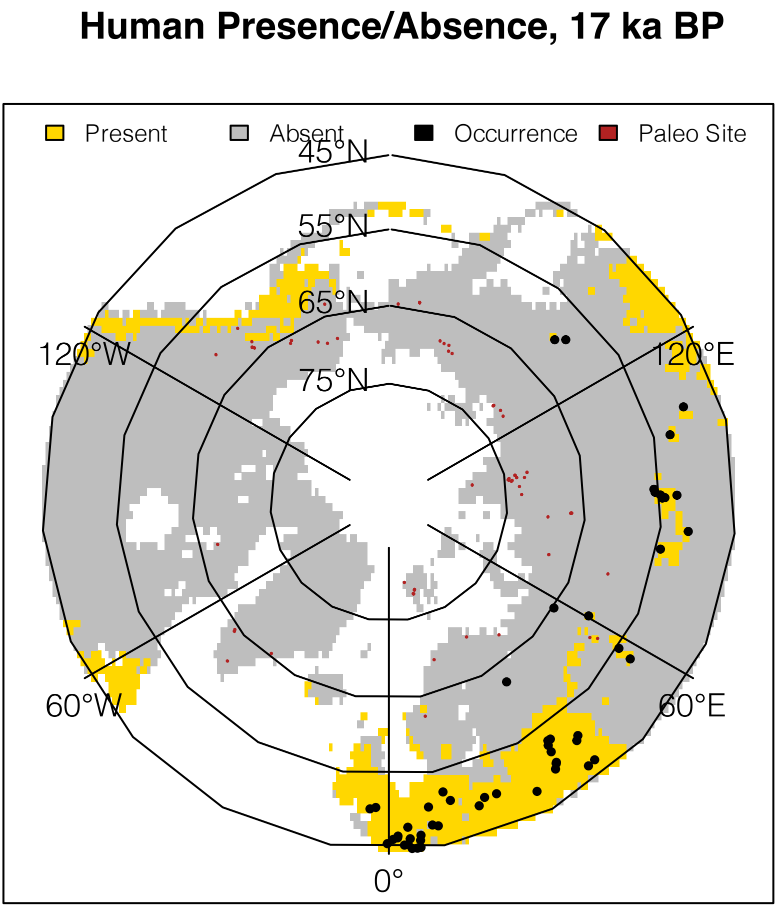

Human Presence/Absence, 17 ka BP
Present
Absent
Occurrence
Paleo Site
45°N
55°N
65°N
75°N
120°W
120°E
60°W
60°E
0°

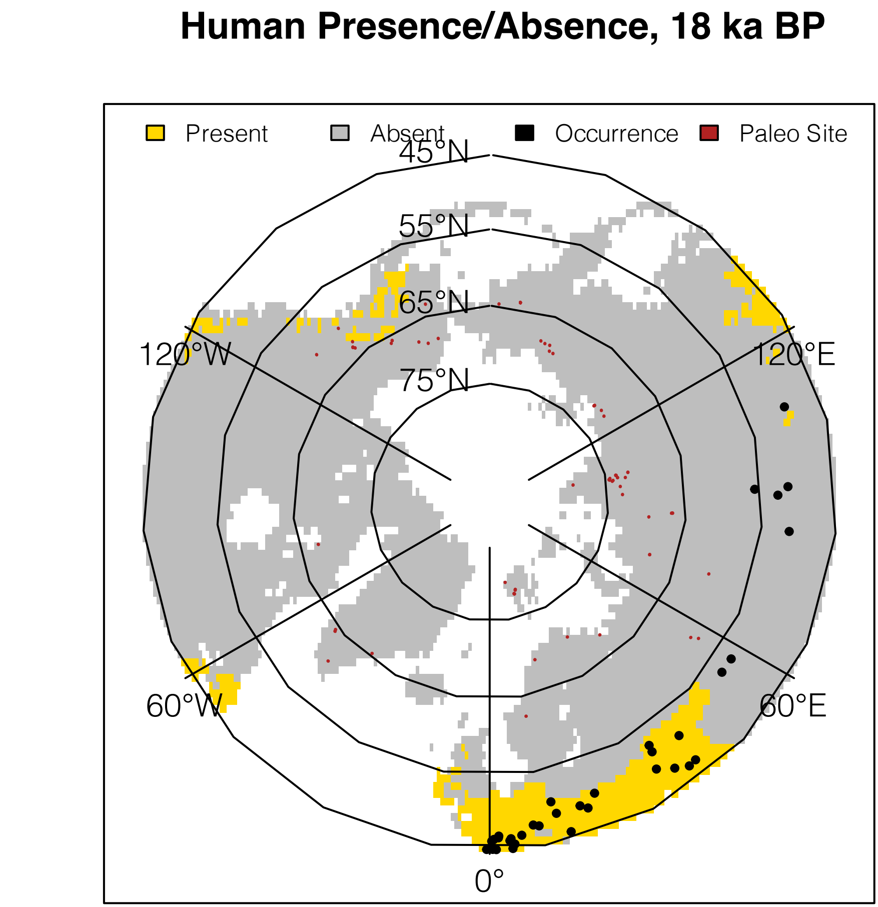
Human Presence/Absence, 18 ka BP
Present
Absent
Occurrence
Paleo Site
45°N
55°N
65°N
75°N
120°W
120°E
60°W
60°E
0°

Human Presence/Absence, 19 ka BP

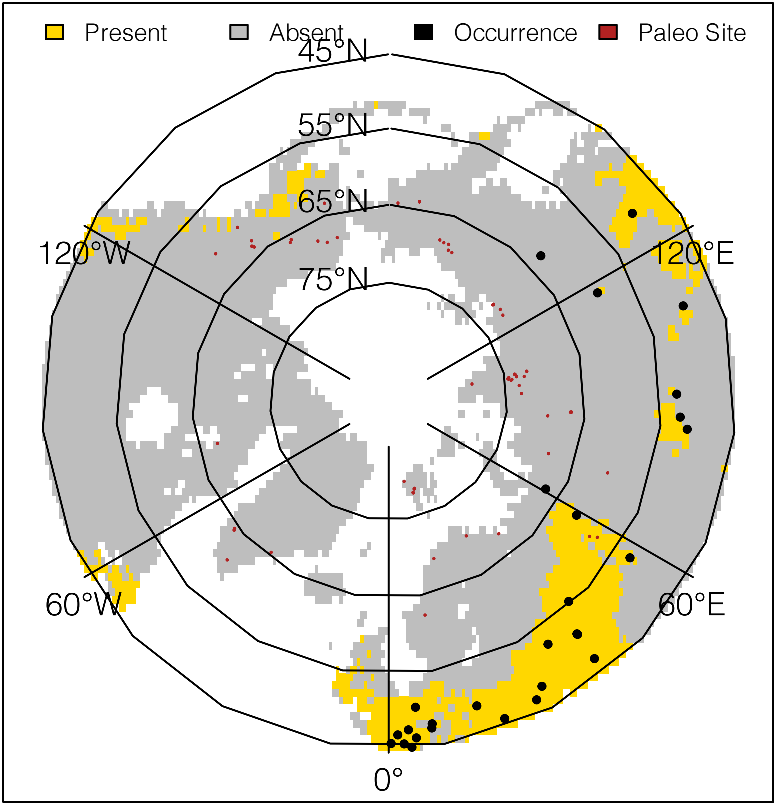

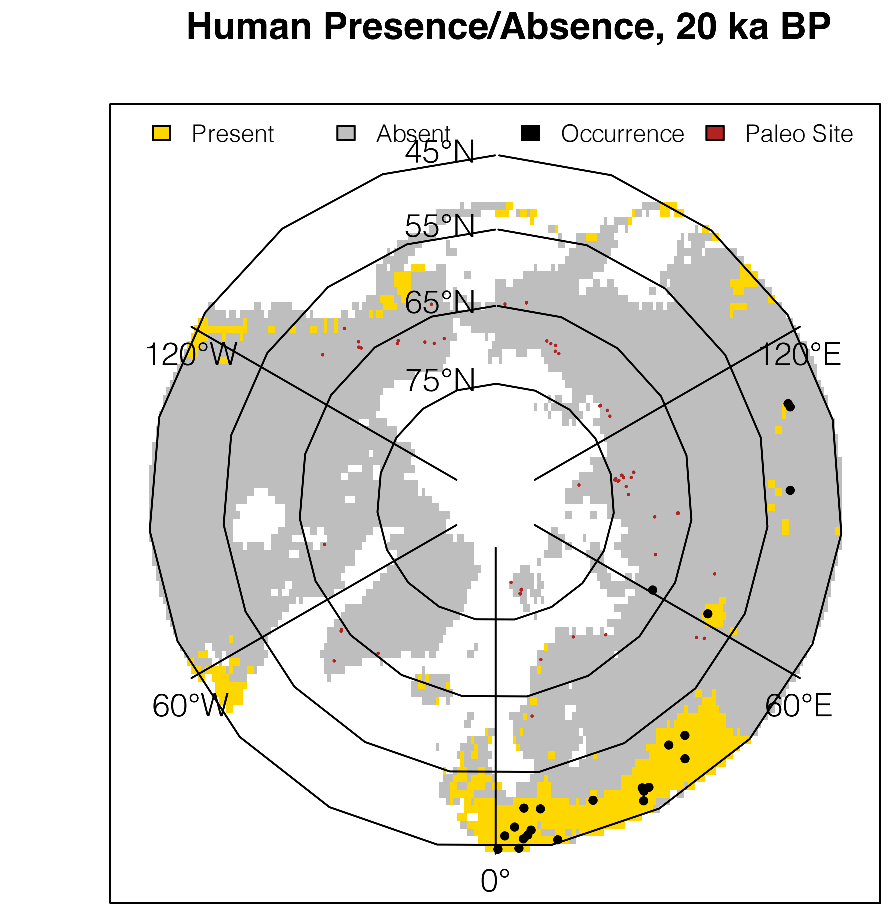
Human Presence/Absence, 20 ka BP
Present
Absent
Occurrence
Paleo Site
45°N
55°N
65°N
75°N
120°W
120°E
60°W
60°E
0°

Human Presence/Absence, 21 ka BP

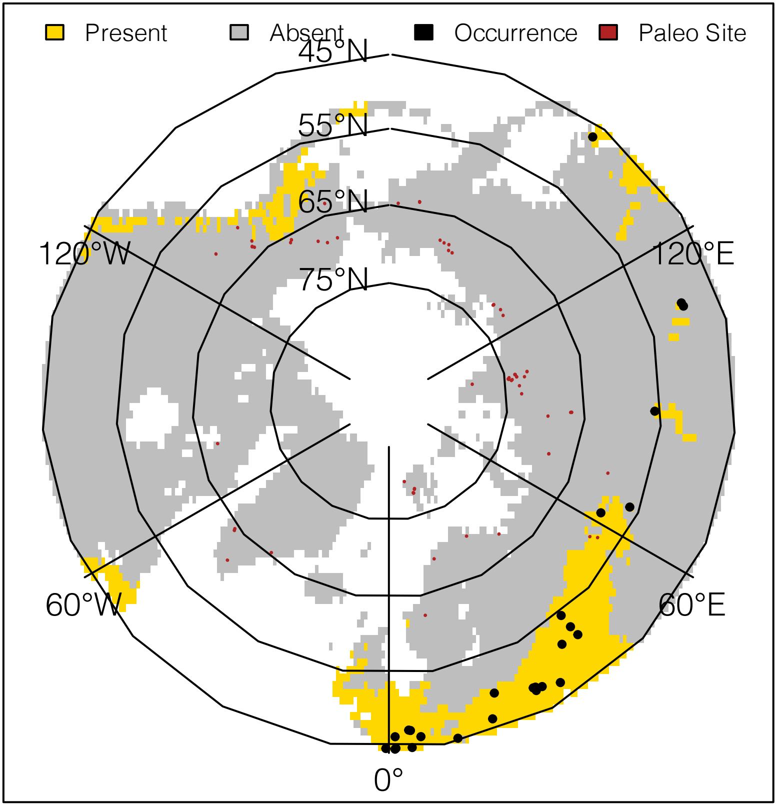

Human Presence/Absence, 22 ka BP

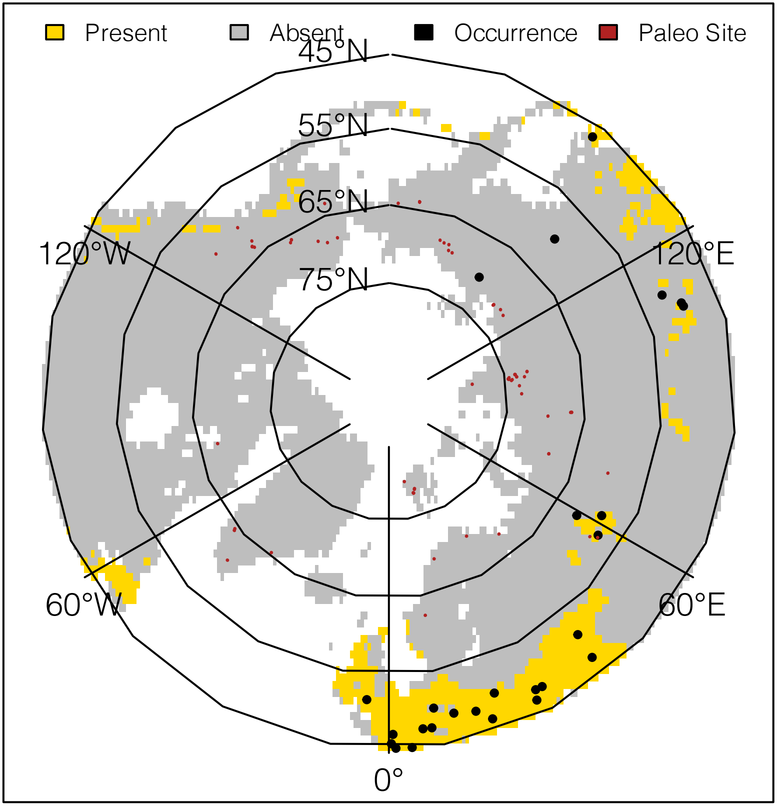

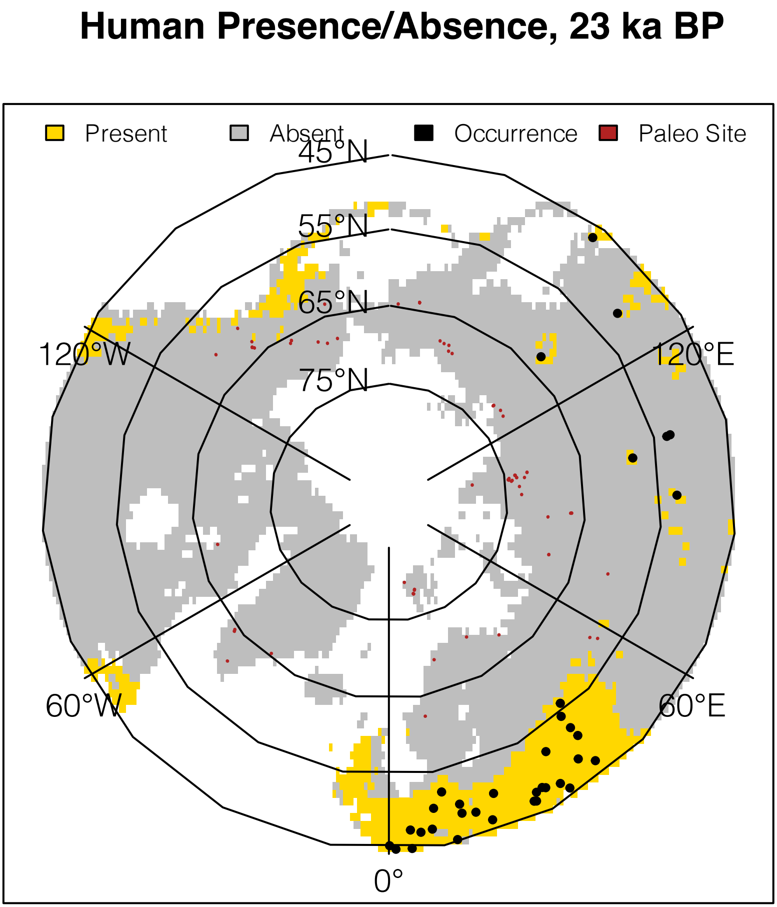
Human Presence/Absence, 23 ka BP
Present
Absent
Occurrence
Paleo Site
45°N
55°N
65°N
75°N
120°W
120°E
60°W
60°E
0°

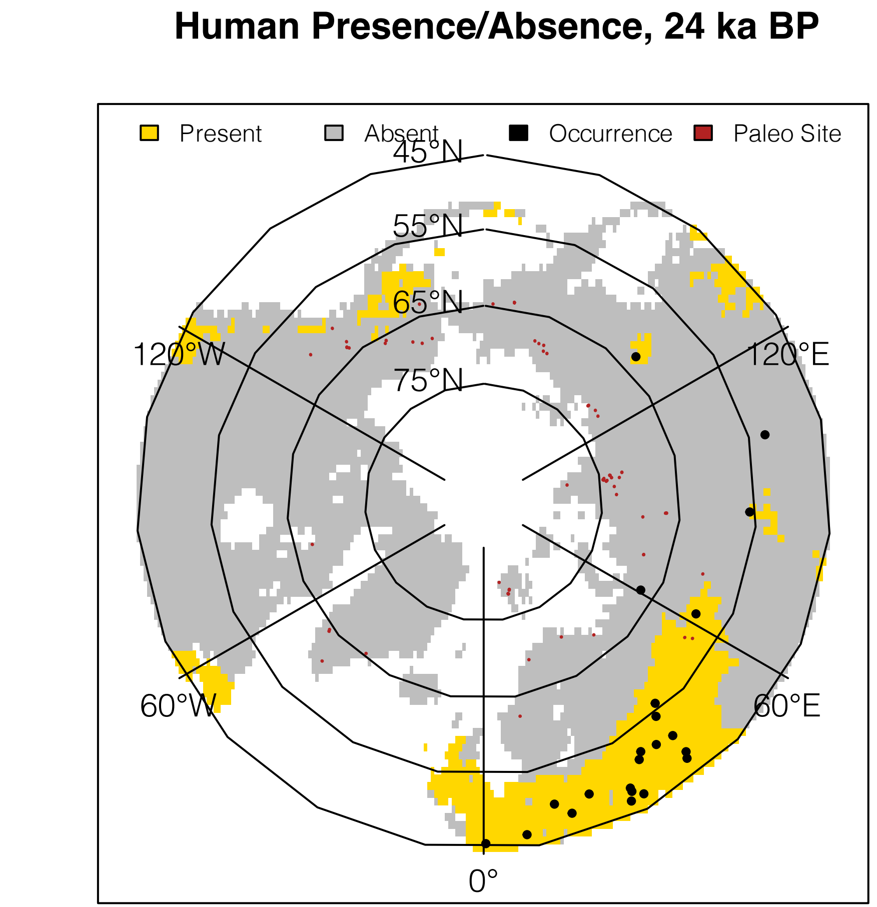

Human Presence/Absence, 24 ka BP
Present
Absent
Occurrence
Paleo Site
45°N
55°N
65°N
75°N
120°W
120°E
60°W
60°E
0°

Human Presence/Absence, 25 ka BP

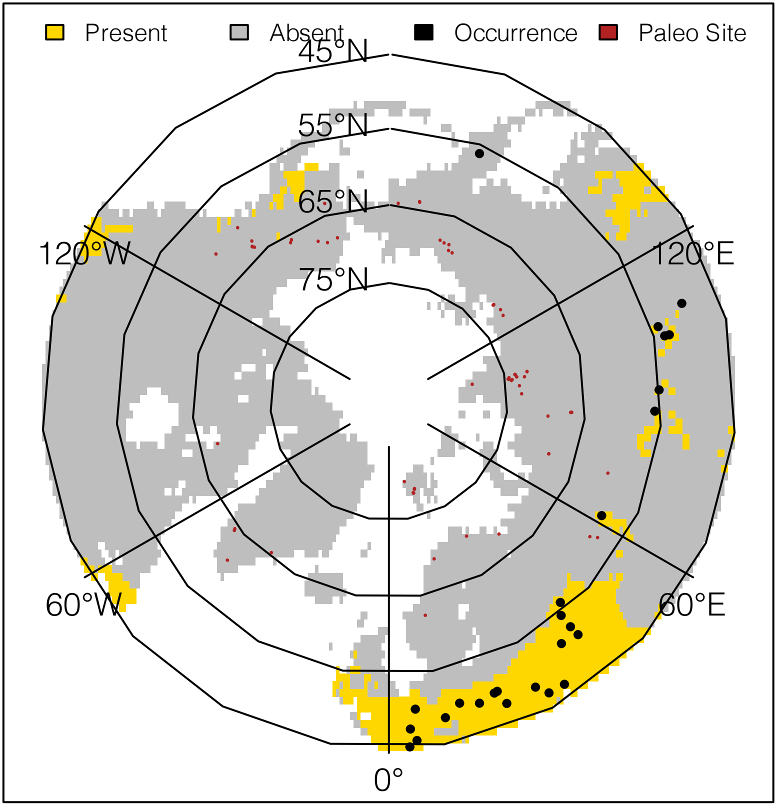

Human Presence/Absence, 26 ka BP

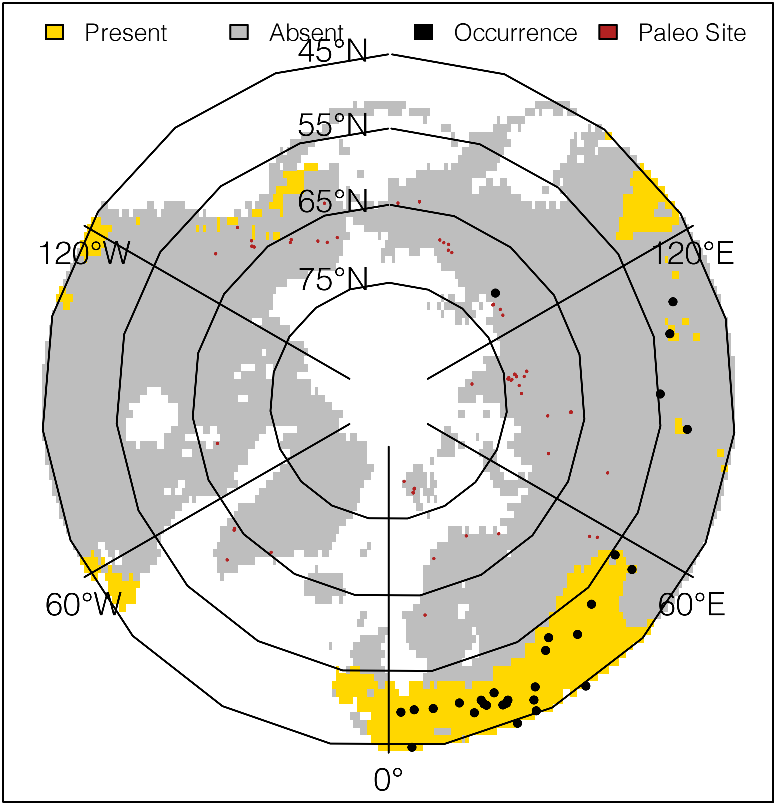

Human Presence/Absence, 27 ka BP

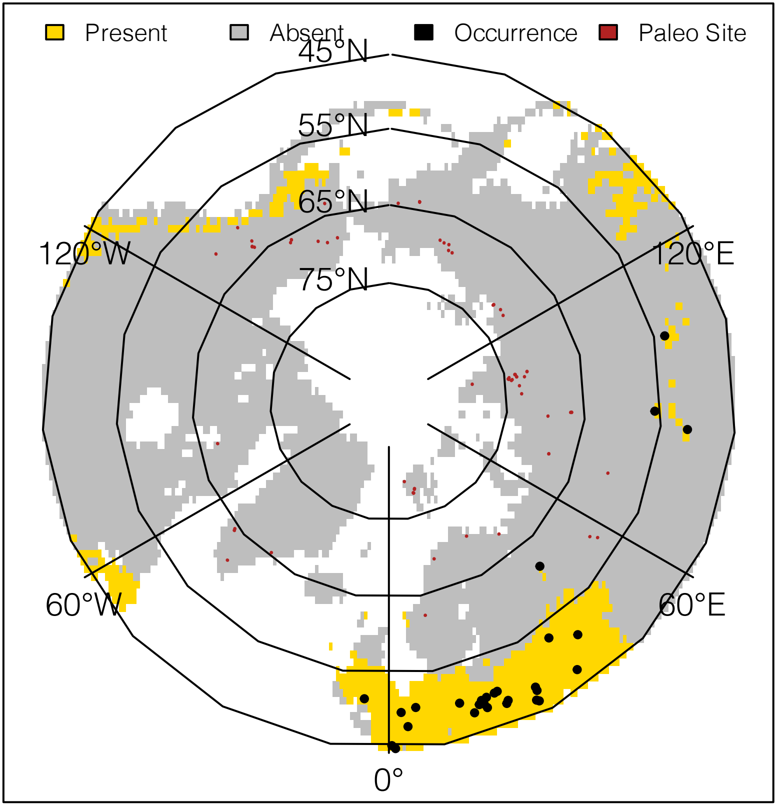

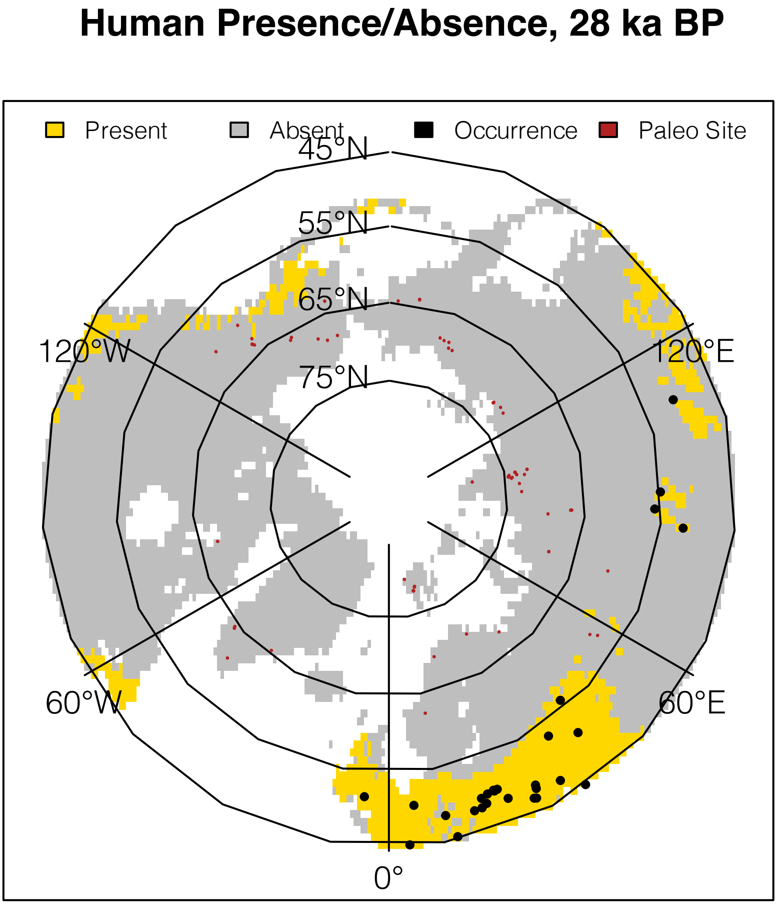
Human Presence/Absence, 28 ka BP
Present
Absent
Occurrence
Paleo Site
45°N
55°N
65°N
75°N
120°W
120°E
60°W
60°E
0°

Human Presence/Absence, 29 ka BP

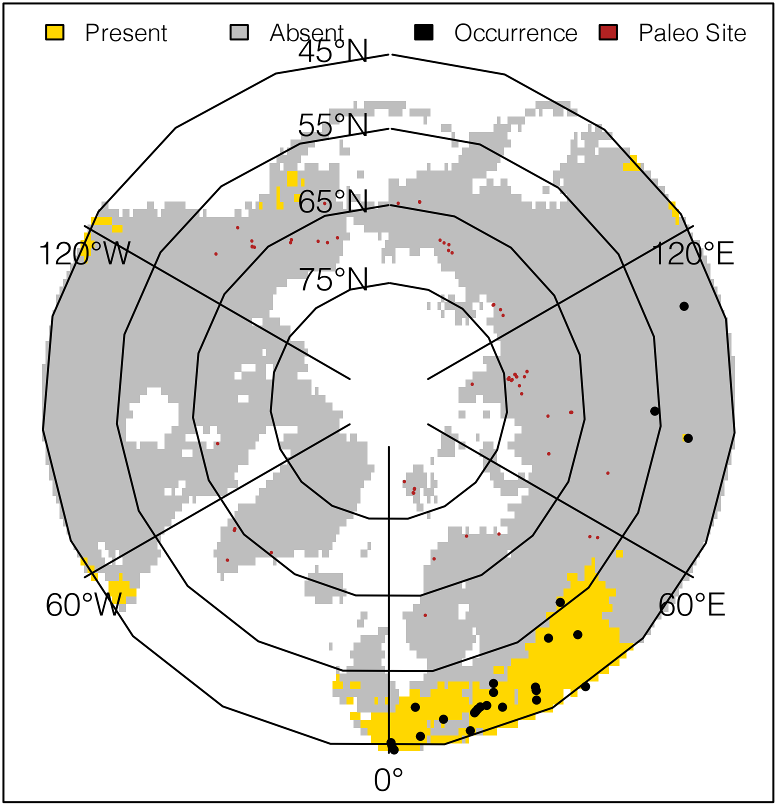

Human Presence/Absence, 30 ka BP

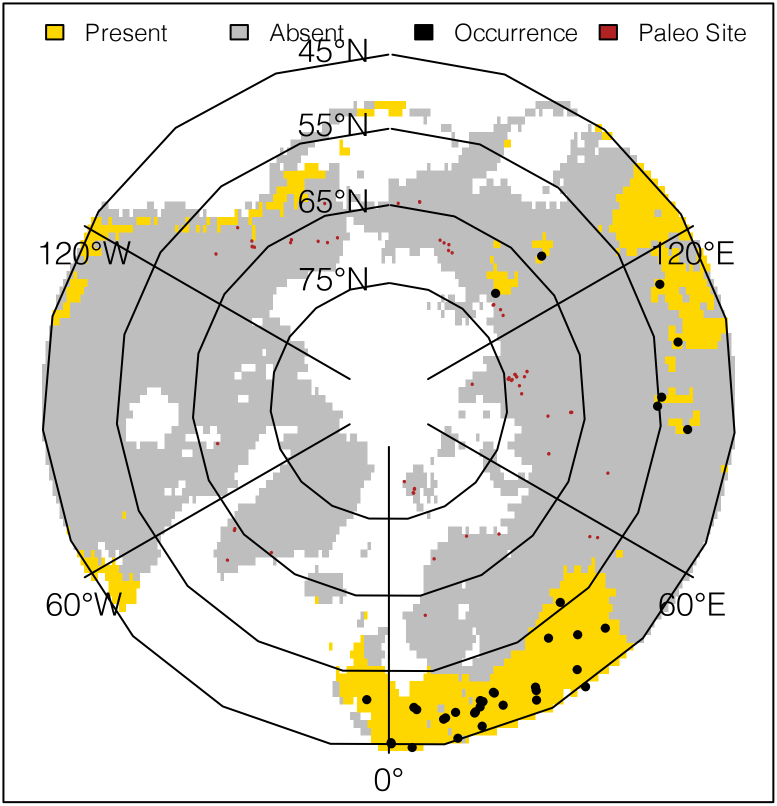

Human Presence/Absence, 31 ka BP

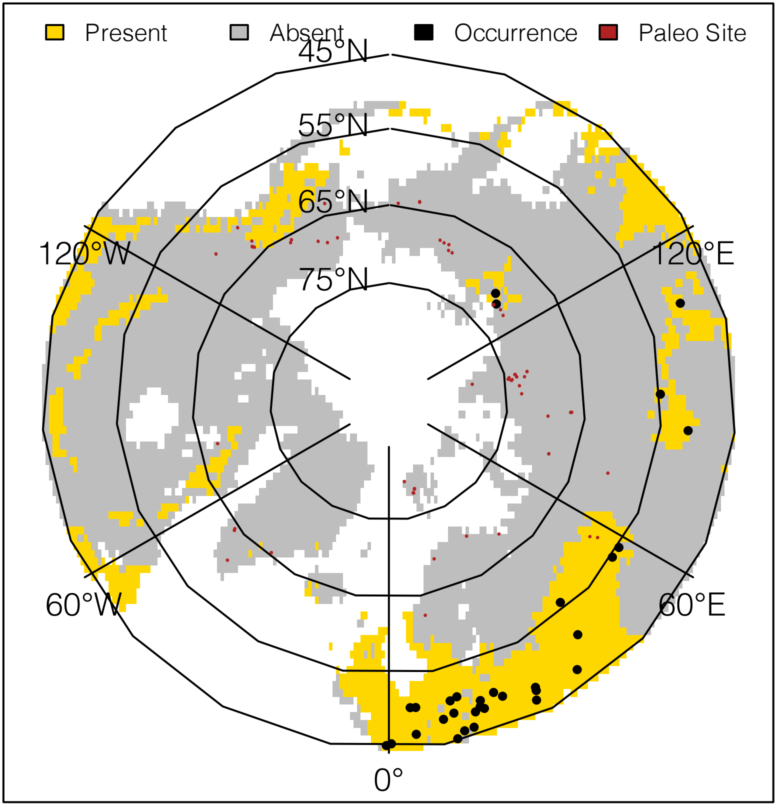

Human Presence/Absence, 32 ka BP

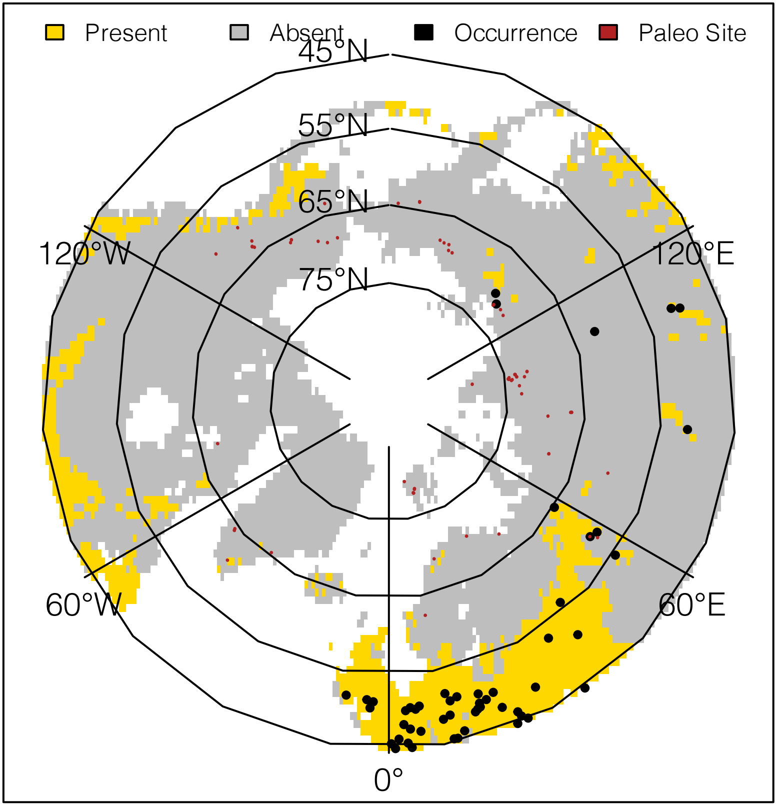

Human Presence/Absence, 34 ka BP

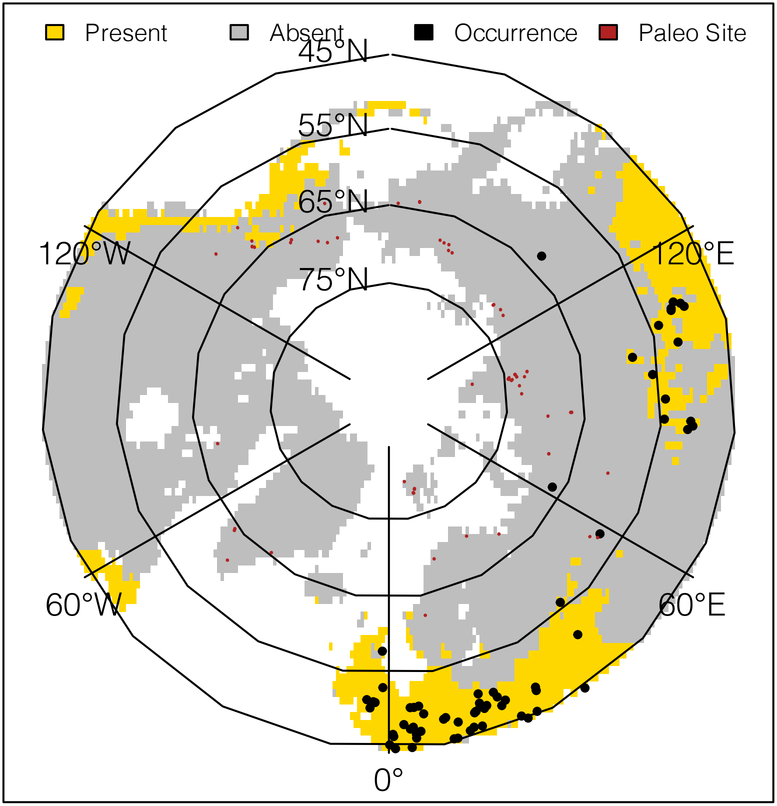

Human Presence/Absence, 36 ka BP

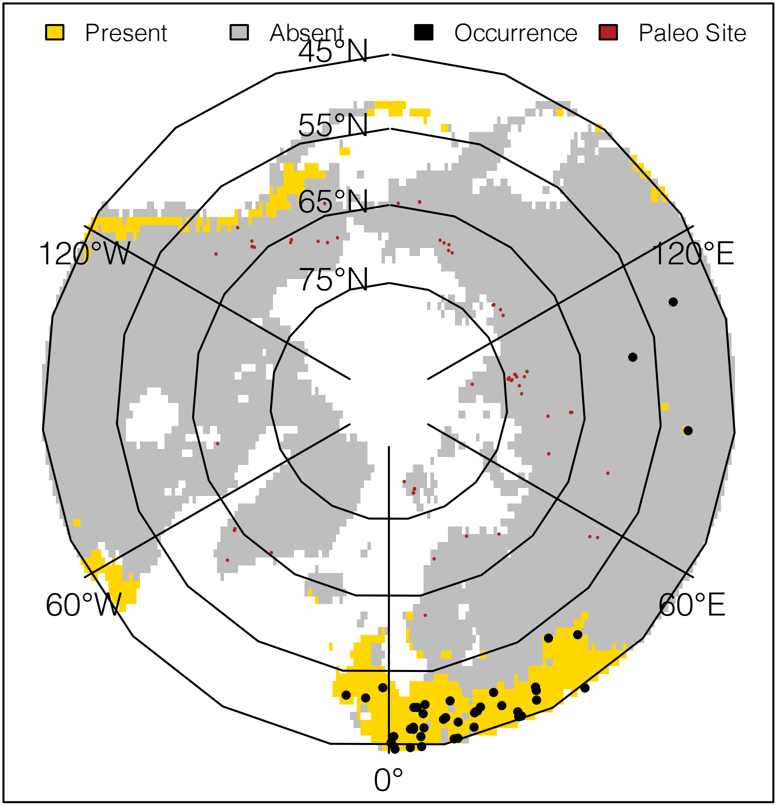

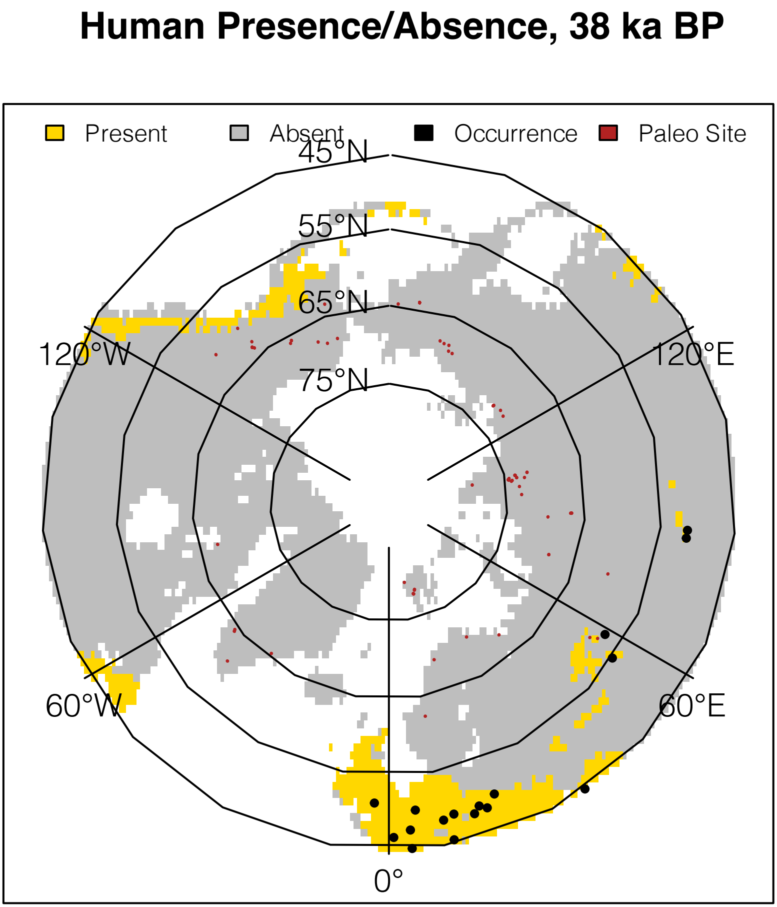

Human Presence/Absence, 38 ka BP
Present
Absent
Occurrence
Paleo Site
45°N
55°N
65°N
75°N
120°W
120°E
60°W
60°E
0°

Human Presence/Absence, 40 ka BP

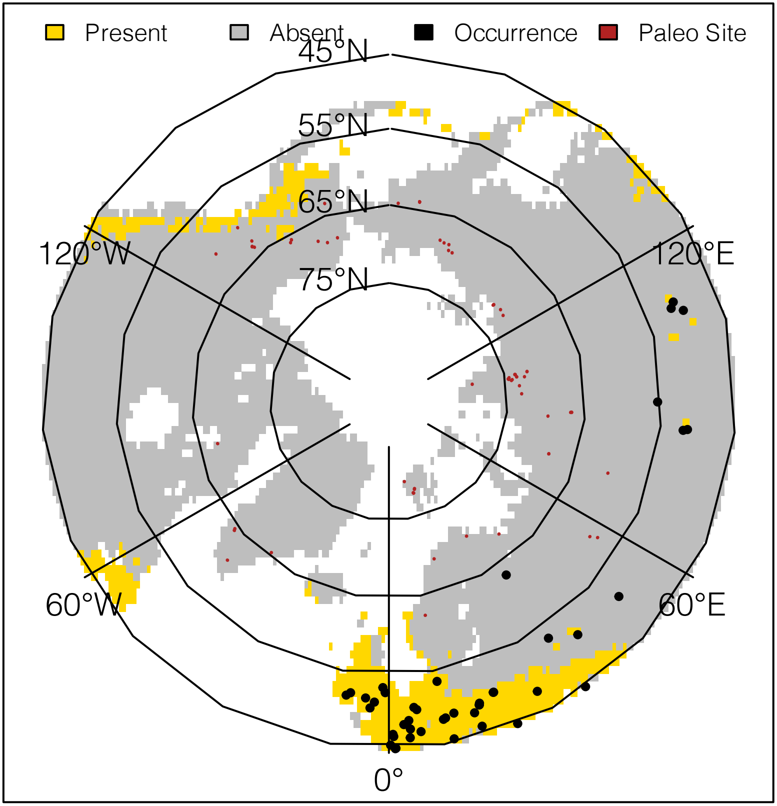

Human Presence/Absence, 42 ka BP

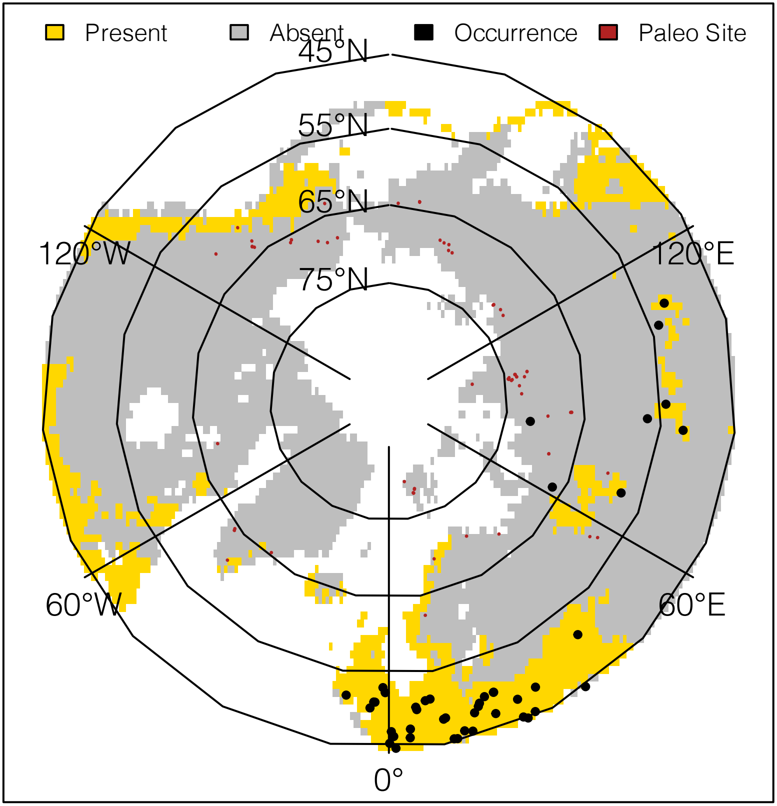

Human Presence/Absence, 44 ka BP

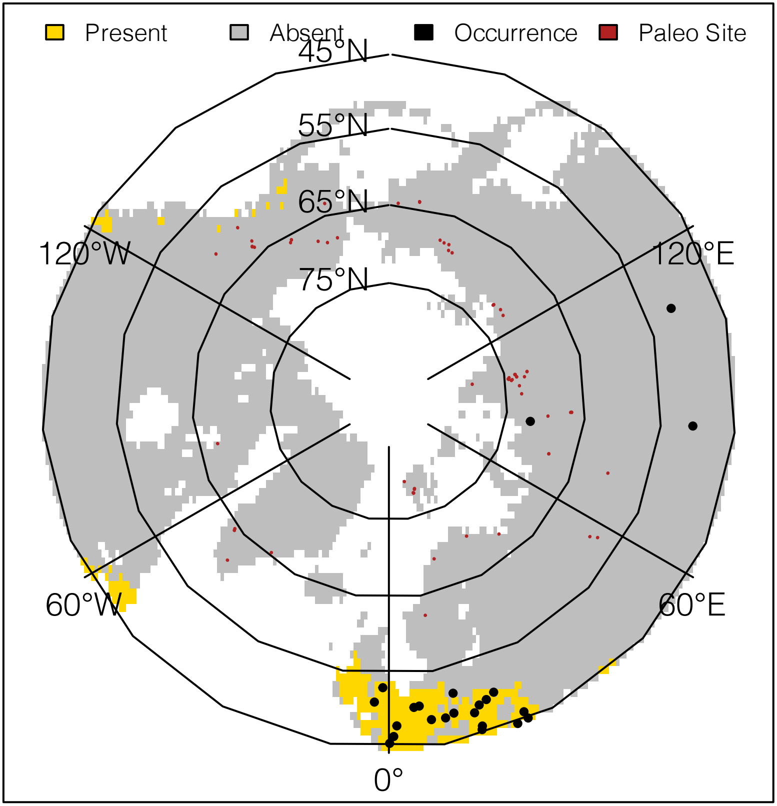

Human Presence/Absence, 46 ka BP

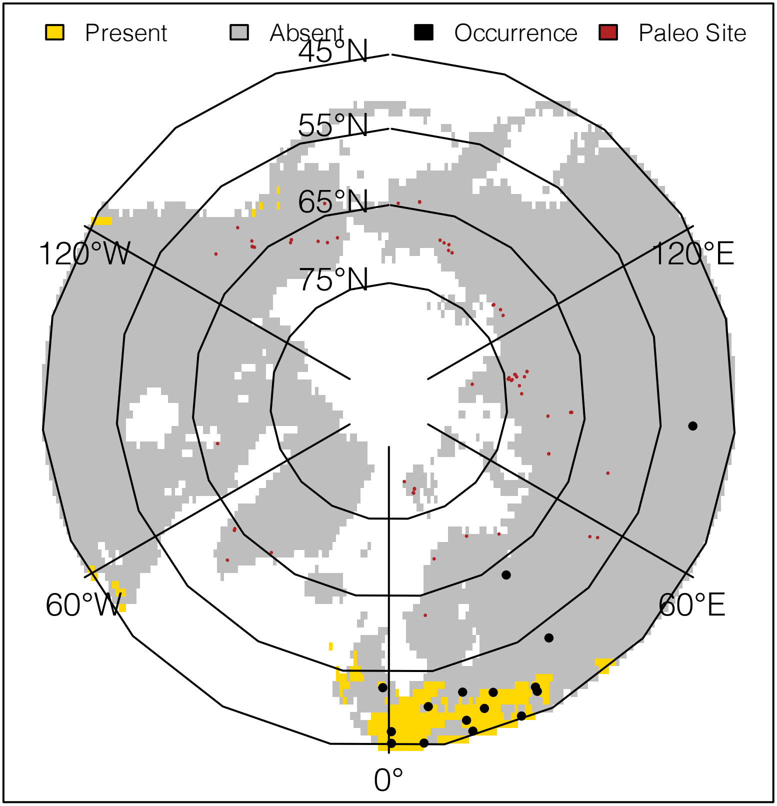

Supplement: Human Presence/Absence — A PDF file showing the modelled environmentally suitable conditions for Palaeolithic human occurrence. [file 41586_2021_4016_MOESM9_ESM.pdf]
